# Delimiting Areas of Endemism through Kernel Interpolation

**DOI:** 10.1371/journal.pone.0116673

**Published:** 2015-01-22

**Authors:** Ubirajara Oliveira, Antonio D. Brescovit, Adalberto J. Santos

**Affiliations:** 1 Departamento de Zoologia, Universidade Federal de Minas Gerais, Belo Horizonte, Minas Gerais, Brasil; 2 Laboratório Especial de Coleções Zoológicas, Instituto Butantan, São Paulo, São Paulo, Brasil; Scientific Research Centre, Slovenian Academy of Sciences and Arts, SLOVENIA

## Abstract

We propose a new approach for identification of areas of endemism, the Geographical Interpolation of Endemism (GIE), based on kernel spatial interpolation. This method differs from others in being independent of grid cells. This new approach is based on estimating the overlap between the distribution of species through a kernel interpolation of centroids of species distribution and areas of influence defined from the distance between the centroid and the farthest point of occurrence of each species. We used this method to delimit areas of endemism of spiders from Brazil. To assess the effectiveness of GIE, we analyzed the same data using Parsimony Analysis of Endemism and NDM and compared the areas identified through each method. The analyses using GIE identified 101 areas of endemism of spiders in Brazil GIE demonstrated to be effective in identifying areas of endemism in multiple scales, with fuzzy edges and supported by more synendemic species than in the other methods. The areas of endemism identified with GIE were generally congruent with those identified for other taxonomic groups, suggesting that common processes can be responsible for the origin and maintenance of these biogeographic units.

## Introduction

The identification of areas of endemism is a most fundamental task of biogeography, since they are considered basic units for historical biogeographic studies [1], as well as for the definition of conservation priorities [2]. However, the delimitation of areas of endemism is frequently difficult, mostly because species do not respond in exactly the same way to factors that could limit their distribution (e.g., [3]). As a consequence, the distribution ranges of species from the same area are not perfectly overlapped, and the borders of areas of endemism can be diffuse [4–6].

The variability in the overlapping of species distribution ranges has influenced not only the concept of areas of endemism, but also the methods proposed for their delimitation [6]. Although the first formal definition of areas of endemism emphasized the complete congruence of species range limits [1], it soon became obvious that this situation would be extremely rare in nature and, consequently, should not be a prerequisite for delimitation of areas of endemism [7]. Hence, both the definition and delimitation of areas of endemism should take into account the degree of overlap between species ranges. Platnick [8] proposed to solve this problem by modifying the original concept of area of endemism, which should be considered as the area within the limits of two or more minimally congruent species ranges. Linder [9] proposed a more inclusive definition, in which areas of endemism are considered the sum of maximally congruent distribution ranges of two or more species. The fact that species do not conform to exactly the same areas of distribution has major implications not only on the definition of areas of endemism, but mainly on their delimitation, since that would require methods to evaluate quantitatively the overlap of species ranges. Several algorithms have been proposed recently and most of them are basically methods for clustering smaller geographic units, usually grid cells disposed over maps. A simple approach adopted by some authors is the measurement of similarity between grid cells, based on the number of shared species, using the Jaccard’s similarity index (e.g., [9]), followed by clustering and delimitation of areas of endemism based on an arbitrary threshold of similarity. However, the most used method of clustering grid cells is an adaptation of cladistic analysis, the Parsimony Analysis of Endemism (PAE), which identifies areas of endemism using a data matrix with grid cells arranged on a map of species distribution as terminals and species as characters [10]. The trees resulting from parsimony analysis of the matrix are then rooted in a hypothetical area where all the species are absent. Areas of endemism are identified on the strict consensus tree as clades supported by at least two endemic species.

Since the modifications proposed by Morrone [10], PAE has been widely used, but also widely criticized, as a tool for the delimitation of areas as endemism [11]. Although PAE is the most widely used method for identification of areas of endemism, many authors have raised questions about its efficiency, especially regarding the use of a hypothetical, all-zero root, which could lead to grouping areas that have fewer species together [12]. Moreover, the performance of PAE to identify areas of endemism may be unsatisfactory, mainly because it can cluster disjoint areas based on widely distributed species [13–15]. Some modifications have been proposed to circumvent these deficiencies (e.g., [16, 17]), but they have not been widely used.

The problems identified in the use of PAE stimulated the proposal of alternative quantitative methods for delimitation of areas of endemism. Two of those methods, Nested Clade Area Analysis [17] and NDM [5, 14] differ markedly from other methods by grouping grid cells according to the number of shared species in a spatially explicit way. Methods like cluster analysis and PAE build groups of grid cells in tree-like diagrams that must be interpreted and projected in a map. Those recent methods delimit groups of cells (not necessarily adjacent) directly in the map, which makes the interpretation of their results much easier.

All methods proposed so far use grid cells as basic units. A caveat of this procedure is that the size of quadrats used as spatial units can affect the results of PAE [18], NDM [5, 14], and possibly of any other similar method. Moreover, neighboring grid cells can be grouped because of a species that occur at the edge of a cell, even if those cells are different in species composition. Due to these problems, Morrone and Escalante [18] argue that the use of ‘somewhat more natural’ areas can help in understanding their historical/ecological relationships. Furthermore, Crother and Murray [6] argue that areas of endemism, as natural units, should present fuzzy edges, something that could not be easily represented by methods dependent on grids.

Given the problems of the methods currently used for identifying areas of endemism, we propose a new approach based on the quantification of the co-occurrence of species, weighted by the distance between the points of distribution records. Unlike all methods already proposed (e.g., [5, 10, 17]), this new approach does not depend on grid cells, it allows the use of occurrence data with gaps and delimits areas of endemism with fuzzy edges. In this study we describe this method and apply it to a database of spiders from Brazil. Additionally, the efficacy of this new method is compared to two methods commonly used to delimit areas of endemism, PAE and NDM.

## Materials and Methods

### Geographic Interpolation of Endemism (GIE)

**Figure 1:**
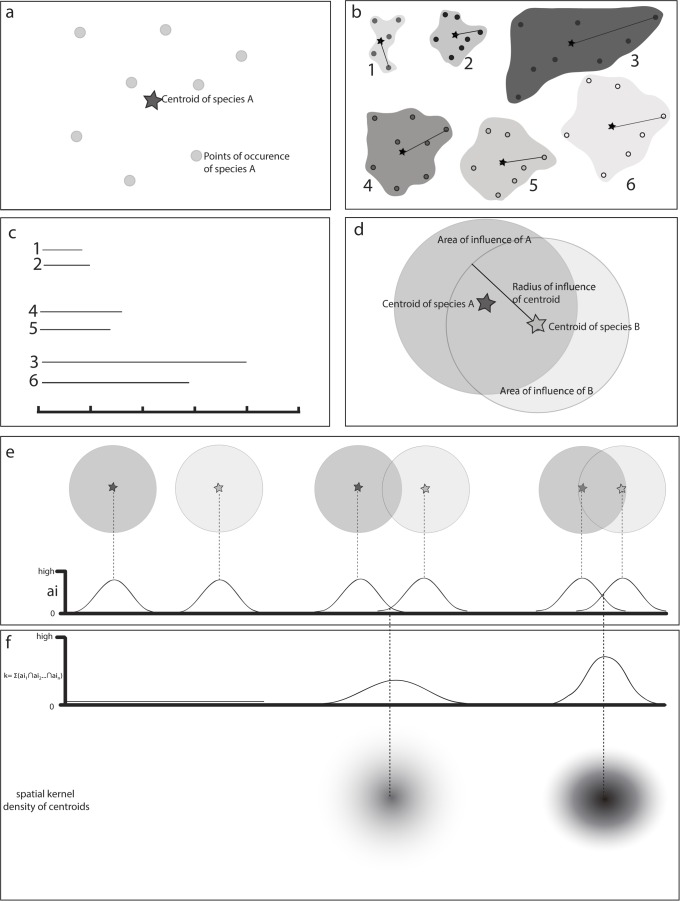
Step-by-step implementation of Geographic Interpolation of Endemism (GIE) analysis. a: a centroid is estimated for the points of occurrence of each species. b: For each species, the distance between the centroid and its farthest point is measured. c: species are organized in groups, according to the distance measured in step b. d: This distance is used to define a circular area of influence around each species centroid. This procedure makes it possible to quantify the overlap between areas of distribution among species. e: The degree of overlap between species areas of influence is measured according to a Gaussian function around each species centroid. f: The density of species on each area of overlap, weighted by the degree of overlapping, is converted into interpolated curves using the kernel interpolation function (at left). These curves can be rasterized for display on maps.

**Figure 2:**
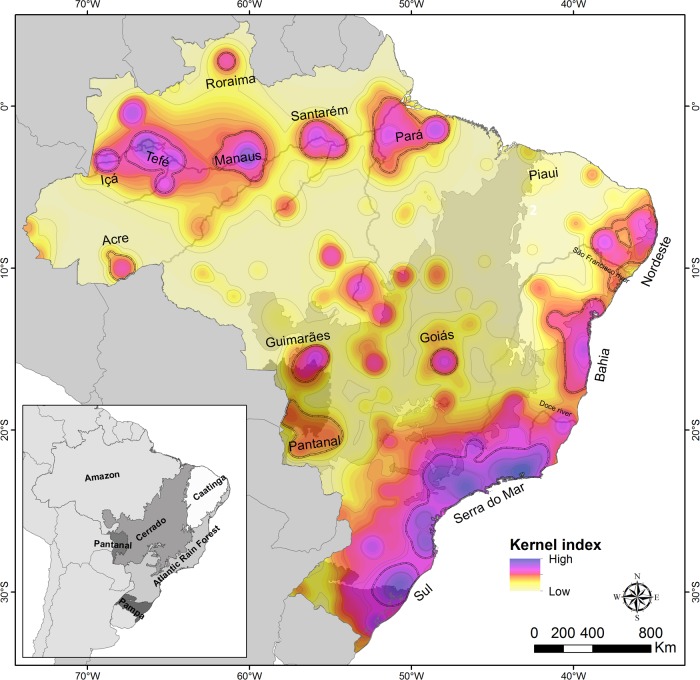
Areas of endemism of spiders in Brazil, identified using GIE. Shaded areas indicate the areas of endemism, dashed lines indicate the major areas of endemism delimited according to the kernel index. The insert shows the Brazilian biomes, discussed in the text.

We propose the use of a kernel interpolation function, a method commonly used in Geographic Information Systems (GIS) analysis and implemented in several GIS software, to delimit areas of endemism. This interpolation method is based on the definition of circular areas of influence around point occurrences of a phenomenon. Within the area of influence, which is usually defined by the user, the influence of the phenomenon decreases from the point to the limits according to a Gaussian function [19]. For instance, in an epidemiological study of Rabies in China, the area of influence around each infected subject reported was defined as the range of subject´s movement, reflecting transmission probability [20]. The kernel density function estimates the density of occurrence of the phenomenon based on the overlap of the areas of influence [19]. Thus, the results are summarized on a map, expressed as a surface that indicates estimated values of point density. In the method proposed here, the distributional overlap between species is estimated through the distance between centroids of each species distribution range. This method can be applied for identifying areas of endemism through the following sequence of procedures:

1. Given a set of occurrence points, the centroid of the distribution of each species is estimated through the arithmetic mean of the latitude and longitude of its points (Fig. 1a).
2. The distance between the centroid and its farthest point of occurrence is measured for each species (Fig. 1b), and this value is used to sort the species into categories of range size (Fig. 1c). The definition of these categories is necessary to define the area of influence of the centroid, as described below, which is a requirement for the kernel index estimation [19].
3. For each category a value of a radius around the centroid is defined, in order to delimit a circular area of influence of each species range (Fig. 1d). This value can be established through the maximum value of distance between the centroid and the farthest point of each category. For example, in this study all species with up to 100 km of distance between the centroid and the farthest point were grouped in the same category, and this value was defined as the radius around the centroid of all species in the category. The area of influence of each species is a generalization of its distribution range, and thus it must be defined as realistically as possible. Grouping species with differently sized distribution ranges (e.g., species with up to 50 km together with those up to 200 km of maximum distance between the centroid and its farthest point) could result in the overestimation of the range of the more restricted species.
4. For each category, the overlap between the areas of influence of the species (Fig. 1d) is estimated by the kernel algorithm. The area of influence of each species is expressed as a value that decreases from the centroid to the limits of the circular area according to a Gaussian function (Fig. 1e). The overlap between the areas of influence is estimated through the sum of the values of the overlapping portion, resulting in the kernel index (k). Consequently, the kernel index varies spatially according to the sum of the values of each area of influence, generating a series of overlapped Gaussian curves (Fig. 1e, f). These curves are rasterized, generating a map of density of overlap of areas of influence of species (Fig. 1f), and the kernel index is an indicator of the degree of species distribution overlap.
5. The results of steps 3 and 4 can be expressed separately for each category or assembled in a consensus map of areas of endemism (Fig. 2). The spatial variation of the kernel index can be displayed with color hues or with isolines (level curves) representing equal values of the index (Fig. 2). The latter option is useful to show the hierarchy between areas of endemism.

To perform these procedures, a software that calculates the area of influence of each species, as well as an ArcGIS toolbox to implement the method described above, are available in the S1 File in Supporting Information.

### Applying GIE to Brazilian spiders

We applied the method described above to delimit areas of endemism of spiders in Brazil using a database of all published distribution records of species described between 1767 and 2013 and two online databases: GBIF [21] and speciesLink [22]. The database includes 3,425 species distributed in 25,072 records (meaning at least one individual of a species collected) and 3,787 localities. About 40% of the species were represented only by single records, 45% had between 2 and 15 records, 10% had between 16 and 60 records and only 2% of the species showed more than 100 records (S1 Fig.). All coordinates reported in the literature and online databases were checked using the ArcGIS software and vector layers of the political boundaries of Brazil to determine whether they actually fit the municipalities and states mentioned in the original data. Of these, 59% of the records were in the correct coordinates. The records that presented incorrect coordinates were georeferenced, as well as records that had no coordinates originally reported. The georeferencing was based on gazetteers and online databases, and 32% of the records were georeferenced in specific localities and only 8% were georeferenced by the location of the municipality. The species taxonomy follows Platnick [23], and species considered *nomina dubia* were excluded from the analyses. These same records and species were used in the comparative analysis with PAE and NDM. For analysis through GIE the species were classified in nine groups, according to the distance between the centroid and the farthest point: up to 50 km, 51–200 km, 201–400 km, 401–600 km, 601–800 km, 801–1,000 km, 1,001–1,500 km, 1,501–2,000 km and between 2,001 and 3,299 km. Since the definition of these classes can affect the number and location of the areas of endemism, we repeated the analysis with two other categorization schemes: a more inclusive classification with five categories (up to 50 km, 51–400 km, 401–600 km, 601–800 km, 801–3,299 km) and other less inclusive, with 18 categories (up to 25 km, 26–50 km, 51–100 km, 101–200 km, 201–300 km, 301–400 km, 401–500 km, 501–600 km, 601–700 km, 701–800 km, 801–900 km, 901–1,000 km, 1,001–1,300 km, 1,301–1,600 km, 1,601–1,750 km, 1,751–2,000 km, 2,001–2,500 km, 2,501–3,299 km). To compare results, we used Pearson correlation. To generate the consensus map of areas of endemism, the values of the kernel index of each category were standardized between 0 and 1 before assembling the maps. The number of records of a species can affect the position of its distributional centroid, consequently influencing the estimate of the overlap between species in GIE. We estimated this effect through a rarefaction procedure, in which we randomly removed 10, 20 and 30% of the occurrence points and measured the mean deviation of the centroids of the species in 100 randomizations.

The analysis with PAE was based on a presence/absence matrix of spider species over a grid with 168 2×2° cells, completely covering the Brazilian territory (S2 File). As the size of the grid cells can influence the results, we tested several cell sizes (0.5° to 5°) and used the size that allowed the identification of more areas of endemism. The matrix was analyzed through the software TNT [24], based on twenty trees generated by random-addition sequences, followed by TBR Branch Swapping, retaining 20 trees per replicate. The shortest trees obtained were submitted to an additional round of TBR to assure global optimum was found. The trees obtained were rooted in a hypothetical cell with all taxa absent. The areas of endemism were delimited from clades unambiguously supported by at least one non-homoplastic species occurrence, identified in the strict consensus tree.

The same database was analyzed by NDM using the program VNDM [25] (matrix in S3 File), with 2×2° cells. Search factors were set to retain areas with scores equal or above one and presenting one or more endemic species. The search was repeated 100 times, keeping overlapping areas only if 90% of the species in each area are unique. We did not use any parameters to assume the presence of the species in places where they have not been recorded. The results were summarized through the procedure “consensus flexible areas of endemism”, gathering areas that share at least 40% of their endemic species (for more details see [26]).

The results obtained in GIE were compared to results from PAE and NDM through the number of synendemic species (endemic species occurring together in a given area) that supported areas of endemism spatially congruent between methods. In these cases, we consider that areas identified with the highest number of endemic species should indicate a better fit between the boundaries of the area of endemism and the distribution of its species. We also compared the number of areas identified and visually evaluated the overlap between areas generated by each method. Both NDM and GIE show indexes to quantify the support of each area of endemism, so we analyzed the correlation between the score of the areas obtained through these methods using Pearson correlation analysis in Past 1.95 [27]. This analysis was based on values from grid cells of NDM, so the same grid was overlapped to GIE consensus map and 10 random points were used to estimate the average value of the kernel index for each cell.

## Results

We identified 101 areas of endemism of spiders in Brazil (Figs. 2 and S2 and S1 Table), each supported by a minimum of one and a maximum of 400 endemic species (*Serra do Mar*in Fig. 2 and area A4 in S2 Fig.). All areas are well separated geographically, with clear boundaries between them. Eastern Brazil, including the Atlantic Forest and the Pampas, is divided in four major areas of endemism, subdivided into 17 smaller areas. These areas have between 1 and 400 endemic species. The largest discontinuity observed among areas of endemism of this region is located around the Doce River (Fig. 2). The Amazon region has 46 areas of endemism, each containing from one to 46 endemic species (Fig. 2 and S2 Fig.) These areas are relatively isolated small islands, mostly aligned along the Amazon River (Fig. 2). As for other Brazilian biomes, we identified 19 areas of endemism in the Cerrado, five in the Pantanal and seven in the Caatinga.

The results of the GIE using different categorization schemes showed little variation in the main areas of endemism identified. This was demonstrated by the high correlation between the maps of the three analyses of GIE (r> 0.91, S3 Fig.). The most remarkable difference between the three analyses is the presence of micro areas of endemism in 18-classes analysis (S3 Fig.). Regarding the effect of missing points on the estimation of species centroid, the rarefaction procedure showed that most species centroids moved less than 50 km on average. However, the number of species whose centroids moved on average more than 50 km increases with the number of missing points (S4 Fig.). Moreover, the variation in centroid movement between rarefaction replicates was relatively low. More than 50% the species showed variance between rarefaction replicates below 50 km, regardless of the proportion of missing points (S4 Fig.).

**Figure 3:**
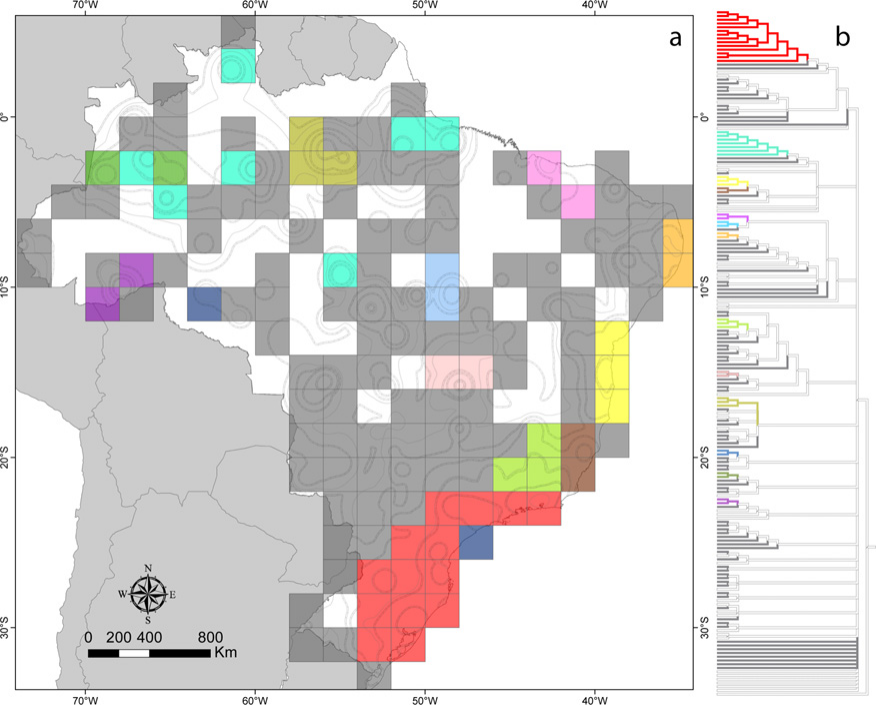
Areas of endemism of spiders in Brazil identified using PAE. a: Areas of endemism of spiders in Brazil identified using PAE, superimposed to the isolines of GIE’s areas of endemism depicted in Fig. 2. Colored polygons indicate areas of endemism composed by more than one grid cell, dark gray quadrats indicate areas that were restricted to a single, not clustered grid cell. b: PAE consensus cladogram obtained through parsimony analysis of the whole spider dataset. Colored branches indicate areas of endemism formed by more than two grid cells (same colors as the corresponding location on the map); gray terminal branches indicate areas that were restricted to a single, not clustered cell; white terminal branches indicate grid cells that did not form areas of endemism due to the absence of at least one endemic species.

The PAE analysis resulted in 99,999 equally parsimonious trees with 9,508 steps, resulting in a poorly resolved strict consensus tree (Fig. 3, S5 Fig. for more details). Twelve areas of endemism in the consensus tree have two or more grid cells, and 102 not clustered grid cells with one or more endemic species (Fig. 3). The Atlantic Forest included six major areas of endemism, four of these within GIE’s *Serra do Mar* (Fig. 3). We identified five major areas of endemism in the Amazon, one of them quite congruent with GIE’s *Içá*and *Tefé*. Three of these areas brought together disjoint cells.

**Figure 4:**
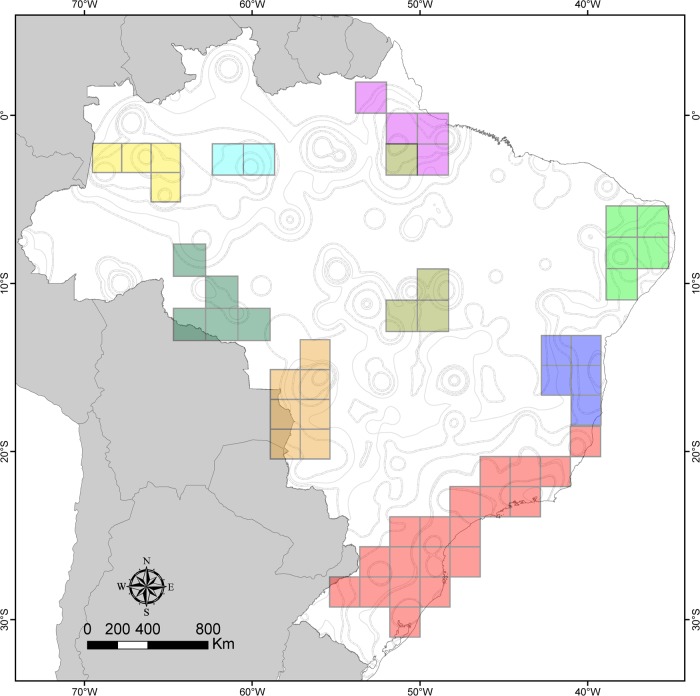
Areas of endemism of spiders in Brazil identified using NDM. Map showing some contiguous consensus areas of endemism identified by NDM analysis, indicated by colours.

The NDM analysis identified 384 candidate areas of endemism, which were summarized in 106 consensus areas [26] (see S1 Appendix). These areas comprised scores between 2 and 49.79 and included between two and 155 synendemic species. The largest continuous areas of consensus identified were similar to the areas delimited with GIE (Fig. 4), as *Serra do Mar* + *Sul, Bahia, Nordeste, Pantanal* + *Guimarães, Tefé + Içá, Manaus* and *Pará*. Additionally, 76 areas of endemism were formed by groups of disjoint grid cells (S1 Appendix). However, these areas had low scores. The NDM and GIE scores were positively and significantly correlated (r = 0.63, p <0.01).

## Discussion

### Identification of areas of endemism by Geographic Interpolation of Endemism (GIE)

**Figure 5:**
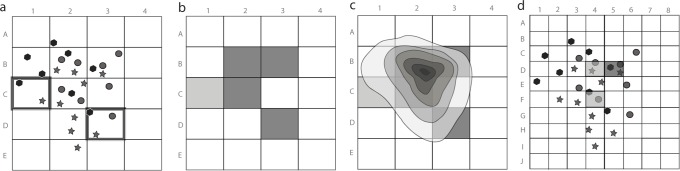
The problems of using grid cells for delimitation of areas of endemism, illustrated by a hypothetical example. a: Points of occurrence of three species (illustrated by different symbols) overlapped by a grid of large cells. The presence of an occurrence point inside a cell, even in its borders (as in the highlighted cells), is interpreted as if the species is present in the whole cell. b: As a consequence, areas of endemism delimited from the grid are overestimated. c: GIE optimization, which does not depend on grid cells, estimates areas of endemism with contours closer to the actual overlap between species distribution. d: Using a grid with smaller cells could generate more realistic areas of endemism, but it makes it more difficult to detect the overlap between the species distribution.

GIE allows the identification of areas of endemism at multiple scales and with fuzzy edges, which is consistent with the current concept of areas of endemism [5, 6, 14]. This is possible because GIE uses interpolation of centroids of species distribution to estimate the distributional overlap between them. The methods traditionally used for delimitation of areas of endemism (e.g., [5, 10, 17]) are based on plotting the occurrence of species in grid cells. This procedure is based on Boolean logic, in which the presence of a single point of occurrence of a species in one grid cell is interpreted as if the species occurs all over that cell (even if the species occurs exactly over an edge of the cell). Thus, the overlap between two species are considered complete when they co-occur in the same cell, even when they are represented by only one point per species, in opposite edges of the cell (Fig. 5). GIE solves this problem by weighting the extent of overlap by the proximity of centroids of species distribution ranges using the area of influence of species. The area of influence is represented by a Gaussian curve with values that decrease from the centroid. Thus, when the areas of influence are summed, the sites with high degree of overlap between the distributions of species would show higher values of the kernel index. The resultant areas of endemism encompass the points of occurrence more accurately, avoiding generalizations made by grid cells.

Another problem with other methods of delimitation of areas of endemism is the choice of the size of the grid cells. The use of small grid cells may hamper the grouping of cells in PAE [18], a problem that possibly occurs in other methods based on grids [5], since smaller cells reduce the possibility of species overlap (Fig. 5a, d). Additionally, methods that use grid cells are limited in scale by the size of the cells. This limitation occurs because it is not possible to identify an area of endemism on a scale smaller than the size of the grid cell, since this is the basic unit of analysis. This could be solved by using smaller cells, but that could reduce the overlap between the species distributions to a point in which there will be no grouping of cells. GIE has no such limitation, since the size of the areas of endemism depend mostly on the species distribution data. However, to implement a GIE analysis the user must define areas of influence around the centroids through the delimitation of categories of distribution area. Although this decision could affect the result of the analysis, we demonstrate here that, at least for the database analysed in this study, the number of classes did not change significantly the number and configuration of the areas of endemism (S3 Fig.). It is worth mentioning that the choice of a categorization scheme for species distribution areas is different from choosing the grid cell size in one fundamental aspect, which is the fact that the GIE analysis can embrace several size classes, conforming to the species distribution areas. Analysis with methods based on grids can be executed only with a same cell size for all species, both narrowly endemic and widely distributed. On the other hand, the categorization employed in the GIE increases the probability of detecting maximum co-occurrence between species within each size class, before summarizing global results in a consensus map. We recommend that future users explore different species distribution categorization schemes, to evaluate the effect of this decision on their results.

The use of grid cells can also be particularly sensitive to georeferencing issues, since even a small mistake in the geographic coordinates can move a point of occurrence to a different cell. In GIE this problem is less severe, since the records of occurrence of the species will be summarized by centroids, so an error in one point of occurrence will not change dramatically the position of the centroid. However, the position of the centroid in a map is dependent on the number and position of occurrence points, and thus can be affected by sampling deficiency. The rarefaction procedure implemented on the Brazilian spiders database demonstrated that the removal of occurrence points cause moderate centroid displacement for most species (S4 Fig.). Considering the spatial scale of this analysis, in which most areas of endemism have hundreds of kilometers (Fig. 2), it does not seem to us that a moderate shift in species centroids would change significantly the configuration of the areas of endemism obtained. Nevertheless, the effect of sampling deficiency should be evaluated quantitatively not only for the GIE, but also for all other methods of delimitation of areas of endemism. Until this important aspect is evaluated, the users of GIE (or other methods) should consider restricting their analysis to species with a minimum number of distribution records.

Another interesting aspect of GIE is that it displays directly on the map smaller areas of endemism contained in more inclusive areas of endemism (the Russian nesting dolls analogy of Crother and Murray [6]), keeping the hierarchy between these areas. The definition of operational areas of endemism, for whatever objective, is a matter of spatial scale [28]. For instance, in the results of this study the areas *Manaus, Nordeste* and *Serra do Mar* contain smaller areas within their boundaries (Fig. 2), which could be treated as independent areas of endemism according to the objectives of the study. Different studies require areas with clear-cut borders, which must be defined over the diffuse areas of endemism. GIE allows it to be done in a more objective and reproducible way using isolines. Other methods, such as PAE, also allow the establishment of areas of endemism at multiple scales [6]. However, in GIE this is a direct consequence of the spatial distribution of the species, while in PAE this is a result of the number of synendemic species in grid cells.

The results obtained by PAE and GIE in the current study differed mainly in the shape of the areas of endemism and the number of endemic species identified in each area, while NDM results were relatively similar to those from GIE. Some areas of endemism identified with GIE emerged from PAE as only one grid cell (grey cells in Fig. 3). For instance, the areas *Pantanal* and *Nordeste* were represented by more than three non-grouped grid cells in PAE. Additionally, both PAE and NDM failed to identify the *Piauí* area, because the points of occurrence of the species present in that area are separated in different cells. NDM also failed to identify other areas, as *Roraima* and *Acre*, probably because they had low scores in the analysis, resulting in their exclusion from the consensus. Thus, compared to other methods GIE was able to identify areas of endemism with more synendemic species and produced results with greater spatial resolution.

Another important characteristic of GIE is the possibility to separately analyse data of species with different distribution extents, enabling the delimitation of areas of endemism at different spatial scales. This could be important because more restricted species can have areas of endemism located with only partial overlap with those estimated for widely distributed species. The results obtained from the analysis of different range size categories can be summarised through different procedures, according to specific research objectives. For instance, in this study species were divided into seven categories according to the extent of its distribution (estimated by the distance between the centroid and the farthest point of occurrence), and then the results were summarized in a consensus map. The consensus map can be generated by summing the values of the kernel index of different spatial scales, but this procedure gives more weight to scales with more species. This can be avoided by standardizing the values of the kernel index between 0 and 1 in each range size category before assembling the consensus, as done in this study. However, after standardizing, it is possible to insert a multiplication factor to weight a scale considered more relevant or reliable. These strategies will not change significantly the isolines of the resultant areas of endemism, but can change the difference in values of the kernel index between levels within the same area of endemism. Thus, some areas may be more evident than others, depending on the consensus criterion. This index can be used as an objective criterion to define a threshold for delimitation of operational areas. Furthermore, the choice of consensus criteria may be particularly important in some situations, like in definition of conservation priorities. In these cases, it may be interesting to emphasize scales that present species with more restricted distribution.

As described above, the estimation of the overlap between species distribution ranges through the kernel index requires a generalization of each species distribution range in a circular area of influence around the centroid. This procedure would not be a major limitation, except in the case of species whose distribution points describe an elongated polygon and/or species with disjoint distributions. In these cases, the circular area of influence would include extensive areas without actual distribution records, and the overlap with other species’ areas of influence would be overestimated. Species with elongated distribution does not necessarily represent a problem, as demonstrated by the results of our analysis of Brazilian spiders. Our database includes species with elongated distribution that were assembled in elongated areas of endemism (S6 Fig.). A potentially problematic situation, not observed in our data, would be species with elongated and perfectly congruent distribution. In that case, the GIE would correctly define an area of endemism for these species, but it would be incorrectly represented as a circular area. Although we presume this would be a rare pattern, it should be evaluated by future users. Species of elongated distribution can be common in some specific situations, like in taxa restricted to mountain chains or rivers. In situations like these, the maps with estimated areas of endemism can be clipped according to the habitats of interest, removing areas with overlap, but without distribution records. However, it seems there is no apparent solution to cases of continental species with elongated distribution polygons, without an associated habitat. Thus, users should evaluate whether species distribution polygons are predominantly elongated before using the GIE. This can be done using a function available on the ArcGis toolbox on S1 File

Regarding species with disjoint distribution, a possible solution would be to treat each cluster of distribution points separately, calculating a centroid for each one. In these cases, it is possible that the same species would contribute to support different areas of endemism. On the other hand, it is worth mentioning that diagnosing a species distribution as disjoint could be particularly difficult, considering the possibility that sampling gaps could create a false disjoint appearance. Thereby, it is recommended that the shape of the distribution ranges of the species of interest be examined prior to analysis with the GIE, to deal with species with distribution ranges that could not be reliably generalized as a circular area of influence.

The results of this study suggest that GIE is a method consistent with theoretical expectations regarding areas of endemism, identifying them in multiple scales and without grid cells. In this study, GIE was more efficient than PAE and NDM, identifying areas supported by more endemic species and with boundaries that better fit the distribution of species. In addition, GIE is simple and easy to implement using traditional commercial software such as ArcGIS or free software like Quantum GIS (available in: http://www.qgis.org/en/site/). We provide in S1 File a toolbox built for ArcGIS that enables the rapid identification of areas of endemism from a georeferenced database.

### Areas of endemism of spiders of Brazil

In this study we successfully identified a great number of areas of endemism for spiders in Brazil, some of them supported by many synendemic species. Additionally, as will be emphasized below, the areas of endemism identified were consistent with those proposed for other taxa [29–31], indicating that this pattern is probably general for various taxonomic groups. This is plausible since the process of speciation in different groups may be influenced by common factors in these areas [32–35].

The coast of Brazil, including the Atlantic Rain Forest and part of the transition between this biome and a southern grassland biome (the Pampas), was divided into four major areas of endemism. Their limits were consistent with those observed in other studies delimiting areas of endemism [29–31, 36] and phylogeographic [33, 34, 37–43] and descriptive biogeographic studies [44, 45] with different taxonomic groups. This congruence may indicate that common processes may be limiting several lineages of organisms, at species or population levels, to these areas. These areas of endemism are separated by gaps that have no records of endemic species, but only spider species with distribution ranges larger than 300 km, which is above the amplitude of the areas of endemism of the Atlantic Rain Forest. The eastern coast biomes of Brazil are the better known regarding its spider fauna. This region includes the largest number of spider occurrence records within Brazil, and composes a continuous stretch of relatively well sampled localities [46]. Thus, the discontinuity between the areas of endemism does not represent sampling artifacts, but is possibly related to natural factors that limit spider distribution.

Some studies suggest that rivers act as dispersal barriers in the Brazilian coast (e.g., [38]). Two major rivers flowing into the coast of Brazil, the southeastern Doce River and northeastern São Francisco River, both previously suggested as dispersal barriers, mark major gaps between the areas of endemism of Atlantic Rain Forest. However, as most spiders have high dispersal capacity by ballooning [47], it is unlikely that these rivers may actually represent dispersal barriers to these animals. On the other hand, these gaps coincide with climatically unstable areas, where forest probably retracted during the last glacial maximum [34]. This suggests that the Atlantic forest areas of endemism observed in this study are related to forest refuges, which were stable during past climatic oscillations(see [34, 40, 48, 49]).

The results of this study also identified nested areas of endemism on the Brazilian coast. For instance, the *Serra do Mar* includes several smaller areas at a smaller spatial scale, both consistent with studies based on other taxa [39, 41, 50]. The divergence between these areas is probably related to Tertiary geomorphological and neotectonic events [51]. At a larger spatial scale, the *Serra do Mar* is connected to the area *Sul*, a biogeographical connection also described for primate species [52]. Although the *Sul* encompasses part of the Pampa, it is centered in the ecotone between this biome and the Atlantic Rainforest, which can be related to the large number of common species between these areas.

Different from the Atlantic Forest, other Brazilian biomes show large deficiencies in spider sampling [46]. This hampers the interpretation of the discontinuities between areas of endemism delimited in this study, because these gaps usually coincide with poorly sampled areas. For instance, areas of endemism were identified in the Amazon, some of them relatively well supported. However, the limits of these areas should be taken with caution, given that the entire Amazon has a low density of species occurrence points and a historical concentration of sampling effort mostly in small regions [46]. A similar problem was observed for Amazonian plants [53, 54] and it was suggested that this could lead to the identification of false areas of endemism that actually represent sampling hot spots [53]. However, the large number of spider species unique to each of the areas delimited here, and the small number of species shared between them, is a strong indication of the existence of areas of endemism in these regions, since areas relatively close as *Içá, Tefé, Manaus, Santarém* and *Pará* have a large number of synendemic species. On the other hand, the sampling bias in the Amazon can prevent the identification of areas of endemism in poorly sampled regions, as well as the identification of the actual limits between the areas already identified. Therefore, the boundaries of the currently recognized areas may undergo major changes with the addition of new information on species distribution, which can be obtained with future sampling effort in poorly explored regions.

Despite sampling incompleteness, the Amazonian areas of endemism identified in this study are partially congruent with areas proposed in other studies [52, 55, 56]. GIE’s areas *Acre, Tefé* and *Pará* are contained within the boundaries of Cracraft’s [56] Inambari, Imemberi and Xingu, respectively. In addition, the area *Pará* coincides partially with the area Belém [56]. However, GIE’s areas *Manaus* and *Santarém* are each located over the borders of three areas of endemism proposed earlier [55, 56]. This incongruence is probably related to the fact that rivers, which are usually suggested as the main barriers delimiting Amazonian areas of endemism, could not be effective dispersal barriers for most spiders, as mentioned above. However, the rivers-as-barriers hypothesis postulates that the Quaternary forest contraction led to the current patterns of endemism, which were maintained by rivers operating as contemporaneous dispersal barriers [57]. Thus, even if rivers are not effective barriers to spiders, it is possible that their distribution was influenced by these climate-driven forest contractions. In addition, these areas are partly congruent with the geological events that occurred in the Miocene in the Amazon basin ([58–60] and references therein). However since these events are very old, and considering spiders high dispersal capacity, it is difficult to determine whether these events could have shaped the current spider distribution patterns. In summary, these results suggest that historical or ecological factors influence the distribution of spiders in Amazonia, but a complete understanding of this would depend on higher sampling effort, as well as on phylogenetic, phylogeographic and possibly natural history studies on Amazonian spider species.

The interpretation of our results on Brazilian non-wet forest biomes are hampered by the scarcity of spider sampling in these areas. These biomes are composed of a large savanna (the Cerrado), a swampy lowland (Pantanal) and a seasonally dry tropical forest (Caatinga). The areas of endemism delimited in these biomes coincide with highly sampled spots, surrounded by large sampling gaps [46]. Additionally, unlike Amazonian areas of endemism, the areas delimited in these biomes are supported by few exclusive species. The Cerrado main areas of endemism coincide with limits of floristic areas proposed by Bridgewater *et al.* [61], which are based on floristic similarity. However, as there are large sampling effort gaps in the Cerrado, more samples and biogeographic studies are needed for a complete description of its biogeographic structure. The Pantanal showed smaller areas of endemism, with few exclusive species in an area of transition with the Cerrado. This biome is considered as relatively homogeneous, harboring few endemic species [62]. Although our results could suggest a southern Cerrado-Pantanal area of endemism, we think more data are necessary to understand the composition of this biome. This recommendation can be extended to the Caatinga, which is among the lesser known Brazilian biomes [63], including its spider fauna [46].

In conclusion, despite sampling deficiencies we identified several areas of endemism for spiders in Brazil, most of them congruent with biogeographic units obtained for other taxonomic groups. This also indicates that, in spite of the supposed high dispersion capacities of some groups [47, 64], spiders, especially those with lower dispersal capacity, can be influenced by historical and ecological factors, showing varying degrees of endemism [65–67]. Thus, they can be used as biogeographic models. The new method introduced in this study (GIE) demonstrated to be effective in identifying areas of endemism at multiple scales and with fuzzy edges, which is consistent with how they are expected to occur in nature [6]. Finally, this method was also useful to identify possible sampling problems, which could often be less noticeable in other methods.

## Supporting Information

S1 Appendix NDM areas of endemism. Consensus areas of endemism identified by NDM for spiders in Brazil, and their supporting species and scores.
(PDF)

S1 File Software for identification of areas of endemism by GIE method. ArcGIS toolbox for Geographic interpolation of Endemism (GIE) and software “Geographic distance for GIE”, for calculation of distance between centroid and the farthest point of occurrence for each species.
(ZIP)

S2 File Matrix used for PAE analysis. (SS)

S3 File Matrix used for NDM analysis. (XYD)

S1 Fig Number of records per species in the Brazilian spiders database. (TIF)

S2 Fig Areas of endemism of spiders of Brazil, identified by GIE in differing spatial scales. Text in black indicates the area code in S1 Table. White numbers indicate the number of endemic species in each area of endemism.
(TIF)

S3 Fig Areas of endemism of spiders of Brazil identified by GIE using different ranges size categorization schemes. a: with five classes of range size; b: nine classes of range size and c: 18 classes of range size. Numbers indicates Pearson correlation (r) between maps.
(TIF)

S4 Fig Centroid displacement in analysis of occurrence point rarefaction. Graphs in the same line represent results of analyses removing 10, 20 and 30% of species records. Blue bars indicate frequency distribution of species in classes of average centroid displacement within 100 rarefaction replicates. Pink bars indicate frequency distribution of species in classes of variance of centroid displacement.
(TIF)

S5 Fig Tree of PAE analysis of spiders of Brazil. Numbers indicate endemic species within of grid cells (terminals) and groups of grid cells (branches). colours are the same as in Fig. 3. White branches correspond to terminals without exclusive species.
(TIF)

S6 Fig Examples of elongated areas of endemism in GIE. Elongated areas of endemism identified by GIE for spiders in Brazil. Species indicated by symbols present elongated distribution.
(TIF)

S1 Table Synendemic species of areas of endemism in GIE analysis. Synendemic species of spiders supporting each area of endemism, as depicted in S2 Fig.
(DOCX)
