# Supplementary material for: Delimiting Areas of Endemism through Kernel Interpolation
Source: PLoS One. 2015 Jan 22;10(1):e0116673. doi: 10.1371/journal.pone.0116673 (PMC4303434; doi:10.1371/journal.pone.0116673)
Supplement: S1 Appendix — Consensus areas of endemism identified by NDM for spiders in Brazil, and their supporting species and scores. (PDF) [file pone.0116673.s001.pdf]

Consensus area 0 of 106 (from 28 areas; max. values)

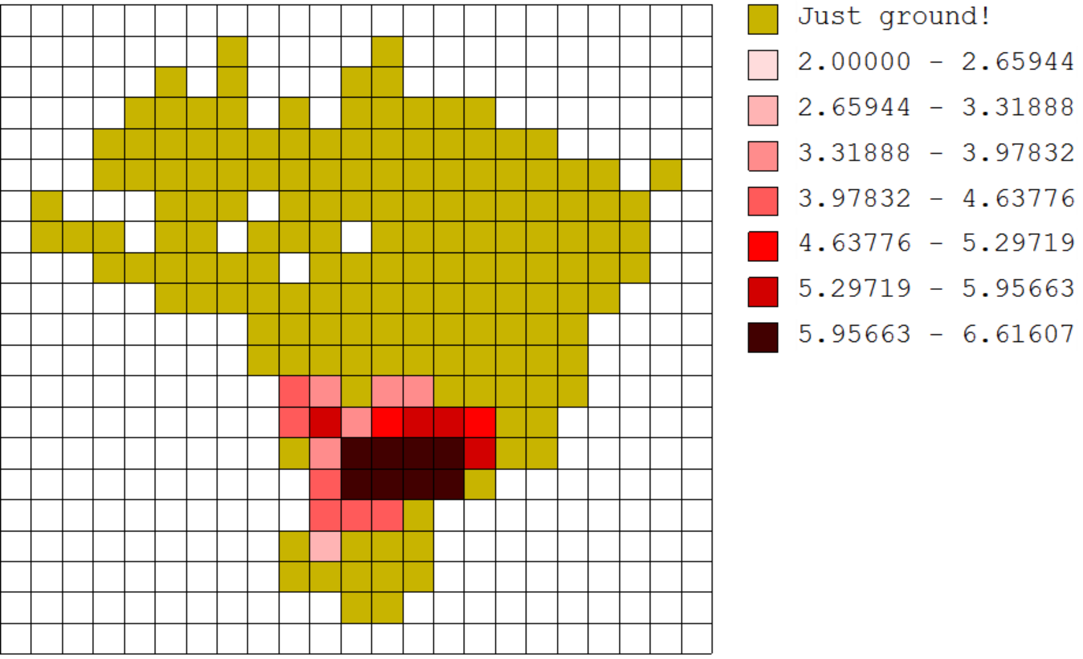

67 species give score:

Amazonepeira_herrera (0.000-0.900)
Anapis_castilla (0.000-0.700)
Anyphaenoides pacifica (0.000-0.700)
Aysha clarovittata (0.000-0.607)
Aysha_guarapuava (0.000-0.513)
Aysha_marinonii (0.000-0.750)
Breda bistriata (0.000-0.800)
Centroctenus acara (0.000-0.700)
Centroctenus miriuma (0.000-0.613)
Corinna colombo (0.000-0.548)
Corinna_recurva (0.000-0.700)
Ctenus_manauara (0.000-0.700)
Cyclosa vieirae (0.000-0.613)
Dolichognatha ducke (0.000-0.700)
Dyrines_ducke (0.000-0.700)
Encyosaccus_sexmaculatus (0.000-0.700)
Ephebopus uatuman (0.000-0.700)
Epicratinus amazonicus (0.000-0.800)
Fageia_clara (0.000-0.700)
Gelanor_heraldicus (0.000-0.700)
Hingstepeira dimona (0.000-0.800)
Hingstepeira folisecens (0.000-0.800)
Hypaeus miles (0.000-0.700)
Hypaeus triplagiatus (0.000-0.700)
Hypognatha_colosso (0.000-0.700)
Isoctenus_strandi (0.000-0.637)
Litoporus dimona (0.000-0.800)
Lygromma gasnieri (0.000-0.800)
Lygromma_huberti (0.000-0.700)
Mangora_mamiraua (0.000-0.700)
Mangora nonoai (0.000-0.667)
Mangora sumauma (0.000-0.900)
Metazygia_uma (0.000-0.700)
Micrathena_coca (0.000-0.787)
Micrathena embira (0.000-0.800)
Myrmecotypus olympus (0.000-0.700)
Neoctenus_eximius (0.000-0.700)
Nhandu_carapoensis (0.000-0.350)
Ochyrocera hamadryas (0.000-0.700)
Olios plumipes (0.000-0.700)
Pachomius sextus (0.000-0.700)
Parachemmis manauara (0.000-0.700)
Peucetia_macroglossa (0.000-0.675)
Phiale_tristis (0.000-0.630)
Psalistopoides fulvimanus (0.000-0.585)
Rhoicinus urucu (0.000-0.700)
Scytodes_balbina (0.000-0.700)
Scytodes_martiusi (0.000-0.700)
Scytodes tyaia (0.000-0.560)
Scytodes ytu (0.000-0.809)
Selenops_lavillai (0.000-0.700)
Stethorrhagus_lupulus (0.000-0.700)
Synotaxus waiwai (0.000-0.700)
Thymoites melloleitaoni (0.000-0.662)
Tmarus caxambuensis (0.000-0.700)
Trachelopachys caviunae (0.000-0.667)
Vitalius_longisternalis (0.000-0.594)
Vitalius_paranaensis (0.000-0.792)
Vitalius vellutinus (0.000-0.900)
Zimiromus kleini (0.000-0.700)
Zimiromus_syenus (0.000-0.700)
Actinopus_paranesis (0.000-0.667)
Berlandiella robertae (0.000-0.671)
Breda nanica (0.000-0.700)
Catanduba_tuskae (0.000-0.750)
Micrathena_abrahami (0.000-0.700)
Neoxyphinus petrogoblin (0.000-0.800)

Consensus area 1 of 106 (from 51 areas; max. values)

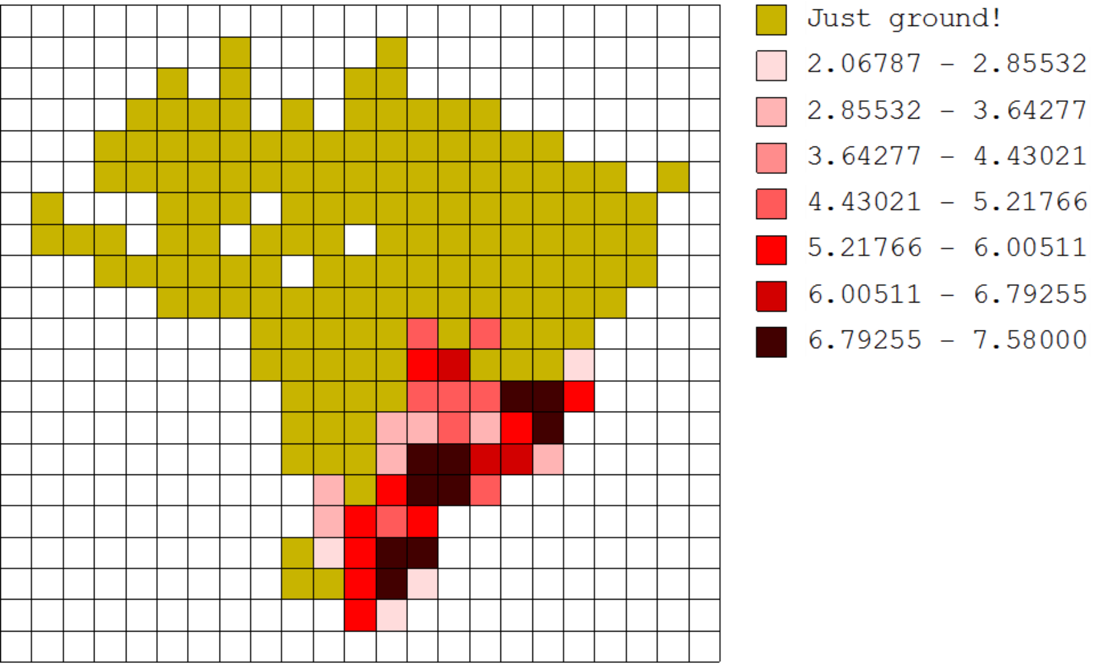

64 species give score:

Alpaida atomaria (0.000-0.805)
Alpaida tijuca (0.000-0.736)
Amazonepeira herrera (0.000-0.900)
Anapis castilla (0.000-0.700)
Anyphaenoides pacifica (0.000-0.700)
Attacobius attarum (0.000-0.733)
Centroctenus acara (0.000-0.700)
Centroctenus miriuma (0.000-0.613)
Chrysometa ludibunda (0.000-0.875)
Corinna capito (0.000-0.860)
Corinna loricata (0.000-0.833)
Corinna recurva (0.000-0.700)
Ctenus manauara (0.000-0.700)
Cyclosa vieirae (0.000-0.613)
Dolichognatha ducke (0.000-0.700)
Dyrines ducke (0.000-0.700)
Eilica modesta (0.000-0.696)
Encyosaccus sexmaculatus (0.000-0.700)
Enoploctenus cyclothorax (0.000-0.767)
Ephebopus uatuman (0.000-0.700)
Epicratinus amazonicus (0.000-0.800)
Eutichurus ravidus (0.000-0.818)
Gelanor heraldicus (0.000-0.700)
Hingstepeira dimona (0.000-0.800)
Hingstepeira folisecens (0.000-0.800)
Hogna sternalis (0.000-0.733)
Hypaeus miles (0.000-0.700)
Hypaeus triplagiatus (0.000-0.700)
Hypognatha colosso (0.000-0.700)
Litoporus dimona (0.000-0.800)
Lycosa thorelli (0.000-0.711)
Lygromma gasnieri (0.000-0.800)
Lygromma huberti (0.000-0.700)
Lyssomanes miniaceus (0.000-0.800)
Mangora aripeba (0.000-0.828)
Mangora mamiraua (0.000-0.700)
Mangora sumauma (0.000-0.900)
Metazygia uma (0.000-0.700)
Micrathena coca (0.000-0.787)
Micrathena embira (0.000-0.800)
Micrathena swainsoni (0.000-0.416)
Myrmecotypus olympus (0.000-0.700)
Ochyrocera hamadryas (0.000-0.700)
Pachomius sextus (0.000-0.700)
Parachemmis manauara (0.000-0.700)
Peucetia macroglossa (0.000-0.675)
Rhoicinus urucu (0.000-0.700)
Runcinioides litteratus (0.000-0.553)
Scytodes balbina (0.000-0.700)
Scytodes martiusi (0.000-0.700)
Scytodes univittata (0.000-0.436)
Selenops lavillai (0.000-0.700)
Stethorrhagus lupulus (0.000-0.700)
Strophius levyi (0.000-0.700)
Synotaxus waiwai (0.000-0.700)
Tariona gounellei (0.000-0.700)
Tobias caudatus (0.000-0.700)
Tupigea nadleri (0.000-0.875)
Wagneriana gavensis (0.000-0.900)
Zimiromus kleini (0.000-0.700)
Zimiromus syenus (0.000-0.700)
Breda nanica (0.000-0.700)
Micrathena abrahami (0.000-0.700)
Neoxyphinus petrogoblin (0.000-0.800)

Consensus area 2 of 106 (from 6 areas; max. values)

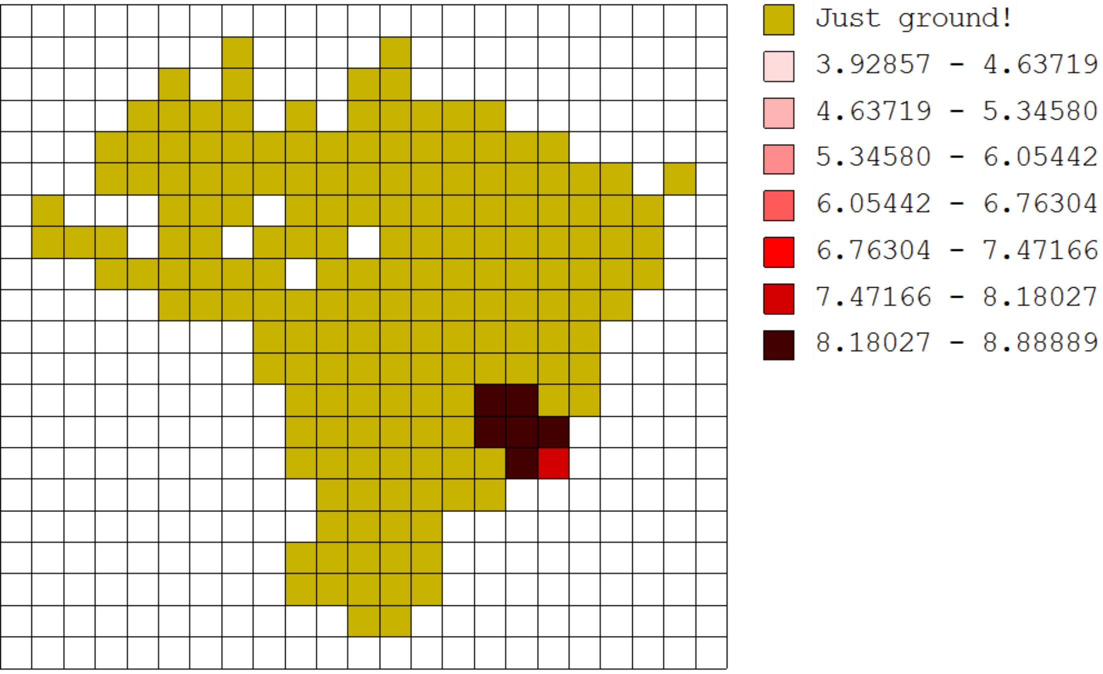

60 species give score:

Amazonepeira herrera (0.000-0.900)
Anapis castilla (0.000-0.700)
Anyphaenoides pacifica (0.000-0.700)
Araneus fronki (0.000-0.800)
Centroctenus acara (0.000-0.700)
Centroctenus miriuma (0.000-0.613)
Corinna plumipes (0.000-0.750)
Corinna recurva (0.000-0.700)
Cryptachaea pallipera (0.000-0.700)
Ctenus manauara (0.000-0.700)
Cyclosa vieirae (0.000-0.613)
Cyrtauchenius maculatus (0.000-0.635)
Diplura uniformis (0.000-0.583)
Dolichognatha ducke (0.000-0.700)
Dolomedes albicoxus (0.000-0.750)
Dubiaranea turbidula (0.000-0.622)
Dyrines ducke (0.000-0.700)
Encyosaccus sexmaculatus (0.000-0.700)
Ephebopus uatuman (0.000-0.700)
Epicratinus amazonicus (0.000-0.800)
Eurymorion insigne (0.000-0.700)
Gelanor heraldicus (0.000-0.700)
Hingstepeira dimona (0.000-0.800)
Hingstepeira folisecens (0.000-0.800)
Hogna sternalis (0.000-0.519)
Hypaeus miles (0.000-0.700)
Hypaeus triplagiatus (0.000-0.700)
Hypognatha colosso (0.000-0.700)
Litoporus dimona (0.000-0.800)
Lygromma gasnieri (0.000-0.800)
Lygromma huberti (0.000-0.700)
Mangora mamiraua (0.000-0.700)
Mangora sumauma (0.000-0.900)
Metazygia uma (0.000-0.700)
Micrathena coca (0.000-0.787)
Micrathena embira (0.000-0.800)
Myrmecotypus olympus (0.000-0.700)
Ochyrocera hamadryas (0.000-0.700)
Pachomius sextus (0.000-0.700)
Parachemmis manauara (0.000-0.700)
Peucetia macroglossa (0.000-0.675)
Phiale radians (0.000-0.750)
Rhoicinus urucu (0.000-0.700)
Scytodes balbina (0.000-0.700)
Scytodes caratinga (0.000-0.700)
Scytodes domhelvecio (0.000-0.700)
Scytodes mangabeiras (0.000-0.750)
Scytodes martiusi (0.000-0.700)
Scytodes tyaiapyssanga (0.000-0.800)
Selenops lavillai (0.000-0.700)
Stethorrhagus lupulus (0.000-0.700)
Synotaxus waiwai (0.000-0.700)
Teudis lenis (0.000-0.700)
Zimiromus kleini (0.000-0.700)
Zimiromus syenus (0.000-0.700)
Brasilomma enigmatica (0.000-0.800)
Breda nanica (0.000-0.700)
Micrathena abrahami (0.000-0.700)
Neoxyphinus petrogoblin (0.000-0.800)
Umuara adfabilis (0.000-0.800)

Consensus area 3 of 106 (from 6 areas; max. values)

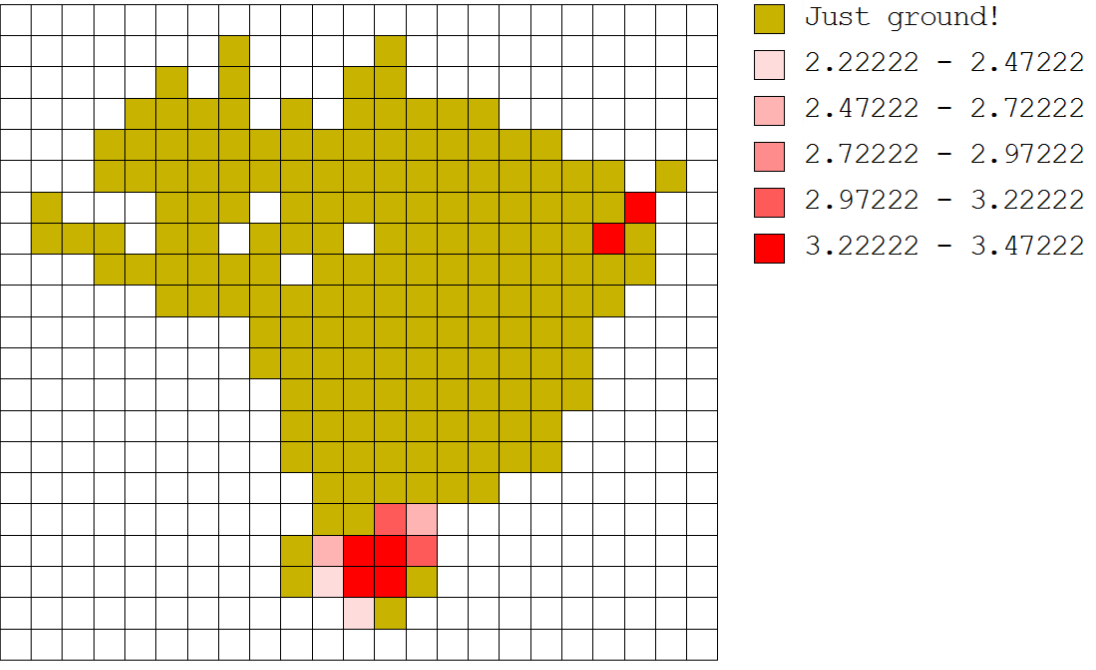

49 species give score:

Amazonepeira herrera(0.000-0.900)
Anyphaenoides pacifica(0.000-0.700)
Centroctenus acara(0.000-0.700)
Corinna recurva(0.000-0.700)
Cyclosa vieirae(0.000-0.613)
Dyrines ducke(0.000-0.700)
Ephebopus uatuman(0.000-0.700)
Gelanor heraldicus(0.000-0.700)
Hingstepeira folisecens(0.000-0.800)
Hypaeus triplagiatus(0.000-0.700)
Larinia bivittata(0.000-0.656)
Lygromma gasnieri(0.000-0.800)
Mangora mamiraua(0.000-0.700)
Metazygia saturnino(0.000-0.750)
Micrathena coca(0.000-0.787)
Mimetus melanoleucus(0.000-0.746)
Ochyrocera hamadryas(0.000-0.700)
Parachemmis manauara(0.000-0.700)
Rhoicinus urucu(0.000-0.700)
Scytodes martiusi(0.000-0.700)
Stethorrhagus lupulus(0.000-0.700)
Synotaxus waiwai(0.000-0.700)
Zimiromus syenus(0.000-0.700)
Eustala belissima(0.000-0.750)
Neoxyphinus petrogoblin(0.000-0.800)
Anapis castilla(0.000-0.700)
Araneus lathyrinus(0.000-0.938)
Centroctenus miriuma(0.000-0.613)
Ctenus manauara(0.000-0.700)
Dolichognatha ducke(0.000-0.700)
Encyosaccus sexmaculatus(0.000-0.700)
Epicratinus amazonicus(0.000-0.800)
Hingstepeira dimona(0.000-0.800)
Hypaeus miles(0.000-0.700)
Hypognatha colosso(0.000-0.700)
Litoporus dimona(0.000-0.800)
Lygromma huberti(0.000-0.700)
Mangora sumauma(0.000-0.900)
Metazygia uma(0.000-0.700)
Micrathena embira(0.000-0.800)
Myrmecotypus olympus(0.000-0.700)
Pachomius sextus(0.000-0.700)
Peucetia macroglossa(0.000-0.675)
Scytodes balbina(0.000-0.700)
Selenops lavillai(0.000-0.700)
Synemosyna taperae(0.000-0.667)
Zimiromus kleini(0.000-0.700)
Breda nanica(0.000-0.700)
Micrathena_abrahami(0.000-0.700)

Consensus area 4 of 106 (from 2 areas; max. values)

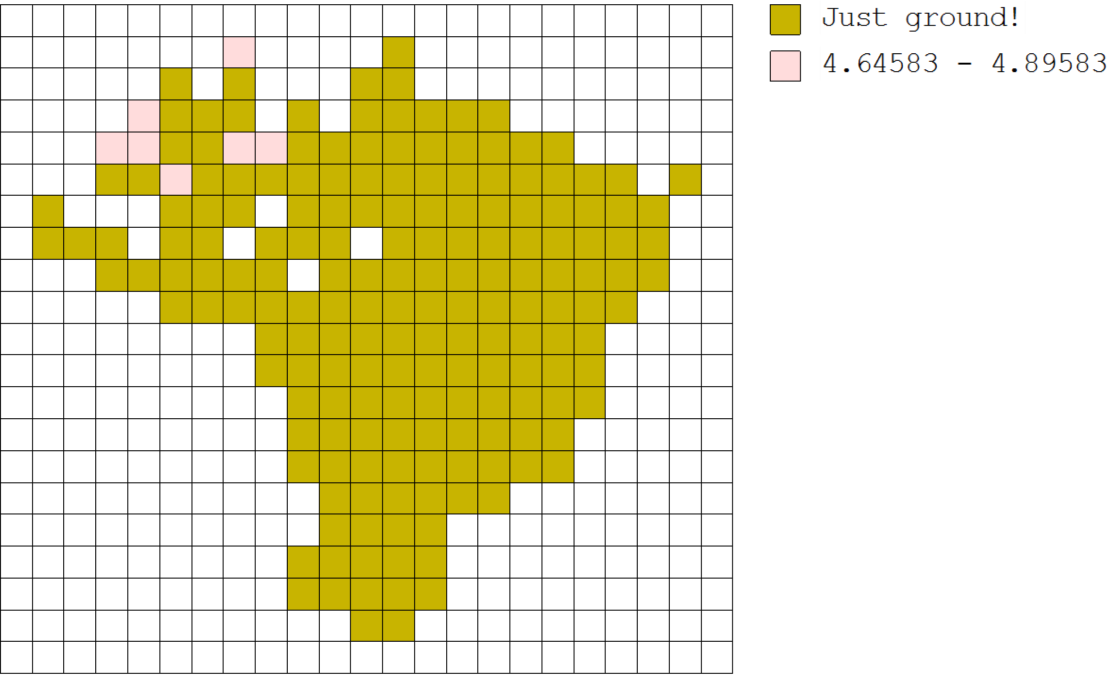

7 species give score:

Echinotheridion utibile (0.000-0.800)
Olios pictitarsis (0.729-0.800)
Otiothops oblongus (0.750-0.800)
Titidius quinquenotatus (0.000-0.750)
Mangora uraricoera (0.800-0.917)
Otiothops hoeferi (0.750-0.800)
Phoroncidia_moyobamba (0.750-0.800)

Consensus area 5 of 106 (from 1 areas; max. values)

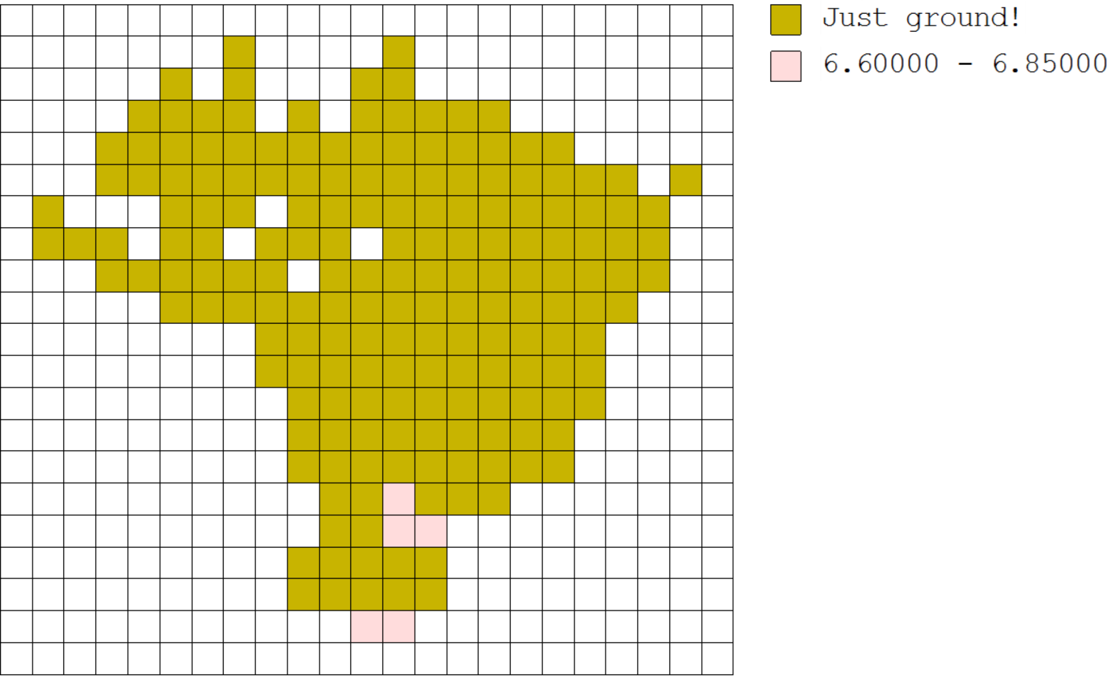

10 species give score:

Grammostola actaeon (0.800)
Metazygia mundulella (0.700)
Otiothops inflatus (0.700)
Radulphius bidentatus (0.700)
Grammostola anthracina (0.700)
Hogna auricoma (0.600)
Notiohyphantes excelsus (0.600)
Pirata velox (0.700)
Theridiosoma chiripa (0.400)
Hilaira frigida (0.700)

Consensus area 6 of 106 (from 16 areas; max. values)

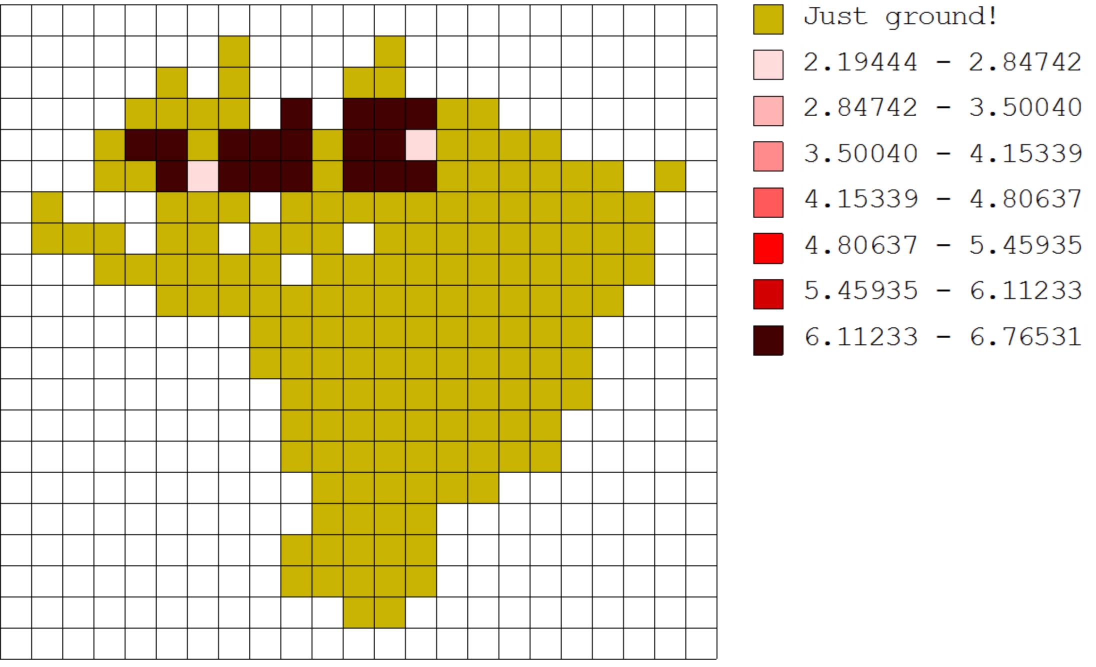

14 species give score:

Abapeba_taruma (0.000-0.698)
Centroctenus_auberti (0.000-0.813)
Ctenus_minor (0.000-0.650)
Mago_acutidens (0.000-0.694)
Micrepeira_fowleri (0.000-0.772)
Tupirinna_rosae (0.000-0.750)
Neoxyphinus barreirosi (0.000-0.696)
Carapoia_fowleri (0.000-0.737)
Corinna_ducke (0.000-0.750)
Lyssomanes_longipes (0.000-0.700)
Metagonia_taruma (0.000-0.754)
Thwaitesia_simoni (0.000-0.833)
Wagneriana_maseta (0.000-0.750)
Soesiladeepakius gasnieri (0.000-0.750)

Consensus area 7 of 106 (from 14 areas; max. values)

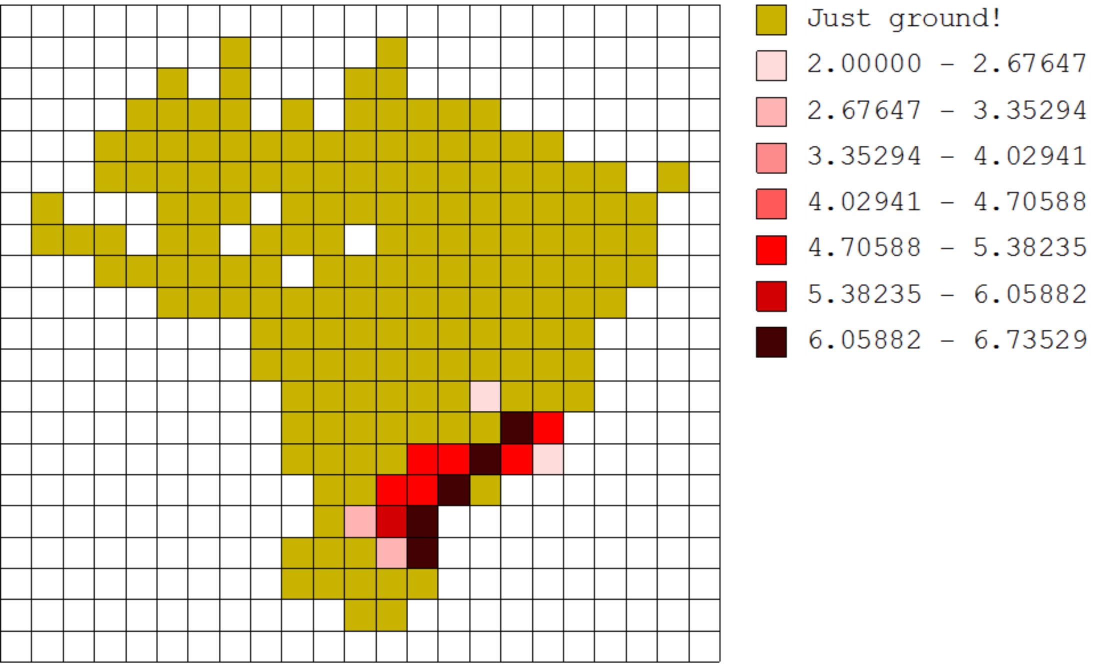

64 species give score:

Acacesia yacuiensis (0.000-0.563)
Anapis castilla (0.000-0.700)
Arnoliseus calcarifer (0.000-0.570)
Buckupiella imperatriz (0.000-0.889)
Centroctenus miriuma (0.000-0.613)
Corinna recurva (0.000-0.700)
Cyclosa vieirae (0.000-0.613)
Dolichognatha ducke (0.000-0.700)
Encyosaccus sexmaculatus (0.000-0.700)
Epicratinus amazonicus (0.000-0.800)
Hingstepeira dimona (0.000-0.800)
Hypaeus miles (0.000-0.700)
Hypognatha colosso (0.000-0.700)
Lygromma gasnieri (0.000-0.800)
Lyssomanes tristis (0.000-0.371)
Mangora mamiraua (0.000-0.700)
Mastophora ypiranga (0.000-0.833)
Metazygia uma (0.000-0.700)
Micrathena embira (0.000-0.800)
Myrmecotypus olympus (0.000-0.700)
Olios caprinus (0.000-0.833)
Parachemmis manauara (0.000-0.700)
Phoneutria keyserlingi (0.000-0.658)
Saitis cyanipes (0.000-0.722)
Scytodes martiusi (0.000-0.700)
Sphecozone labiata (0.000-1.000)
Synotaxus waiwai (0.000-0.700)
Wagneriana dimastophora (0.000-0.646)
Xiruana affinis (0.000-0.900)
Zimiromus syenus (0.000-0.700)
Exocora girotii (0.000-0.778)
Micrathena abrahami (0.000-0.700)
Amazonepeira herrera (0.000-0.900)
Anyphaenoides pacifica (0.000-0.700)
Aysha guarapuava (0.000-0.684)
Centroctenus acara (0.000-0.700)
Chrysso compressa (0.000-0.570)
Ctenus manauara (0.000-0.700)
Dipoena granulata (0.000-0.583)
Dyrines ducke (0.000-0.700)
Ephebopus uatuman (0.000-0.700)
Gelanor heraldicus (0.000-0.700)
Hingstepeira folisecens (0.000-0.800)
Hypaeus triplagiatus (0.000-0.700)
Litoporus dimona (0.000-0.800)
Lygromma huberti (0.000-0.700)
Mangora aripeba (0.000-0.612)
Mangora sumauma (0.000-0.900)
Metagonia bifida (0.000-1.000)
Micrathena coca (0.000-0.787)
Micrathena reali (0.000-1.000)
Ochyrocera hamadryas (0.000-0.700)
Pachomius sextus (0.000-0.700)
Peucetia macroglossa (0.000-0.675)
Rhoicinus urucu (0.000-0.700)
Scytodes balbina (0.000-0.700)
Selenops lavillai (0.000-0.700)
Stethorrhagus lupulus (0.000-0.700)
Tutaibo velox (0.000-0.632)
Wagneriana heteracantha (0.000-0.295)
Zimiromus kleini (0.000-0.700)
Breda nanica (0.000-0.700)
Exocora ribeiroi (0.000-1.000)
Neoxyphinus petrogoblin (0.000-0.800)

Consensus area 8 of 106 (from 3 areas; max. values)

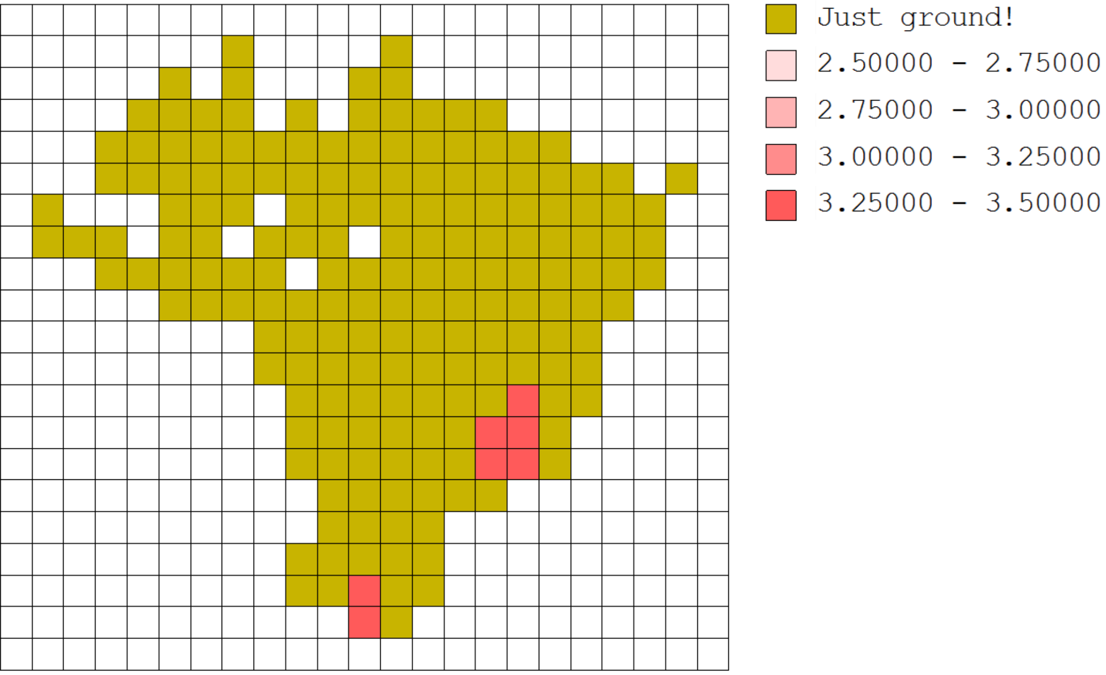

5 species give score:

Anelosimus nigrescens(0.000-0.619)
Nesticus taim(0.643-0.833)
Gayenna fuscotaeniata(0.643-0.833)
Loxosceles rufipes(0.643-0.833)
Patrera_virgata(0.000-0.714)

Consensus area 9 of 106 (from 1 areas; max. values)

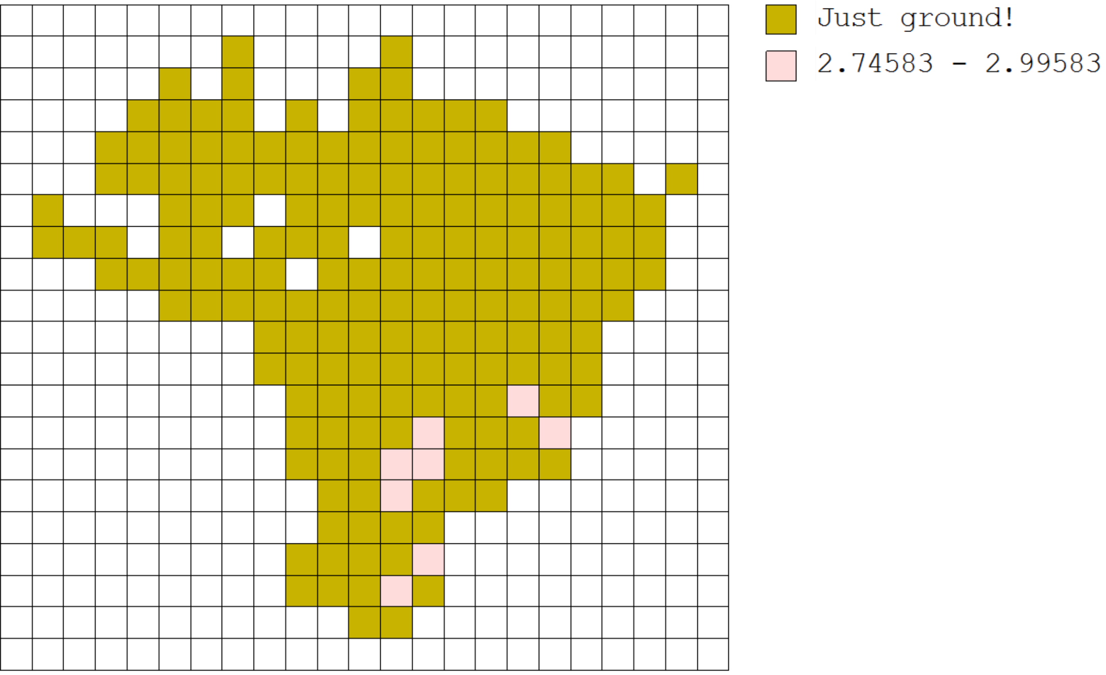

5 species give score:

Chrysso compressa(0.550)
Theridion calcynatum(0.300)
Zimiromus montenegro(0.550)
Macrophyes jundiai(0.750)
Wagneriana_dimastophora(0.596)

Consensus area 10 of 106 (from 1 areas; max. values)

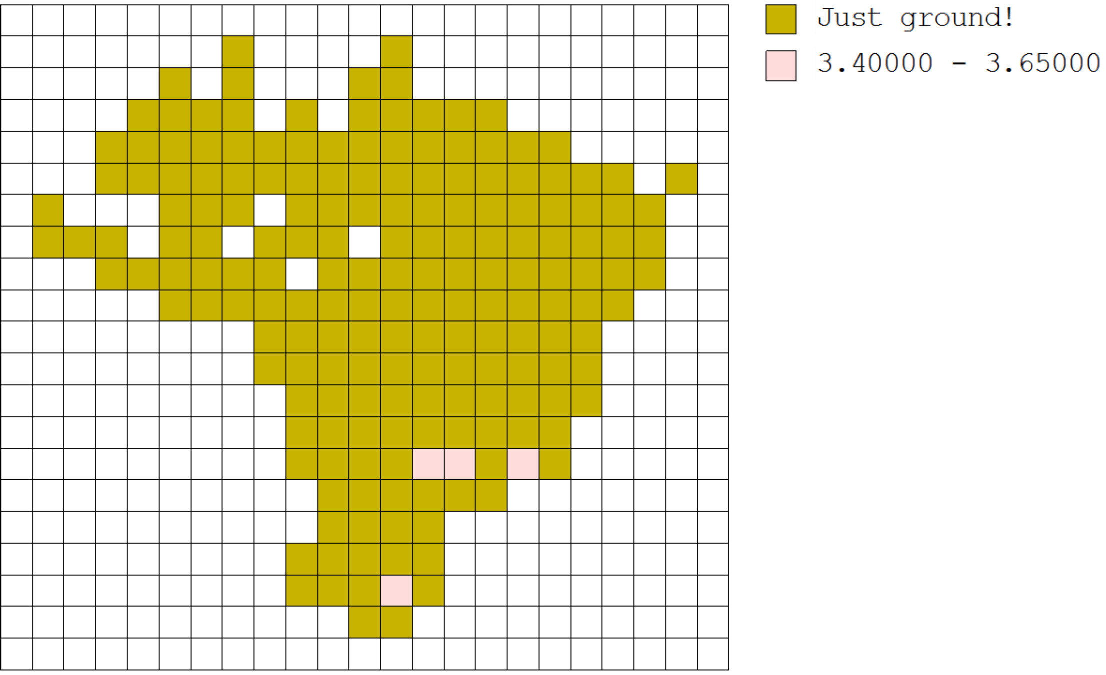

5 species give score:

Chrysometa cambara(0.700)
Patrera longipes(0.400)
Theridion opolon(0.700)
Frigga quintensis(1.000)
Patrera_procera(0.600)

Consensus area 11 of 106 (from 5 areas; max. values)

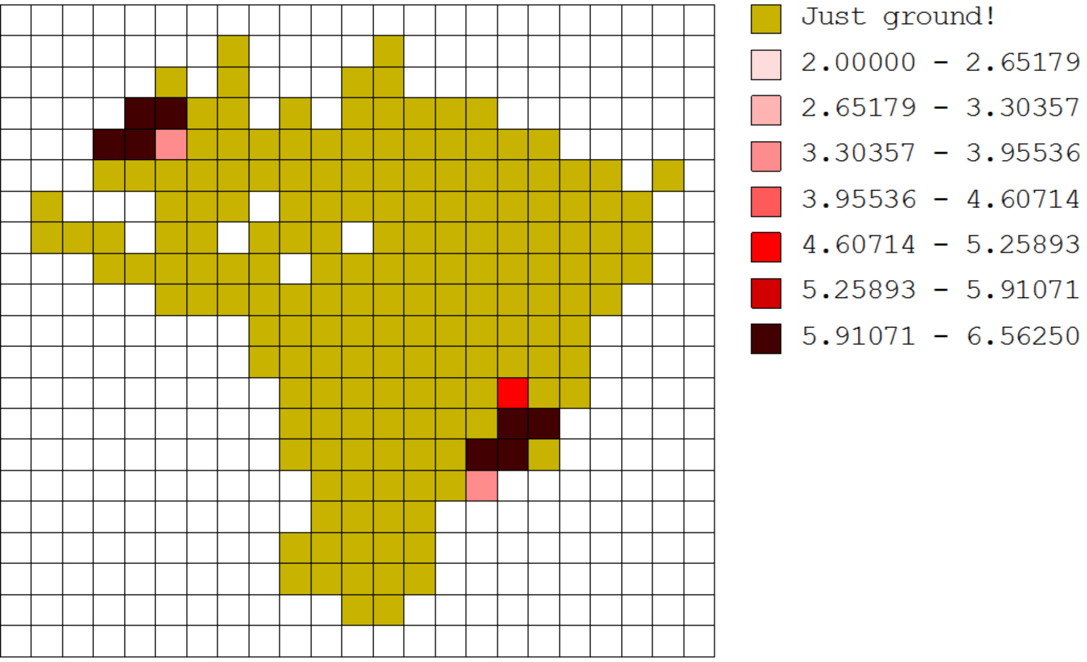

56 species give score:

Amazonepeira herrera (0.000-0.900)
Anyphaenoides pacifica (0.000-0.700)
Centroctenus miriuma (0.000-0.613)
Corinna recurva (0.000-0.700)
Cyclosa vieirae (0.000-0.613)
Dolichognatha ducke (0.000-0.700)
Encyosaccus sexmaculatus (0.000-0.700)
Epicratinus amazonicus (0.000-0.800)
Hingstepeira dimona (0.000-0.800)
Hypaeus miles (0.000-0.700)
Hypognatha colosso (0.000-0.700)
Lygromma gasnieri (0.000-0.800)
Mangora mamiraua (0.000-0.700)
Mesabolivar difficilis (0.000-0.750)
Micrathena coca (0.000-0.787)
Myrmecotypus olympus (0.000-0.700)
Ocrepeira klossi (0.000-0.833)
Parachemmis manauara (0.000-0.700)
Phoroncidia biocellata (0.000-1.000)
Radulphius laticeps (0.000-0.349)
Runcinioides pustulatus (0.000-0.667)
Scytodes martiusi (0.000-0.700)
Sphecotypus niger (0.000-0.667)
Strophius mendax (0.000-0.667)
Titidius multifasciatus (0.000-0.722)
Zimiromus kleini (0.000-0.700)
Breda nanica (0.000-0.700)
Neoxyphinus petrogoblin (0.000-0.800)
Anapis castilla (0.000-0.700)
Centroctenus acara (0.000-0.700)
Coleosoma acutiventer (0.000-0.500)
Ctenus manauara (0.000-0.700)
Dipoena keyserlingi (0.000-0.833)
Dyrines ducke (0.000-0.700)
Ephebopus uatuman (0.000-0.700)
Gelanor heraldicus (0.000-0.700)
Hingstepeira folisecens (0.000-0.800)
Hypaeus triplagiatus (0.000-0.700)
Litoporus dimona (0.000-0.800)
Lygromma huberti (0.000-0.700)
Mangora sumauma (0.000-0.900)
Metazygia uma (0.000-0.700)
Micrathena embira (0.000-0.800)
Ochyrocera hamadryas (0.000-0.700)
Pachomius sextus (0.000-0.700)
Peucetia macroglossa (0.000-0.675)
Proshapalopus anomalus (0.000-0.729)
Rhoicinus urucu (0.000-0.700)
Scytodes balbina (0.000-0.700)
Selenops lavillai (0.000-0.700)
Stethorrhagus lupulus (0.000-0.700)
Synotaxus waiwai (0.000-0.700)
Tmarus camellinus (0.000-1.000)
Zimiromus syenus (0.000-0.700)
Micrathena abrahami (0.000-0.700)
Vitalius nondescriptus (0.000-0.833)

Consensus area 12 of 106 (from 6 areas; max. values)

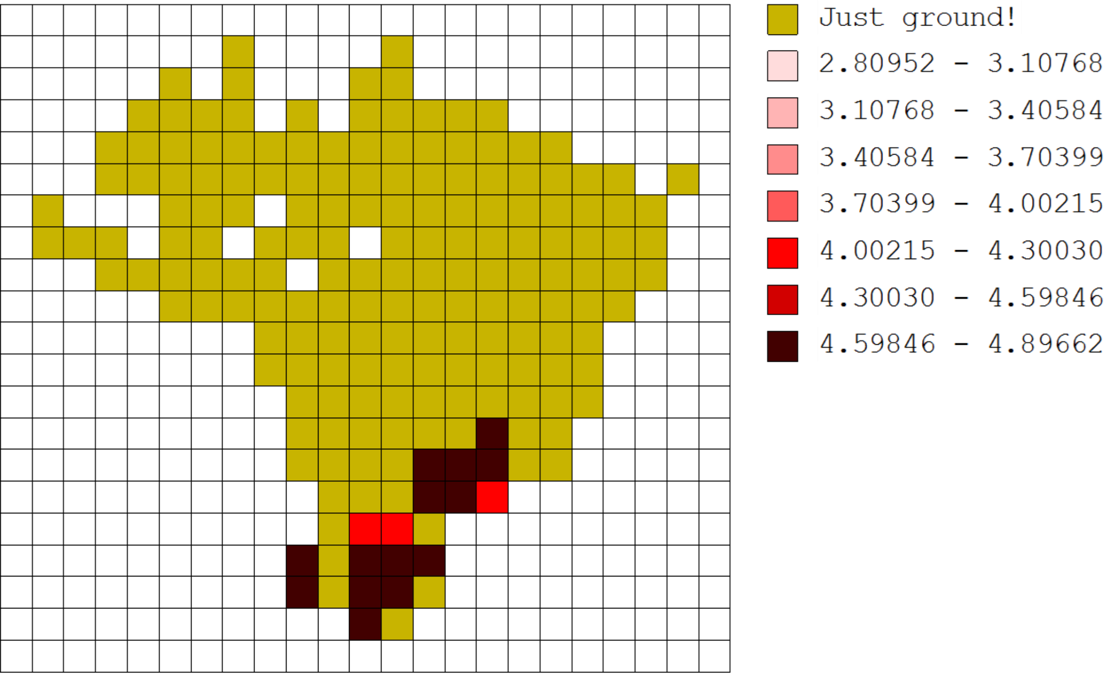

9 species give score:

Alpaida gallardoi (0.629-0.889)
Chrosiothes niteroi (0.000-0.813)
Nesticus brignolii (0.719-1.000)
Sanogasta maculatipes (0.000-0.808)
Vitalius roseus (0.000-0.559)
Camillina major (0.000-0.682)
Meriola cetiformis (0.000-0.393)
Polybetes pythagoricus (0.286-0.781)
Selenops_rapax (0.000-0.656)

Consensus area 13 of 106 (from 6 areas; max. values)

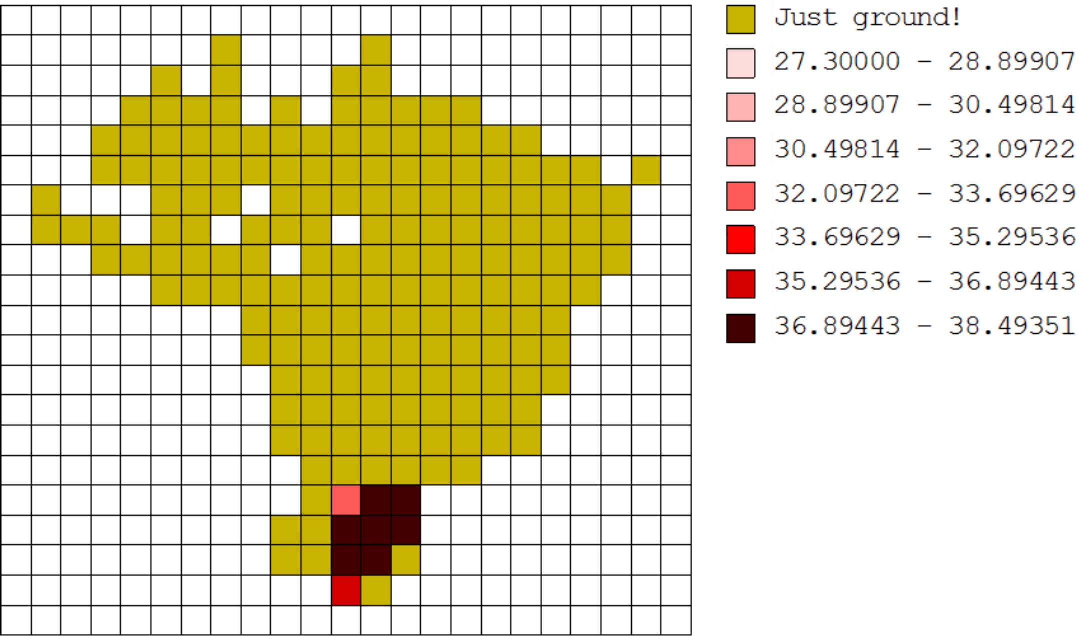

82 species give score:

Acanthogonatus ericae (0.643-0.833)
Acanthogonatus tacuariensis (0.643-0.833)
Acentroscelus ramboi (0.600-1.000)
Achaearanea digitus (0.000-0.800)
Aglaoctenus oblongus (0.584-0.900)
Alpaida ericae (0.714-1.000)
Alpaida lomba (0.622-0.900)
Alpaida rostratula (0.600-1.000)
Alpaida sobradinho (0.667-0.875)
Araneus blumenau (0.000-0.643)
Arctosa sapiranga (0.643-0.833)
Aysha bonaldoi (0.643-0.833)
Aysha chicama (0.643-0.833)
Aysha vacaria (0.643-0.833)
Bryantella smaragdus (0.000-0.667)
Caponina alegre (0.526-0.667)
Cryptachaea isana (0.000-0.583)
Cybaeodamus taim (0.000-0.700)
Cyclosa turbinata (0.000-0.800)
Diplura catharinensis (0.000-0.643)
Dipoena foliata (0.000-0.583)
Dipoena plaumanni (0.000-0.700)
Epicadus pallidus (0.000-0.750)
Euophrys saitiformis (0.500-0.900)
Eustala photographica (0.000-0.325)
Fageia amabilis (0.000-0.833)
Faiditus plaumanni (0.000-0.583)
Gamasomorpha m-scripta (0.000-0.544)
Gelanor altithorax (0.444-0.857)
Gelanor mixtus (0.643-0.833)
Grammostola iheringi (0.000-0.643)
Guaraniella mahnerti (0.000-0.667)
Hamataliwa banksi (0.000-0.800)
Hogna auricoma (0.000-0.544)
Homoeomma villosum (0.000-0.700)
Isoctenus minusculus (0.500-0.900)
Kaira erwini (0.643-0.833)
Kochiura decolorata (0.000-0.643)
Latonigena auricomus (0.000-0.800)
Linothele annulifila (0.000-0.643)
Lycosa paranensis (0.000-0.584)
Lycosa pictipes (0.000-0.700)
Mangora fundo (0.400-0.600)
Mangora velha (0.000-0.643)
Misumenoides variegatus (0.714-1.000)
Moyosi prativaga (0.333-0.750)
Neomaso damocles (0.000-0.750)
Neoxyphinus ogloblini (0.389-0.667)
Nesticus brasiliensis (0.000-0.325)
Nops meridionalis (0.000-0.526)
Notiohyphantes excelsus (0.000-0.544)
Petrichus meridionalis (0.000-0.700)
Polybetes quadrifoveatus (0.000-0.700)
Scolecura propinqua (0.643-0.833)
Scytodes chapeco (0.000-0.533)
Scytodes maquine (0.000-0.357)
Scytodes tabuleiro (0.000-0.643)
Smermisia parvoris (0.000-0.700)
Synaemops notabilis (0.000-0.526)
Synema bipunctatum (0.000-0.622)
Synema haemorrhoidale (0.000-0.700)
Tasata taim (0.000-0.700)
Tekellina guaiba (0.643-0.833)
Theridiosoma chiripa (0.444-0.700)
Thymoites puer (0.389-0.667)
Tmarus variatus (0.000-0.875)
Trachelopachys gracilis (0.000-0.583)
Trachyzelotes lyonneti (0.000-0.700)
Tulpius gauchus (0.643-0.833)
Tupigea lisei (0.000-0.583)
Tupigea paula (0.643-0.833)
Tutaibo rusticellus (0.622-0.800)
Verrucosa undecimvariolata (0.600-0.800)
Xenonemesia araucaria (0.714-1.000)
Xiombarg plaumanni (0.000-0.800)
Alpaida ocotolobata (0.000-0.700)
Anelosimus decoloratus (0.000-0.455)
Eustala crista (0.000-0.357)
Gayenna maculatipes (0.000-0.700)
Latonigena lami (0.000-0.583)
Pterinopelma vitiosum (0.667-0.875)
Stenoteromata palmar (0.000-0.584)

Consensus area 14 of 106 (from 1 areas; max. values)

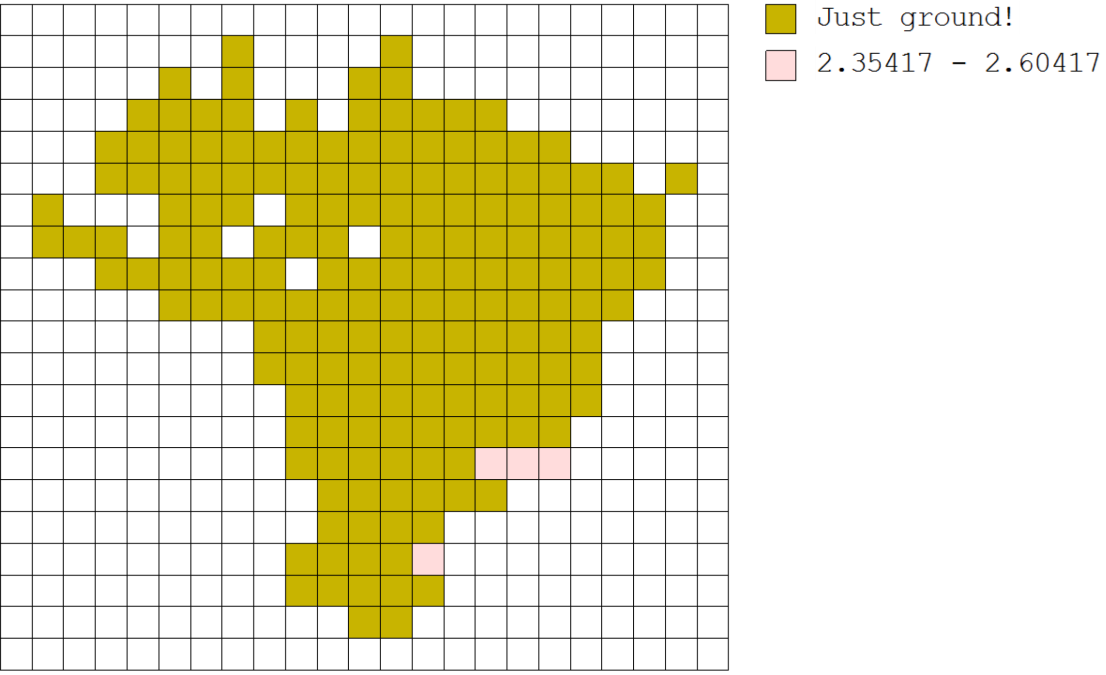

3 species give score:
Ameridion unanimum(0.875)
Fernandezina_tijuca(0.750)
Olios caprinus(0.729)

Consensus area 15 of 106 (from 5 areas; max. values)

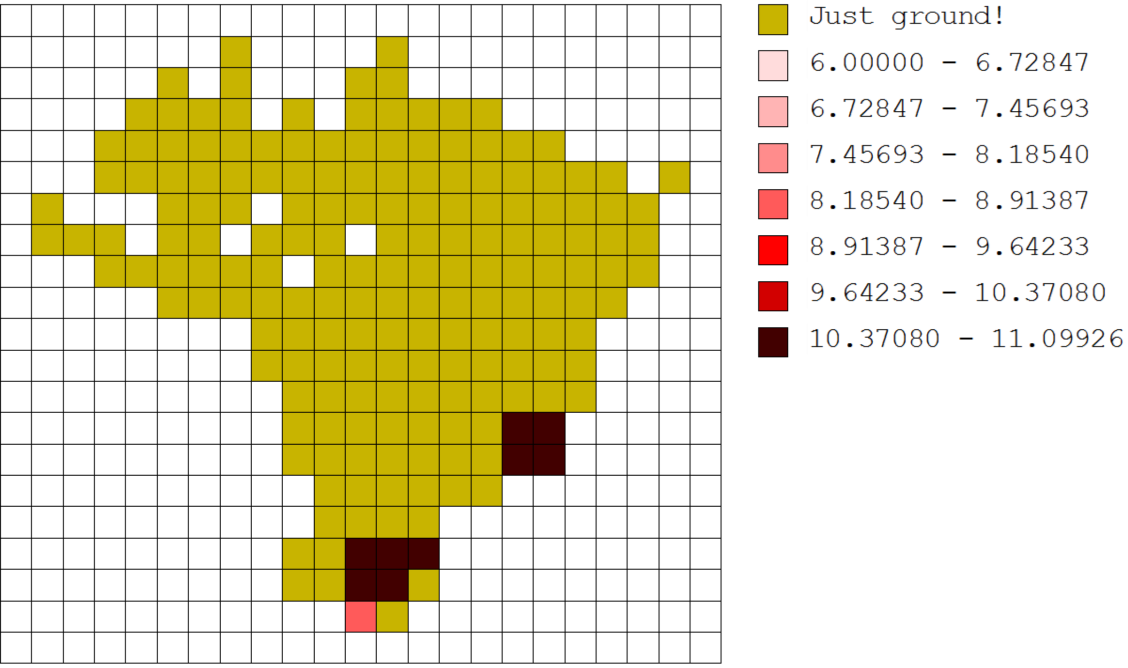

22 species give score:
Ameridion_unanimum(0.000-0.714)
Chrysso_nigrosterna(0.625-0.643)
Cryptachaea_bellula(0.000-0.635)
Enoploctenus_maculipes(0.000-0.714)
Ero_catharinae(0.000-0.500)
Fernandezina_tijuca(0.000-0.643)
Isoctenus_malabaris(0.000-0.556)
Leucauge_volupis(0.547-0.714)
Metazygia_floresta(0.625-0.643)
Metepeira_vigilax(0.551-0.714)
Micrathena_rubicundula(0.557-0.714)
Nesticus_taim(0.000-0.643)
Ocrepeira_lisei(0.000-0.556)
Onocolus_infelix(0.000-0.571)
Patrera_virgata(0.000-0.714)
Phoroncidia_tricuspidata(0.625-0.643)
Smermisia_vicosana(0.000-0.643)
Taczanowskia sextuberculata(0.000-0.643)
Tariona_bruneti(0.000-0.643)
Tobias_corticatus(0.000-0.643)
Gayenna fuscotaeniata(0.000-0.643)
Metazygia glomerabilis(0.000-0.643)

Consensus area 16 of 106 (from 12 areas; max. values)

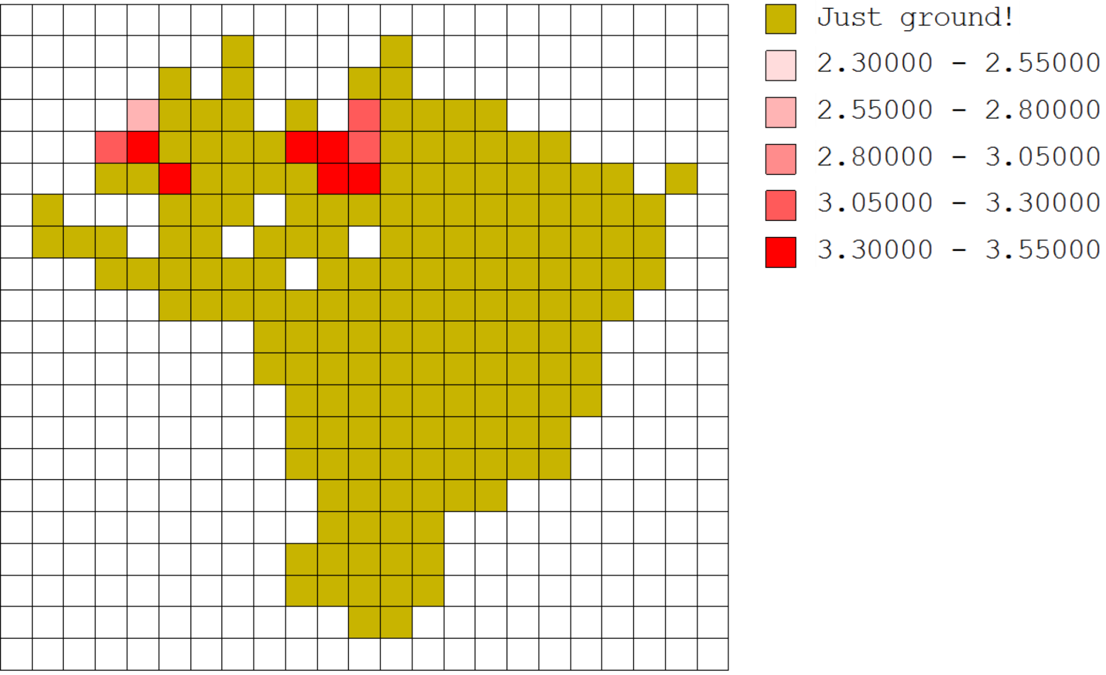

6 species give score:

Cyriocosmus elegans (0.667-0.833)
Metagonia taruma (0.000-0.900)
Neopisinus urucu (0.000-0.800)
Hypaeus frontosus (0.000-0.833)
Synotaxus siolii (0.618-0.800)
Neoxyphinus barreirosi (0.000-0.720)

Consensus area 17 of 106 (from 9 areas; max. values)

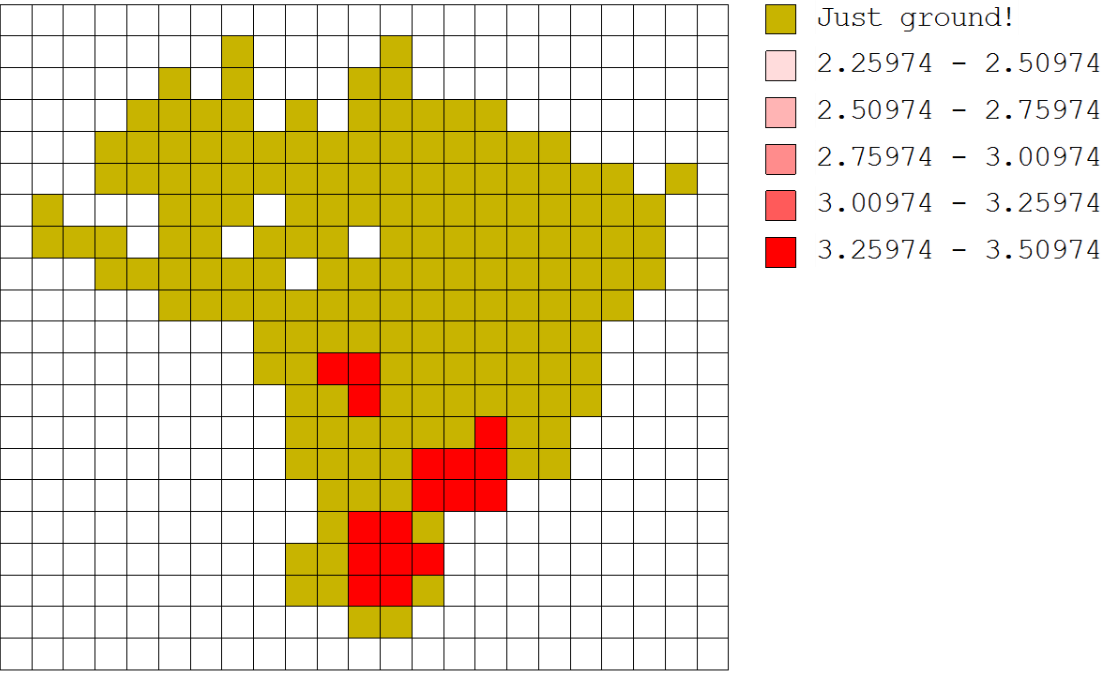

5 species give score:

Agelista andina (0.700-0.773)
Lycosa nordenskjoldi (0.667-0.727)
Aysha paiassaquera (0.000-0.733)
Alpaida lanei (0.000-0.731)
Theridion_orgea (0.577-0.767)

Consensus area 18 of 106 (from 6 areas; max. values)

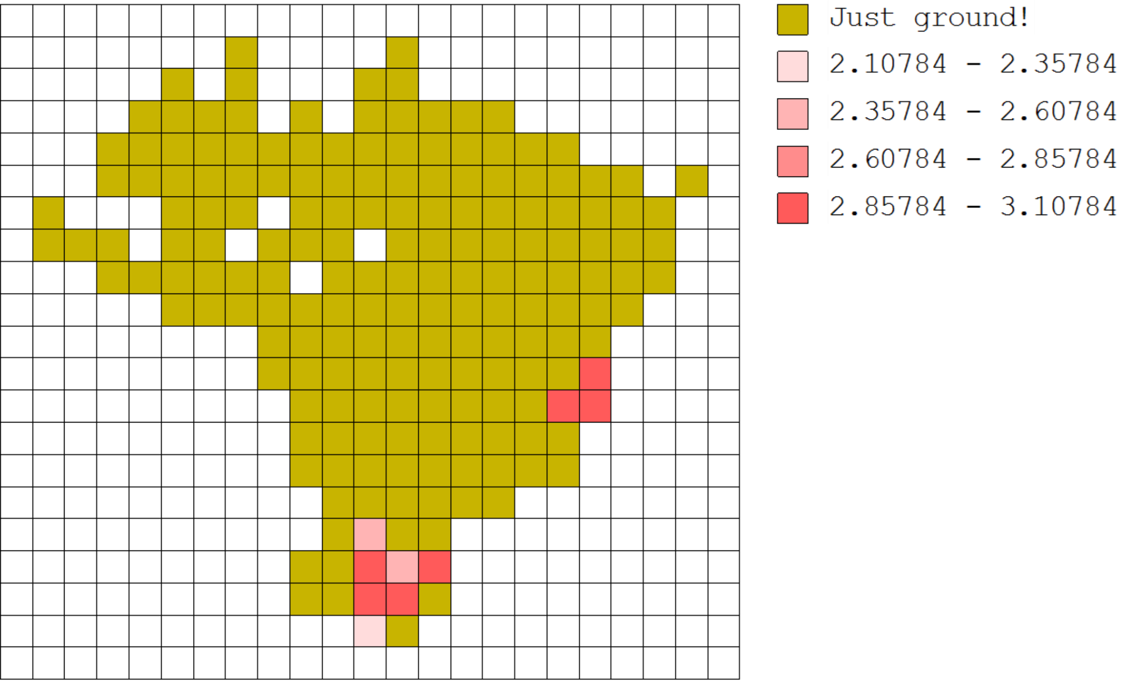

6 species give score:
Alpaida octolobata (0.539-0.733)
Caayguara_apiaba (0.000-0.508)
Isigonia camacan (0.551-0.735)
Lyssomanes camacanensis (0.000-0.833)
Metazygia genialis (0.000-0.786)
Onocolus infelix (0.000-0.555)

Consensus area 19 of 106 (from 3 areas; max. values)

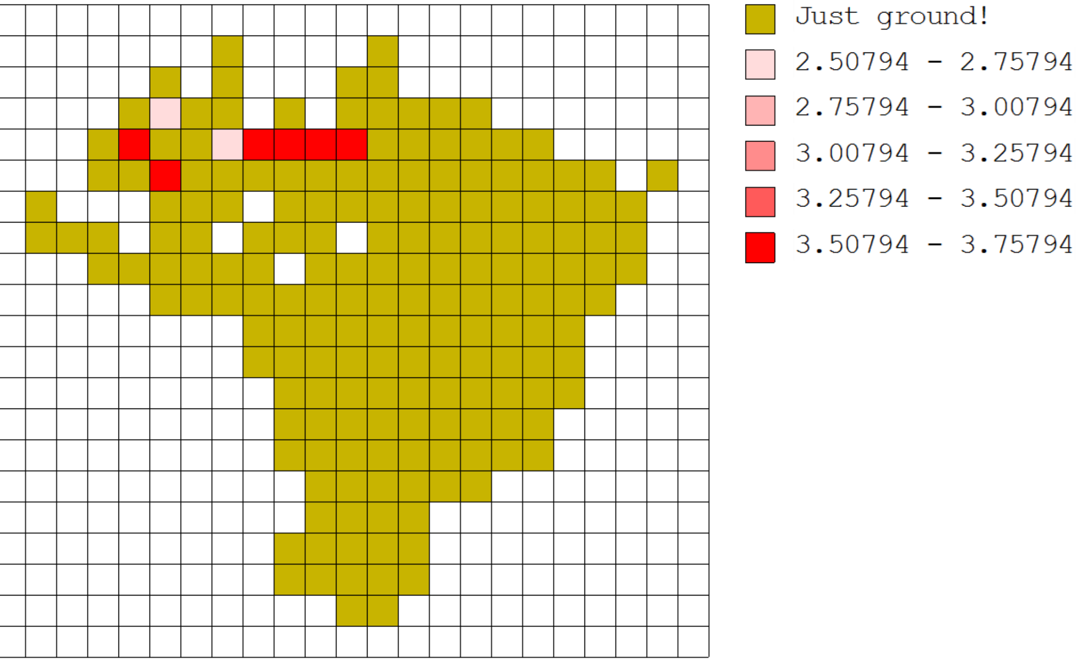

5 species give score:
Asthenoctenus_longistylus (0.682-0.900)
Carapoia_fowleri (0.000-0.614)
Metagonia_taruma (0.675-1.000)
Syntrechalea_adis (0.682-1.000)
Neoxyphinus barreirosi (0.000-0.682)

Consensus area 20 of 106 (from 5 areas; max. values)

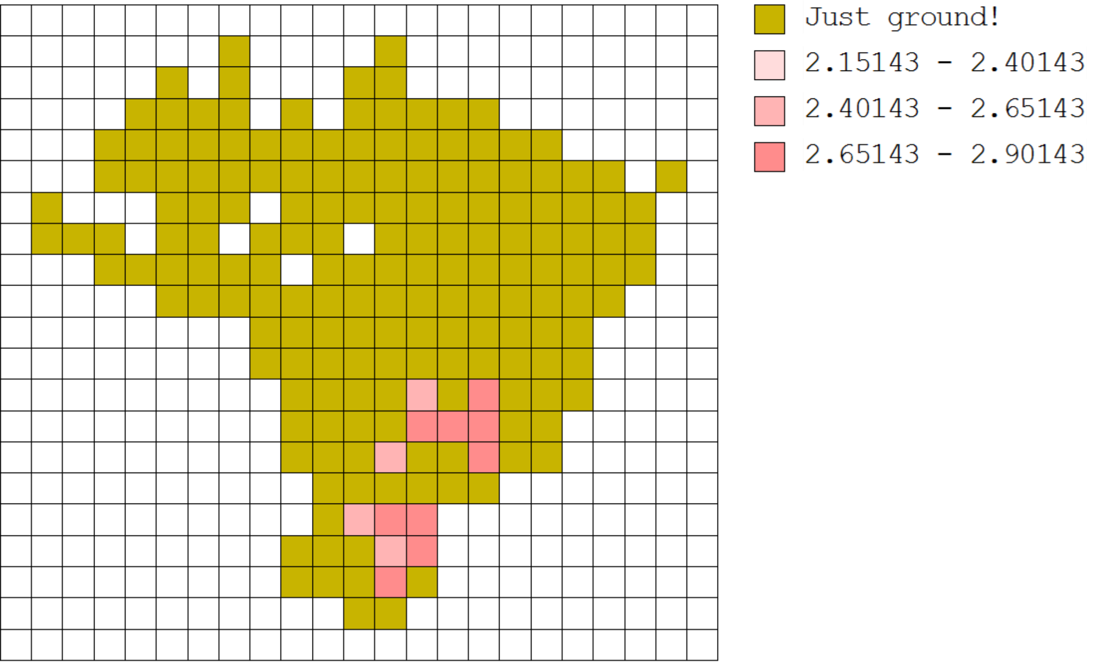

49 species give score:

Acacesia yacuiensis (0.000-0.467)
Amazonepeira herrera (0.000-0.900)
Anyphaenoides pacifica (0.000-0.700)
Centroctenus miriuma (0.000-0.613)
Corinna recurva (0.000-0.700)
Cyclosa vieirae (0.000-0.613)
Dyrines ducke (0.000-0.700)
Ephebopus uatuman (0.000-0.700)
Gelanor heraldicus (0.000-0.700)
Hingstepeira folisecens (0.000-0.800)
Hypaeus triplagiatus (0.000-0.700)
Kaira gibberosa (0.000-0.779)
Lygromma gasnieri (0.000-0.800)
Mangora mamiraua (0.000-0.700)
Metazygia uma (0.000-0.700)
Micrathena embira (0.000-0.800)
Myrmecotypus olympus (0.000-0.700)
Pachomius sextus (0.000-0.700)
Parafluda banksi (0.000-0.711)
Rhoicinus urucu (0.000-0.700)
Scytodes martiusi (0.000-0.700)
Stethorrhagus lupulus (0.000-0.700)
Zimiromus kleini (0.000-0.700)
Breda nanica (0.000-0.700)
Neoxyphinus petrogoblin (0.000-0.800)
Alpaida citrina (0.000-0.800)
Anapis castilla (0.000-0.700)
Centroctenus acara (0.000-0.700)
Corinna colombo (0.000-0.286)
Ctenus manauara (0.000-0.700)
Dolichognatha ducke (0.000-0.700)
Encyosaccus sexmaculatus (0.000-0.700)
Epicratinus amazonicus (0.000-0.800)
Hingstepeira dimona (0.000-0.800)
Hypaeus miles (0.000-0.700)
Hypognatha colosso (0.000-0.700)
Litoporus dimona (0.000-0.800)
Lygromma huberti (0.000-0.700)
Mangora sumauma (0.000-0.900)
Micrathena coca (0.000-0.787)
Misumenops bivittatus (0.000-0.622)
Ochyrocera hamadryas (0.000-0.700)
Parachemmis manauara (0.000-0.700)
Peucetia macroglossa (0.000-0.675)
Scytodes balbina (0.000-0.700)
Selenops lavillai (0.000-0.700)
Synotaxus waiwai (0.000-0.700)
Zimiromus syenus (0.000-0.700)
Micrathena_abrahami (0.000-0.700)

Consensus area 21 of 106 (from 5 areas; max. values)

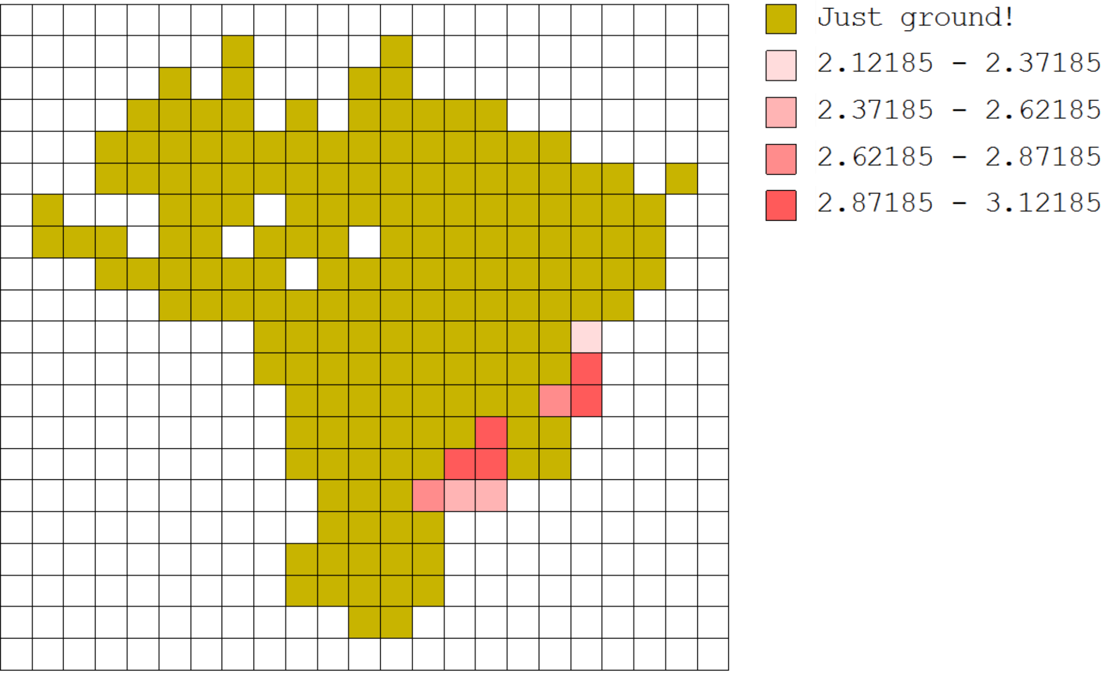

6 species give score:

Allocosa brasiliensis (0.000-0.700)
Mastophora piras (0.000-0.622)
Ocrepeira jacara (0.643-0.700)
Avicularia sooretama (0.000-0.786)
Metazygia bahia (0.438-0.917)
Vinnius subfasciatus (0.000-0.600)

Consensus area 22 of 106 (from 4 areas; max. values)

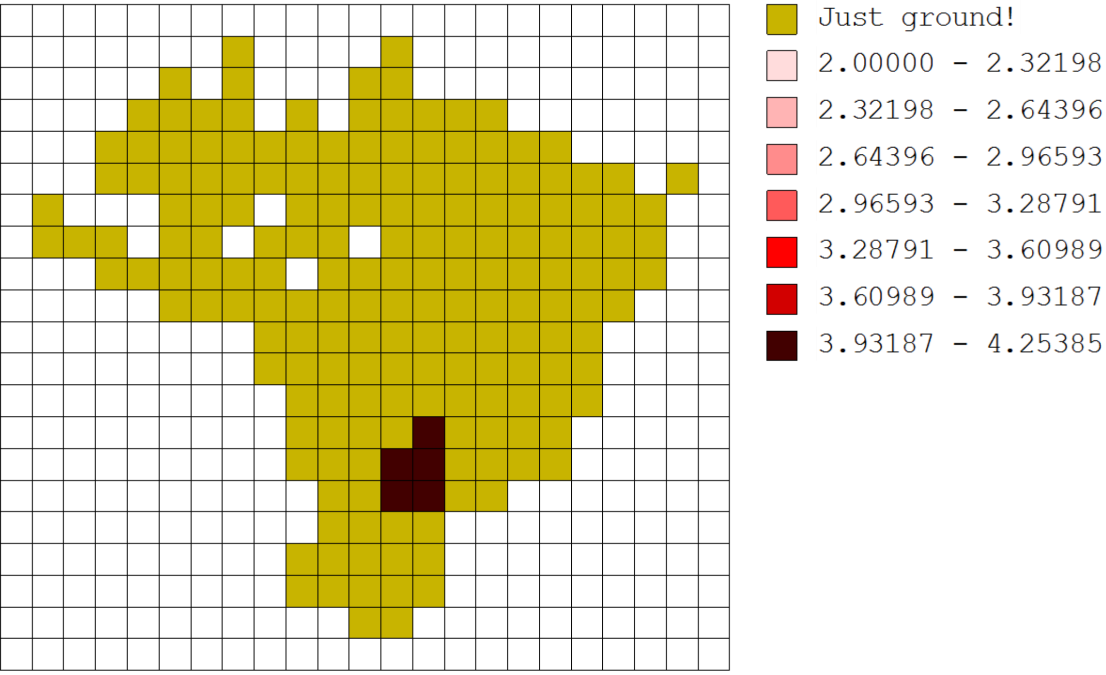

7 species give score:

Arnoliseus graciosa (0.000-0.485)
Camillina cordoba (0.000-0.833)
Isoctenus eupalaestrus (0.700-1.000)
Catanduba tuskae (0.000-0.377)
Aysha marinonii (0.000-0.750)
Eidmannella pallida (0.000-0.682)
Tmarus_pleuronotatus (0.700-1.000)

Consensus area 23 of 106 (from 9 areas; max. values)

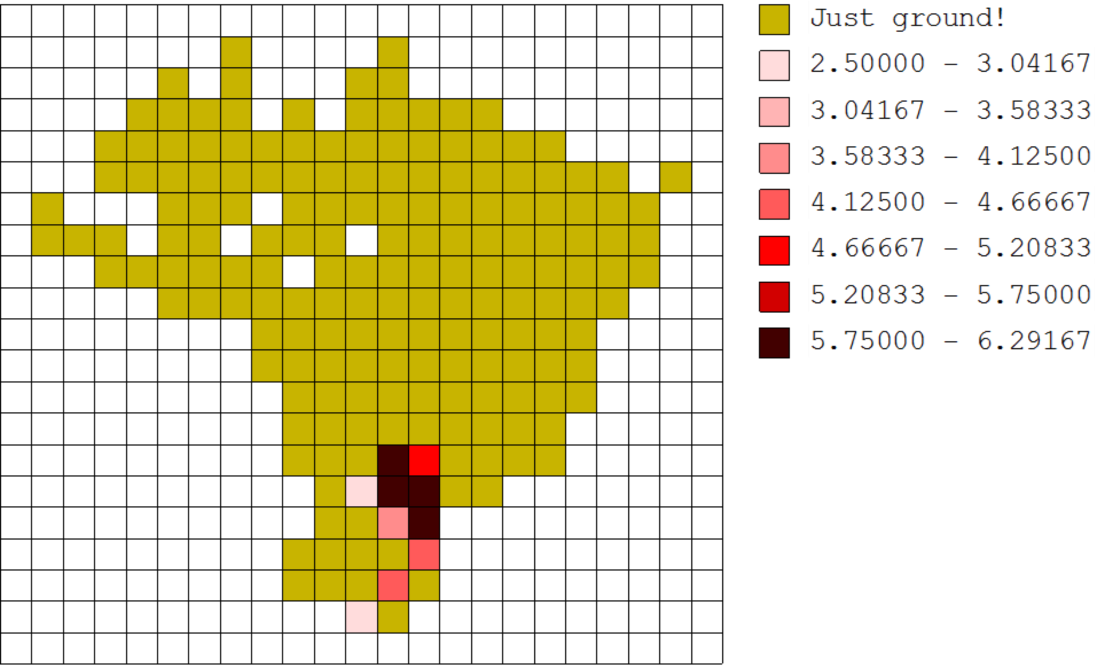

62 species give score:

Amazonepeira herrera(0.000-0.900)
Anapis castilla(0.000-0.700)
Anyphaenoides pacifica(0.000-0.700)
Architis fritzmuelleri(0.000-1.000)
Arnoliseus graciosa(0.000-0.500)
Centroctenus acara(0.000-0.700)
Centroctenus miriuma(0.000-0.613)
Corinna recurva(0.000-0.700)
Ctenus fasciatus(0.000-0.750)
Ctenus manauara(0.000-0.700)
Cyclosa vieirae(0.000-0.613)
Diplura maculata(0.000-0.656)
Dolichognatha ducke(0.000-0.700)
Dyrines ducke(0.000-0.700)
Encyosaccus sexmaculatus(0.000-0.700)
Ephebopus uatuman(0.000-0.700)
Epicratinus amazonicus(0.000-0.800)
Gelanor heraldicus(0.000-0.700)
Hingstepeira dimona(0.000-0.800)
Hingstepeira folisecens(0.000-0.800)
Hypaeus miles(0.000-0.700)
Hypaeus triplagiatus(0.000-0.700)
Hypognatha colosso(0.000-0.700)
Isoctenus eupalaestrus(0.000-0.833)
Isoctenus strandi(0.000-0.438)
Kaira echinus(0.000-0.750)
Kaira gibberosa(0.000-0.547)
Litoporus dimona(0.000-0.800)
Lygromma gasnieri(0.000-0.800)
Lygromma huberti(0.000-0.700)
Mangora mamiraua(0.000-0.700)
Mangora sumauma(0.000-0.900)
Metagonia bonaldoi(0.000-0.750)
Metazygia crabroniphila(0.000-0.375)
Metazygia uma(0.000-0.700)
Micrathena coca(0.000-0.787)
Micrathena embira(0.000-0.800)
Myrmecotypus olympus(0.000-0.700)
Ochyrocera hamadryas(0.000-0.700)
Pachomius sextus(0.000-0.700)
Parachemmis manauara(0.000-0.700)
Peucetia macroglossa(0.000-0.675)
Radulphius pintodarochai(0.000-0.750)
Rhoicinus urucu(0.000-0.700)
Scytodes balbina(0.000-0.700)
Scytodes martiusi(0.000-0.700)
Scytodes pintodarochai(0.000-0.500)
Scytodes tabuleiro(0.000-0.818)
Selenops lavillai(0.000-0.700)
Stethorrhagus lupulus(0.000-0.700)
Synotaxus waiwai(0.000-0.700)
Testudinaria bonaldoi(0.000-0.750)
Tmarus pleuronotatus(0.000-0.833)
Vitalius lucasae(0.000-0.729)
Wagneriana uzaga(0.000-0.656)
Zimiromus kleini(0.000-0.700)
Zimiromus syenus(0.000-0.700)
Breda nanica(0.000-0.700)
Metagonia bonaldoa(0.000-0.614)
Micrathena_abrahami(0.000-0.700)
Neoxyphinus petrogoblin(0.000-0.800)
Prosharmonicon maculatum(0.000-0.656)

Consensus area 24 of 106 (from 14 areas; max. values)

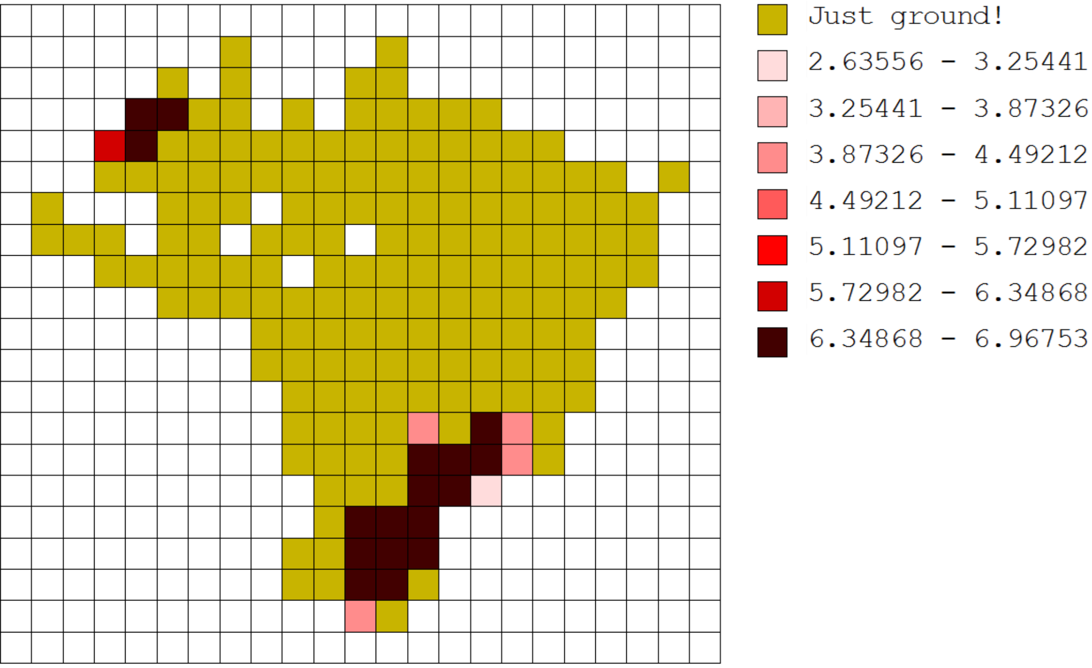

16 species give score:

Zozis_geniculata(0.000-0.800)
Alpaida_quadrilorata(0.000-0.668)
Cyclosa_camargoi(0.000-0.658)
Episinus_malachinus(0.000-0.633)
Ilargus_coccineus(0.000-0.764)
Testudinaria_lemniscata(0.000-0.735)
Theridion_plaumanni(0.000-0.649)
Vesicapalpus simplex(0.000-0.750)
Acacesia_villalobosi(0.000-0.699)
Chrysometa_boraceia(0.000-0.833)
Dipoena_variabilis(0.000-0.590)
Faiditus_striatus(0.000-0.805)
Jessica_pachecoi(0.000-0.733)
Testudinaria_unipunctata(0.000-0.875)
Theridion_quadripartitum(0.000-0.800)
Zimiromus medius(0.000-0.571)

Consensus area 25 of 106 (from 2 areas; max. values)

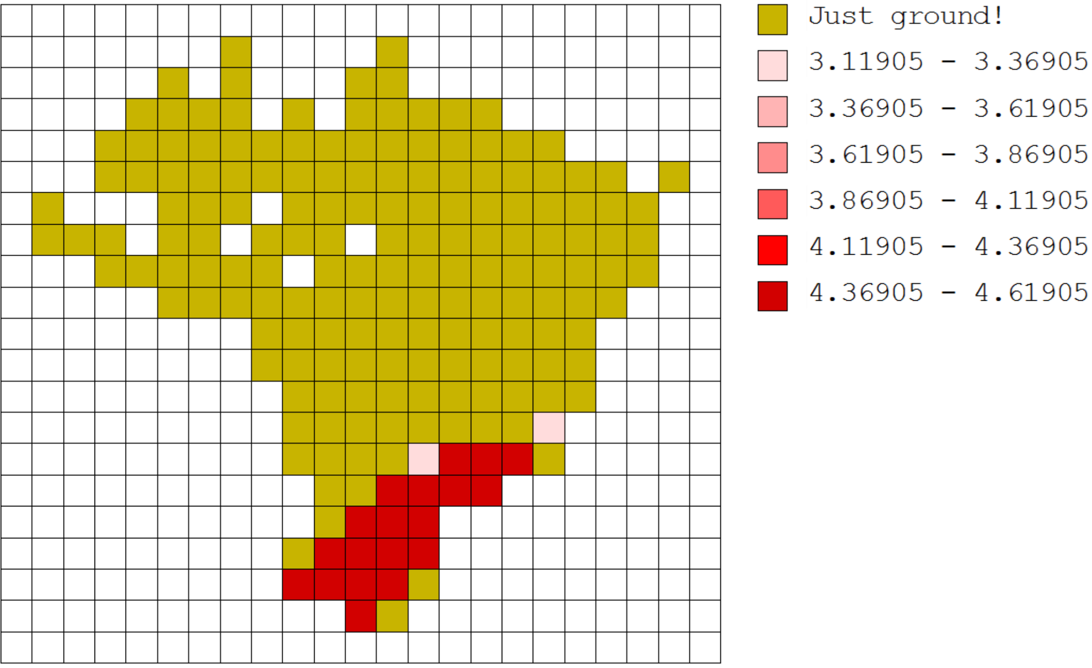

6 species give score:

Aysha_guarapuava(0.000-0.658)
Lycosa_poliostoma(0.738-0.763)
Selenops rapax(0.730-0.833)
Chrosiothes_niteroi(0.000-0.763)
Paratrechalea_galianoae(0.730-0.833)
Trechaleoides biocellata(0.714-0.737)

Consensus area 26 of 106 (from 2 areas; max. values)

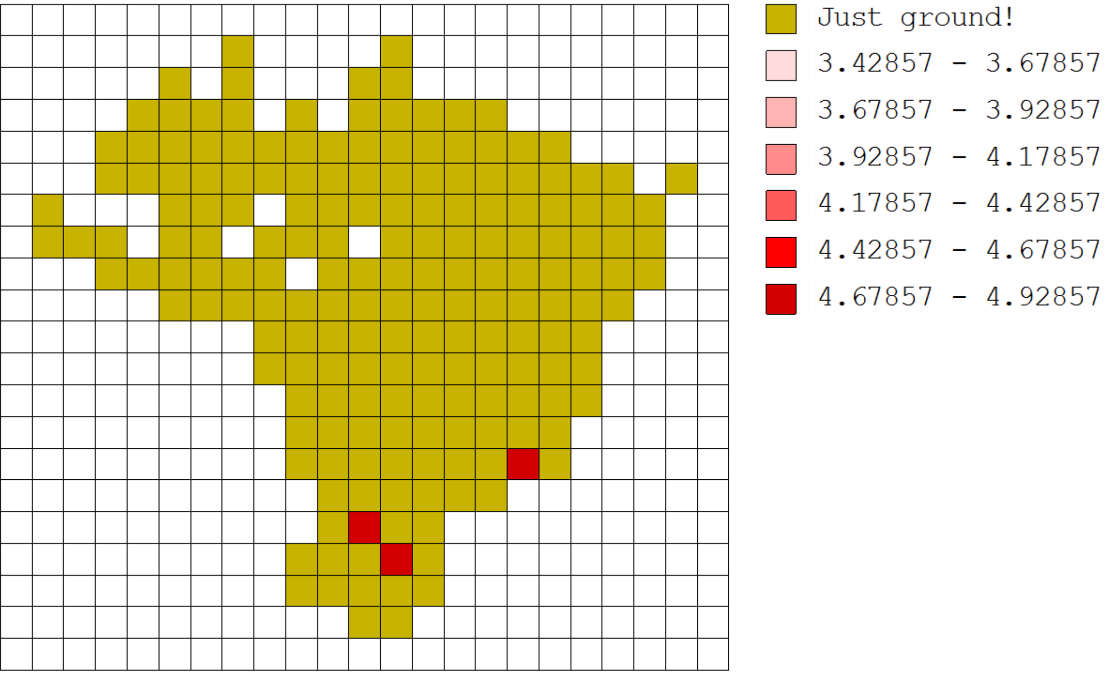

6 species give score:

Aysha taeniata (0.000-0.833)
Ero catharinae (0.714-0.889)
Tariona bruneti (0.833-1.000)
Cryptachaea bellula (0.714-0.889)
Isoctenus malabaris (0.000-0.556)
Tobias corticatus (0.833-1.000)

Consensus area 27 of 106 (from 8 areas; max. values)

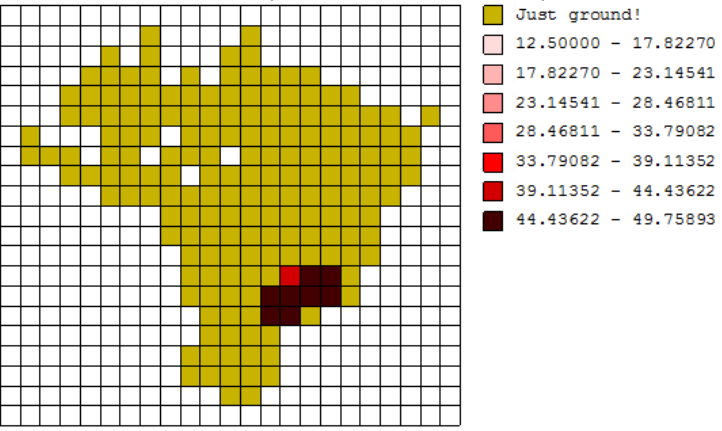

155 species give score:

Abapeba sicarioides (0.000-0.643)
Acanthoscurria gomesiana (0.000-0.643)
Alpaida angra (0.000-0.833)
Alpaida biasii (0.000-0.700)
Alpaida boraceia (0.000-0.700)
Alpaida caxias (0.000-0.688)
Amazonepeira herrera (0.000-0.900)
Anapis castilla (0.000-0.700)
Anelosimus baeza (0.000-0.625)
Anelosimus dubiosus (0.000-0.688)
Anyphaenoides pacifica (0.000-0.700)
Aphirape misionensis (0.000-0.600)
Araneus abeicus (0.000-0.625)
Architis colombo (0.000-0.589)
Arctosa pugil (0.000-0.625)
Ariadna crassipalpa (0.000-0.750)
Arnoliseus graciosa (0.000-0.700)
Atelurius segmentatus (0.000-0.750)
Aysha fortis (0.000-0.625)
Aysha robusta (0.000-0.750)
Aysha striolata (0.000-0.625)
Aysha subruba (0.000-0.446)
Azilia boudeti (0.000-0.688)
Balmaceda anulipes (0.000-1.000)
Bromelina oliola (0.000-0.589)
Caayguara catuoca (0.000-0.833)
Camillina claro (0.000-0.750)
Camillina nova (0.000-0.750)
Castianeira littoralis (0.000-1.000)
Castianeira maculata (0.000-0.625)
Centroctenus acara (0.000-0.700)
Centroctenus miriuma (0.000-0.613)
Cheiracanthium montanum (0.000-0.625)
Chrysometa sumare (0.000-0.625)
Chrysso pulchra (0.000-0.625)
Corinna bristoweana (0.000-1.000)
Corinna recurva (0.000-0.700)
Coryphasia cardoso (0.000-0.625)
Coryphasia melloleitaoi (0.000-0.750)
Cryptachaea eramus (0.000-0.625)
Cryptachaea jequirituba (0.000-1.000)
Ctenus fasciatus (0.000-0.600)
Ctenus manauara (0.000-0.700)
Cyclosa vieirae (0.000-0.613)
Dipoena santacatarinae (0.000-1.000)
Dolichognatha ducke (0.000-0.700)
Dyrines ducke (0.000-0.700)
Eidmannella pallida (0.000-0.600)
Eilica maculipes (0.000-0.800)
Encyosaccus sexmaculatus (0.000-0.700)
Enna caparao (0.000-0.536)
Ephebopus uatuman (0.000-0.700)
Epicadus rubripes (0.000-0.625)
Epicratinus amazonicus (0.000-0.800)
Eurymorion nobile (0.000-0.700)
Eutichurus ibiuna (0.000-0.700)
Faiditus acuminatus (0.000-0.589)
Gelanor heraldicus (0.000-0.700)
Heliconius antiochus (0.000-1.000)
Hingstepeira dimona (0.000-0.800)
Hingstepeira folisecens (0.000-0.800)
Homoeomma montanum (0.000-0.833)
Hypaeus miles (0.000-0.700)
Hypaeus triplagiatus (0.000-0.700)
Hypognatha colosso (0.000-0.700)
Isoctenus eupalaestrus (0.000-0.750)
Isoctenus janeirus (0.000-0.813)
Itatiaya modesta (0.000-0.778)
Jessica campesina (0.000-0.536)
Litoporus dimona (0.000-0.800)
Loxosceles adelaida (0.000-0.544)
Lygromma gasnieri (0.000-0.800)
Lygromma huberti (0.000-0.700)
Lyssomanes boraceia (0.000-0.800)
Mangora blumenau (0.000-0.589)
Mangora mamiraua (0.000-0.700)
Mangora sumauma (0.000-0.900)
Mastophora_felis (0.000-0.625)
Mesabolivar cavicelatus (0.000-0.833)
Mesabolivar cyaneomaculatus (0.000-0.688)
Metazygia uma (0.000-0.700)
Micrathena coca (0.000-0.787)
Micrathena embira (0.000-0.800)
Myrmarachne brasiliensis (0.000-0.688)
Myrmecotypus olympus (0.000-0.700)
Neostothis gigas (0.000-0.778)
Neotama cunhabebe (0.000-0.536)
Neriene redacta (0.000-0.750)
Ochyrocera hamadryas (0.000-0.700)
Osoriella rubella (0.000-0.536)
Pachomius sextus (0.000-0.700)
Parachemmis manauara (0.000-0.700)
Paradiestus aurantiacus (0.000-0.800)
Pensacolops rubrovittata (0.000-0.750)
Peucetia macroglossa (0.000-0.675)
Phiale bipunctata (0.000-0.700)
Phoroncidia rubromaculata (0.000-0.625)
Plesiopelma insulare (0.000-0.648)
Poecilochroa trifasciata (0.000-0.625)
Prorachias bristowei (0.000-0.833)
Pycnothele perdita (0.000-0.625)
Radulphius bicolor (0.000-0.625)
Radulphius lane (0.000-1.000)
Rhoicinus urucu (0.000-0.700)
Scytodes antonina (0.000-0.600)
Scytodes balbina (0.000-0.700)
Scytodes itapecerica (0.000-1.000)
Scytodes martiusi (0.000-0.700)
Scytodes nambiobyrassu (0.000-0.700)
Scytodes vassununga (0.000-0.700)
Scytodes vittata (0.000-0.688)
Selenops lavillai (0.000-0.700)
Selenops zumac (0.000-0.700)
Sphecozone venialis (0.000-0.536)
Stemmops vicosa (0.000-0.625)
Stenoterommata maculata (0.000-0.589)
Stethorrhagus lupulus (0.000-0.700)
Symphytognatha carstica (0.000-0.495)
Synema bellum (0.000-0.700)
Synotaxus waiwai (0.000-0.700)
Theridion evexum (0.000-0.625)
Thymoites palo (0.000-1.000)
Tidarren sisyphoides (0.000-0.625)
Titidiops melanosternus (0.000-0.625)
Tmarus albifrons (0.000-0.750)
Tmarus alticola (0.000-0.625)
Tmarus bisectus (0.000-0.750)
Tmarus mutabilis (0.000-0.750)
Tmarus pizai (0.000-1.000)
Tmarus pleuronotatus (0.000-0.750)
Tmarus primitivus (0.000-0.625)
Tobias gradiens (0.000-0.625)
Trachelopachys ammobates (0.000-0.625)
Trochosa pardaloides (0.000-0.589)
Trogloneta cantareira (0.000-0.580)
Vitalius buecherli (0.000-0.929)
Vitalius lucasae (0.000-0.467)
Wirada tijuca (0.000-0.625)
Zimiromus_kleini (0.000-0.700)
Zimiromus_syenus (0.000-0.700)
Acanthoctenus rubritaeniatus (0.000-0.625)
Breda nanica (0.000-0.700)
Caayguara ybytyriguara (0.000-0.750)
Castianeira pyriformis (0.000-0.625)
Centroctenus sai (0.000-1.000)
Homeomma montanum (0.000-0.833)
Itatiaya apidema (0.000-0.625)
Lyssomanes leucomellas (0.000-0.589)
Micrathena abrahami (0.000-0.700)
Micrathena ruschii (0.000-0.491)
Neoxyphinus keyserlingi (0.000-0.696)
Neoxyphinus petrogoblin (0.000-0.800)
Olios hieroglyphicus (0.000-0.625)
Predatoroonops_mctiernani (0.000-0.491)
Predatoroonops peterhalli (0.000-0.778)

Consensus area 28 of 106 (from 1 areas; max. values)

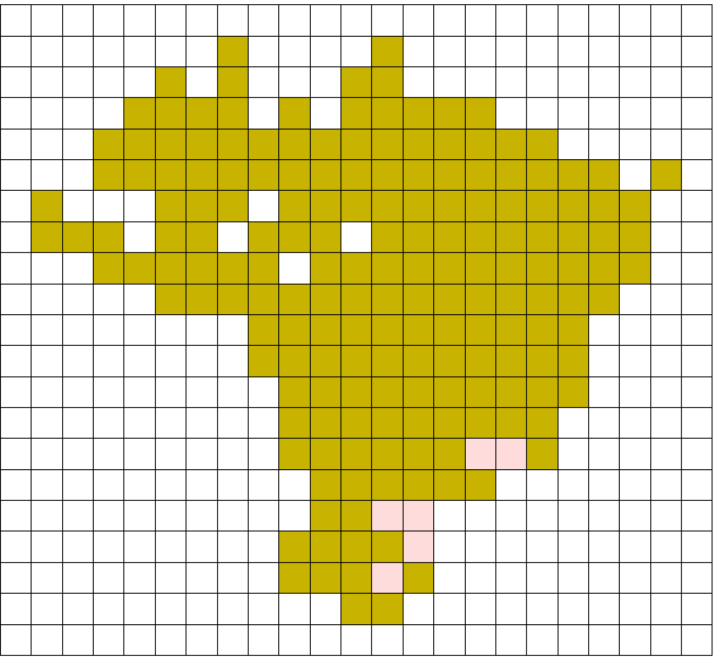

Just ground!

6.53704 - 6.78704

12 species give score:

Ameridion_unanimum(0.593)
Berlandiella_polyacantha(0.222)
Fernandezina_tijuca(0.667)
Saitis_cyanipes(0.519)
Tutaibo_velox(0.741)
Exocora girotii(0.583)
Arnoliseus_calcarifer(0.500)
Craspedisia_cornuta(0.500)
Olios_caprinus(0.667)
Theridion_opolon(0.583)
Wagneriana_heteracantha(0.370)
Exocora ribeiroi(0.593)

Consensus area 29 of 106 (from 1 areas; max. values)

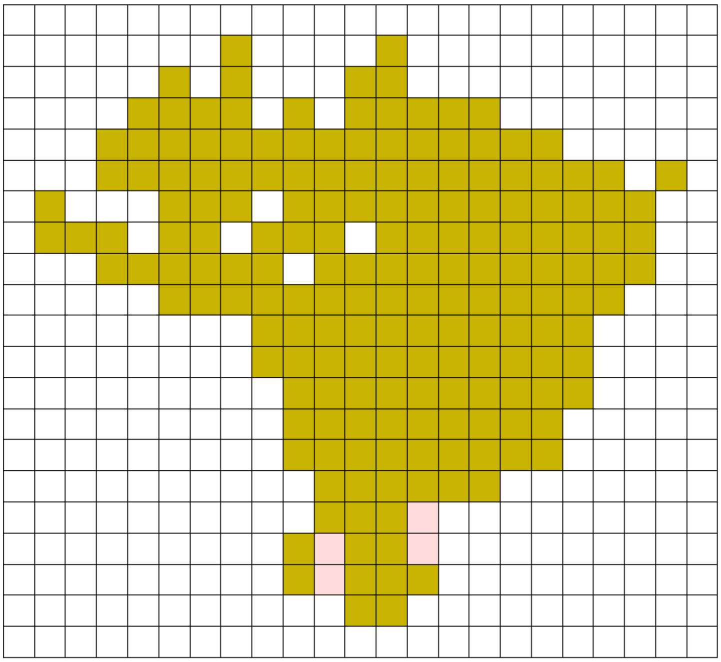

Just ground!

2.35714 - 2.60714

4 species give score:

Eustala photographica(0.321)
Synaemops notabilis(0.643)
Gamasomorpha m-scripta(0.643)
Stenoteromata palmar(0.750)

Consensus area 30 of 106 (from 6 areas; max. values)

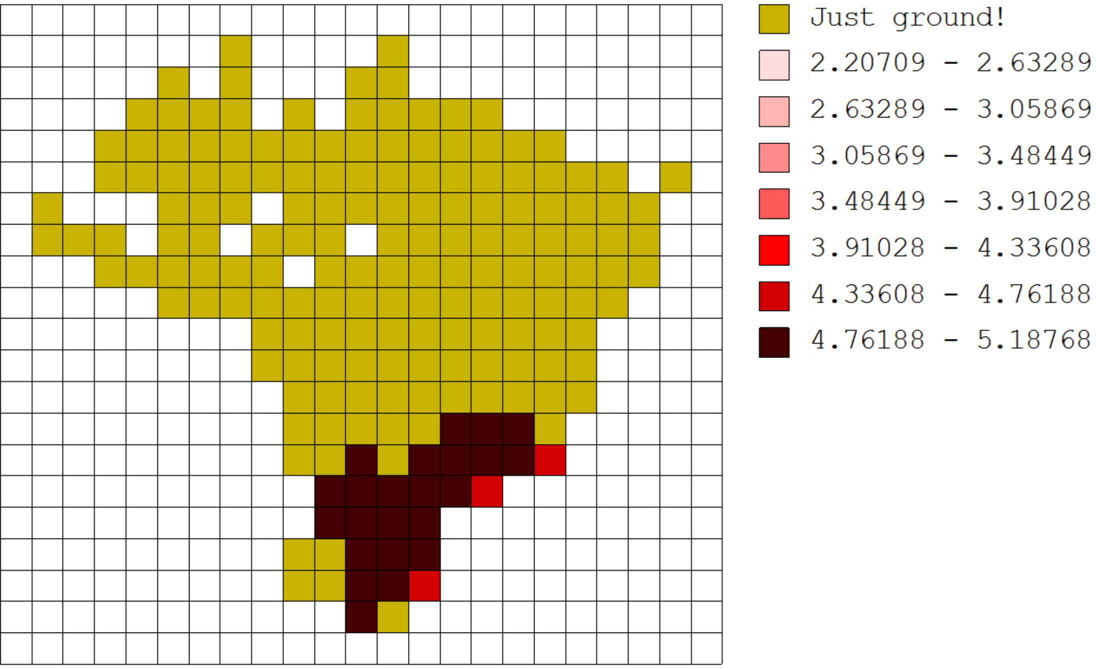

14 species give score:

Alpaida grayi (0.385-0.826)
Bertrana rufostriata (0.000-0.462)
Corinna mourai (0.000-0.769)
Isoctenus malabaris (0.000-0.708)
Mangora sobradinho (0.000-0.639)
Metagonia argentinensis (0.000-0.483)
Vitalius longisternalis (0.000-0.571)
Araneus omnicolor (0.000-0.813)
Corinna colombo (0.000-0.525)
Eustala sagana (0.000-0.750)
Isoctenus ordinario (0.000-0.436)
Mesabolivar luteus (0.000-0.750)
Micrathena spitzi (0.000-0.769)
Wagneriana heteracantha (0.000-0.737)

Consensus area 31 of 106 (from 4 areas; max. values)

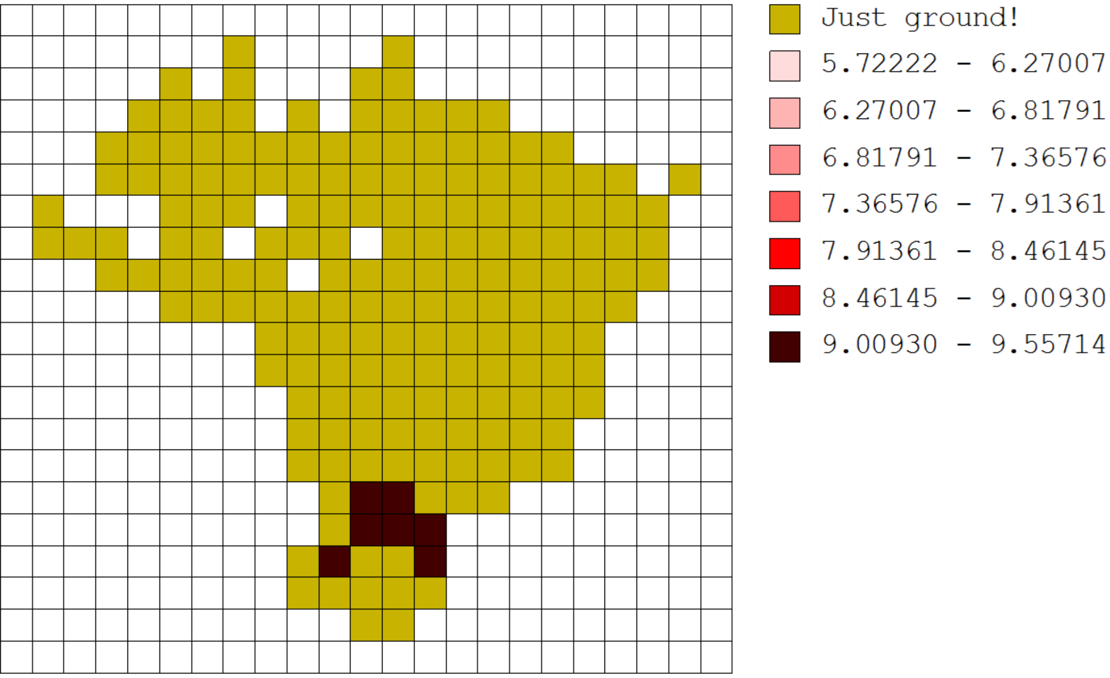

23 species give score:

Cryptachaea isana (0.000-0.614)
Diplura maculata (0.000-0.564)
Dipoena foliata (0.564-0.694)
Gamasomorpha m-scripta (0.000-0.600)
Guaraniella bracata (0.000-0.400)
Guaraniella mahnerti (0.000-0.500)
Helvetia humillima (0.667-0.833)
Isoctenus charada (0.667-0.833)
Isoctenus strandi (0.278-0.577)
Moyosi prativaga (0.000-0.400)
Neoxyphinus ogloblini (0.000-0.500)
Nyetnops guarani (0.000-0.750)
Scytodes chapeco (0.000-0.400)
Scytodes imbituba (0.000-0.667)
Scytodes maquine (0.000-0.288)
Scytodes tabuleiro (0.000-0.705)
Thymoites puer (0.462-0.556)
Trachelopachys gracilis (0.564-0.694)
Tupigea lisei (0.564-0.694)
Anelosimus decoloratus (0.000-0.400)
Eustala_crista (0.068-0.200)
Metagonia_bonaldoa (0.750-1.000)
Prosharmonicon maculatum (0.000-0.564)

Consensus area 32 of 106 (from 2 areas; max. values)

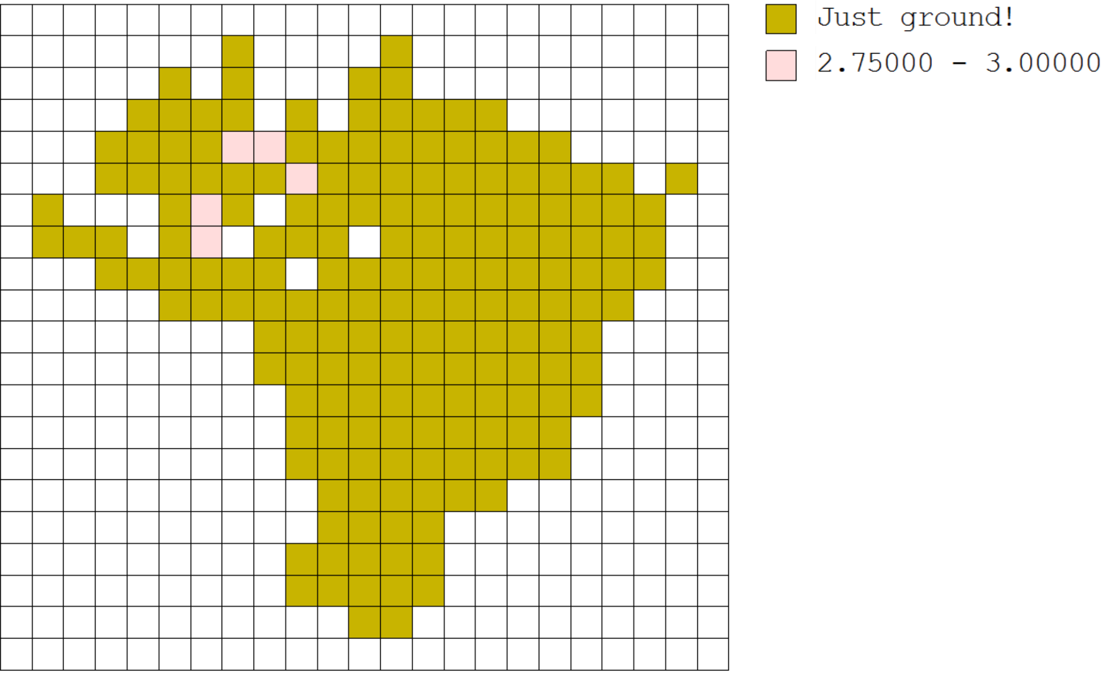

5 species give score:
Ancylometes hewitsoni (0.000-0.583)
Lyssomanes benderi (0.000-0.750)
Scytodes curupira (0.656-0.667)
Breda variolosa (0.750)
Psecas_viridipurpureus (0.750)

Consensus area 33 of 106 (from 2 areas; max. values)

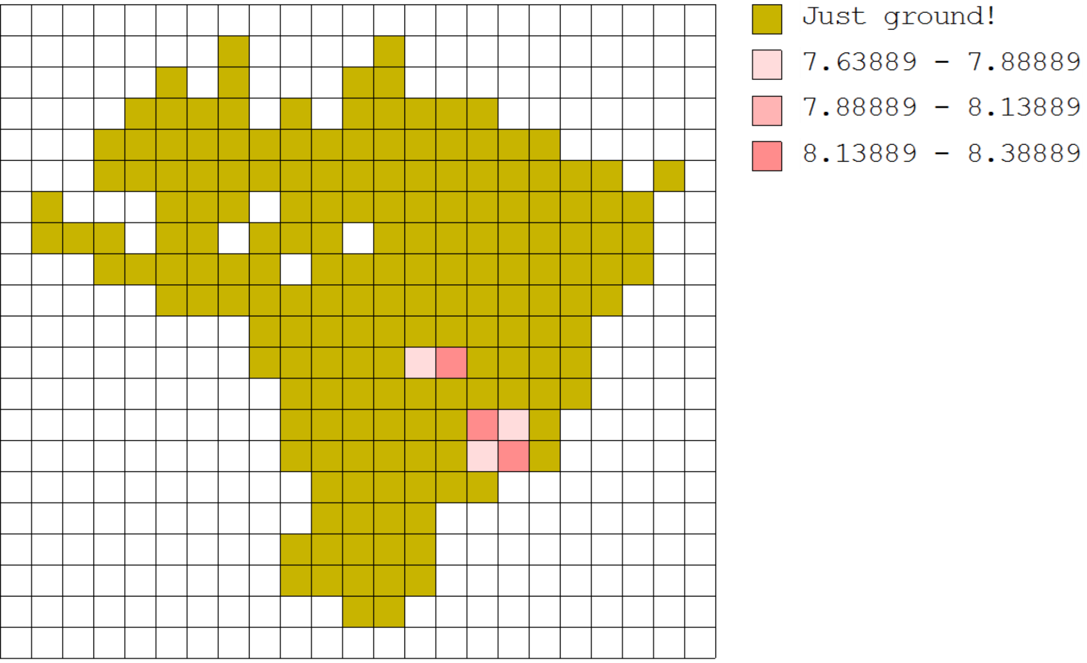

12 species give score:
Argiope_legionis (0.667-0.833)
Corinna_loricata (0.000-0.519)
Cyrtauchenius_maculatus (0.750-1.000)
Hogna_sternalis (0.667-0.875)
Lyssomanes_miniaceus (0.593-0.729)
Teudis tensipes (0.667-0.833)
Attacobius_attarum (0.519-0.625)
Corinna_phalerata (0.667-0.833)
Cyrtophora_citricola (0.000-0.593)
Leucauge_formosa (0.667-0.833)
Neodiplothele_fluminensis (0.667-0.833)
Paradiestus egregia (0.667-0.833)

Consensus area 34 of 106 (from 5 areas; max. values)

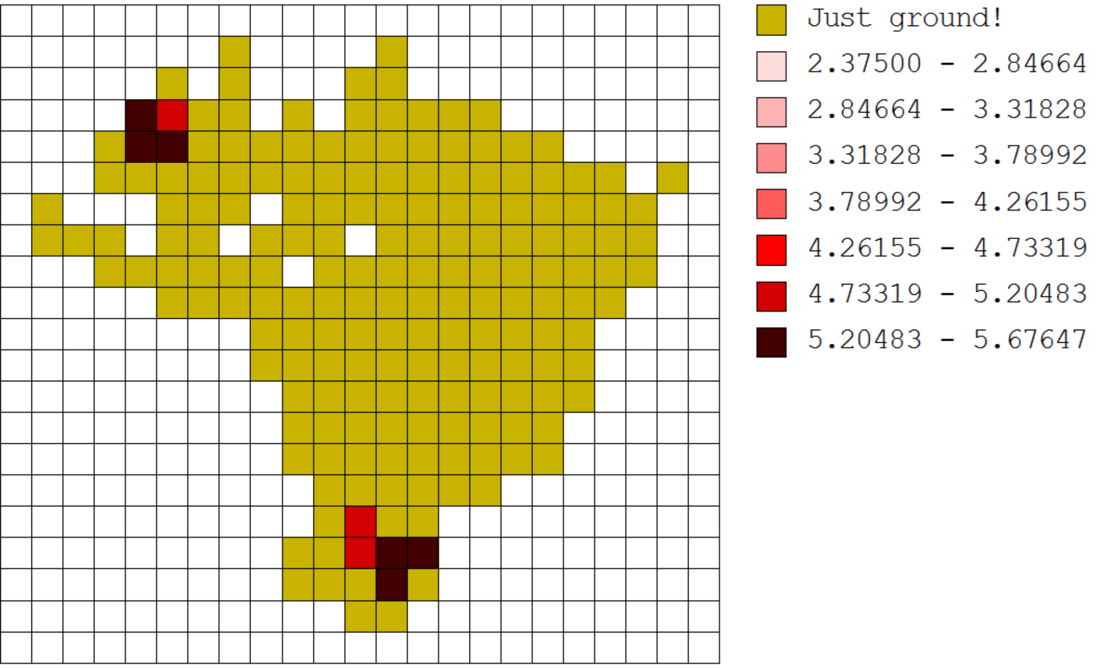

54 species give score:

Alpaida nigrofrenata (0.000-0.650)
Anapis castilla (0.000-0.700)
Beata germaini (0.000-0.619)
Centroctenus miriuma (0.000-0.613)
Corinna recurva (0.000-0.700)
Cyclosa vieirae (0.000-0.613)
Dyrines ducke (0.000-0.700)
Encyosaccus sexmaculatus (0.000-0.700)
Epicratinus amazonicus (0.000-0.800)
Gelanor heraldicus (0.000-0.700)
Hingstepeira folisecens (0.000-0.800)
Hypaeus triplagiatus (0.000-0.700)
Litoporus dimona (0.000-0.800)
Lygromma huberti (0.000-0.700)
Mangora sumauma (0.000-0.900)
Micrathena coca (0.000-0.787)
Myrmecotypus olympus (0.000-0.700)
Pachomius sextus (0.000-0.700)
Peucetia macroglossa (0.000-0.675)
Scytodes balbina (0.000-0.700)
Selenops lavillai (0.000-0.700)
Stethorrhagus lupulus (0.000-0.700)
Synotaxus waiwai (0.000-0.700)
Testudinaria gravatai (0.000-0.750)
Zimiromus kleini (0.000-0.700)
Breda nanica (0.000-0.700)
Micrathena abrahami (0.000-0.700)
Amazonepeira herrera (0.000-0.900)
Anyphaenoides pacifica (0.000-0.700)
Centroctenus acara (0.000-0.700)
Chirothecia semiornata (0.000-0.679)
Ctenus manauara (0.000-0.700)
Dolichognatha ducke (0.000-0.700)
Encolpius guaraniticus (0.000-0.667)
Ephebopus uatuman (0.000-0.700)
Eustala palmares (0.000-0.569)
Hingstepeira dimona (0.000-0.800)
Hypaeus miles (0.000-0.700)
Hypognatha colosso (0.000-0.700)
Lygromma gasnieri (0.000-0.800)
Mangora mamiraua (0.000-0.700)
Metazygia uma (0.000-0.700)
Micrathena embira (0.000-0.800)
Ochyrocera hamadryas (0.000-0.700)
Parachemmis manauara (0.000-0.700)
Rhoicinus urucu (0.000-0.700)
Scytodes martiusi (0.000-0.700)
Sidymella spinifera (0.000-0.750)
Synema nitidulum (0.000-0.750)
Tacuna minensis (0.000-0.750)
Tobias monstrosus (0.000-0.675)
Zimiromus syenus (0.000-0.700)
Dipoena santacaterinae (0.000-0.688)
Neoxyphinus petrogoblin (0.000-0.800)

Consensus area 35 of 106 (from 4 areas; max. values)

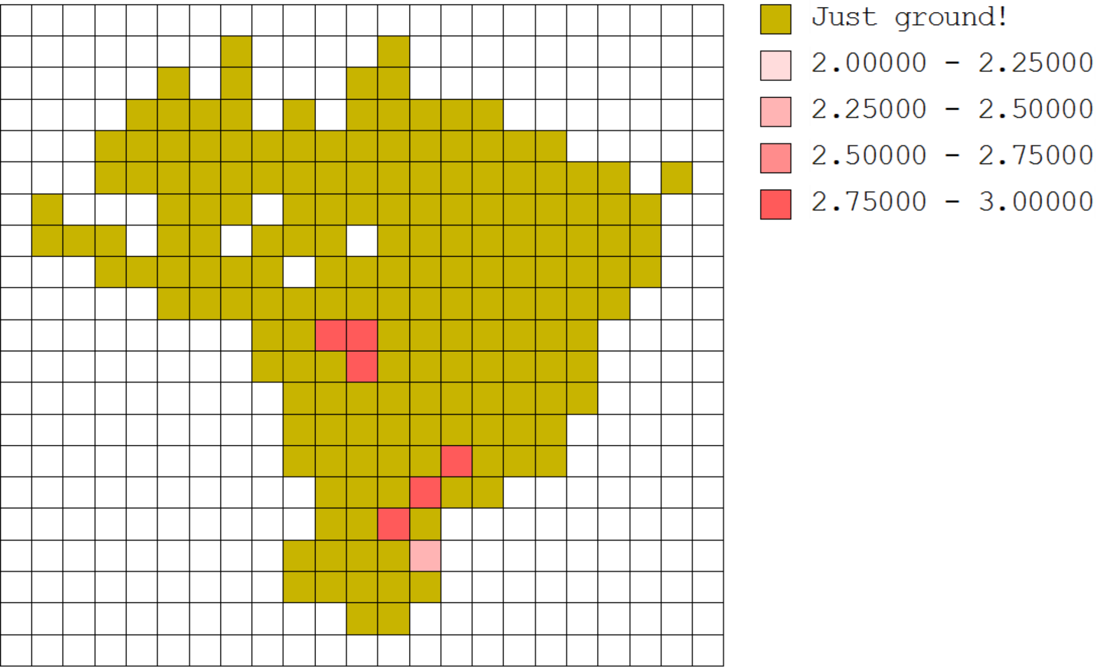

48 species give score:

Actinopus paranensis(0.000-1.000)
Amazonepeira herrera(0.000-0.900)
Anapis castilla(0.000-0.700)
Anyphaenoides pacifica(0.000-0.700)
Centroctenus acara(0.000-0.700)
Centroctenus miriuma(0.000-0.613)
Corinna recurva(0.000-0.700)
Ctenus manauara(0.000-0.700)
Cybaeodamus ornatus(0.000-0.833)
Cyclosa vieirae(0.000-0.613)
Dolichognatha ducke(0.000-0.700)
Dyrines ducke(0.000-0.700)
Encyosaccus sexmaculatus(0.000-0.700)
Ephebopus uatuman(0.000-0.700)
Epicratinus amazonicus(0.000-0.800)
Gelanor heraldicus(0.000-0.700)
Hingstepeira dimona(0.000-0.800)
Hingstepeira folisecens(0.000-0.800)
Hypaeus miles(0.000-0.700)
Hypaeus triplagiatus(0.000-0.700)
Hypognatha colosso(0.000-0.700)
Litoporus dimona(0.000-0.800)
Lygromma gasnieri(0.000-0.800)
Lygromma huberti(0.000-0.700)
Mangora mamiraua(0.000-0.700)
Mangora sumauma(0.000-0.900)
Metaphidippus odiosus(0.000-1.000)
Metazygia uma(0.000-0.700)
Micrathena coca(0.000-0.787)
Micrathena embira(0.000-0.800)
Myrmecotypus olympus(0.000-0.700)
Ochyrocera hamadryas(0.000-0.700)
Pachomius sextus(0.000-0.700)
Parachemmis manauara(0.000-0.700)
Peucetia macroglossa(0.000-0.675)
Rhoicinus urucu(0.000-0.700)
Scytodes balbina(0.000-0.700)
Scytodes martiusi(0.000-0.700)
Selenops lavillai(0.000-0.700)
Stethorrhagus_lupulus(0.000-0.700)
Synema pereirai(0.000-0.685)
Synemosyna myrmeciaeformis(0.000-0.685)
Synotaxus waiwai(0.000-0.700)
Zimiromus kleini(0.000-0.700)
Zimiromus syenus(0.000-0.700)
Breda nanica(0.000-0.700)
Micrathena abrahami(0.000-0.700)
Neoxyphinus petrogoblin(0.000-0.800)

Consensus area 36 of 106 (from 5 areas; max. values)

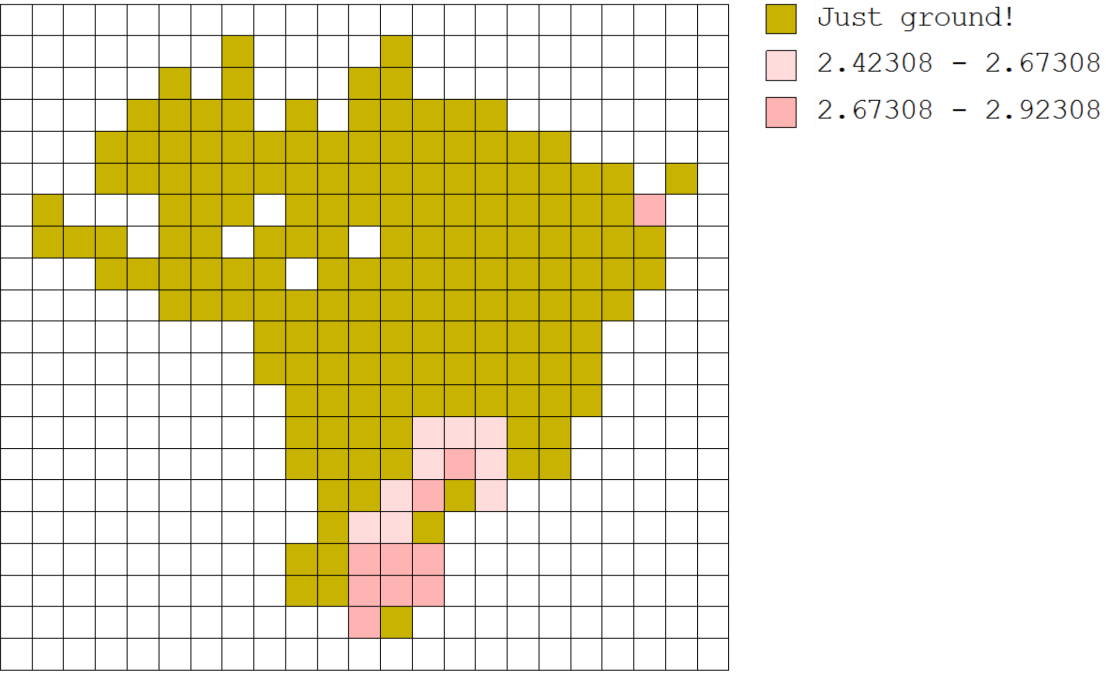

5 species give score:

Eustala minuscula (0.616-0.923)
Eustala perfida (0.595-0.808)
Larinia bivittata (0.000-0.750)
Senoculus_monastoides (0.000-0.688)
Urarachne longa (0.000-0.643)

Consensus area 37 of 106 (from 5 areas; max. values)

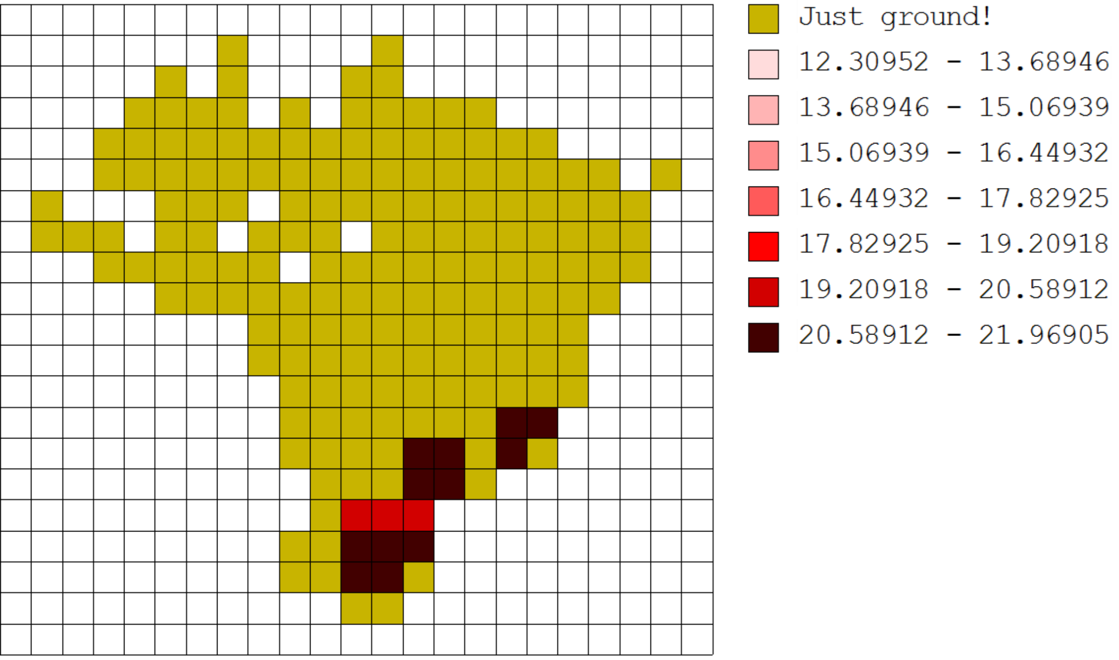

49 species give score:

Alpaida grayi (0.000-0.347)	Alpaida iguazu (0.000-0.667)
Anelosimus nigrescens (0.000-0.571)	Anodoration claviferum (0.000-0.708)
Arachosia praesignis (0.000-0.601)	Araneus orgaos (0.607-0.800)
Araneus sicki (0.573-0.724)	Araneus workmani (0.048-0.553)
Arnoliseus calcarifer (0.000-0.649)	Aysha rubromaculata (0.386-0.636)
Berlandiella polyacantha (0.419-0.625)	Bertrana rufostriata (0.000-0.561)
Buckupiella imperatriz (0.000-0.724)	Chrysometa cambara (0.679-1.000)
Chrysso compressa (0.000-0.649)	Corinna mourai (0.000-0.611)
Craspedisia cornuta (0.464-0.714)	Cryptachaea passiva (0.571-0.750)
Epilineutes globosus (0.607-0.800)	Episinus teresopolis (0.607-0.800)
Eustala sagana (0.000-0.462)	Faiditus sicki (0.607-0.800)
Frigga quintensis (0.000-0.724)	Ianduba varia (0.214-0.764)
Isoctenus ordinario (0.000-0.594)	Lyssomanes tristis (0.000-0.520)
Mangora strenua (0.000-0.575)	Mastophora carpogaster (0.536-0.625)
Mesabolivar luteus (0.000-0.556)	Micrathena spitzi (0.000-0.531)
Ocrepeira malleri (0.000-0.614)	Olios caprinus (0.000-0.588)
Paratrechalea galianoae (0.000-0.510)	Patrera longipes (0.267-0.636)
Patrera procera (0.000-0.747)	Phoneutria keyserlingi (0.000-0.536)
Psilocymbium lineatum (0.000-0.800)	Rhyphelia variegata (0.000-0.636)
Saitis cyanipes (0.000-0.700)	Steatoda grossa (0.000-0.581)
Synemosyna aurantiaca (0.557-0.786)	Theridion opolon (0.000-0.679)
Trechaleoides biocellata (0.000-0.660)	Triplogyna ignitula (0.000-0.614)
Wagneriana dimastophora (0.000-0.750)	Zimiromus montenegro (0.000-0.664)
Berlandiella meridionalis (0.000-0.588)	Cryptachaea_taim (0.000-0.510)
Exocora girotii (0.000-0.633)	

Consensus area 38 of 106 (from 2 areas; max. values)

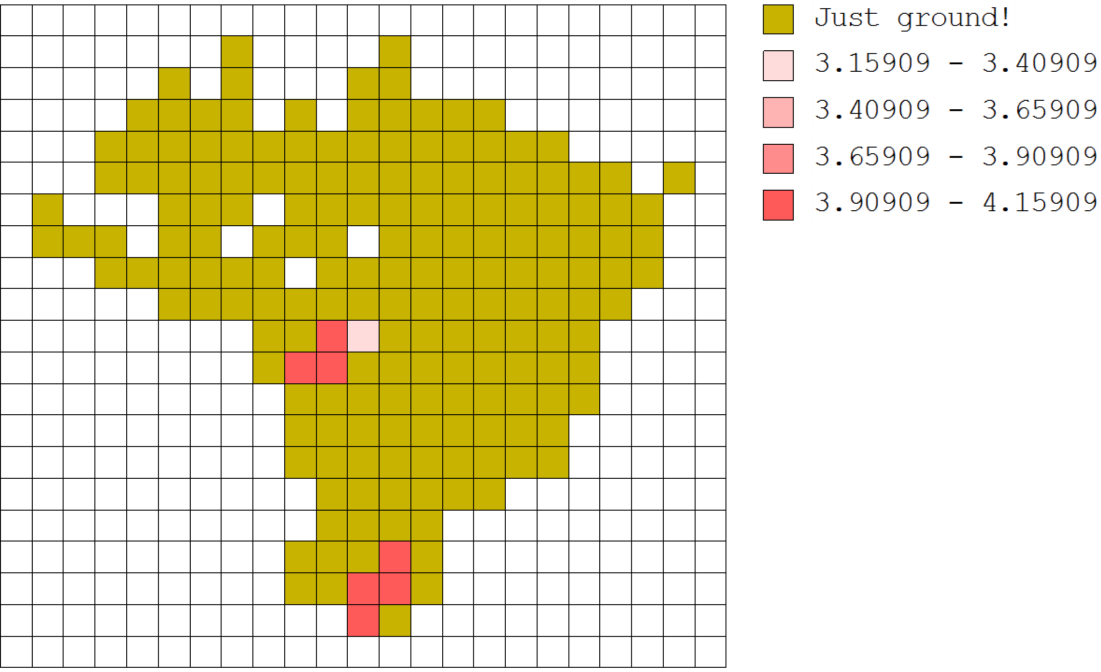

8 species give score:

Cyclosa walckenaeri (0.000-0.667)
Gea heptagon (0.714-0.750)
Micrathena furva (0.530-0.600)
Scoloderus gibber (0.579-0.606)
Gastromicans noxiosa (0.000-0.714)
Labicymbium rusticulum (0.000-0.643)
Oxyopes birabeni (0.000-0.606)
Phoroncidia piratini (0.000-0.857)

Consensus area 39 of 106 (from 4 areas; max. values)

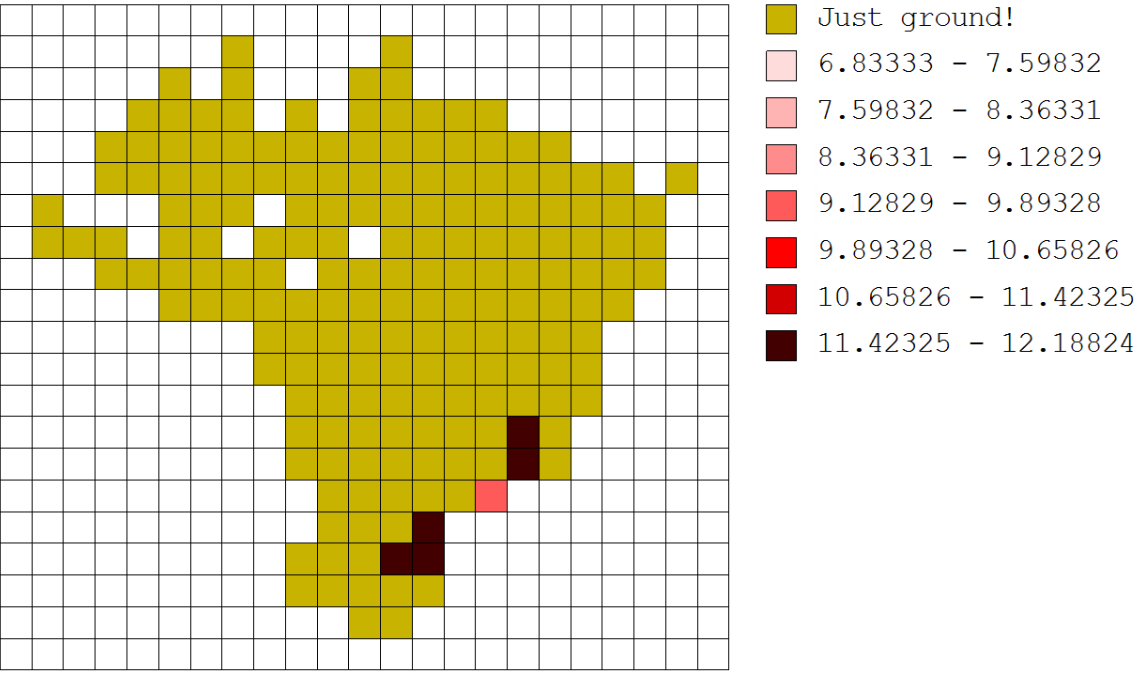

23 species give score:

Ameridion unanimum (0.000-0.635)
Dipoena pusilla (0.700-1.000)
Epilineutes globosus (0.000-0.618)
Exalbidion sexmaculatum (0.700-1.000)
Mangora blumenau (0.519-0.667)
Neospintharus obscurus (0.700-1.000)
Onocolus infelix (0.000-0.476)
Spermophora maculata (0.700-1.000)
Stenoterommata maculata (0.519-0.667)
Tobias corticatus (0.000-0.700)
Trochosa pardaloides (0.000-0.556)
Exocora girotii (0.000-0.453)
Cryptachaea bellula (0.000-0.706)
Enoploctenus maculipes (0.000-0.618)
Ero catharinae (0.000-0.706)
Fernandezina tijuca (0.000-0.750)
Mastophora ypiranga (0.000-0.700)
Olios caprinus (0.000-0.519)
Saitis cyanipes (0.000-0.519)
Sphecozone venialis (0.000-0.694)
Tariona bruneti (0.000-0.700)
Toca samba (0.618-0.833)
Xiruana_affinis (0.519-0.706)

Consensus area 40 of 106 (from 1 areas; max. values)

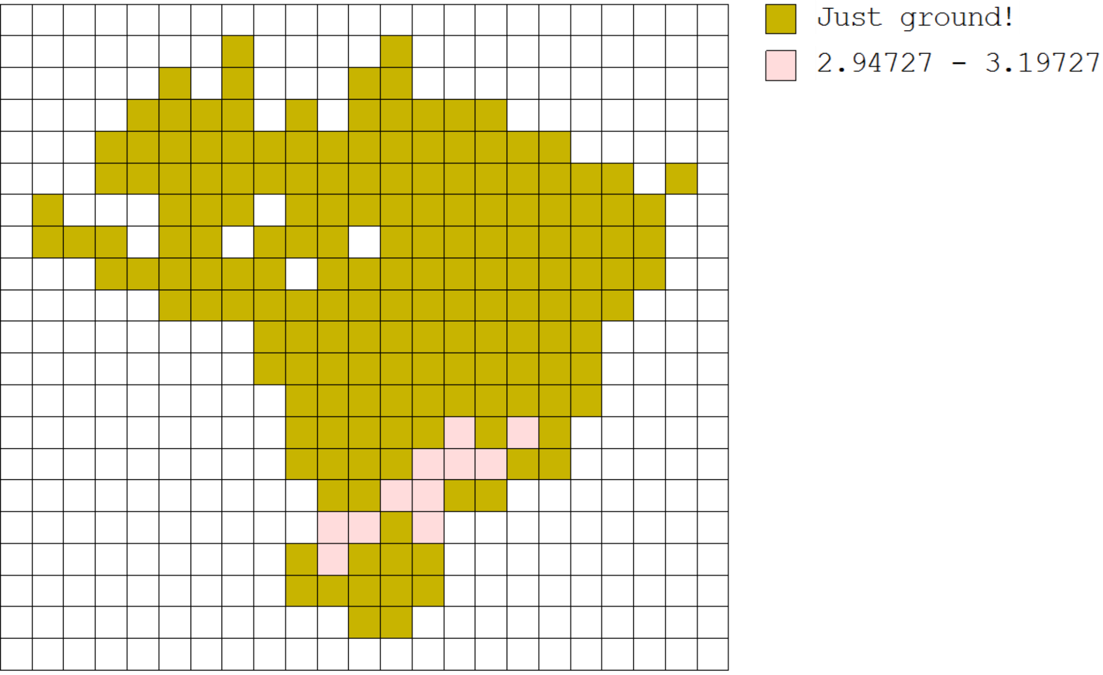

5 species give score:

Aysha guarapuava (0.573)
Caayguara cupepemassu (0.518)
Dipoena granulata (0.682)
Isoctenus_ordinario (0.622)
Berlandiella robertae (0.553)

Consensus area 41 of 106 (from 1 areas; max. values)

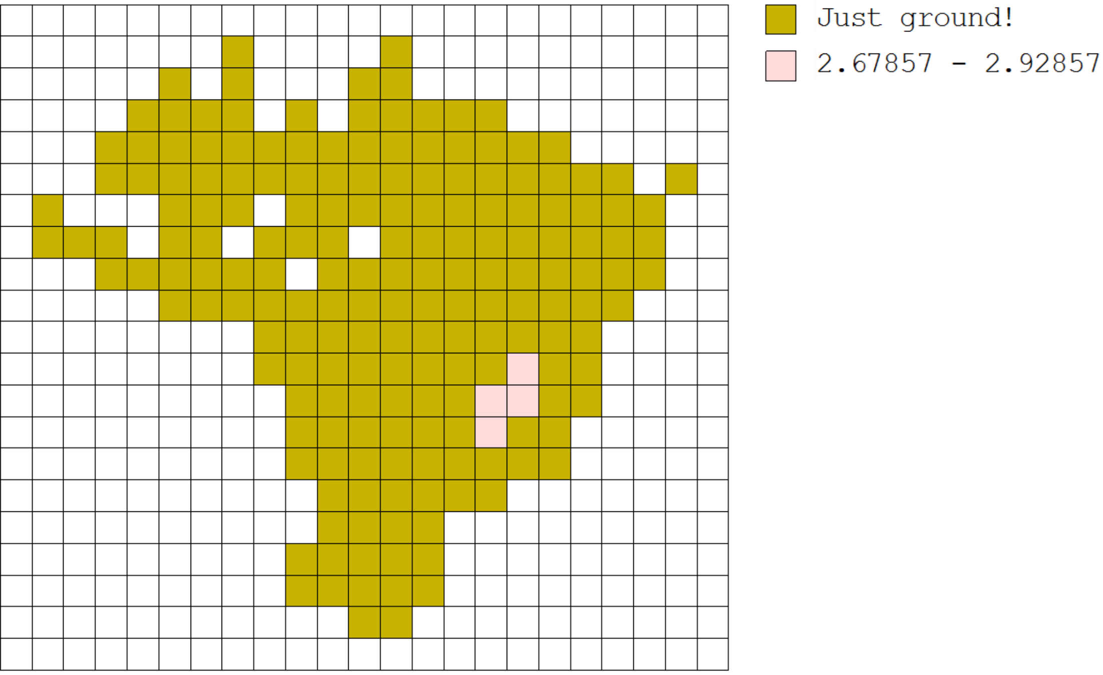

4 species give score:

Araneus fronki (0.643)
Scytodes mangabeiras (0.643)
Scytodes tyaiapyssanga (0.643)
Scytodes upia (0.750)

Consensus area 42 of 106 (from 2 areas; max. values)

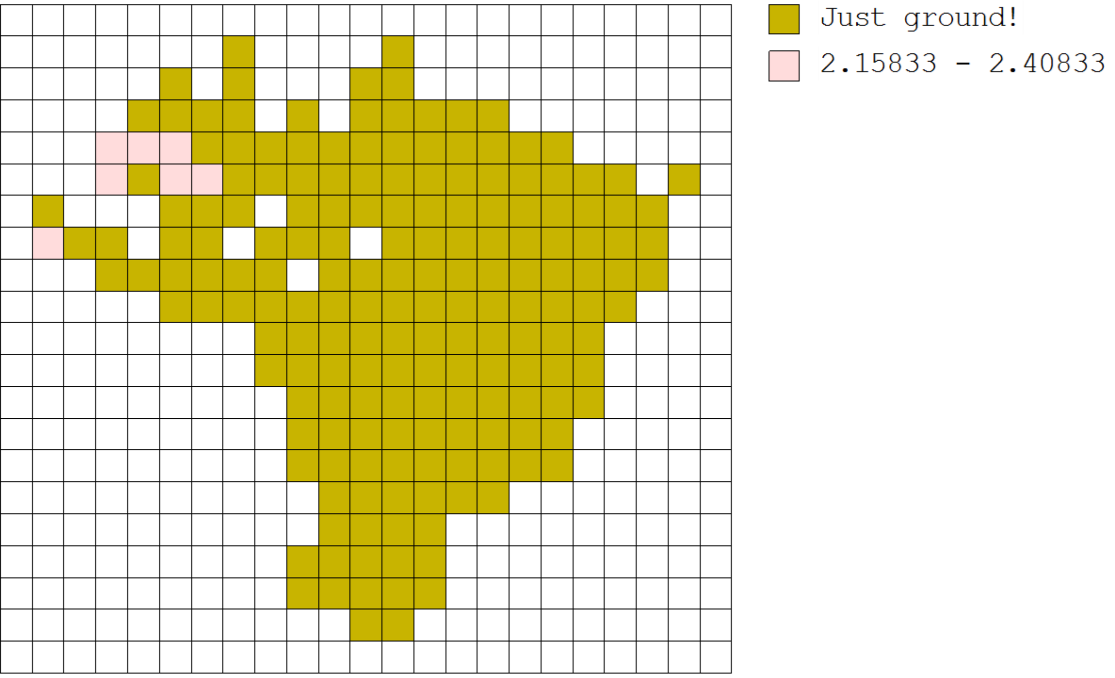

3 species give score:
Cyriocosmus sellatus(0.758-0.786)
Mangora_mathani(0.642-0.655)
Mangora tefe(0.758-0.786)

Consensus area 43 of 106 (from 3 areas; max. values)

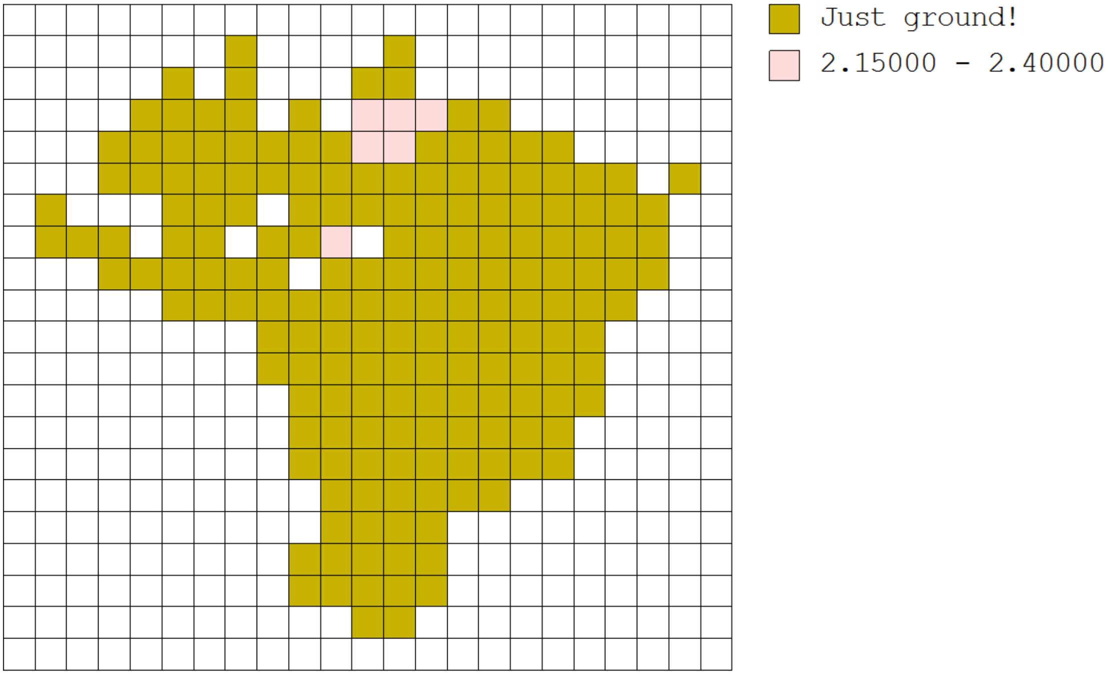

4 species give score:
Micrathena armigera(0.750)
Myrmarachne mocamboensis(0.000-0.750)
Parachemmis hassleri(0.750)
Fufius auricomis(0.000-0.656)

Consensus area 44 of 106 (from 1 areas; max. values)

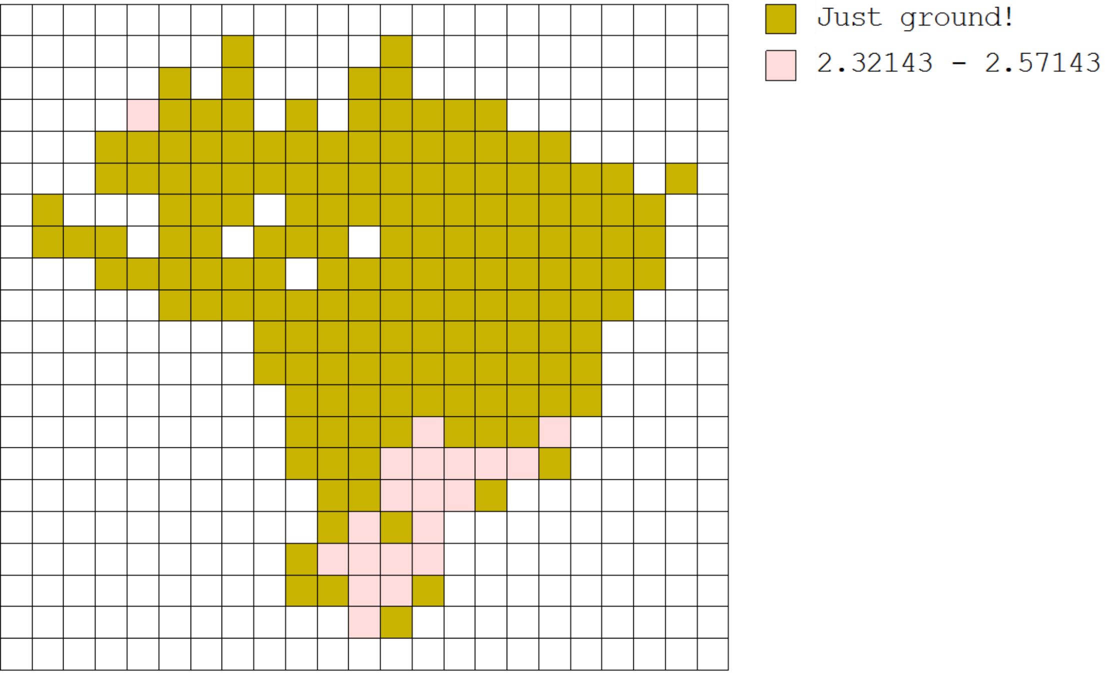

3 species give score:

Acacesia villalobosi (0.766)
Dipoena variabilis (0.696)
Araneus_uniformis (0.859)

Consensus area 45 of 106 (from 2 areas; max. values)

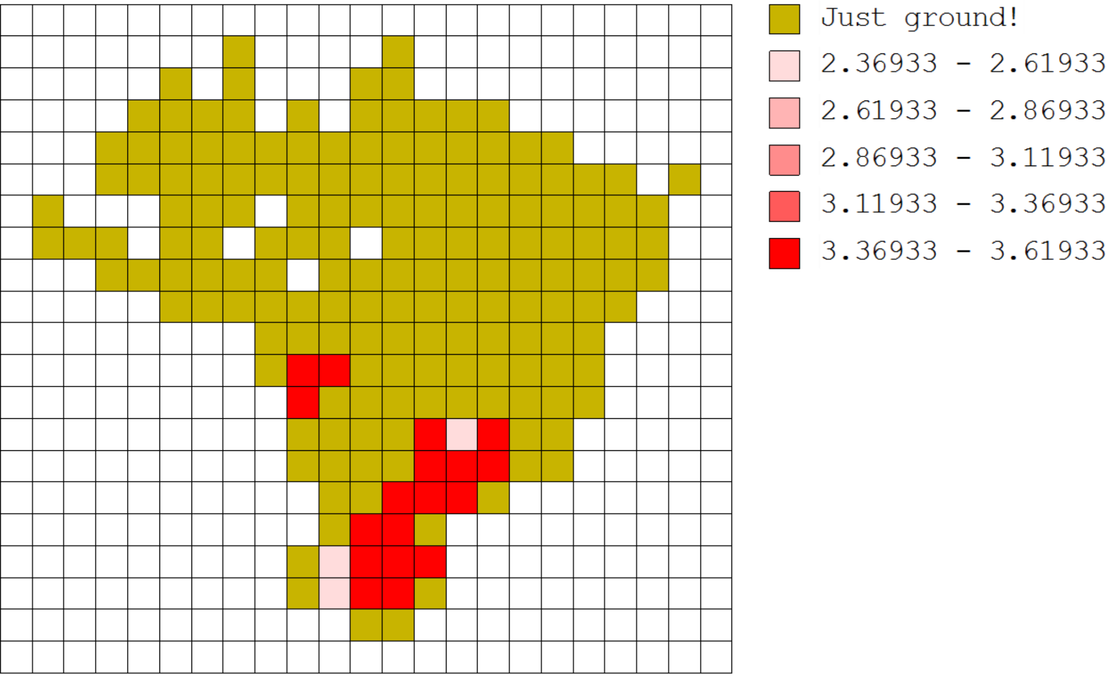

6 species give score:

Alpaida lanei (0.000-0.667)
Jessica fidelis (0.336-0.432)
Trechalea bucculenta (0.538-0.590)
Apopyllus iheringi (0.711-0.722)
Ocrepeira hirsuta (0.581-0.637)
Aysha paiassaquera (0.000-0.694)

Consensus area 46 of 106 (from 3 areas; max. values)

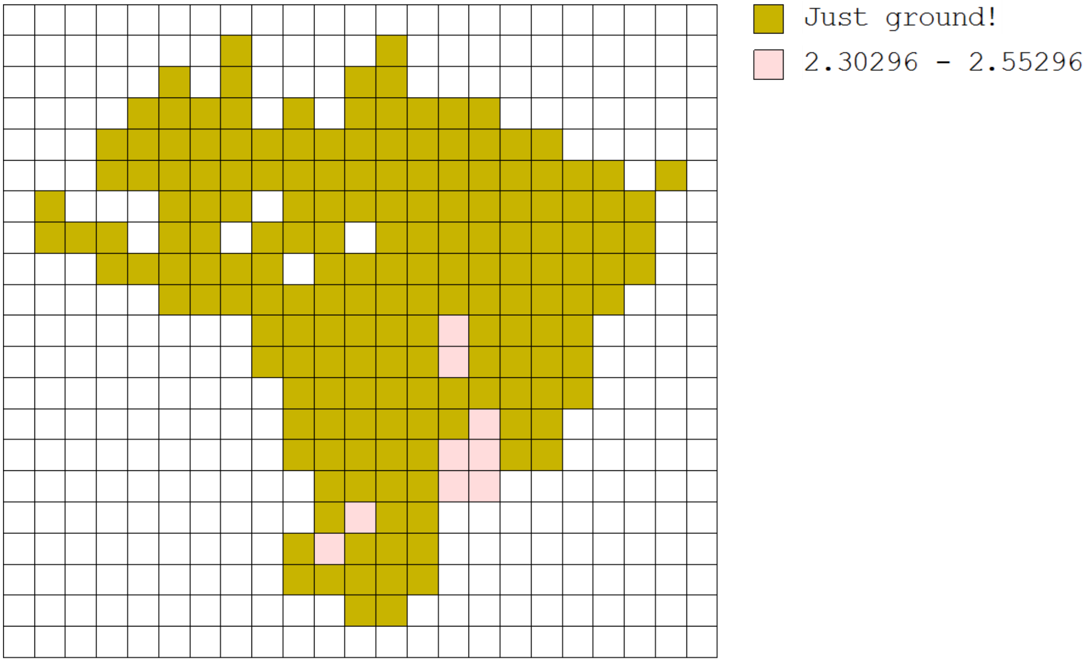

3 species give score:

Mangora ramirezi (0.786-0.833)
Noegus vulpio (0.732-0.776)
Mecynogea_lemniscata (0.698-0.786)

Consensus area 47 of 106 (from 2 areas; max. values)

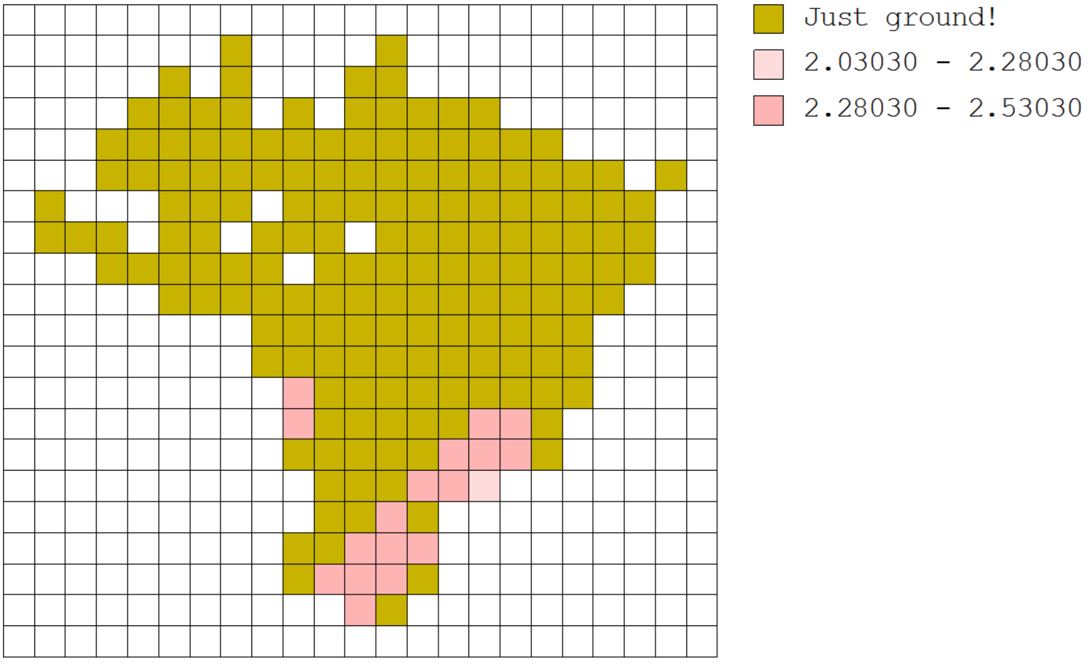

4 species give score:

Araneus corporosus (0.758-0.824)
Tmarus polyandrus (0.545-0.735)
Metazygia viriosa (0.000-0.727)
Trechalea bucculenta (0.000-0.765)

Consensus area 48 of 106 (from 1 areas; max. values)

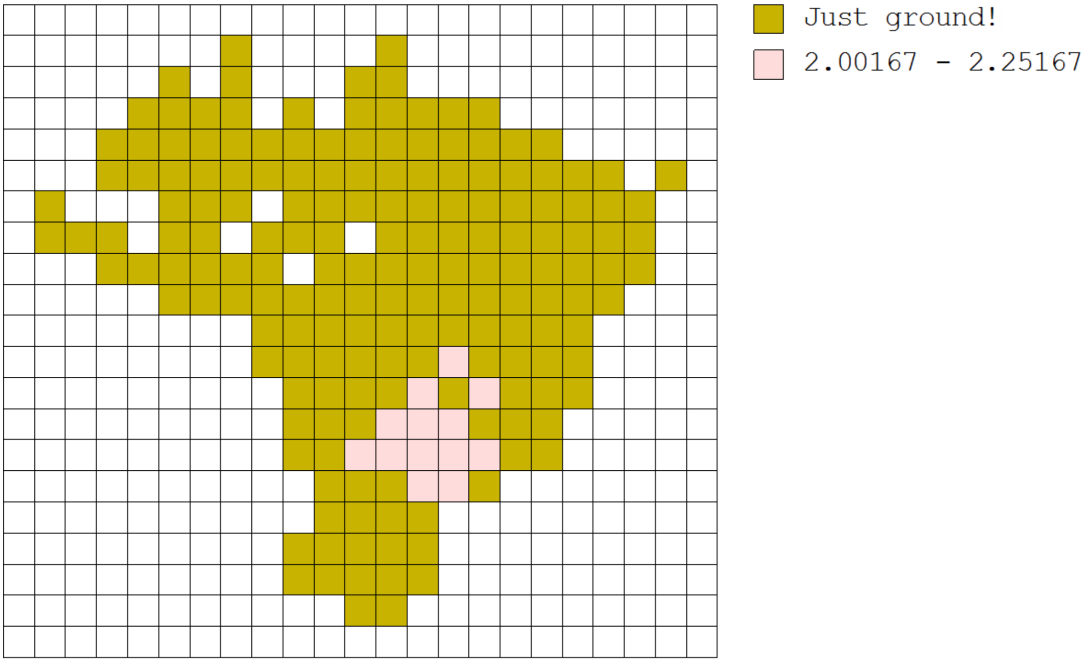

3 species give score:
Abapeba rioclaro(0.597)
Micrathena_swainsoni(0.597)
Vitalius vellutinus(0.808)

Consensus area 49 of 106 (from 3 areas; max. values)

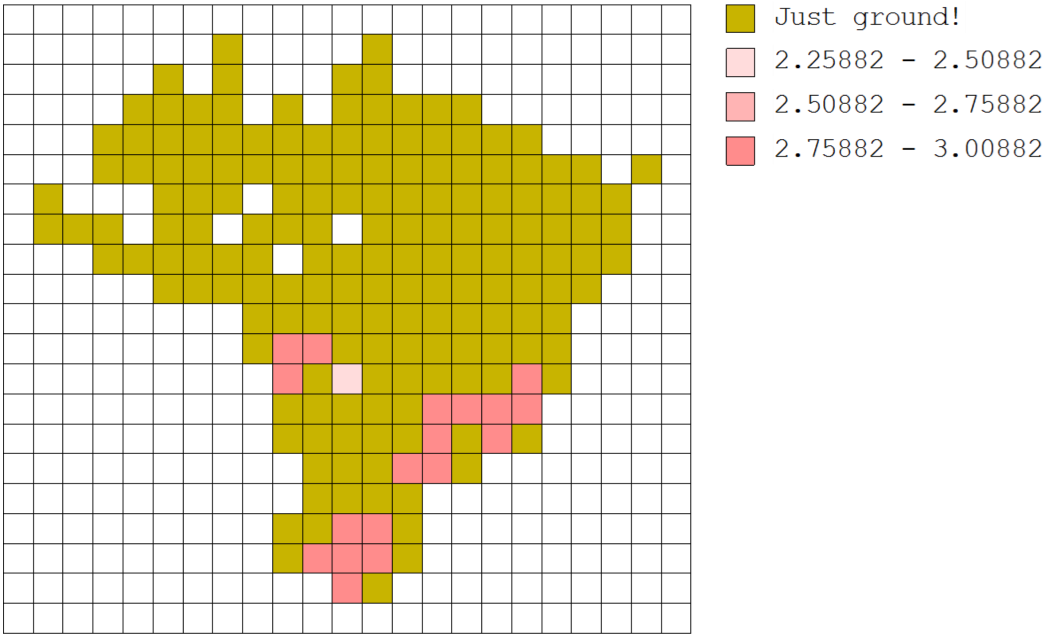

5 species give score:
Chira thysbe(0.000-0.659)
Ocrepeira gnomo(0.806-0.893)
Theridion orgea(0.000-0.633)
Trechalea_bucculenta(0.000-0.681)
Trechona venosa(0.694-0.750)

Consensus area 50 of 106 (from 4 areas; max. values)

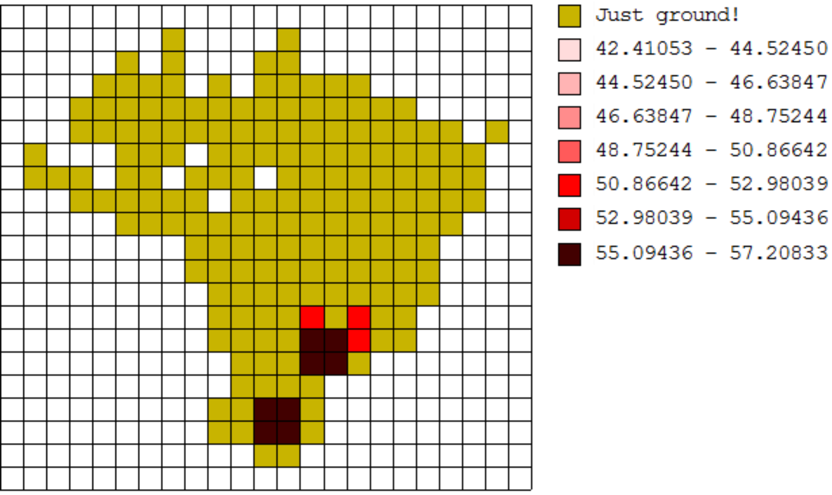

131 species give score:

Acacesia graciosa (0.000-0.688)	Acentroscelus versicolor (0.000-0.409)
Achaearanea triguttata (0.426-0.729)	Actinopus crassipes (0.556-0.626)
Aculepeira vittata (0.606-0.716)	Aillutticus nitens (0.000-0.643)
Alpaida bischoffi (0.611-0.700)	Alpaida citrina (0.000-0.714)
Alpaida nonoai (0.451-0.606)	Alpaida pedro (0.463-0.701)
Alpaida versicolor (0.295-0.545)	Anelosimus nigrescens (0.000-0.394)
Arachosia praesignis (0.000-0.525)	Araneus bandelieri (0.000-0.606)
Araneus orgaos (0.000-0.626)	Araneus sicki (0.000-0.716)
Argiope_ericae (0.000-0.584)	Asaphobelis_physonychus (0.426-0.729)
Ashtabula sexguttata (0.479-0.667)	Asthenoctenus borellii (0.347-0.409)
Aysha ericae (0.000-0.323)	Aysha lisei (0.000-0.584)
Aysha montenegro (0.606-0.716)	Aysha piassaguera (0.611-0.700)
Aysha proseni (0.000-0.584)	Aysha prospera (0.000-0.364)
Aysha rubromaculata (0.000-0.424)	Aysha tertulia (0.000-0.515)
Bertrana rufostriata (0.000-0.455)	Camillina chilensis (0.632-0.714)
Camillina pulchra (0.553-0.649)	Castianeira obscura (0.611-0.700)
Chira distincta (0.000-0.786)	Chrysometa cambara (0.000-0.657)
Chrysometa itaimba (0.519-0.711)	Craspedisia cornuta (0.000-0.591)
Cryptachaea passiva (0.000-0.405)	Ctenus longipes (0.584-0.667)
Ctenus tarsalis (0.556-0.626)	Cyclosa espumoso (0.000-0.468)
Dipoena ira (0.000-0.500)	Dipoena taeniatipes (0.458-0.547)
Episinus cognatus (0.000-0.649)	Episinus teresopolis (0.000-0.626)
Euophrys sutrix (0.611-0.700)	Eustala clavispina (0.556-0.800)
Eustala photographica (0.000-0.458)	Eustala ulecebrosa (0.611-0.700)
Faiditus sicki (0.000-0.626)	Frigga quintensis (0.000-0.606)
Gelanor zonatus (0.000-0.625)	Helvetia albovittata (0.000-0.649)
Hetschkia gracilis (0.426-0.583)	Ianduba varia (0.000-0.379)
Idiops camelus (0.000-0.606)	Isoctenus ordinario (0.000-0.424)
Kaira conica (0.611-0.700)	Larinia montecarlo (0.426-0.606)
Larinia t-notata (0.237-0.566)	Larinia tucuman (0.611-0.700)
Lycosa_paranensis (0.000-0.611)	Lygarina_silvicola (0.667-0.800)
Lyssomanes tristis (0.000-0.485)	Mangora strenua (0.000-0.258)
Mangora v-signata (0.000-0.714)	Mastophora carpogaster (0.000-0.364)
Micrathena guanabara (0.000-0.688)	Micrathena reali (0.000-0.700)
Mopiopia comatula (0.626-0.714)	Nesticus brasiliensis (0.000-0.458)
Noegus australis (0.455-0.632)	Nops meridionalis (0.000-0.688)
Ocrepeira galianoae (0.126-0.455)	Ocrepeira malleri (0.000-0.389)
Odo obscurus (0.000-0.625)	Ostearius melanopygius (0.000-0.526)
Otiothops birabeni (0.000-0.643)	Parawixia inopinata (0.556-0.667)
Patrera longipes (0.000-0.530)	Polybetes rubrosignatus (0.571-0.778)
Pseudotyphistes cristatus (0.722-0.900)	Pycnothele singularis (0.611-0.700)
Rudra dagostinae (0.556-0.626)	Sarinda marcosi (0.626-0.714)
Sassacus helenicus (0.000-0.643)	Scolecura parilis (0.722-0.895)
Scytodes maquine (0.000-0.382)	Semiopyla viperina (0.000-0.584)
Sidymella kolpogaster (0.632-0.714)	Sidymella longispina (0.000-0.649)
Sidymella lucida (0.000-0.779)	Sidymella multispinulosa (0.000-0.649)
Sphecozone castanea (0.556-0.626)	Sphecozone personata (0.611-0.700)
Steatoda grossa (0.000-0.500)	Synemosyna aurantiaca (0.000-0.566)
Synstrophius blanci (0.000-0.584)	Tacuna delecta (0.611-0.722)
Tasata variolosa (0.667-0.800)	Tatepeira itu (0.444-0.584)
Tenedos procreator (0.486-0.553)	Theridion biezankoi (0.426-0.583)
Theridion calcynatum (0.000-0.303)	Theridion opolon (0.000-0.632)
Theridion rubrum (0.611-0.700)	Theridion striatum (0.611-0.700)
Theridula_gonygaster (0.611-0.700)	Thiodina_germaini (0.000-0.643)
Thymoites anicus (0.479-0.667)	Thymoites melloleitaoni (0.000-0.611)
Tmarus albolineatus (0.000-0.643)	Tmarus clavipes (0.000-0.714)
Tmarus estyliferus (0.000-0.643)	Tmarus pugnax (0.000-0.649)
Trachelopachys ignacio (0.000-0.643)	Trechaleoides biocellata (0.000-0.394)
Trechaleoides keyserlingi (0.526-0.611)	Triplogyna ignitula (0.000-0.547)
Tutaibo velox (0.000-0.632)	Urozelotes rusticus (0.000-0.545)
Wagneriana eupalaestra (0.667-0.805)	Wagneriana iguape (0.000-0.455)
Wulfila albus (0.000-0.623)	Wulfilopsis tripunctata (0.000-0.379)
Xiruana gracilipes (0.000-0.429)	Xiruana hirsuta (0.000-0.584)
Alpaida caramba (0.000-0.625)	Anelosimus decoloratus (0.000-0.535)
Eustala crista (0.000-0.500)	Exocora_ribeiroi (0.000-0.626)
Lygarina sylvicola (0.667-0.800)	

Consensus area 51 of 106 (from 2 areas; max. values)

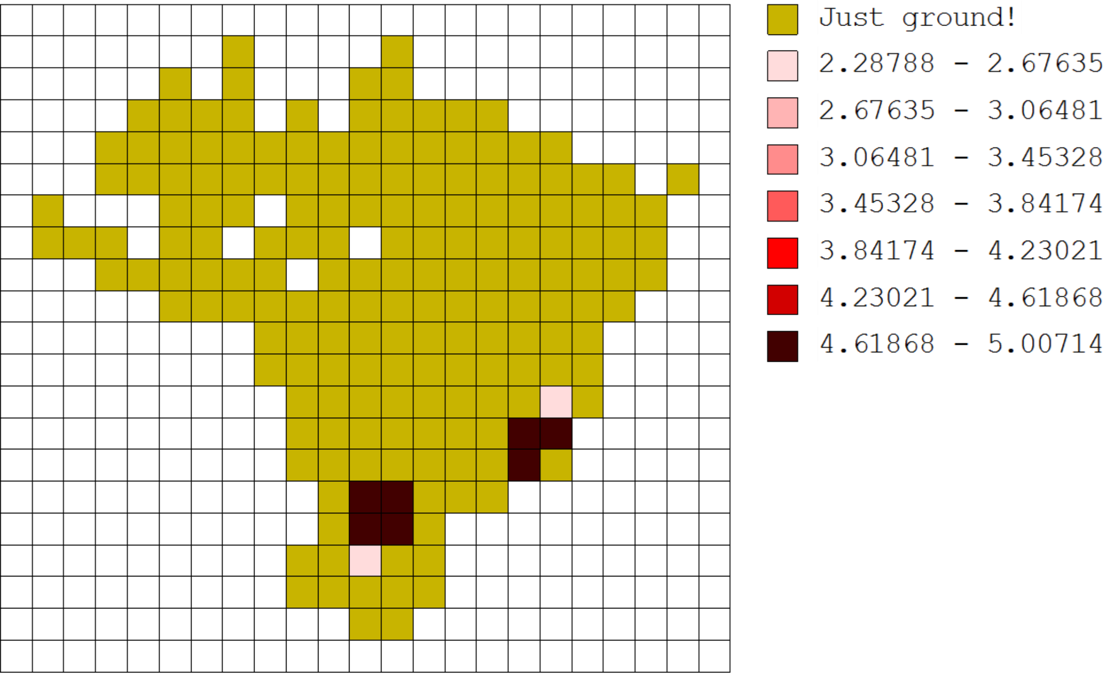

10 species give score:

Araneus pico (0.000-0.643)
Aysha taeniata (0.000-0.643)
Cryptachaea bellula (0.545-0.643)
Ero catharinae (0.000-0.514)
Onocolus infelix (0.000-0.409)
Architis capricorna (0.000-0.386)
Cryptachaea analista (0.643-0.667)
Cyclosa teresa (0.643-0.667)
Iguarima censoria (0.000-0.314)
Toca samba (0.000-0.579)

Consensus area 52 of 106 (from 2 areas; max. values)

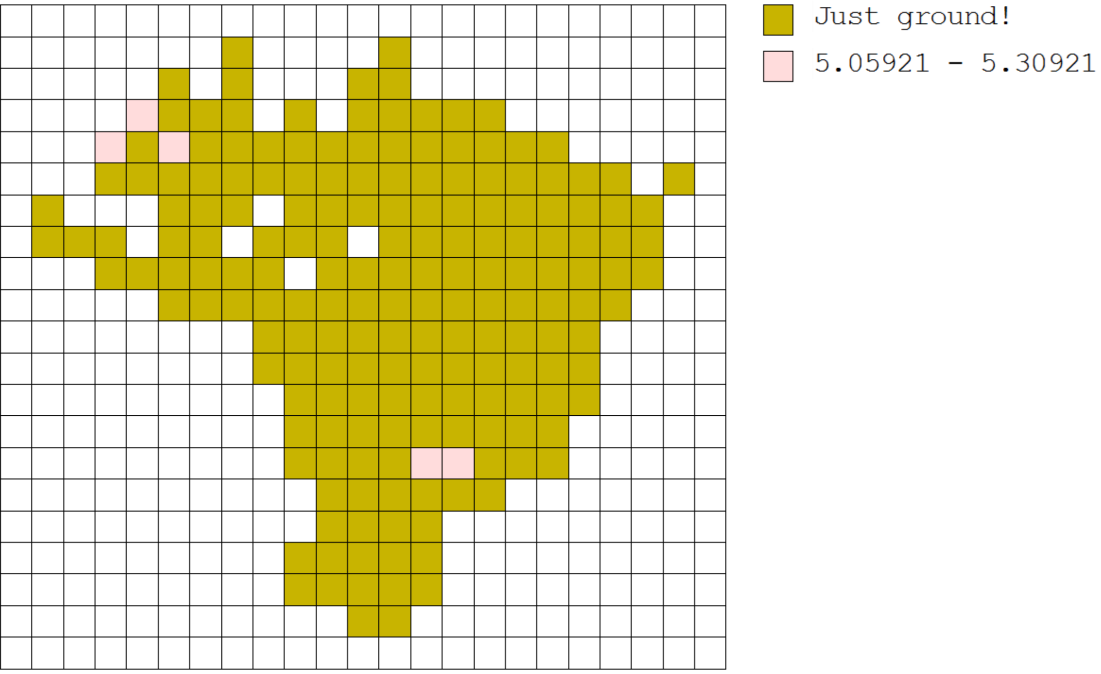

8 species give score:

Acentroscelus_albipes (0.875)
Helvetia_semialba (0.750)
Psecas_chrysogrammus (0.000-0.671)
Semnolius_chrysotrichus (0.750)
Coryphasia_fasciiventris (0.750)
Hypognatha_alho (0.469-0.513)
Psecas_zonatus (0.000-0.750)
Theridion_nigriceps (0.750)

Consensus area 53 of 106 (from 3 areas; max. values)

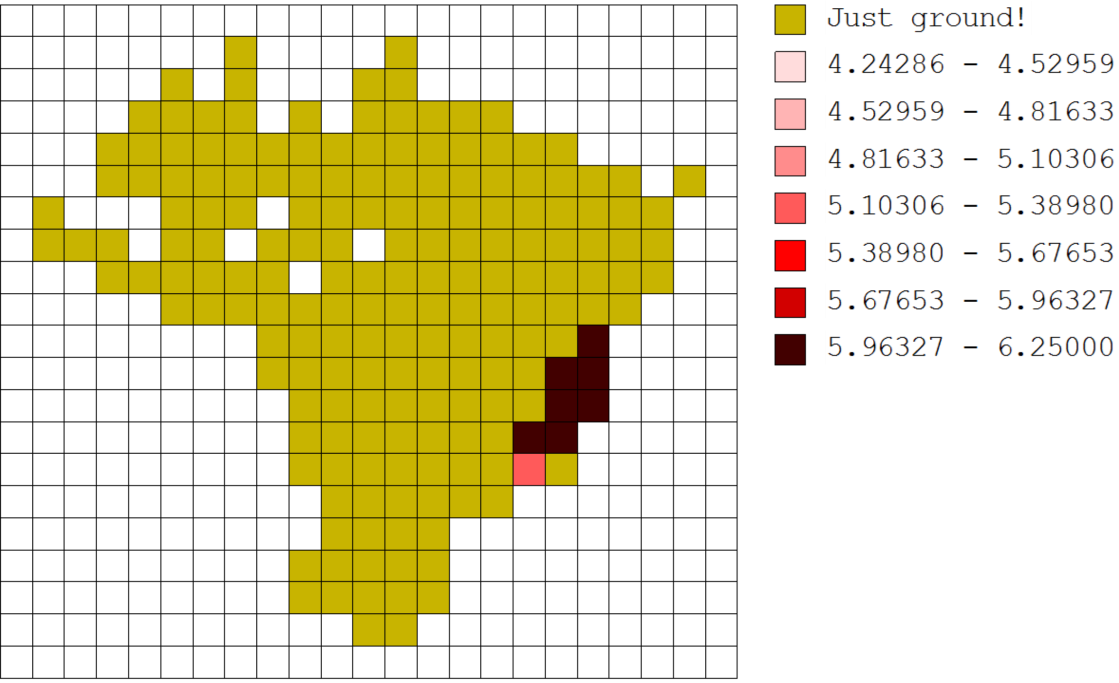

11 species give score:

Avicularia sooretama (0.000-0.595)
Caayguara juati (0.643-0.700)
Linyphia obscurella (0.643-0.700)
Scytodes guapiassu (0.536-0.750)
Vinnius subfasciatus (0.400-0.655)
Litoporus iguassuensis (0.524-0.643)
Caayguara ajuba (0.000-0.714)
Eutichurus itamaraju (0.000-0.667)
Phoneutria pertyi (0.000-0.714)
Scytodes una (0.000-0.667)
Cryptachaea_brescoviti (0.000-0.700)

Consensus area 54 of 106 (from 1 areas; max. values)

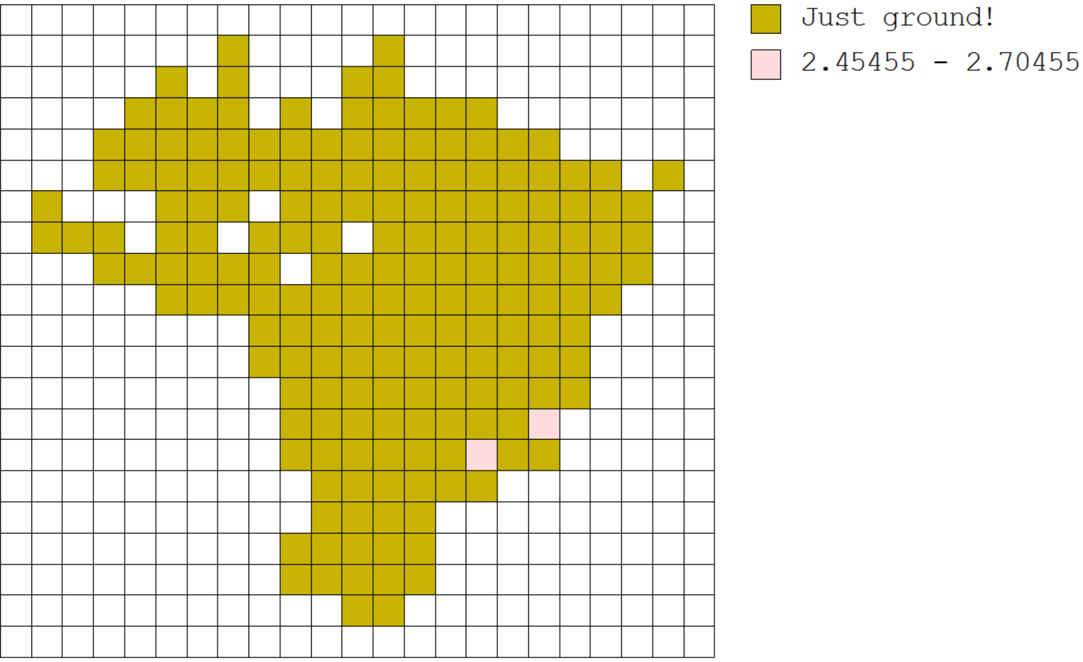

3 species give score:

Carapoia ubatuba (1.000)
Micrathena ruschii (0.455)
Mangora_itatiaia (1.000)

Consensus area 55 of 106 (from 2 areas; max. values)

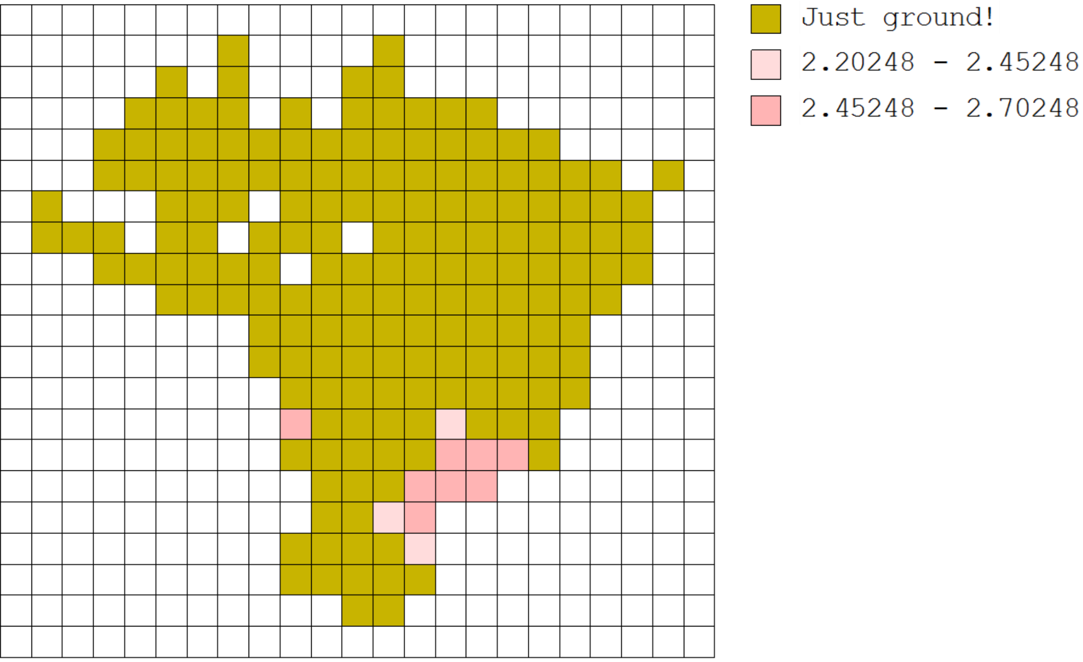

4 species give score:
Metazygia viriosa (0.662-0.727)
Philoponella fasciata (0.717-0.773)
Scytodes bonito (0.000-0.688)
Scytodes brignolii (0.621-0.702)

Consensus area 56 of 106 (from 6 areas; max. values)

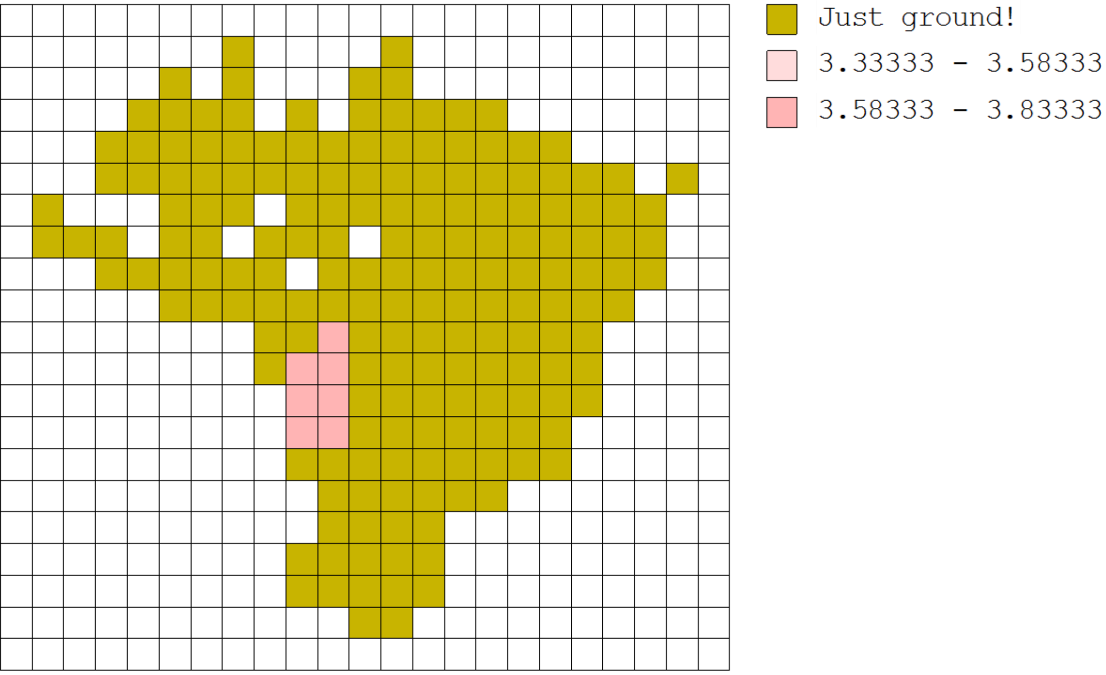

8 species give score:
Anelosimus pantanal (0.700-0.833)
Araneus cuiaba (0.700-0.833)
Escaphiella pocone (0.000-0.635)
Metaphidippus tropicus (0.000-0.700)
Paradossenus corumba (0.700-0.833)
Scytodes akytaba (0.000-0.833)
Micrathena bandeirante (0.000-0.327)
Scytodes tuyuca (0.000-0.750)

Consensus area 57 of 106 (from 3 areas; max. values)

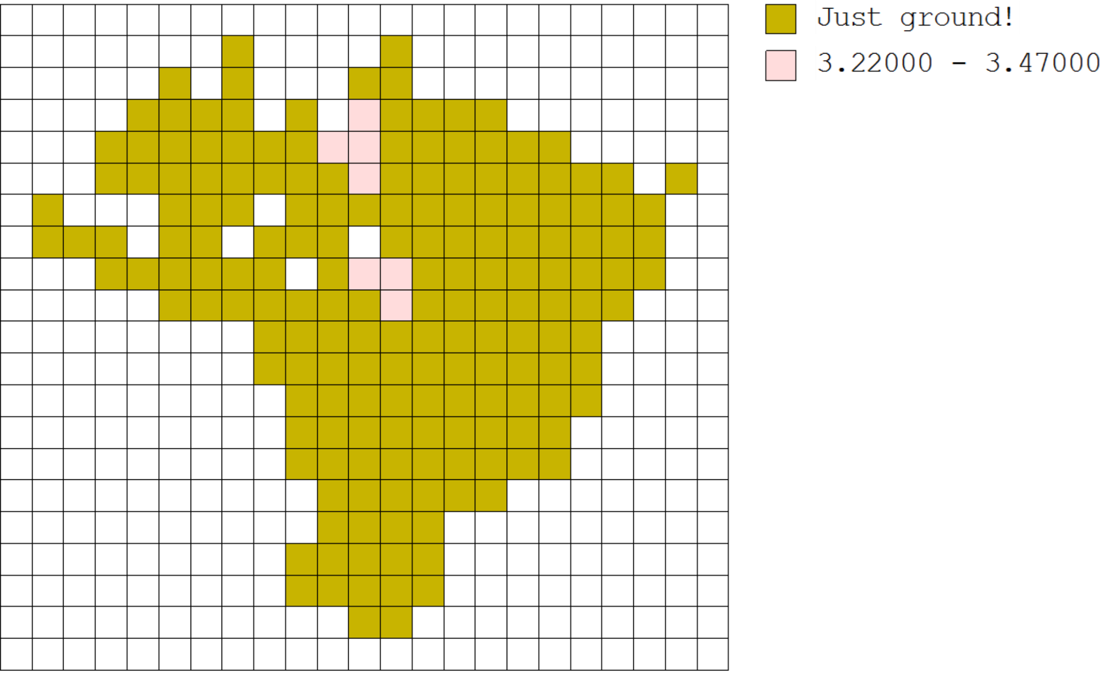

5 species give score:

Ctenus similis (0.700)
Lyssomanes santarem (0.700)
Scaphiella penna (0.560-0.573)
Diplura sanguinea (0.560-0.573)
Psecas_sumptuosus (0.700)

Consensus area 58 of 106 (from 5 areas; max. values)

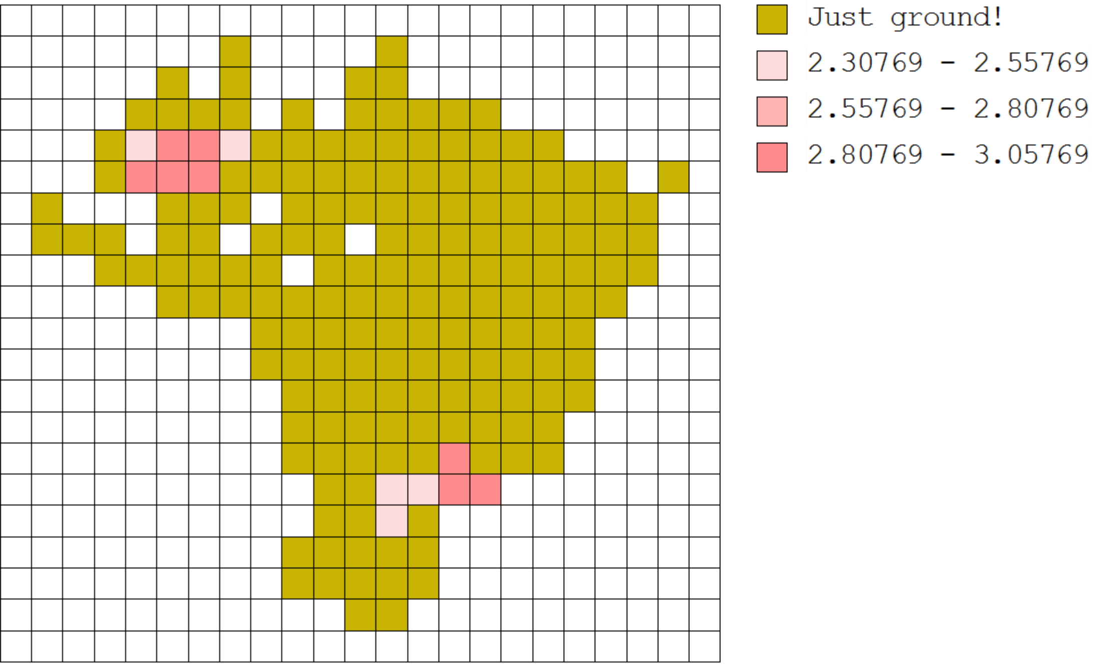

50 species give score:

Alpaida vanzolinii (0.000-0.643)	Amazonepeira herrera (0.000-0.900)
Anapis castilla (0.000-0.700)	Anyphaenoides pacifica (0.000-0.700)
Centroctenus acara (0.000-0.700)	Centroctenus miriuma (0.000-0.613)
Coleosoma acutiventer (0.000-0.514)	Corinna recurva (0.000-0.700)
Ctenus manauara (0.000-0.700)	Cyclosa vieirae (0.000-0.613)
Dipoena tiro (0.000-0.831)	Dolichognatha ducke (0.000-0.700)
Dyrines ducke (0.000-0.700)	Encyosaccus sexmaculatus (0.000-0.700)
Ephebopus uatuman (0.000-0.700)	Epicratinus amazonicus (0.000-0.800)
Faiditus altus (0.000-0.875)	Gelanor heraldicus (0.000-0.700)
Hingstepeira dimona (0.000-0.800)	Hingstepeira folisecens (0.000-0.800)
Hypaeus miles (0.000-0.700)	Hypaeus triplagiatus (0.000-0.700)
Hypognatha colosso (0.000-0.700)	Litoporus dimona (0.000-0.800)
Lygromma gasnieri (0.000-0.800)	Lygromma huberti (0.000-0.700)
Lyssomanes nigropictus (0.000-0.677)	Mangora mamiraua (0.000-0.700)
Mangora sumauma (0.000-0.900)	Metazygia uma (0.000-0.700)
Micrathena coca (0.000-0.787)	Micrathena embira (0.000-0.800)
Myrmecotypus olympus (0.000-0.700)	Noegus fuscimanus (0.000-0.735)
Ochyrocera hamadryas (0.000-0.700)	Pachomius sextus (0.000-0.700)
Parachemmis manauara (0.000-0.700)	Peucetia macroglossa (0.000-0.675)
Psecas chrysogrammus (0.000-0.714)	Rhoicinus urucu (0.000-0.700)
Scytodes balbina (0.000-0.700)	Scytodes martiusi (0.000-0.700)
Selenops lavillai (0.000-0.700)	Stethorrhagus lupulus (0.000-0.700)
Synotaxus waiwai (0.000-0.700)	Zimiromus kleini (0.000-0.700)
Zimiromus syenus (0.000-0.700)	Breda nanica (0.000-0.700)
Micrathena abrahami (0.000-0.700)	Neoxyphinus petrogoblin (0.000-0.800)

Consensus area 59 of 106 (from 4 areas; max. values)

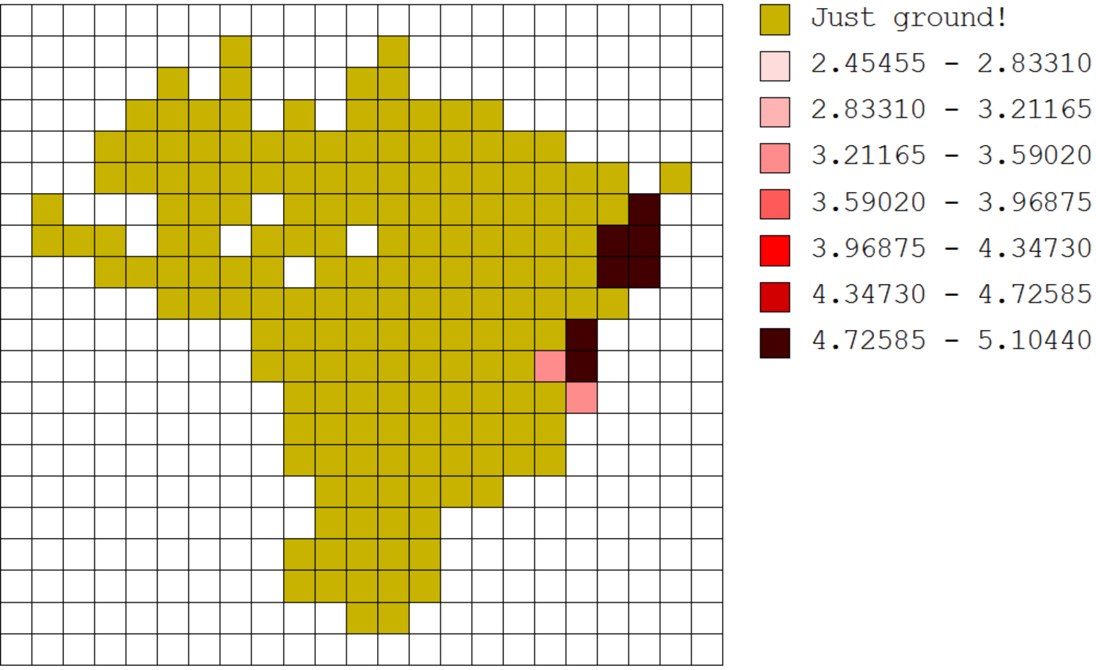

51 species give score:

Amazonepeira herrera(0.000-0.900)
Anyphaenoides pacifica(0.000-0.700)
Centroctenus miriuma(0.000-0.613)
Corinna rubripes(0.000-0.643)
Cyclosa vieirae(0.000-0.613)
Dyrines ducke(0.000-0.700)
Ephebopus uatuman(0.000-0.700)
Gelanor heraldicus(0.000-0.700)
Hingstepeira dimona(0.000-0.800)
Hypaeus miles(0.000-0.700)
Hypognatha colosso(0.000-0.700)
Litoporus dimona(0.000-0.800)
Lygromma huberti(0.000-0.700)
Mangora sumauma(0.000-0.900)
Metazygia uma(0.000-0.700)
Micrathena embira(0.000-0.800)
Nesticella murici(0.000-0.786)
Pachomius sextus(0.000-0.700)
Peucetia macroglossa(0.000-0.675)
Rhoicinus urucu(0.000-0.700)
Scytodes martiusi(0.000-0.700)
Stethorrhagus lupulus(0.000-0.700)
Zimiromus kleini(0.000-0.700)
Breda nanica(0.000-0.700)
Neopisinus recifensis(0.000-0.714)
Typhochlaena paschoali(0.000-0.667)

Anapis castilla(0.000-0.700)
Centroctenus acara(0.000-0.700)
Corinna recurva(0.000-0.700)
Ctenus manauara(0.000-0.700)
Dolichognatha ducke(0.000-0.700)
Encyosaccus sexmaculatus(0.000-0.700)
Epicratinus amazonicus(0.000-0.800)
Guyruita atlantica(0.000-0.786)
Hingstepeira folisecens(0.000-0.800)
Hypaeus triplagiatus(0.000-0.700)
Jessica sergipana(0.000-0.688)
Lygromma gasnieri(0.000-0.800)
Mangora mamiraua(0.000-0.700)
Matta angelomachadoi(0.000-0.643)
Micrathena coca(0.000-0.787)
Myrmecotypus olympus(0.000-0.700)
Ochyrocera hamadryas(0.000-0.700)
Parachemmis_manauara(0.000-0.700)
Proshapalopus_multicuspidatus(0.000-1.000)
Scytodes balbina(0.000-0.700)
Selenops lavillai(0.000-0.700)
Synotaxus waiwai(0.000-0.700)
Zimiromus syenus(0.000-0.700)
Micrathena abrahami(0.000-0.700)
Neoxyphinus_petrogoblin(0.000-0.800)

Consensus area 60 of 106 (from 3 areas; max. values)

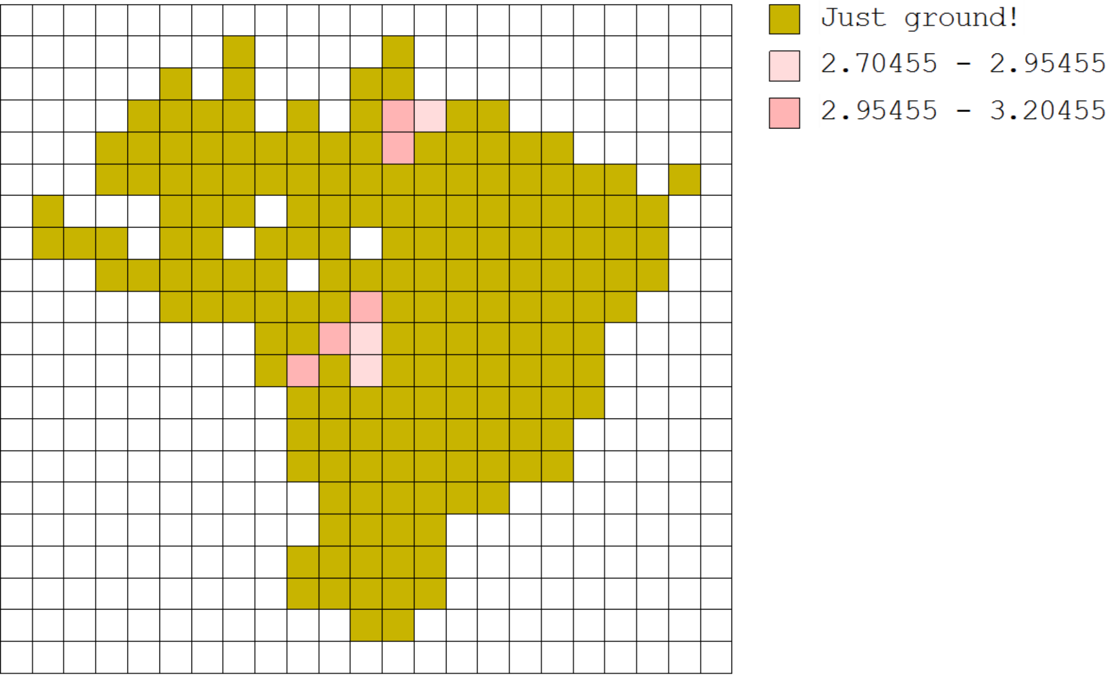

6 species give score:

Alpaida chapada (0.667-0.750)
Alpaida iquitos (0.750-0.875)
Mangora isabel (0.000-0.682)
Marma nigritarsis (0.000-0.675)
Paradossenus minimus (0.000-0.679)
Titidius caninde (0.000-0.679)

Consensus area 61 of 106 (from 2 areas; max. values)

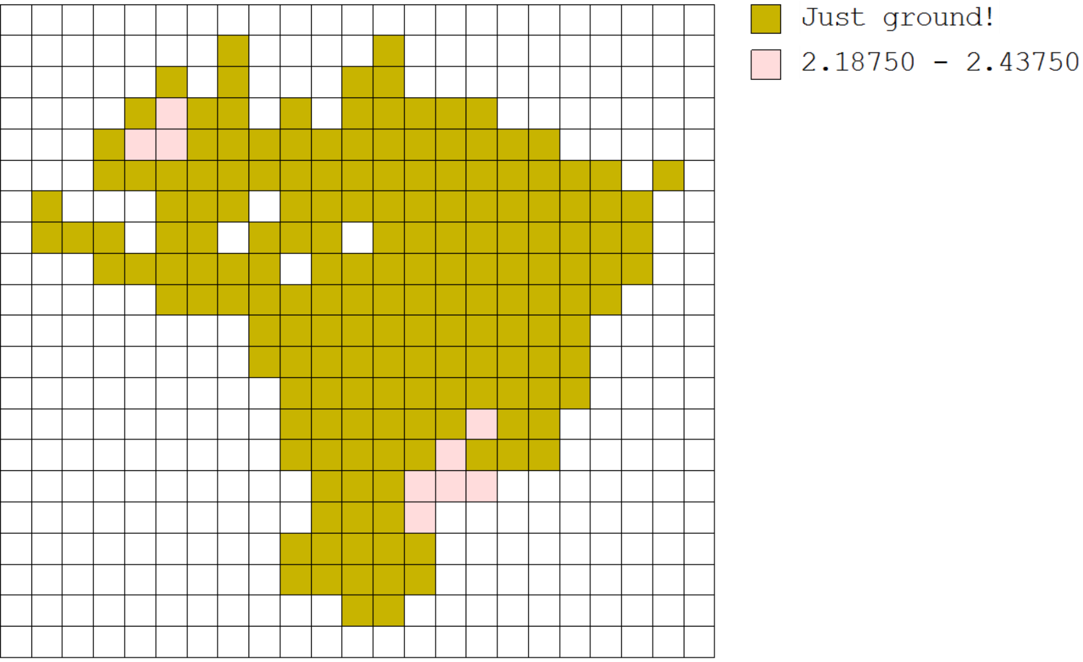

4 species give score:

Zozis geniculata (0.588-0.688)
Alpaida vanzolinii (0.000-0.667)
Mangora enseada (0.431-0.813)
Psecas chrysogrammus (0.688-0.750)

Consensus area 62 of 106 (from 1 areas; max. values)

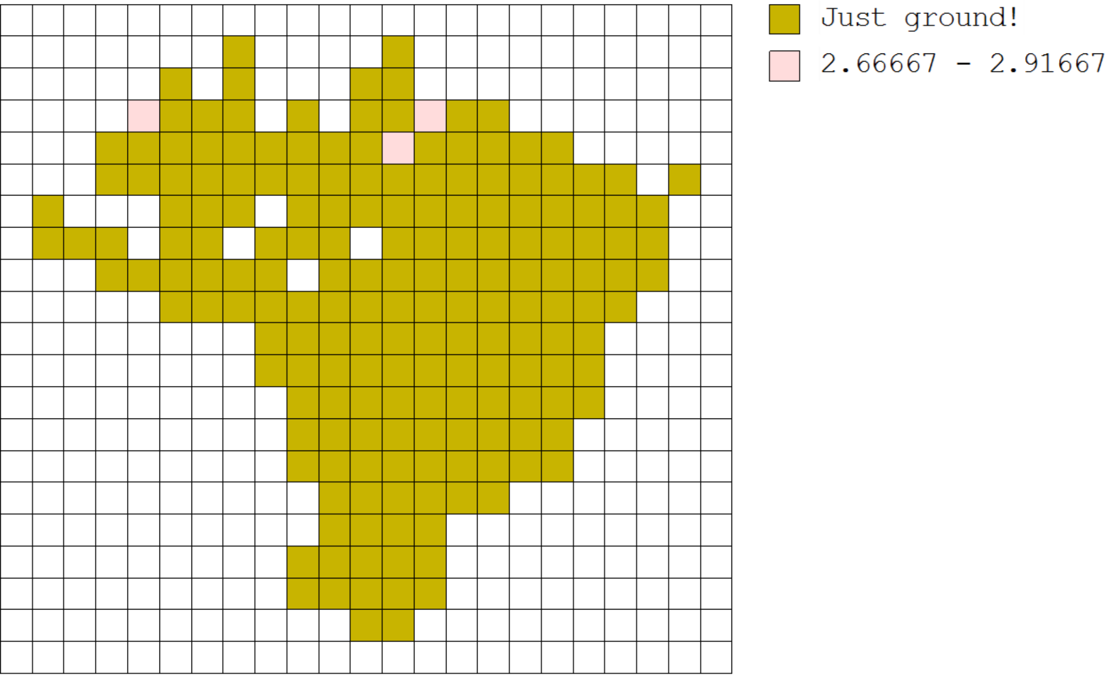

3 species give score:

Alpaida_deborae(0.833)
Scopocira_tenella(1.000)
Stephanopoides sexmaculata(0.833)

Consensus area 63 of 106 (from 2 areas; max. values)

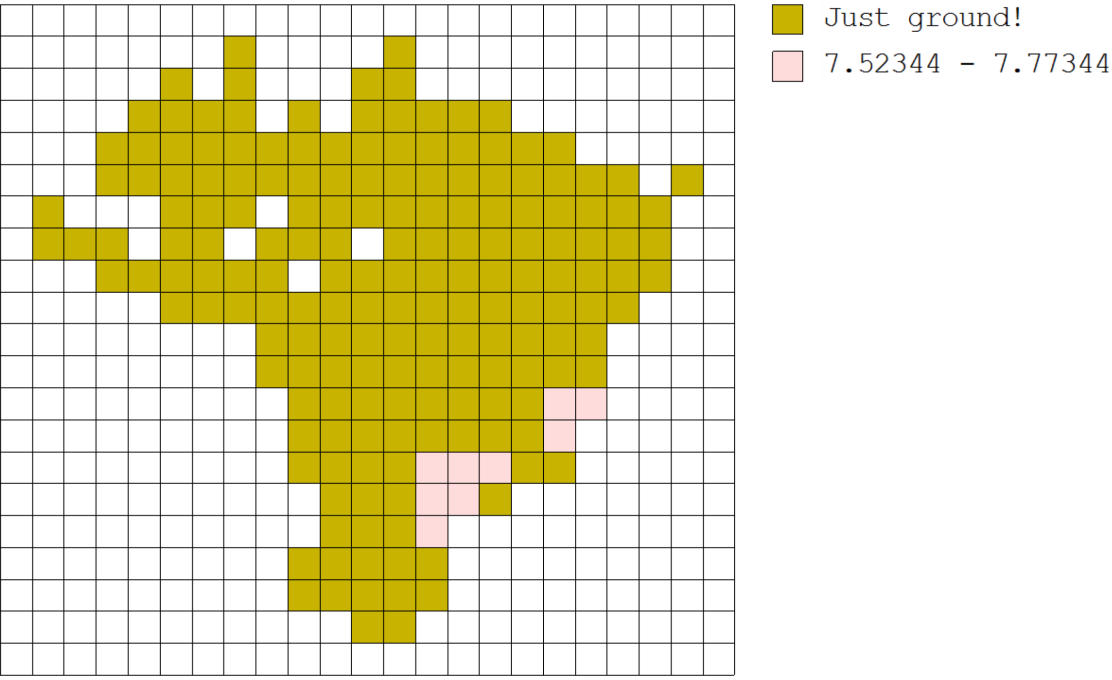

14 species give score:

Allocosa brasiliensis(0.000-0.625)
Architis colombo(0.602-0.611)
Bromelina oliola(0.750)
Buckupiella imperatriz(0.000-0.778)
Enna caparao(0.547-0.556)
Mastophora piras(0.688)
Micrathena annulata(0.688)
Neotama cunhabebe(0.688)
Osoriella rubella(0.563-0.583)
Paratrechalea saopaulo(0.000-0.602)
Wagneriana dimastophora(0.000-0.535)
Wulfilopsis leopoldina(0.547-0.556)
Micrathena ruschii(0.516-0.535)
Neoxyphinus keyserlingi(0.711-0.722)

Consensus area 64 of 106 (from 3 areas; max. values)

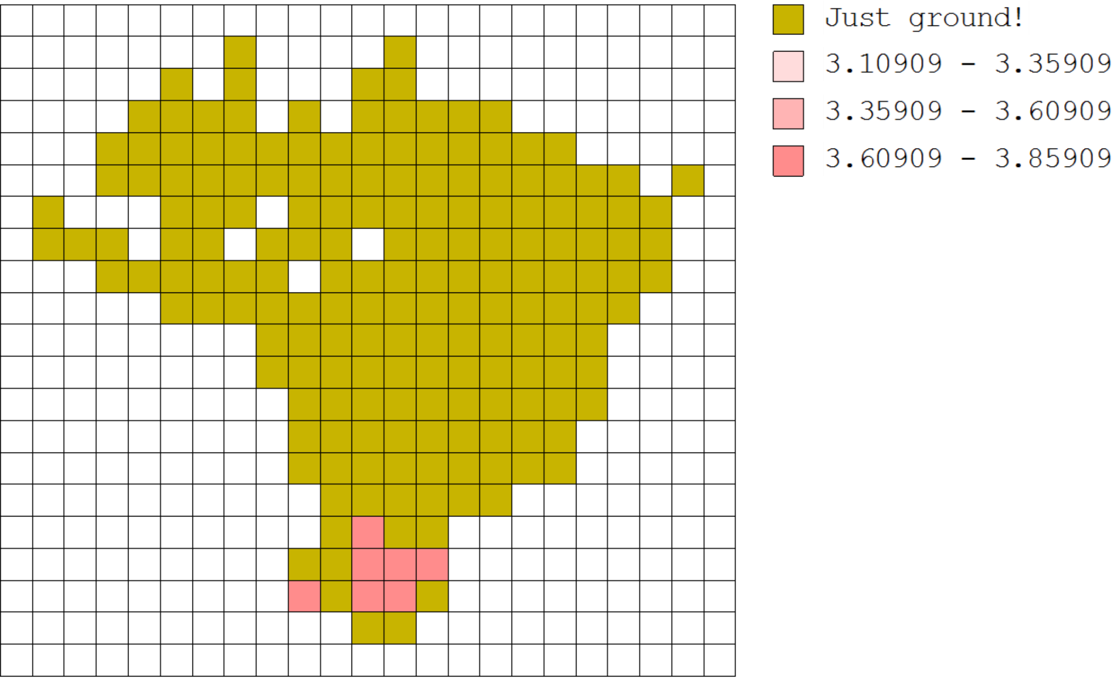

5 species give score:

Aphirape uncifera(0.818-1.000)
Guaraniella bracata(0.509-0.857)
Stenoterommata palmar(0.445-0.600)
Euryopis camis(0.550-0.774)
Meriola_hyltonae(0.643-0.700)

Consensus area 65 of 106 (from 3 areas; max. values)

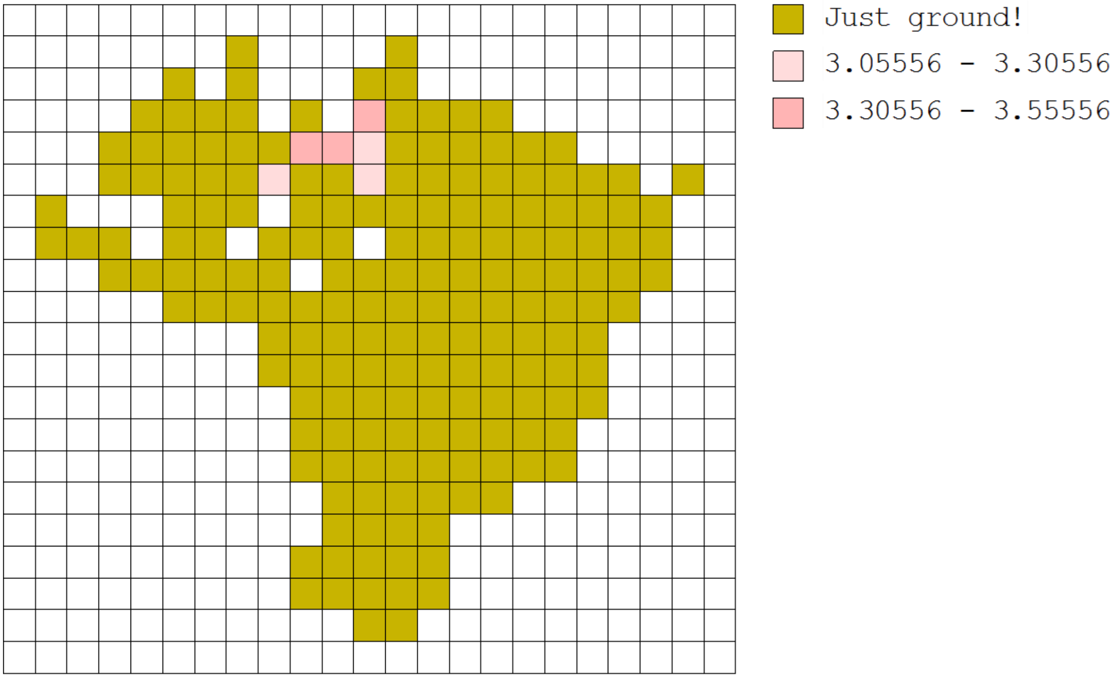

5 species give score:

Ctenus nigritus(0.556-0.818)
Drymusa colligata(0.700-0.833)
Munduruku bicoloratum(0.700-0.833)
Ctenus serratipes(0.700-0.833)
Scytodes_altamira(0.000-0.400)

Consensus area 66 of 106 (from 3 areas; max. values)

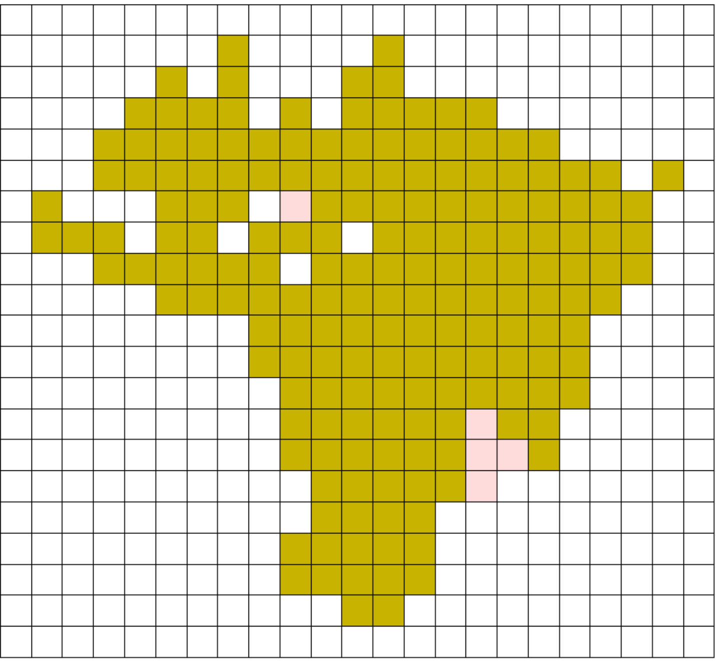

Just ground!

2.38095 - 2.63095

3 species give score:
Cryptachaea rioensis(0.714-0.735)
Myrmecium_vertebratum(0.833)
Wulfilopsis frenata(0.833)

Consensus area 67 of 106 (from 3 areas; max. values)

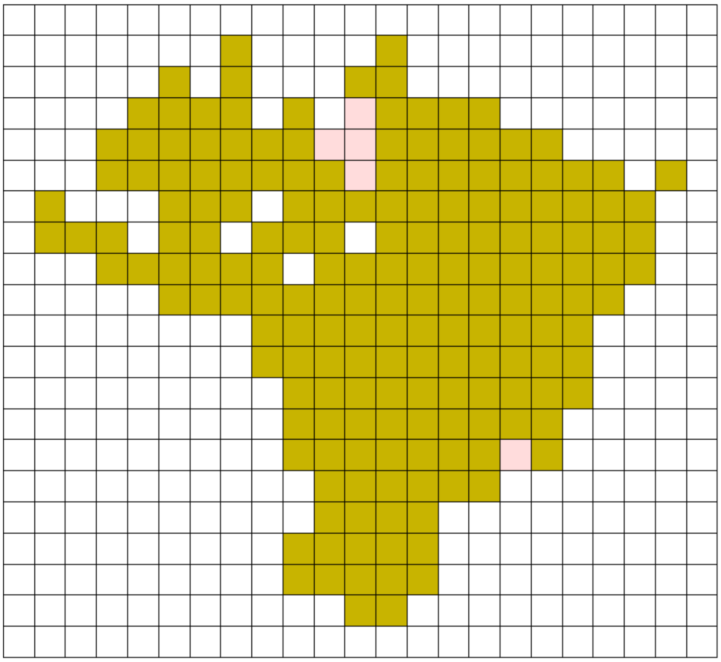

Just ground!

2.29167 - 2.54167

3 species give score:
Cotinusa horatia(0.833)
Mesabolivar_cambridgei(0.625-0.867)
Pippuhana unicolor(0.833)

Consensus area 68 of 106 (from 1 areas; max. values)

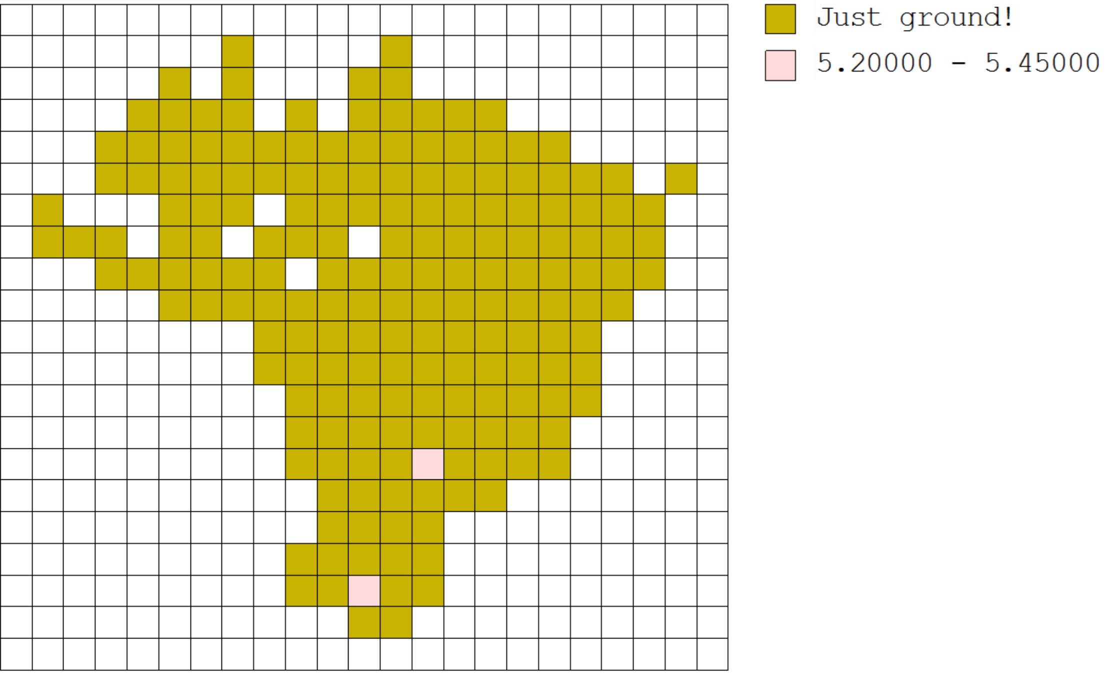

6 species give score:

Chira distincta (0.733)
Thiodina germaini (1.000)
Tmarus estyliferus (1.000)
Semiopyla viperina (0.867)
Tmarus clavipes (0.867)
Tmarus pugnax (0.733)

Consensus area 69 of 106 (from 3 areas; max. values)

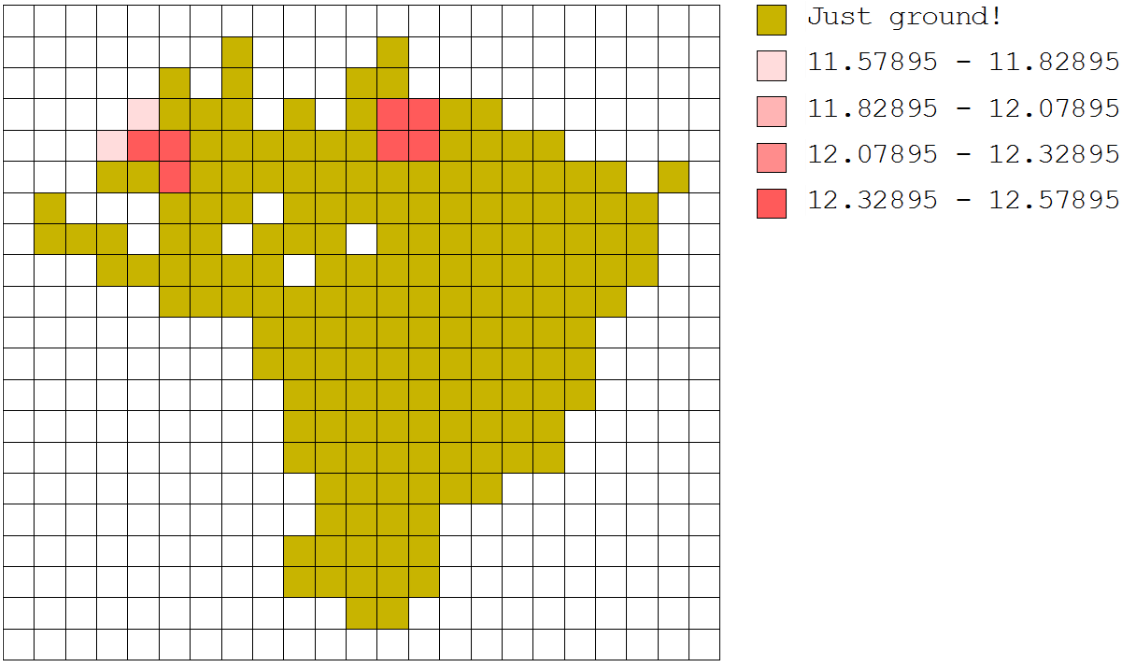

24 species give score:

Alpaida_deborae (0.000-0.667)
Amycus_spectabilis (0.643-0.667)
Bertrana_elinguis (0.500-0.786)
Chirothecia_amazonica (0.575-0.583)
Faiditus_ululans (0.000-0.643)
Kalcerrytus_merretti (0.643-0.667)
Martella_pasteuri (0.643-0.667)
Noegus_transversalis (0.000-0.643)
Pseudosparianthis_ravida (0.000-0.643)
Senoculus_ruficapillus (0.000-0.667)
Stephanopoides sexmaculata (0.000-0.667)
Theraphosa_blondi (0.714-0.750)
Alpaida_guto (0.000-0.643)
Bellota_violacea (0.643-0.667)
Cerocida_ducke (0.575-0.714)
Descanso_ventrosus (0.643-0.667)
Freya_dureti (0.643-0.667)
Lyssomanes_velox (0.000-0.667)
Mermessus_conjunctus (0.000-0.643)
Pachomius_dybowskii (0.643-0.667)
Scopocira_tenella (0.000-0.750)
Sparianthis_amazonica (0.583-0.714)
Stethorrhagus_limbatus (0.575-0.583)
Wagneriana_hassleri (0.000-0.714)

Consensus area 70 of 106 (from 1 areas; max. values)

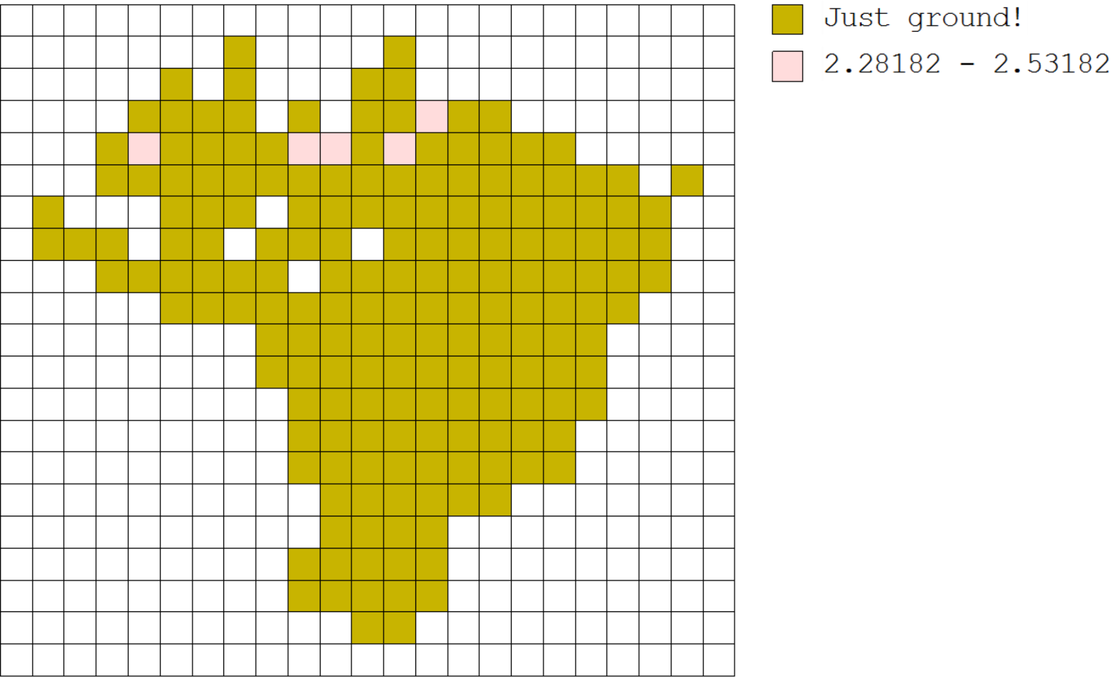

3 species give score:
Ctenus minor(0.655)
Ephebopus_murinus(0.727)
Neoxyphinus barreirosi(0.900)

Consensus area 71 of 106 (from 1 areas; max. values)

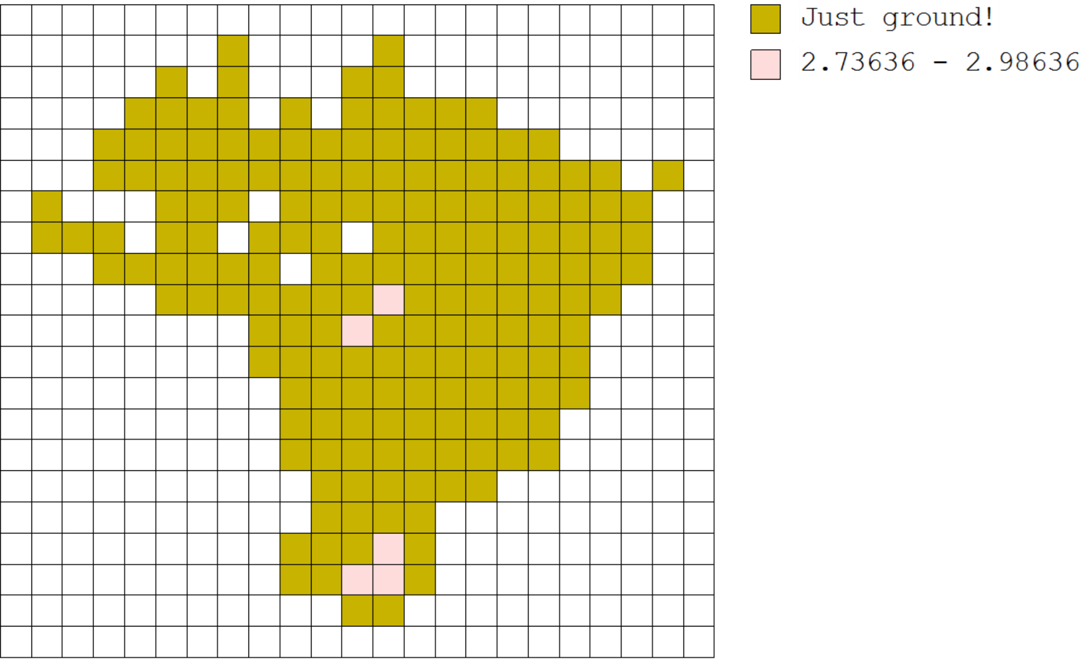

4 species give score:
Cyclosa walckenaeri(0.700)
Kaira cobimcha(0.700)
Oxyopes birabeni(0.636)
Tatepeira tatarendensis(0.700)

Consensus area 72 of 106 (from 1 areas; max. values)

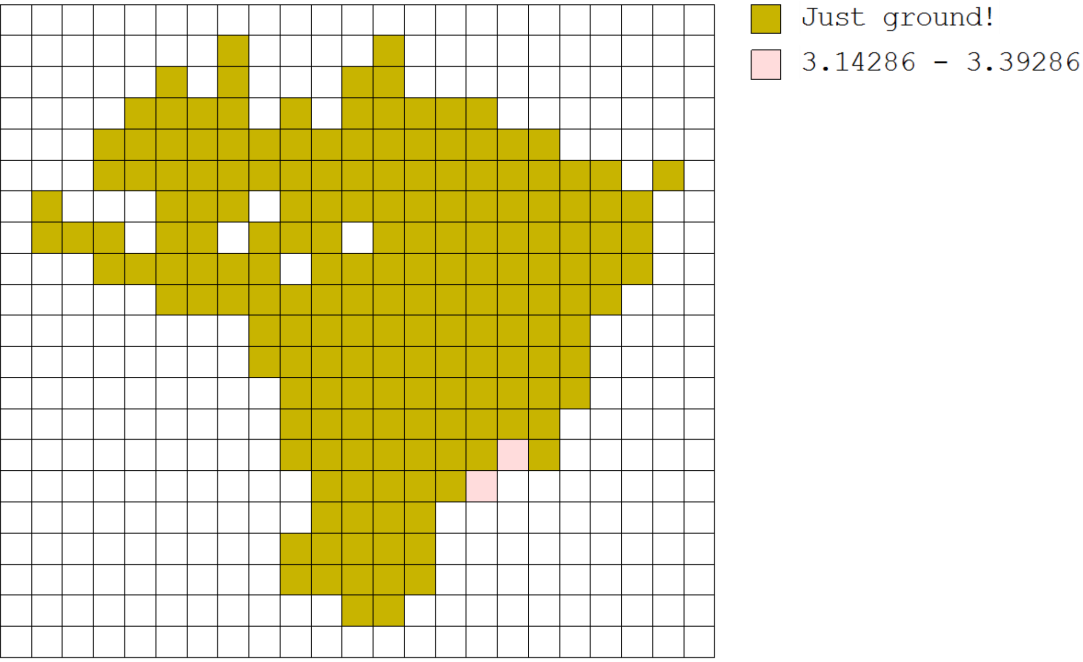

4 species give score:

Alpaida keyserlingi (1.000)
Micrathena ruschii (0.143)
Magulla buecherli (1.000)
Predatoroonops maceliot (1.000)

Consensus area 73 of 106 (from 1 areas; max. values)

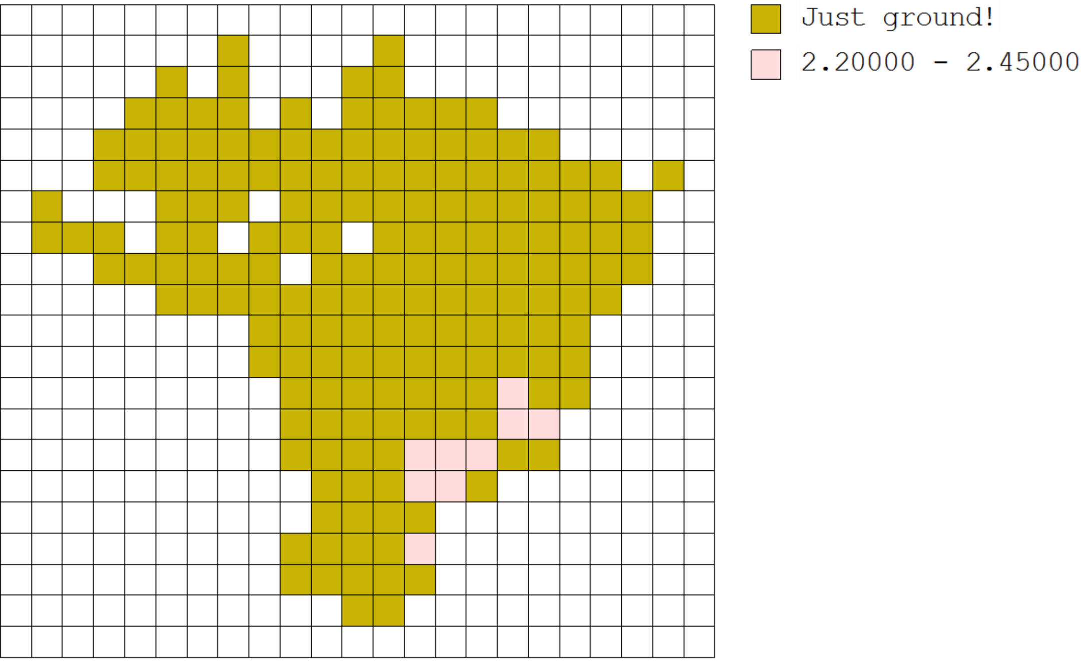

3 species give score:

Buckupiella imperatriz (0.889)
Wagneriana dimastophora (0.778)
Urozelotes_rusticus (0.533)

Consensus area 74 of 106 (from 1 areas; max. values)

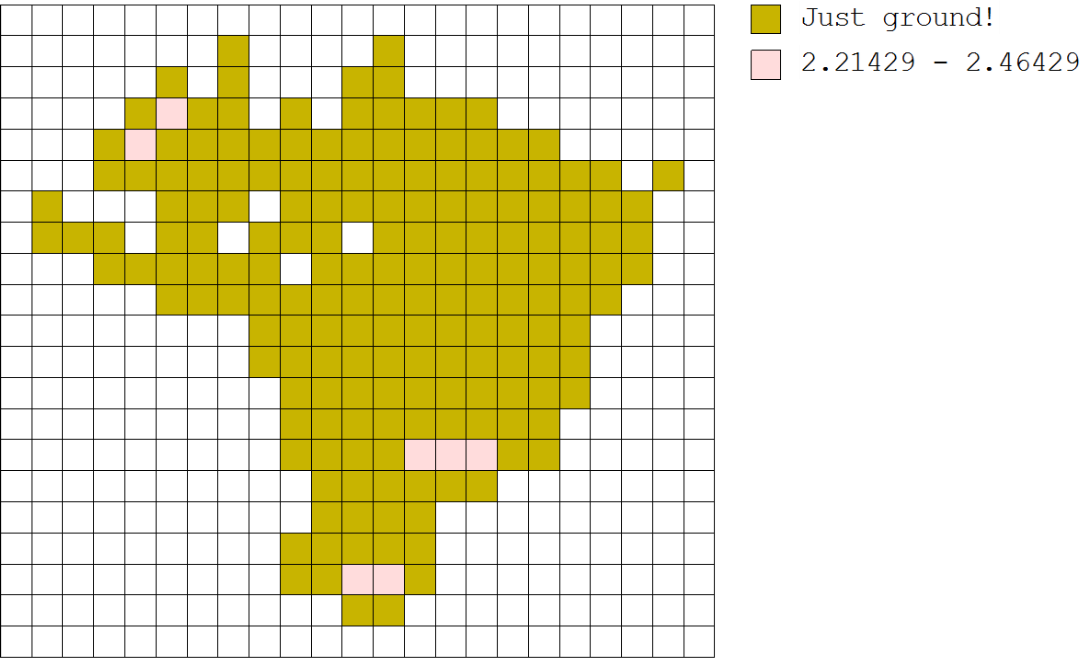

3 species give score:
Cyclosa camargoi(0.673)
Episinus_malachinus(0.612)
Vesicapalpus simplex(0.929)

Consensus area 75 of 106 (from 1 areas; max. values)

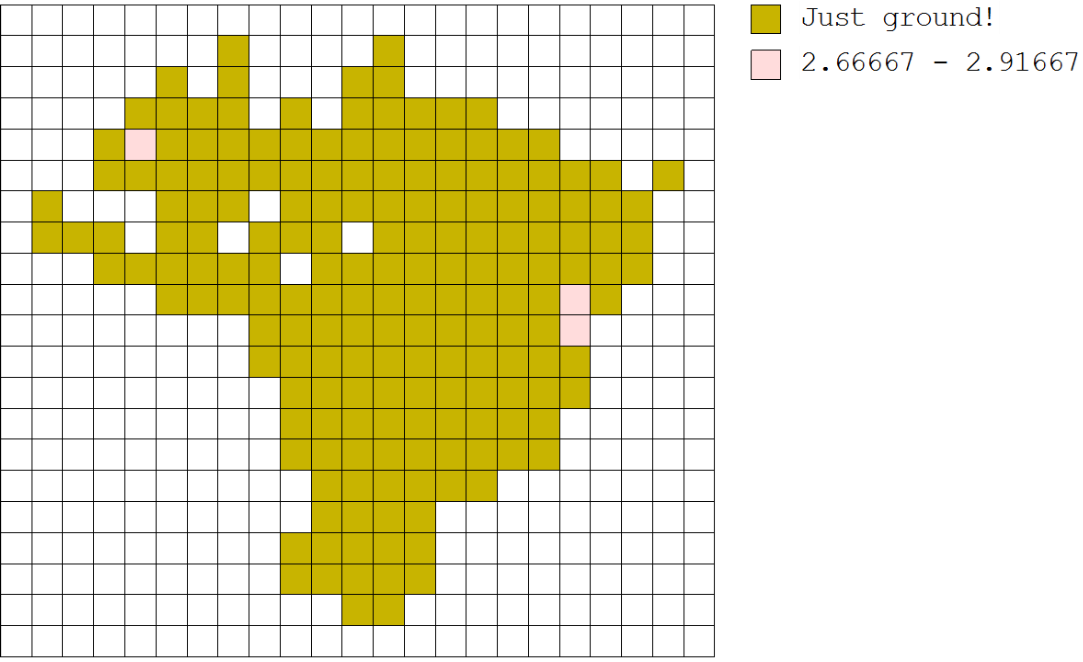

3 species give score:
Epeiroides bahiensis(1.000)
Selenops_kikay(0.833)
Myrmecium velutinum(0.833)

Consensus area 76 of 106 (from 1 areas; max. values)

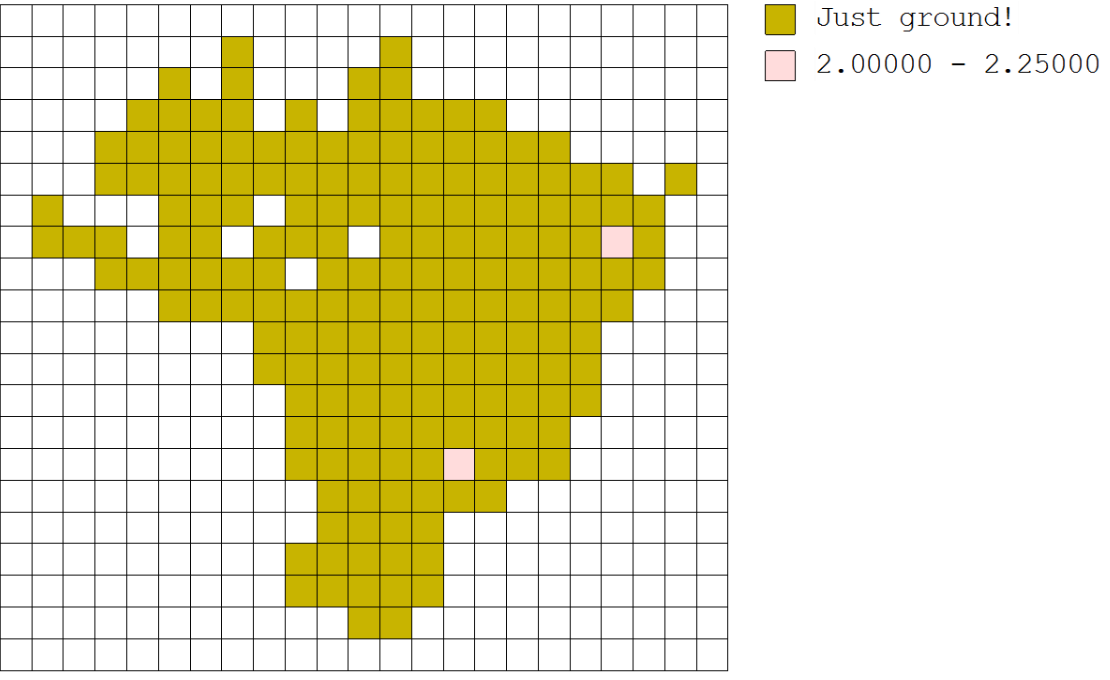

2 species give score:

Anelosimus jucundus (1.000) Micropholcus fauroti (1.000)

Consensus area 77 of 106 (from 1 areas; max. values)

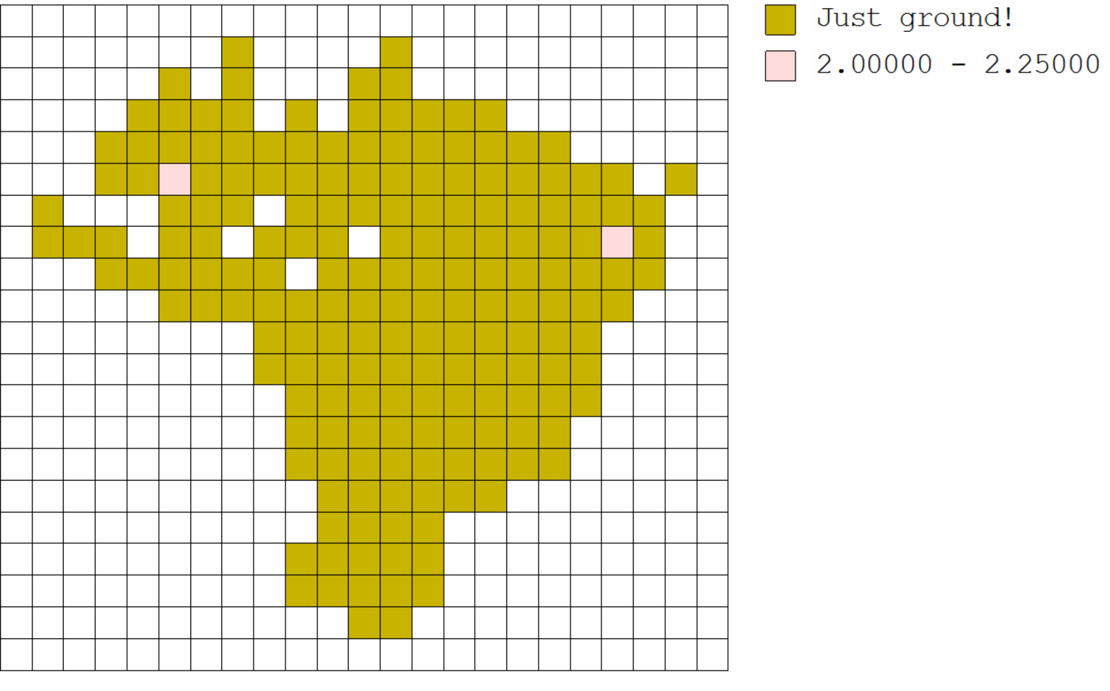

2 species give score:

Episinus nebulosus (1.000) Strophius fidelis (1.000)

Consensus area 78 of 106 (from 1 areas; max. values)

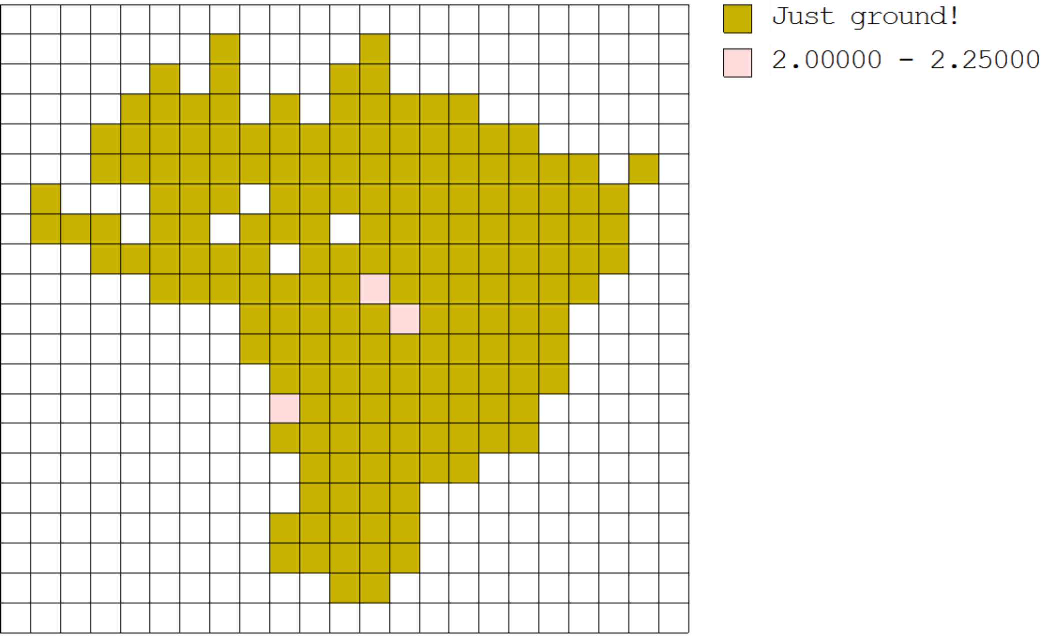

2 species give score:

Paratrechalea wygodzinskyi (1.000) Paratrechalea wygodzinsyi (1.000)

Consensus area 79 of 106 (from 1 areas; max. values)

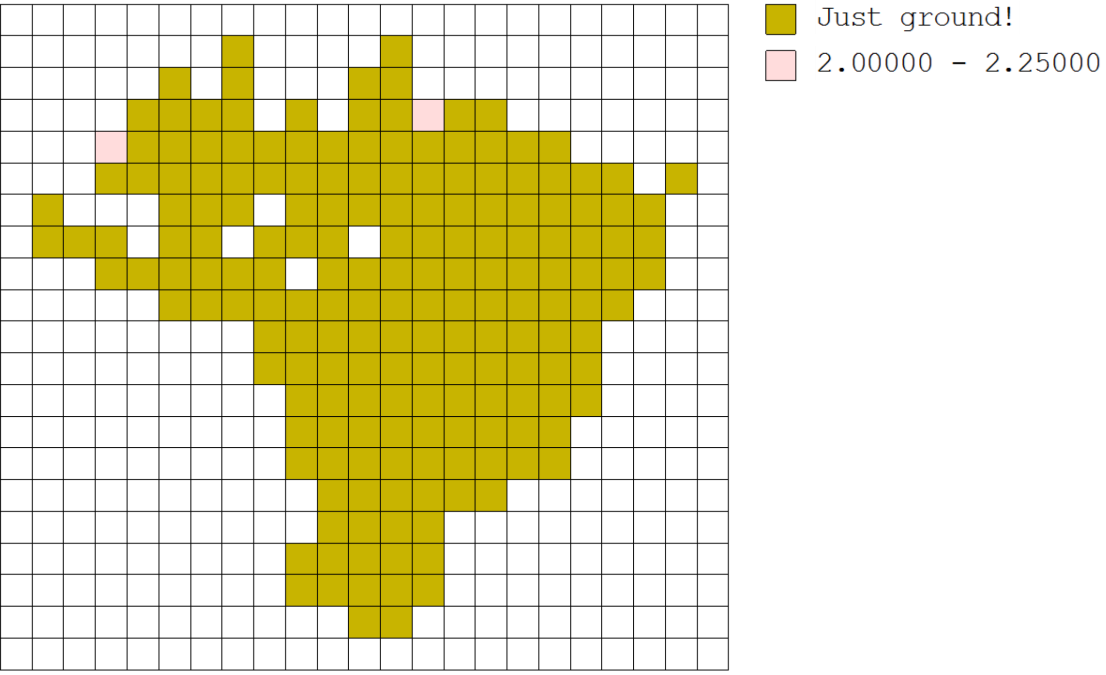

2 species give score:

Lyssomanes velox (1.000) Senoculus ruficapillus (1.000)

Consensus area 80 of 106 (from 1 areas; max. values)

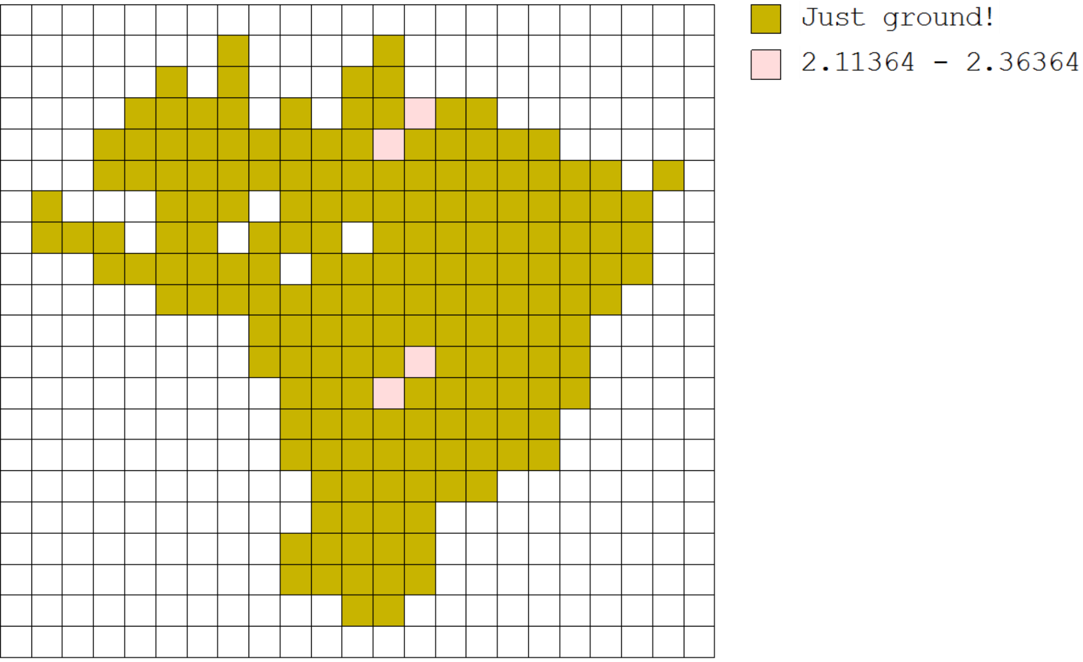

3 species give score:
Scopocira histrio(0.750)
Wixia_abdominalis(0.682)
Fluda goianinae(0.682)

Consensus area 81 of 106 (from 1 areas; max. values)

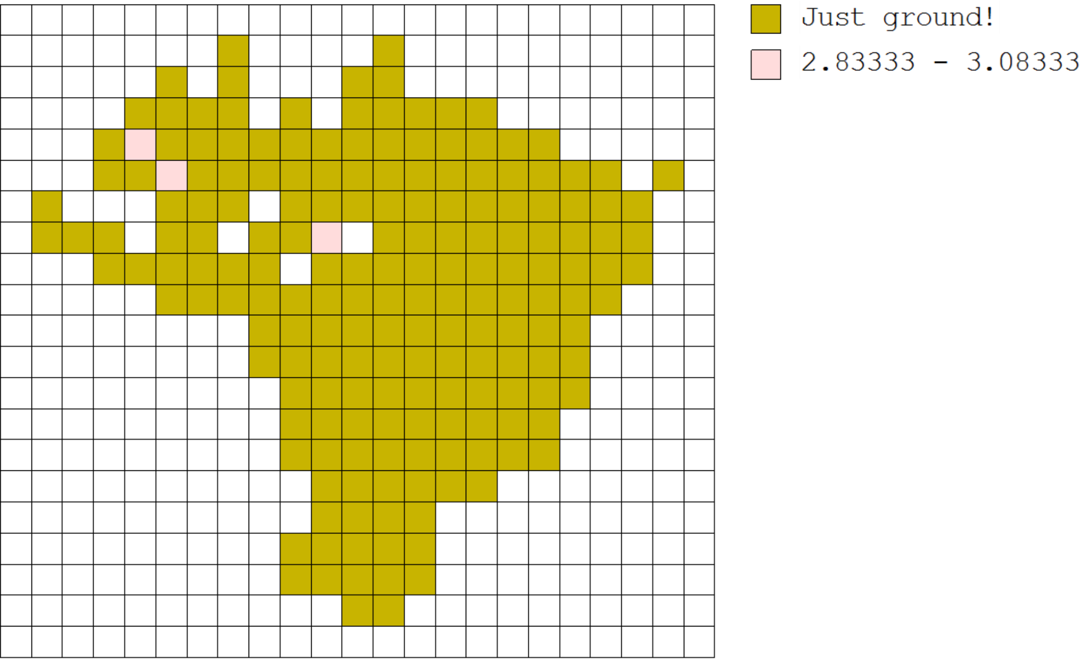

3 species give score:
Cerocida strigosa(0.833)
Dipoena_bryantae(1.000)
Dipoena duodecimpunctata(1.000)

Consensus area 82 of 106 (from 1 areas; max. values)

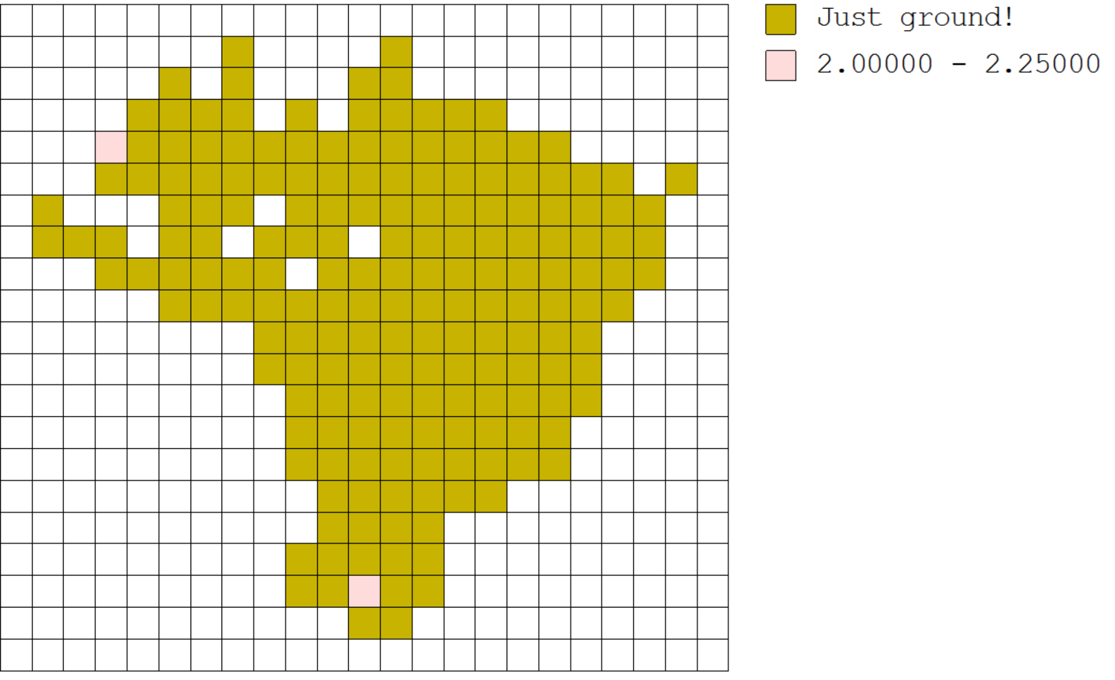

2 species give score:

Tmarus cinereus (1.000)

Tmarus posticatus (1.000)

Consensus area 83 of 106 (from 1 areas; max. values)

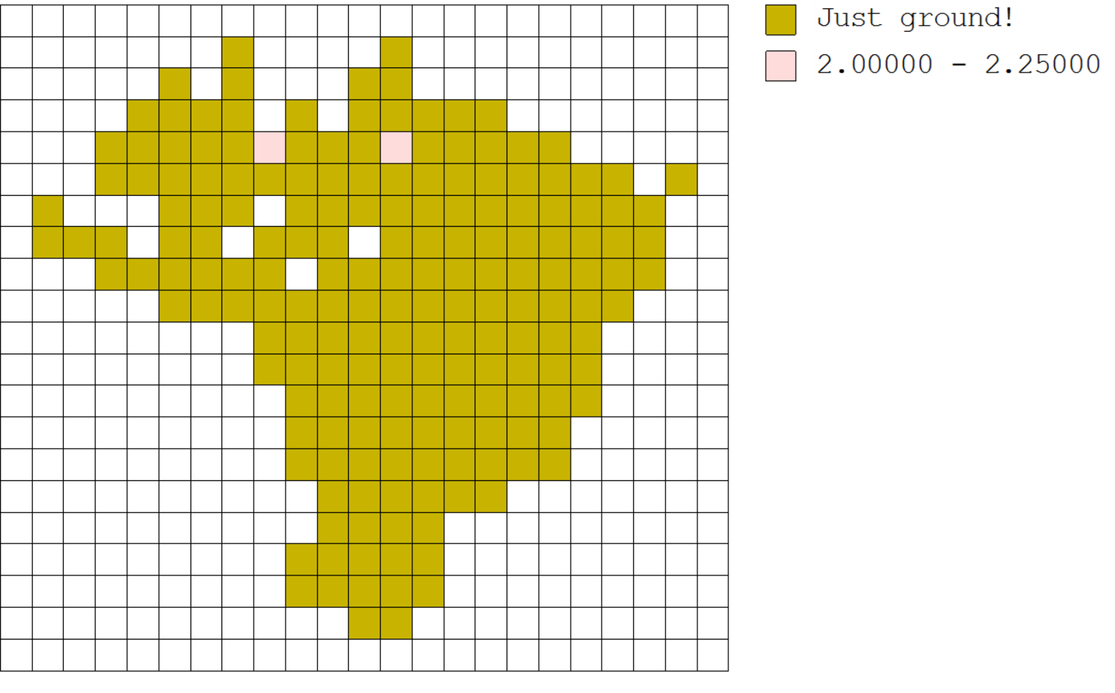

2 species give score:

Noegus arator (1.000)

Parawixia maldonado (1.000)

Consensus area 84 of 106 (from 1 areas; max. values)

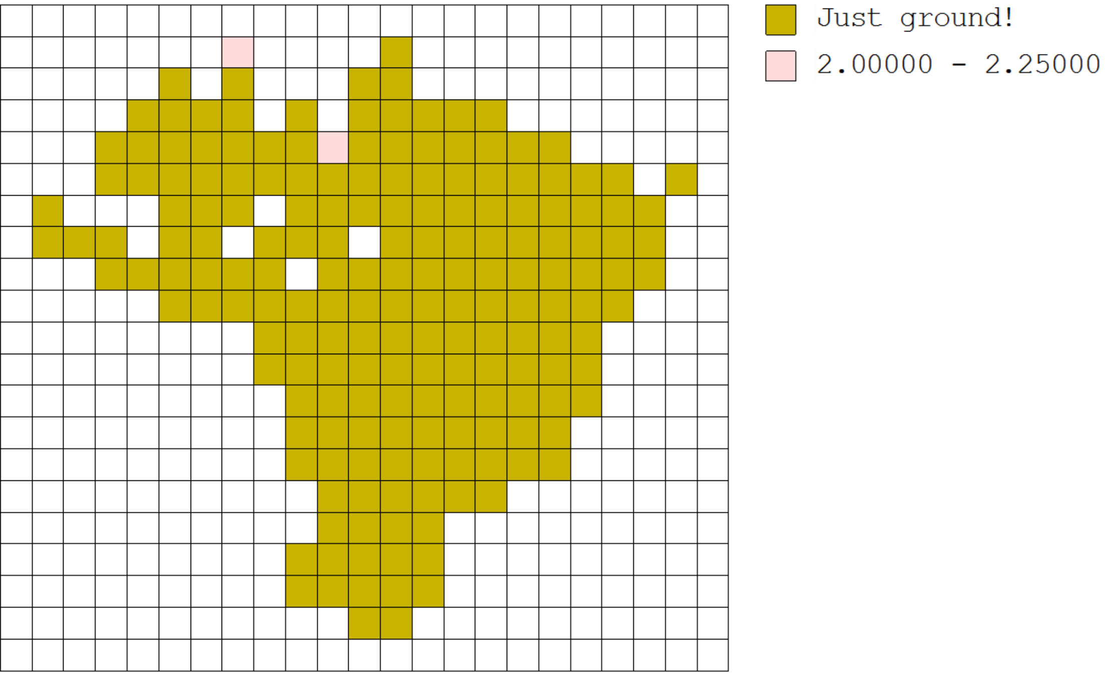

2 species give score:

Mangora pia(1.000)

Olios orchiticus(1.000)

Consensus area 85 of 106 (from 1 areas; max. values)

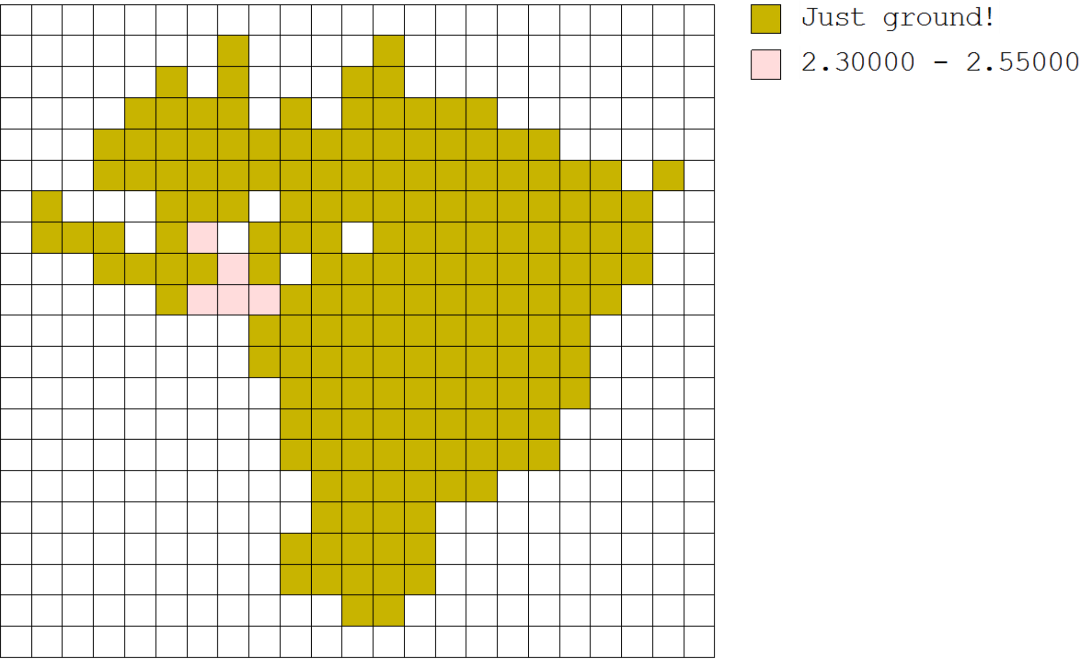

3 species give score:

Cyclosternum bicolor(0.700)

Cyriocosmus_chicoi(0.800)

Pamphobeteus crassifemur(0.800)

Consensus area 86 of 106 (from 1 areas; max. values)

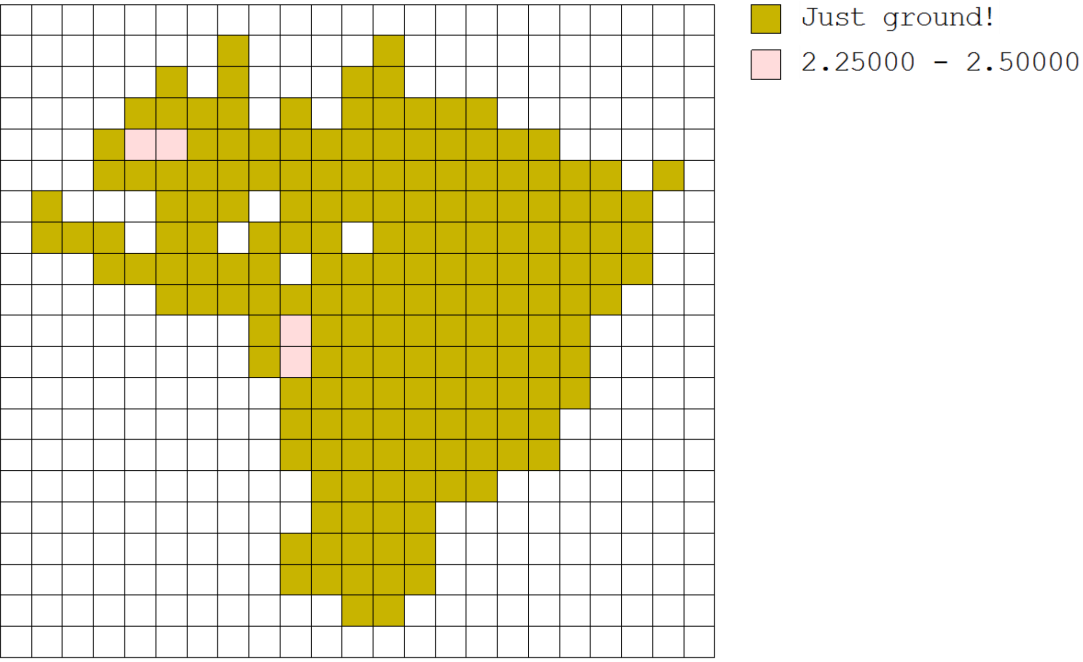

3 species give score:

Parawixia tarapoa(0.750)
Tapixaua callida(0.750)
Pensacola_murina(0.750)

Consensus area 87 of 106 (from 1 areas; max. values)

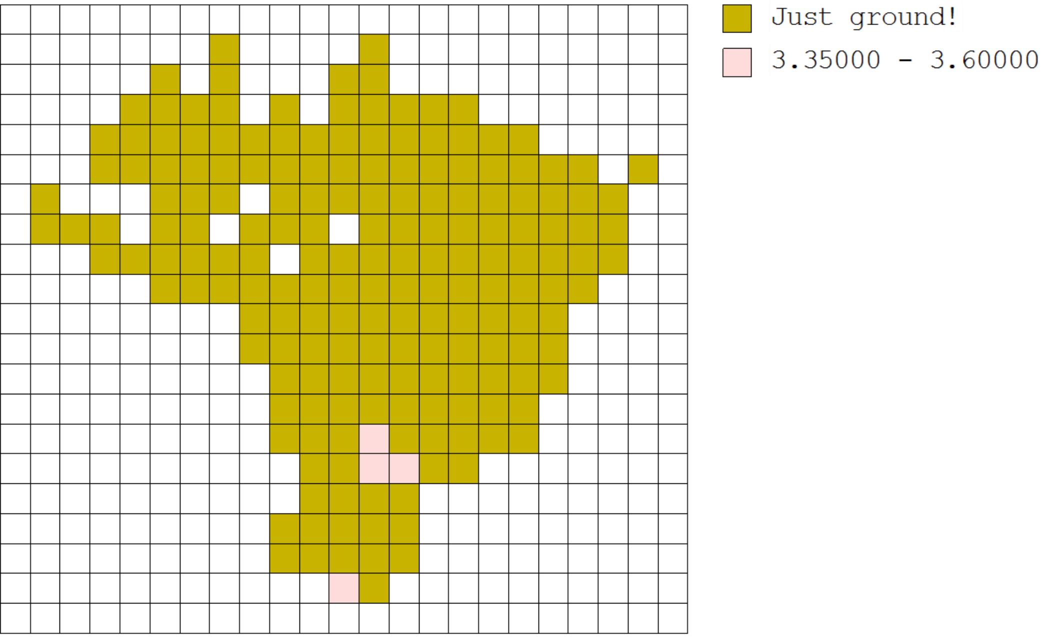

5 species give score:

Ariadna mollis(0.550)
Loxosceles hirsuta(0.750)
Cryptachaea lisei(0.650)
Aysha helvola(0.650)
Radulphius_bidentatus(0.750)

Consensus area 88 of 106 (from 1 areas; max. values)

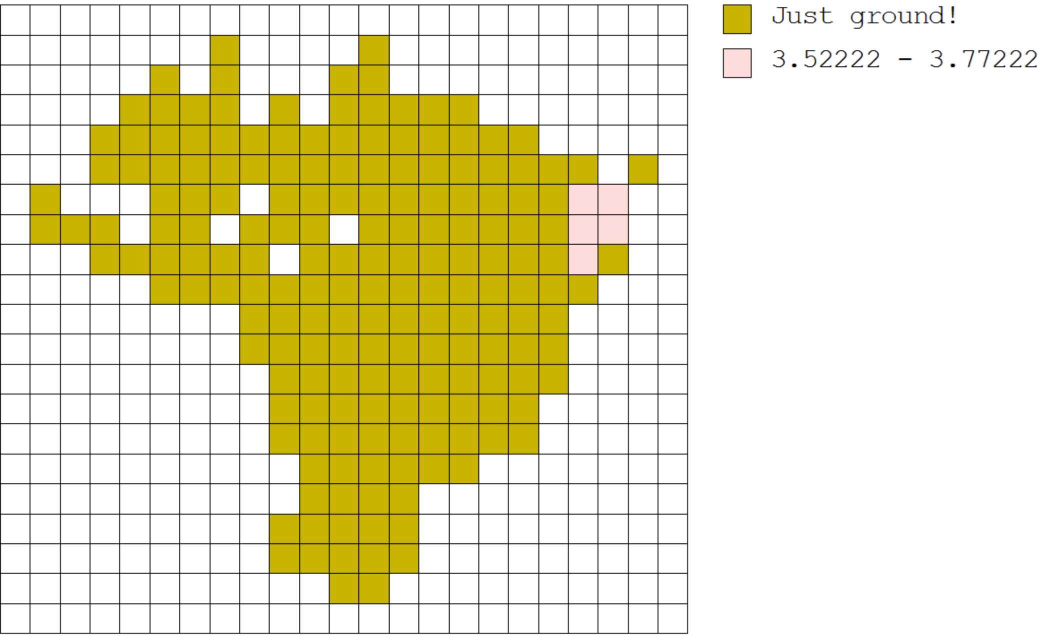

5 species give score:

Ariadna_obscura(0.700)
Iridopelma_hirsutum(0.622)
Pachistopelma_rufonigrum(0.800)
Ctenus_senex(0.700)
Kochiana brunneipes(0.700)

Consensus area 89 of 106 (from 1 areas; max. values)

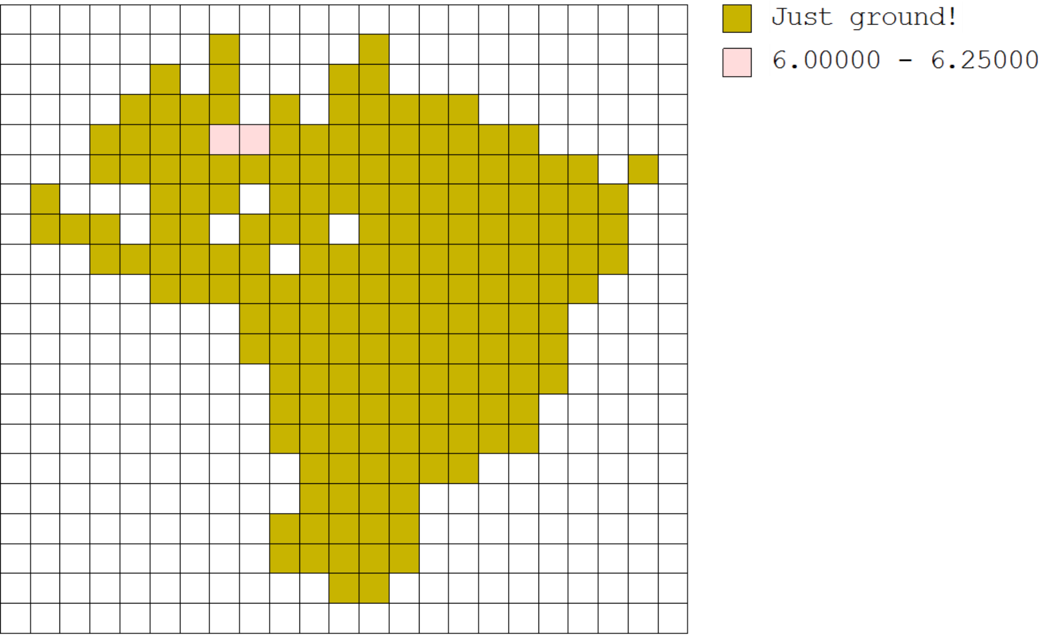

6 species give score:

Fernandezina saira(1.000)
Lyssomanes janauari(1.000)
Mangora balbina(1.000)
Meioneta adami(1.000)
Zimiromus atrifus(1.000)
Zimiromus boistus(1.000)

Consensus area 90 of 106 (from 1 areas; max. values)

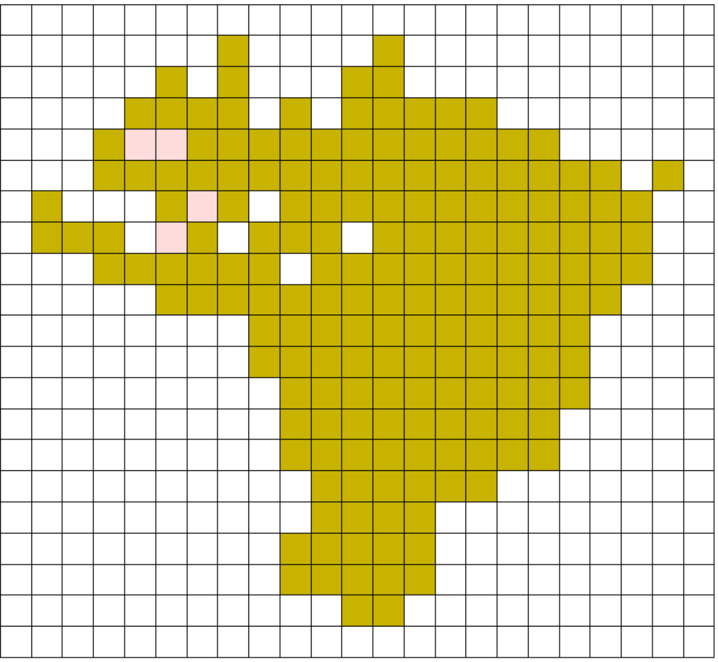

Just ground!

2.90625 - 3.15625

4 species give score:

Acragas longimanus(0.656)
Cotinusa puella(0.750)
Acragas quadriguttatus(0.750)
Itata tipuloides(0.750)

Consensus area 91 of 106 (from 1 areas; max. values)

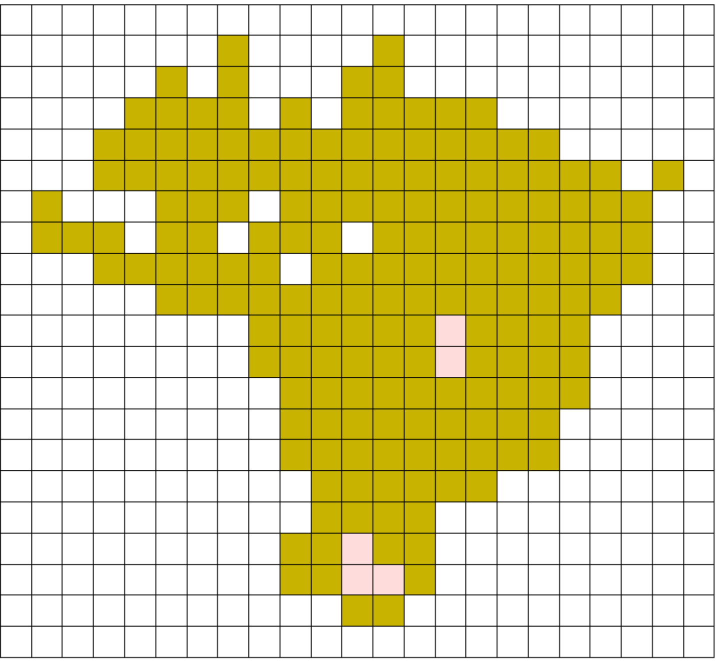

Just ground!

2.89000 - 3.14000

4 species give score:

Alpaida albocincta(0.720)
Ocrepeira pinhal(0.560)
Misumenops callinurus(0.810)
Tmarus elongatus(0.800)

Consensus area 92 of 106 (from 1 areas; max. values)

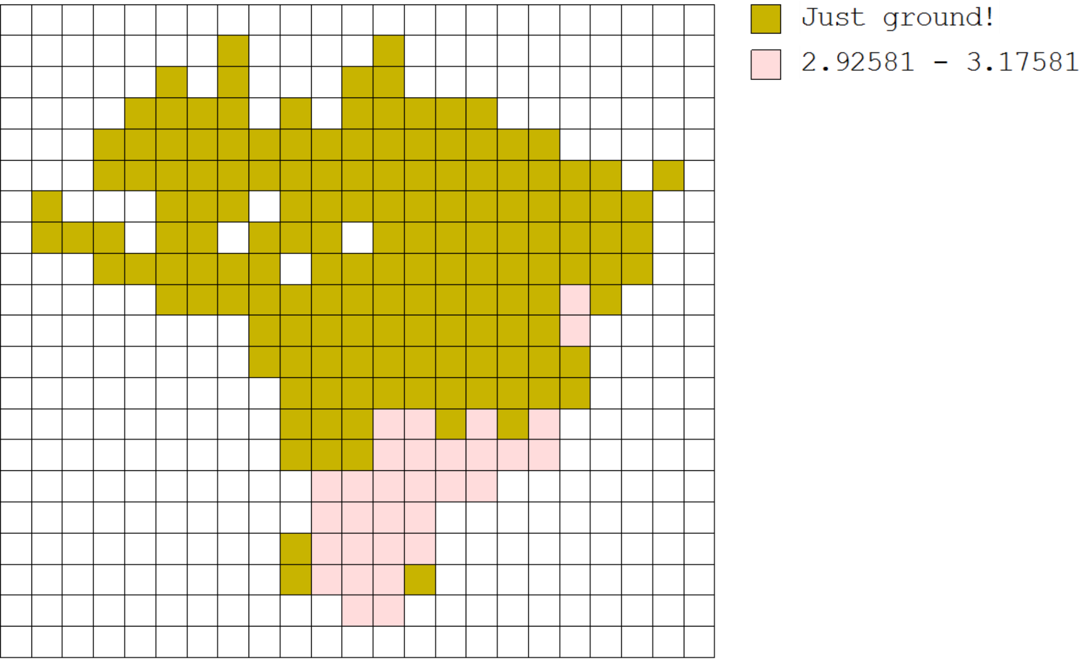

4 species give score:

Araneus stabilis(0.774)
Araneus unanimus(0.806)
Jessica osoriana(0.603)
Vinnius uncatus(0.742)

Consensus area 93 of 106 (from 1 areas; max. values)

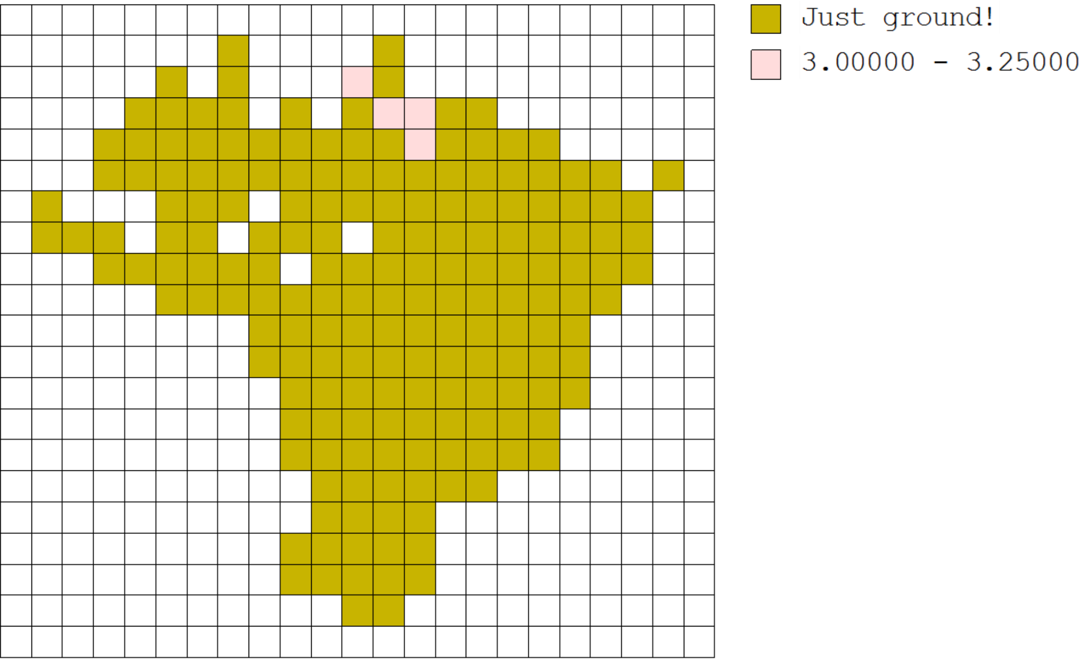

4 species give score:

Alpaida manicata(0.750)
Chaetacis picta(0.750)
Chirothecia clavimana(0.750)
Synemosyna lucasi(0.750)

Consensus area 94 of 106 (from 2 areas; max. values)

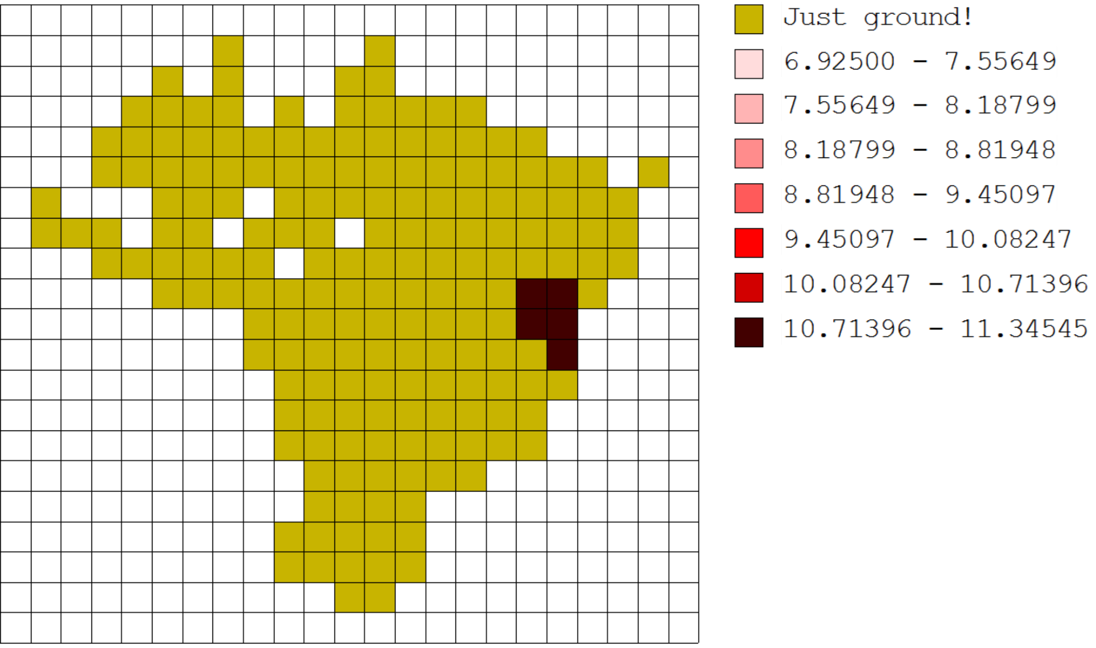

18 species give score:

Avicularia_diversipes(0.700-0.750)
Celaetycheus_flavostriatus(0.700-0.750)
Eustiromastix bahiensis(0.000-0.573)
Leprolochus_mucuge(0.000-0.750)
Otiothops atlanticus(0.800-0.875)
Radulphius_singularis(0.000-0.700)
Scytodes_iabaday(0.000-0.700)
Tmesiphantes nubilus(0.700-0.900)
Predatoroonops blain(0.700-0.750)
Carapoia_rheimsae(0.000-0.700)
Ctenus_paubrasil(0.000-0.573)
Iridopelma zorodes(0.445-0.450)
Martella_gandu(0.000-0.700)
Phoneutria bahiensis(0.000-0.655)
Romitia_patellaris(0.000-0.700)
Tmesiphantes_caymmii(0.700-0.750)
Pachistopelma bromelicola(0.350-0.364)
Sicarius ornatus(0.736-0.800)

Consensus area 95 of 106 (from 1 areas; max. values)

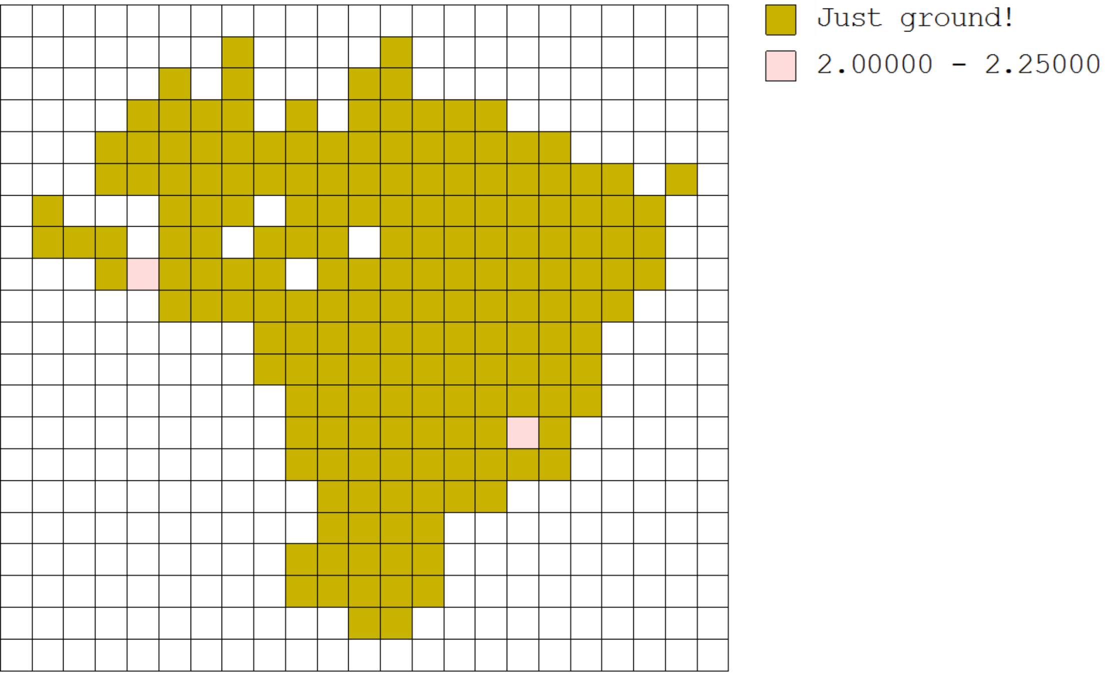

2 species give score:

Loxosceles anomala(1.000)
Aillutticus montans(1.000)

Consensus area 96 of 106 (from 1 areas; max. values)

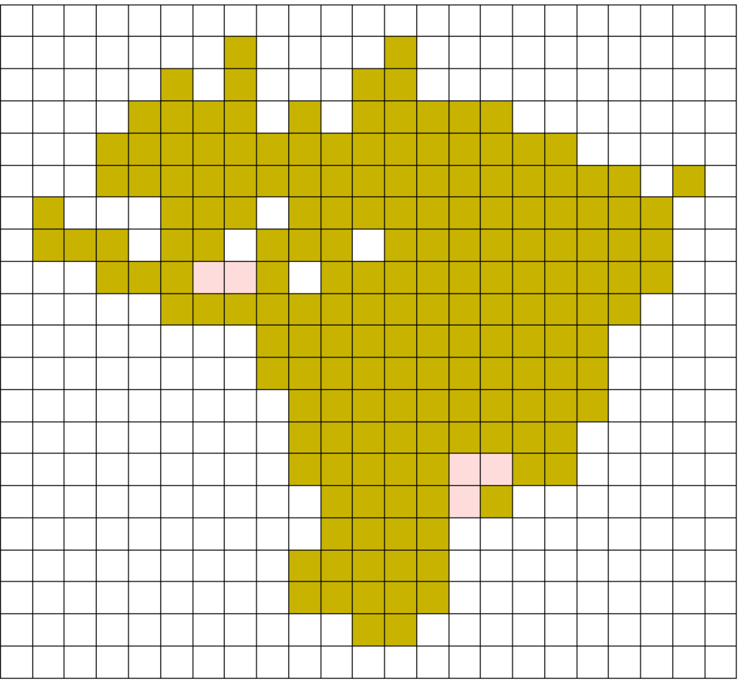

Just ground!

7.12222 - 7.37222

10 species give score:

Carapoia genitalis (0.700)
Eustala guttata (0.700)
Mesabolivar simoni (0.700)
Trachelas robustus (0.622)
Wagneriana juquia (0.900)
Epicadinus tuberculatus (0.700)
Mesabolivar banksi (0.700)
Thymoites ilvan (0.700)
Umuara juquia (0.700)
Wamba congener (0.700)

Consensus area 97 of 106 (from 1 areas; max. values)

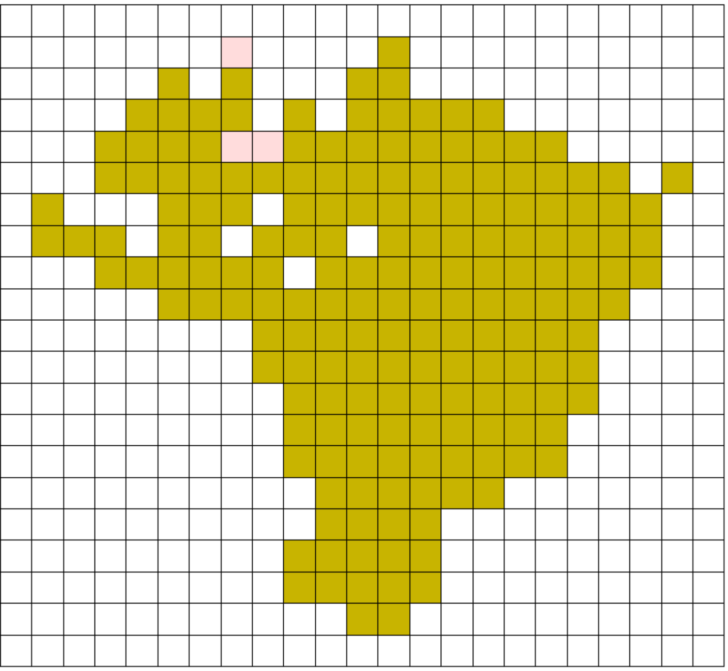

Just ground!

10.00000 - 10.25000

12 species give score:

Acentroscelus_guianensis (0.833)
Hypognatha_deplanata (0.833)
Maraca_horrida (0.833)
Mysmenopsis_ischnamigo (0.833)
Scytodes_saci (0.833)
Thymoites_maracayensis (0.833)
Anelosimus_chickeringi (0.833)
Leprolochus_spinifrons (0.833)
Micrepeira_pachitea (0.833)
Parawixia_tomba (0.833)
Theridion_artum (0.833)
Breda_akypueruna (0.833)

Consensus area 98 of 106 (from 1 areas; max. values)

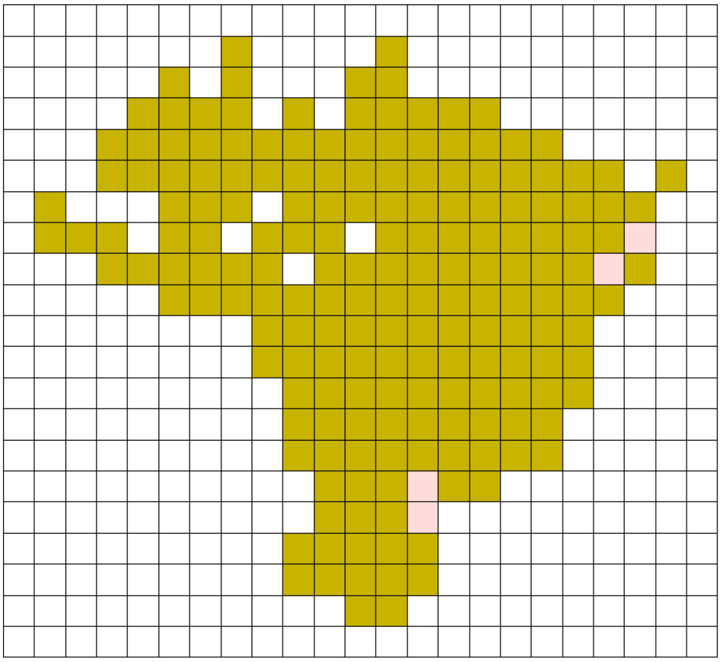

Just ground!

2.15625 - 2.40625

3 species give score:

Dyrines_brescoviti (0.750)

Theridula multiguttata (0.750)

Paratheridula_perniciosa (0.656)

Consensus area 99 of 106 (from 1 areas; max. values)

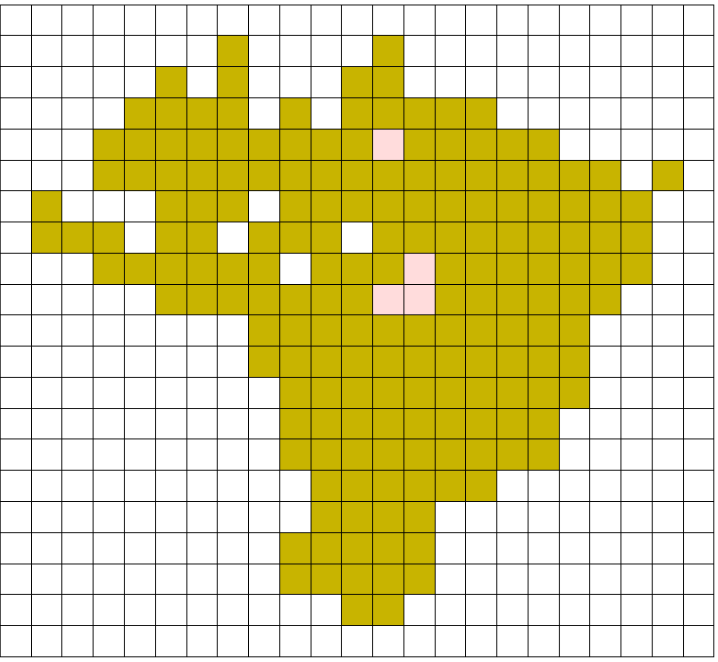

Just ground!

2.25000 - 2.50000

3 species give score:

Alpaida chickeringi (0.750)

Thaumasia caxiuana (0.750)

Noegus_rufus (0.750)

Consensus area 100 of 106 (from 1 areas; max. values)

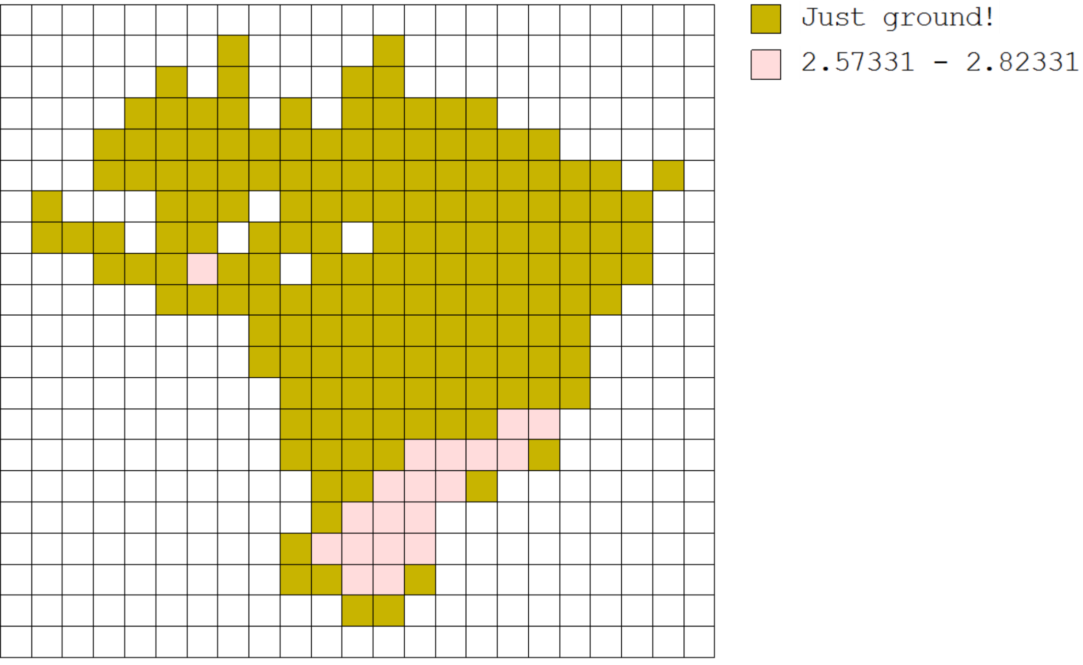

3 species give score:

Apodrassodes mono(0.789)
Micrathena_crassispina(0.855)
Micrathena furcata(0.929)

Consensus area 101 of 106 (from 1 areas; max. values)

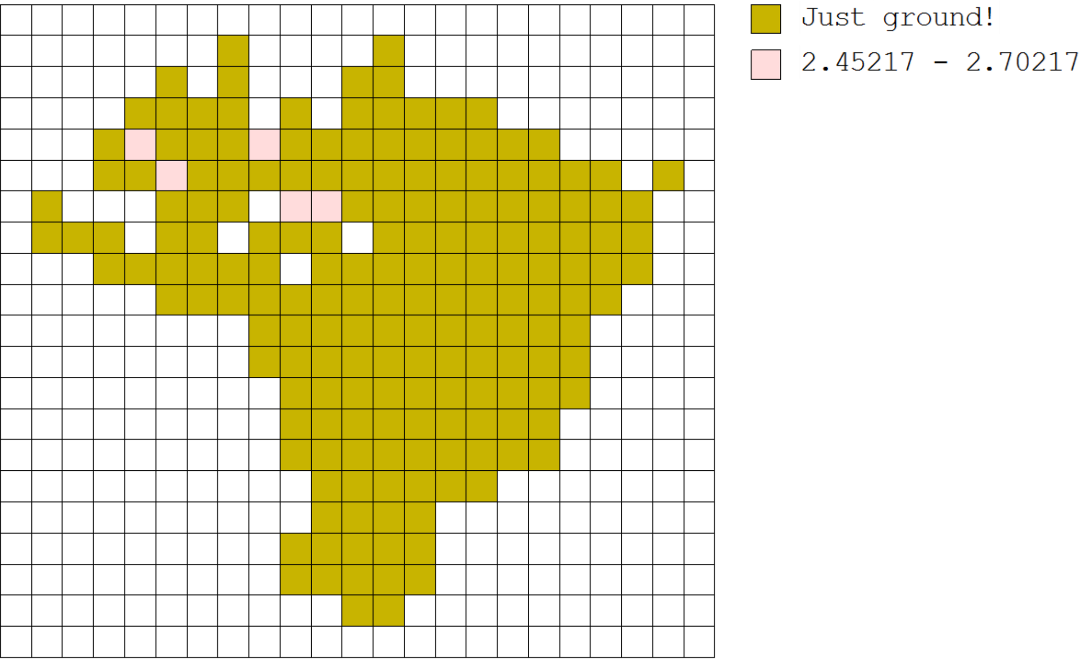

3 species give score:

Carapoia ocaina(0.900)
Mangora_keduc(0.822)
Novafrontina uncata(0.730)

Consensus area 102 of 106 (from 1 areas; max. values)

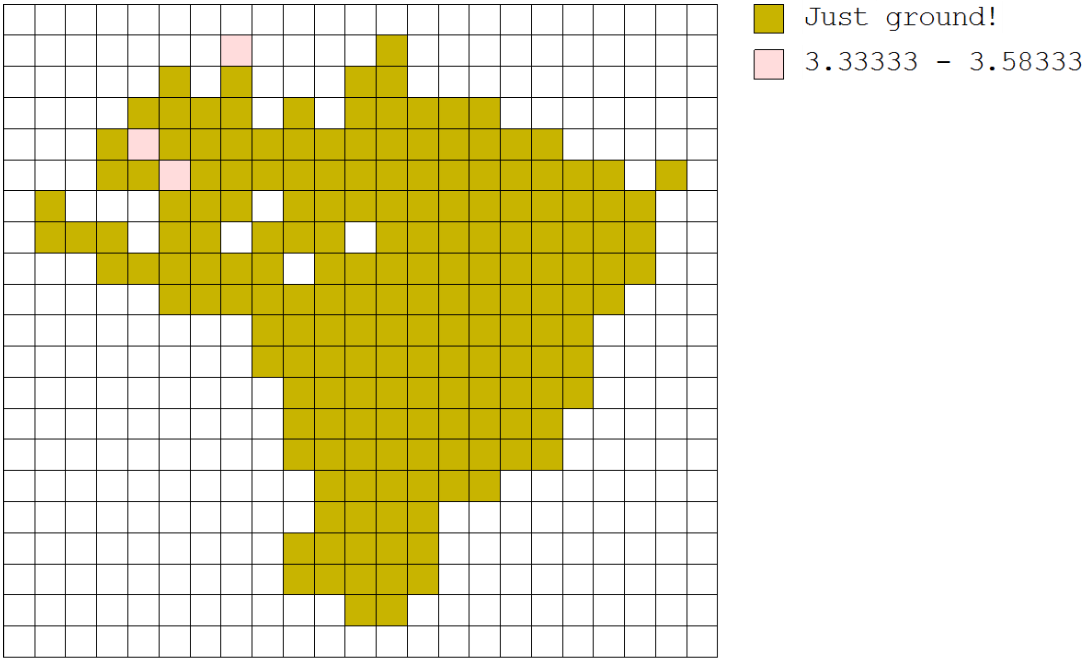

4 species give score:

Chrysso calima (0.833)
Cryptachaea pydanieli (0.833)
Dipoena tingo (0.833)
Tekellina bella (0.833)

Consensus area 103 of 106 (from 1 areas; max. values)

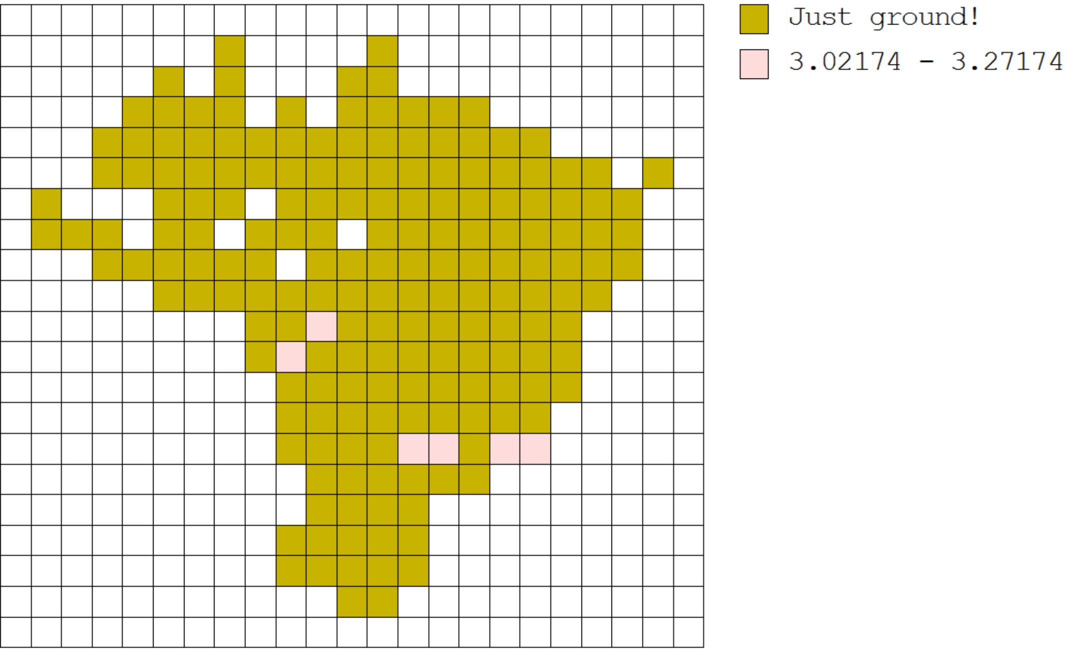

4 species give score:

Episinus bicorniger (0.750)
Erica eugenia (0.750)
Eupalaestrus spinosissimus (0.761)
Wagneriana janeiro (0.761)

Consensus area 104 of 106 (from 1 areas; max. values)

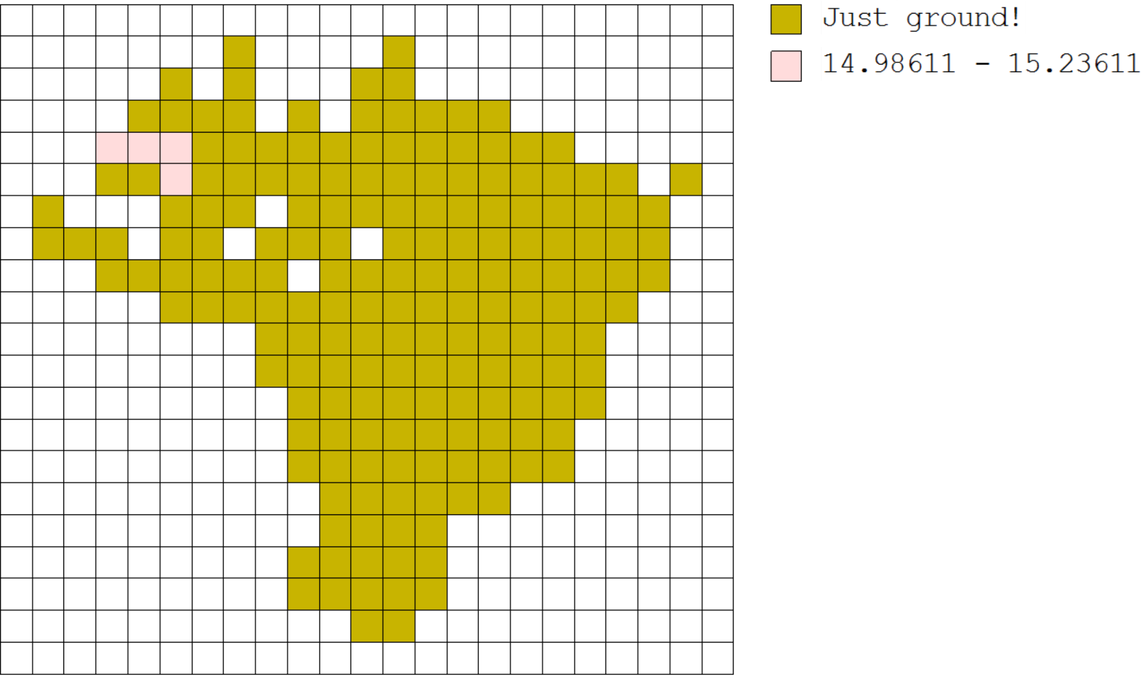

20 species give score:

Amycus annulatus (0.750)	Amycus ectypus (0.750)
Asaracus semifimbriatus (0.750)	Corinna ferox (0.750)
Cryptachaea schneirlai (0.750)	Dipoena olivenca (0.750)
Lasaeola donaldi (0.750)	Mago longidens (0.750)
Mago steindachneri (0.750)	Micrathena exlinae (0.750)
Micrathena ucayali (0.583)	Misumenops robustus (0.750)
Onocolus compactilis (0.750)	Oxyopes hemorrhous (0.875)
Phireza sexmaculata (0.750)	Stenodeza acuminata (0.750)
Thammaca nigritarsis (0.750)	Theridion incertissimum (0.750)
Tmarus viridis (0.750)	Micrathena necopinata (0.778)

Consensus area 105 of 106 (from 1 areas; max. values)

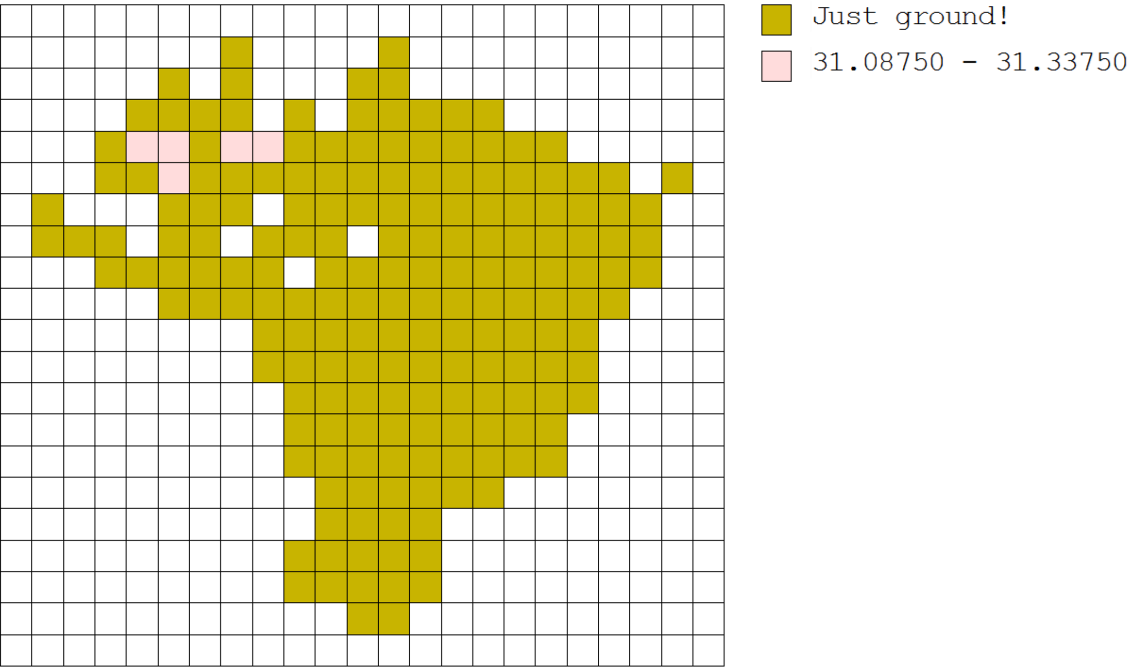

43 species give score:

Amazonepeira_herrera(0.900)
Anyphaenoides_pacifica(0.700)
Centroctenus_miriuma(0.613)
Ctenus_manauara(0.700)
Dolichognatha_ducke(0.700)
Encyosaccus_sexmaculatus(0.700)
Epicratinus_amazonicus(0.800)
Hingstepeira_dimona(0.800)
Hypaeus_miles(0.700)
Hypognatha_colosso(0.700)
Lygromma_gasnieri(0.800)
Mangora_mamiraua(0.700)
Metazygia_uma(0.700)
Micrathena_embira(0.800)
Ochyrocera_hamadryas(0.700)
Parachemmis_manauara(0.700)
Rhoicinus_urucu(0.700)
Scytodes_martiusi(0.700)
Stethorrhagus_lupulus(0.700)
Zimiromus_kleini(0.700)
Breda_nanica(0.700)
Neoxyphinus petrogoblin(0.800)
Anapis_castilla(0.700)
Centroctenus_acara(0.700)
Corinna_recurva(0.700)
Cyclosa_vieirae(0.613)
Dyrines_ducke(0.700)
Ephebopus_uatuman(0.700)
Gelanor_heraldicus(0.700)
Hingstepeira_folisecens(0.800)
Hypaeus_triplagiatus(0.700)
Litoporus_dimona(0.800)
Lygromma_huberti(0.700)
Mangora_sumauma(0.900)
Micrathena_coca(0.787)
Myrmecotypus_olympus(0.700)
Pachomius_sextus(0.700)
Peucetia_macroglossa(0.675)
Scytodes_balbina(0.700)
Selenops_lavillai(0.700)
Synotaxus_waiwai(0.700)
Zimiromus_syenus(0.700)
Micrathena_abrahami(0.700)
