# Supplementary material for: Delimiting Areas of Endemism through Kernel Interpolation
Source: PLoS One. 2015 Jan 22;10(1):e0116673. doi: 10.1371/journal.pone.0116673 (PMC4303434; doi:10.1371/journal.pone.0116673)

Tutorial for Geographical Interpolation of Endemism (GIE)

This tutorial provides instructions to perform the implementation of Geographic Interpolation of Endemism (GIE) in ArcGIS. In addition, the tutorial also shows the instructions for using the software *Geographic distance for GIE,* that calculates the distance used as area of influence for interpolation.

Adding GIE toolbox in ArcGIS

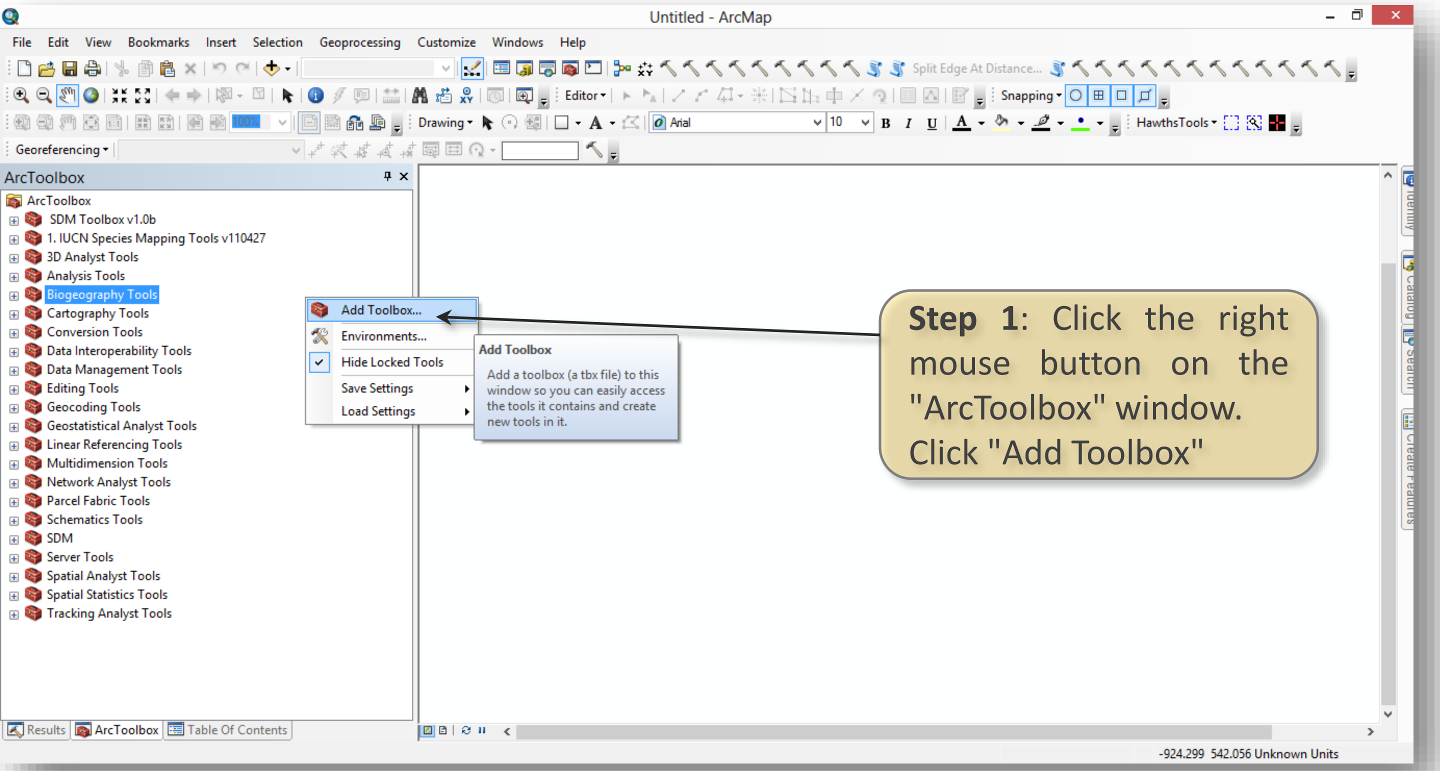

Step 1: Click the right mouse button on the "ArcToolbox" window. Click "Add Toolbox"

Step 2: Select the file GIE.tbx

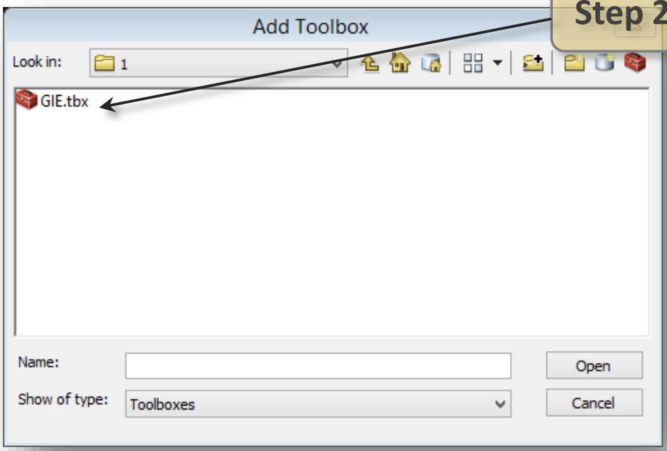

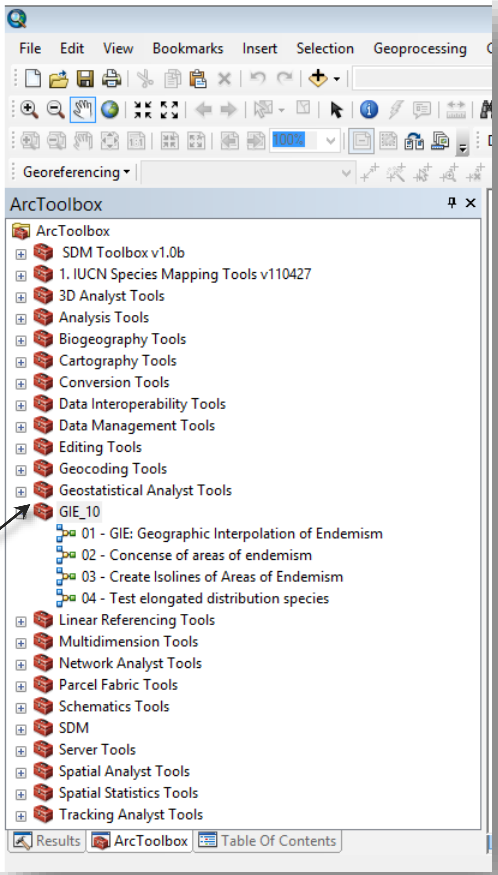

GIE toolbox was added to ArcToolbox in ArcGIS

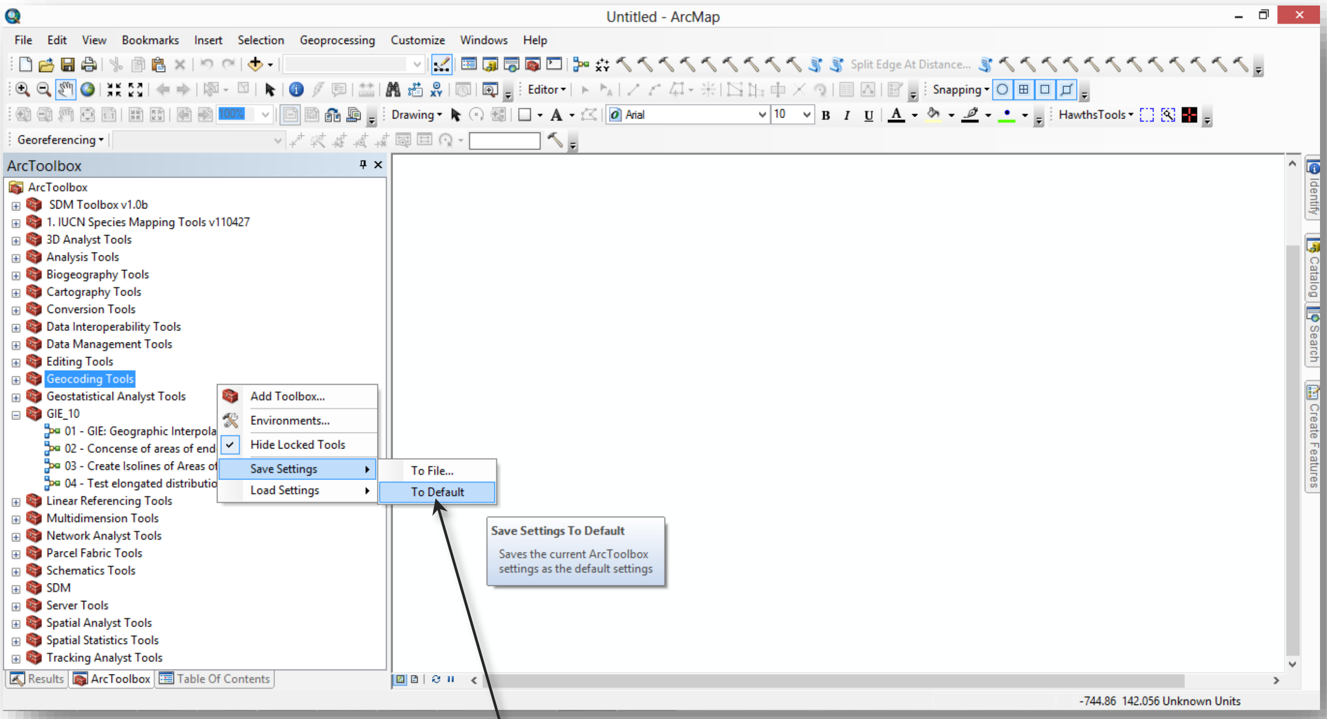

Step 3: To keep the GIE toolbox permanently in the ArcToolbox window click the right mouse button on the window: click “Save Settings" → "To Default"

Performing the GIE analysis

Step 1: Select the type of file used, Text (txt) or Excel (xls)

Step 2: Select the file containing: taxon name, longitude and latitude. The file must not contain headings. Use tab as column separator in txt file. In xls files, the worksheet name must be "data"tab

For additional instructions

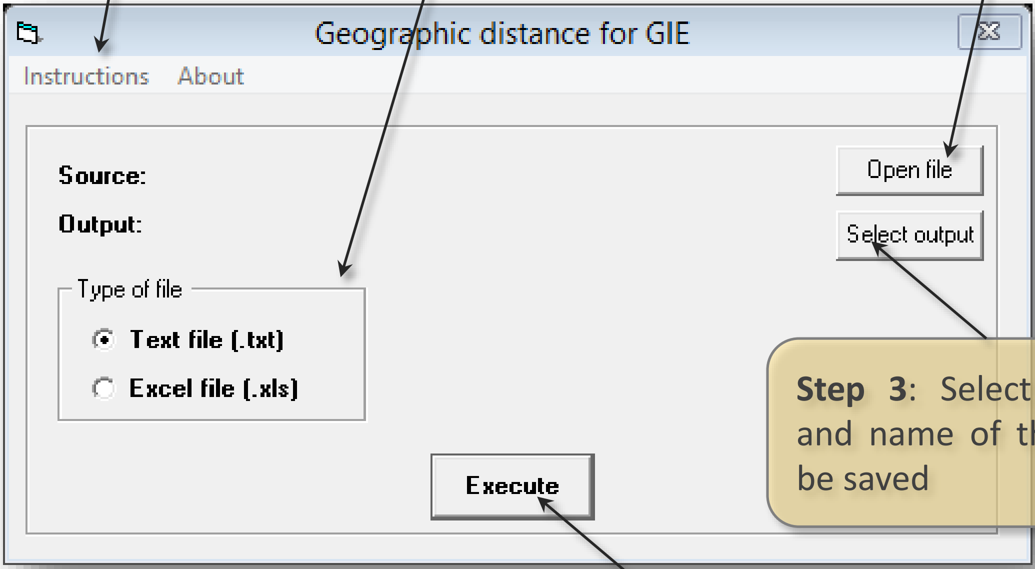

Step 3: Select location and name of the file to be saved

Step 4: Run the function

Result file containing the taxon name and the distance between the centroid and the farthest point of occurrence.

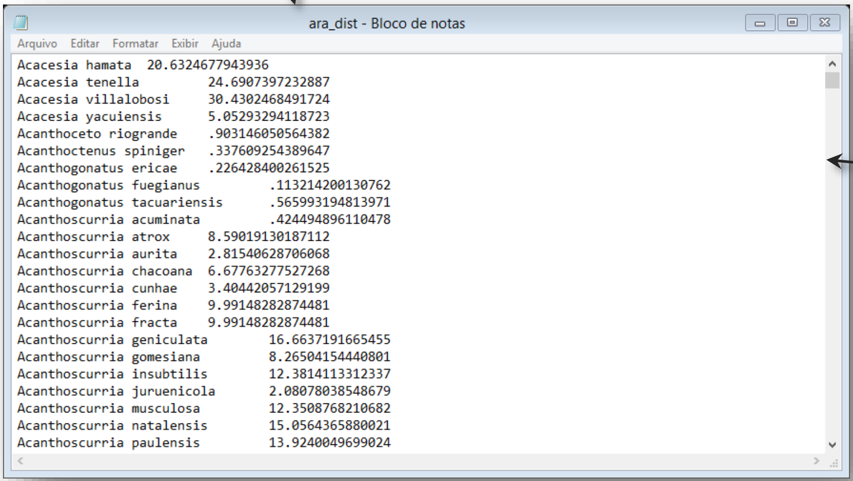

Acacesia hamata	20.6324677943936
Acacesia tenella	24.6907397232887
Acacesia villalobosi	30.4302468491724
Acacesia yacuiensis	5.05293294118723
Acanthoceto riogrande	.903146050564382
Acanthoctenus spiniger	.337609254389647
Acanthogonatus ericae	.226428400261525
Acanthogonatus fuegianus	.113214200130762
Acanthogonatus tacuariensis	.565993194813971
Acanthoscurria acuminata	.424494896110478
Acanthoscurria atrox	8.59019130187112
Acanthoscurria aurita	2.81540628706068
Acanthoscurria chacoana	6.67763277527268
Acanthoscurria cunhae	3.40442057129199
Acanthoscurria ferina	9.99148282874481
Acanthoscurria fracta	9.99148282874481
Acanthoscurria geniculata	16.6637191665455
Acanthoscurria gomesiana	8.26504154440801
Acanthoscurria insubtilis	12.3814113312337
Acanthoscurria juruenicola	2.08078038548679
Acanthoscurria musculosa	12.3508768210682
Acanthoscurria natalensis	15.0564365880021
Acanthoscurria paulensis	13.9240049699024

Step 5: These distances should be used to create the classes of range size . Thus, taxa with similar ranges will be in the same class. Each class will be analyzed separately in the next step (GIE analysis). For this, separate the points of occurrence of taxa of the same range size class in separate files.

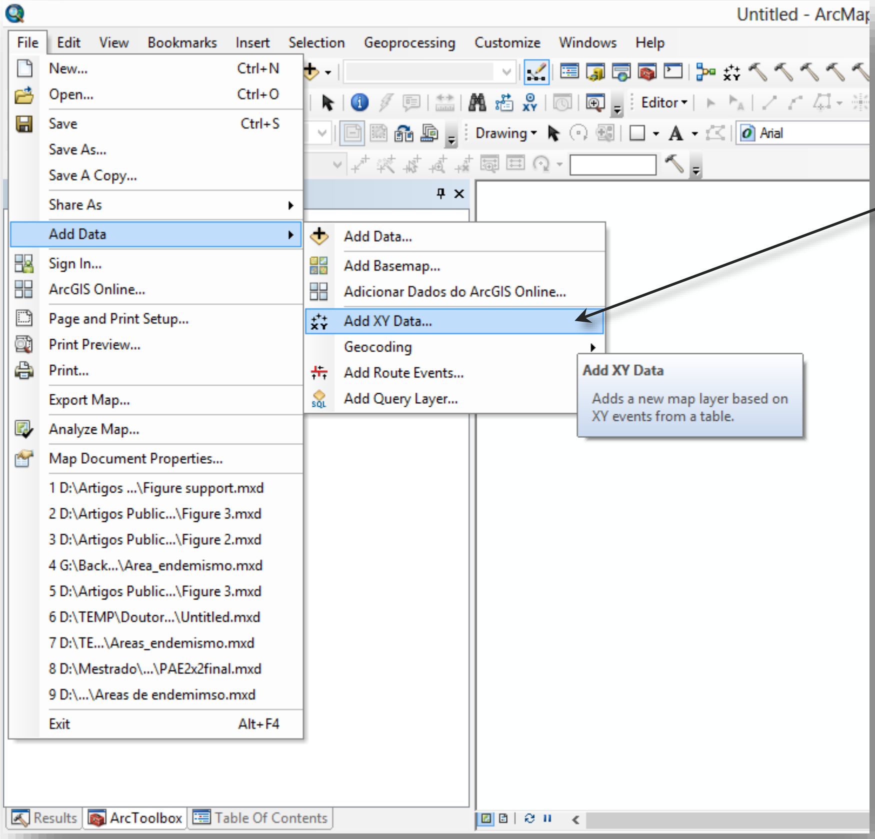

Step 6: To add the points of occurrence to ArcGIS click: File → Add Data → Add XY Data. This procedure must be done for each range size class separately.

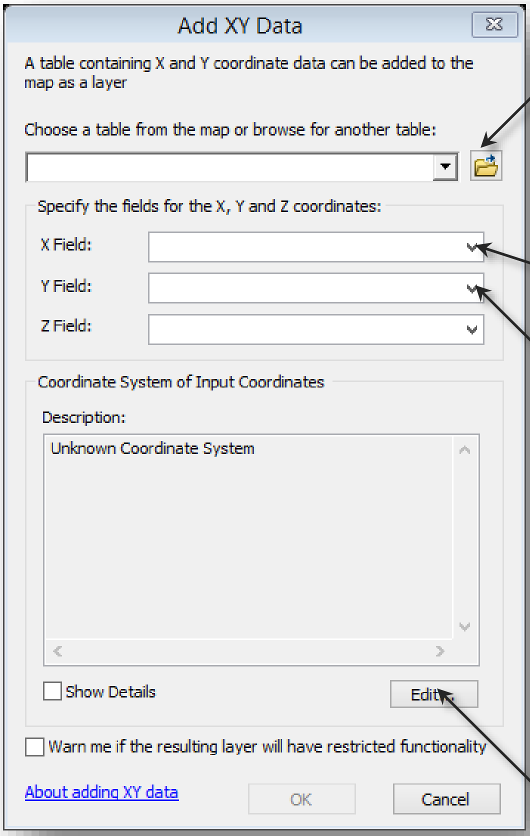

Step 7: Select the file with points of occurrence of species of the same range size class .

Step 8: Select the field with longitude data

Step 9: Select the field with latitude data

Step 10: Select geographic coordinate system (WGS84 is recommended)

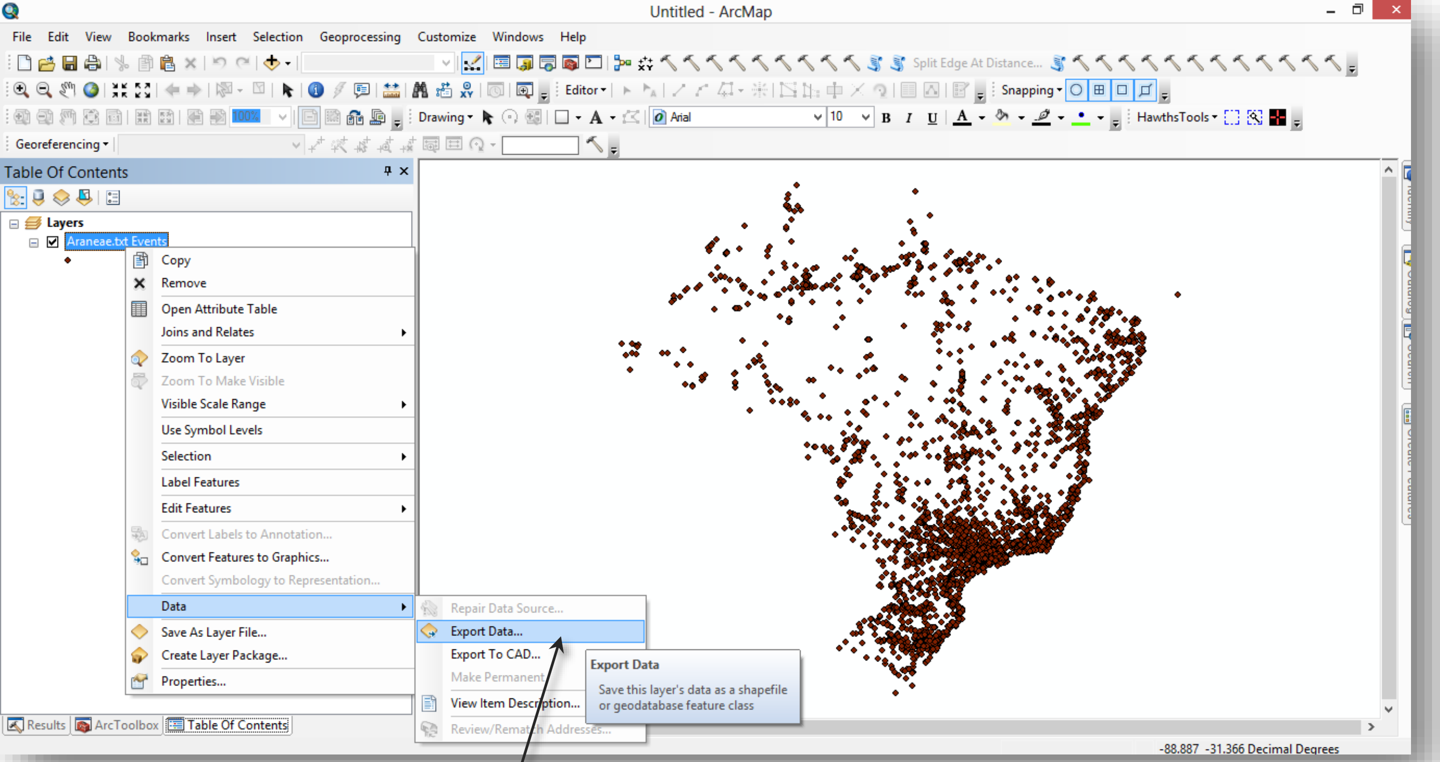

Step 11: Save your file (shapefile) with points of occurrence of species of the same range size class.

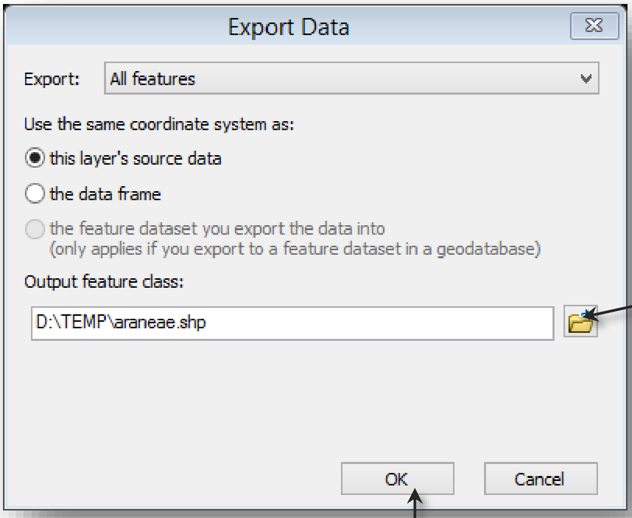

Step 12: Select location and name of the file to be saved.

Step 13: After saving, click "*yes*" for "*Do you want to add the exported data to the map as a layer?*"

Step 14: Select the points (shapefile) created in step 13.

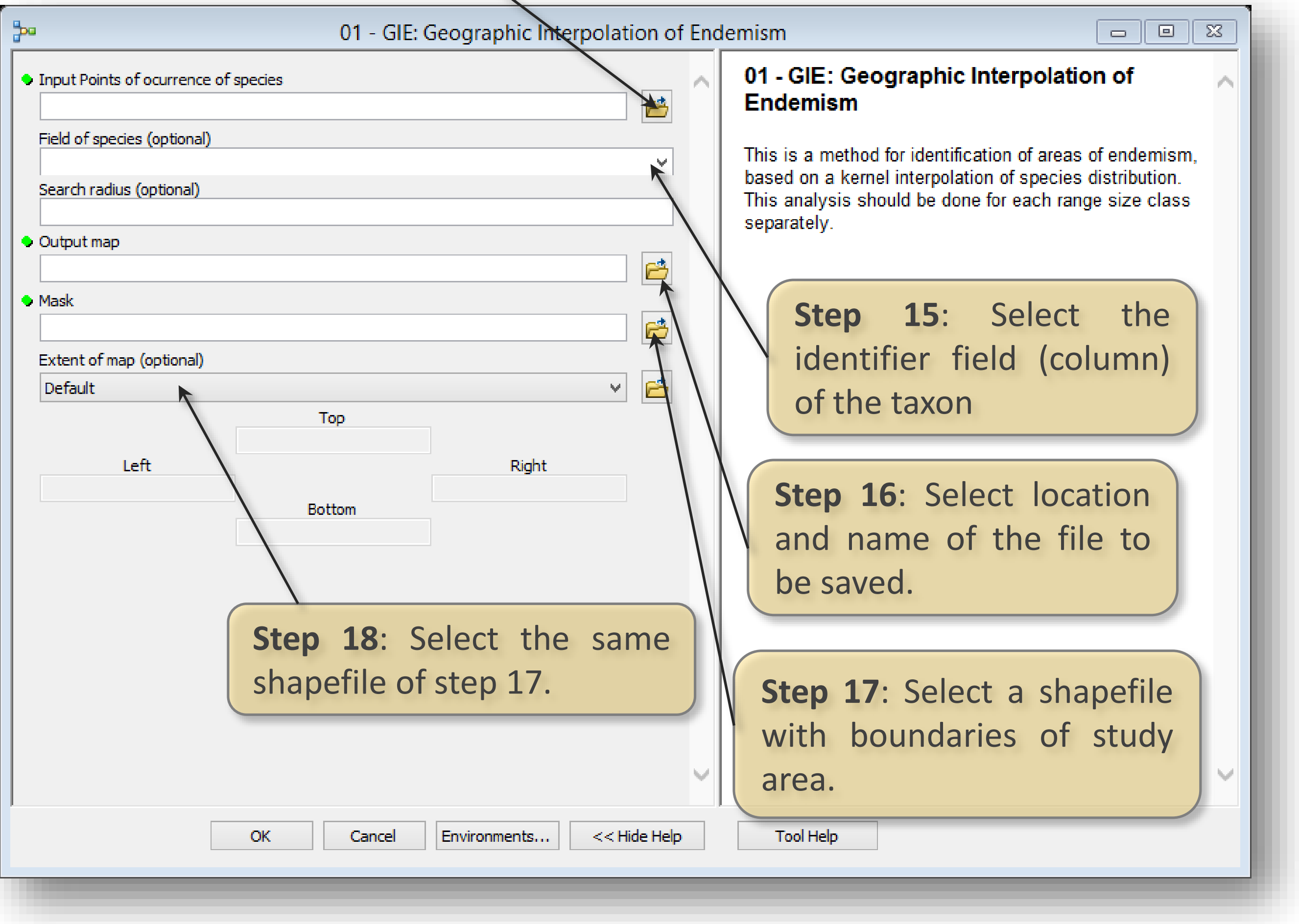

Step 15: Select the identifier field (column) of the taxon

Step 16: Select location and name of the file to be saved.

Step 17: Select a shapefile with boundaries of study area.

Step 18: Select the same shapefile of step 17.

Warning: This procedure must be repeated for each range size class separately.

After performing the procedures of GIE (previous steps) for each range size class, use this tool to generate the consensus of areas of endemism.

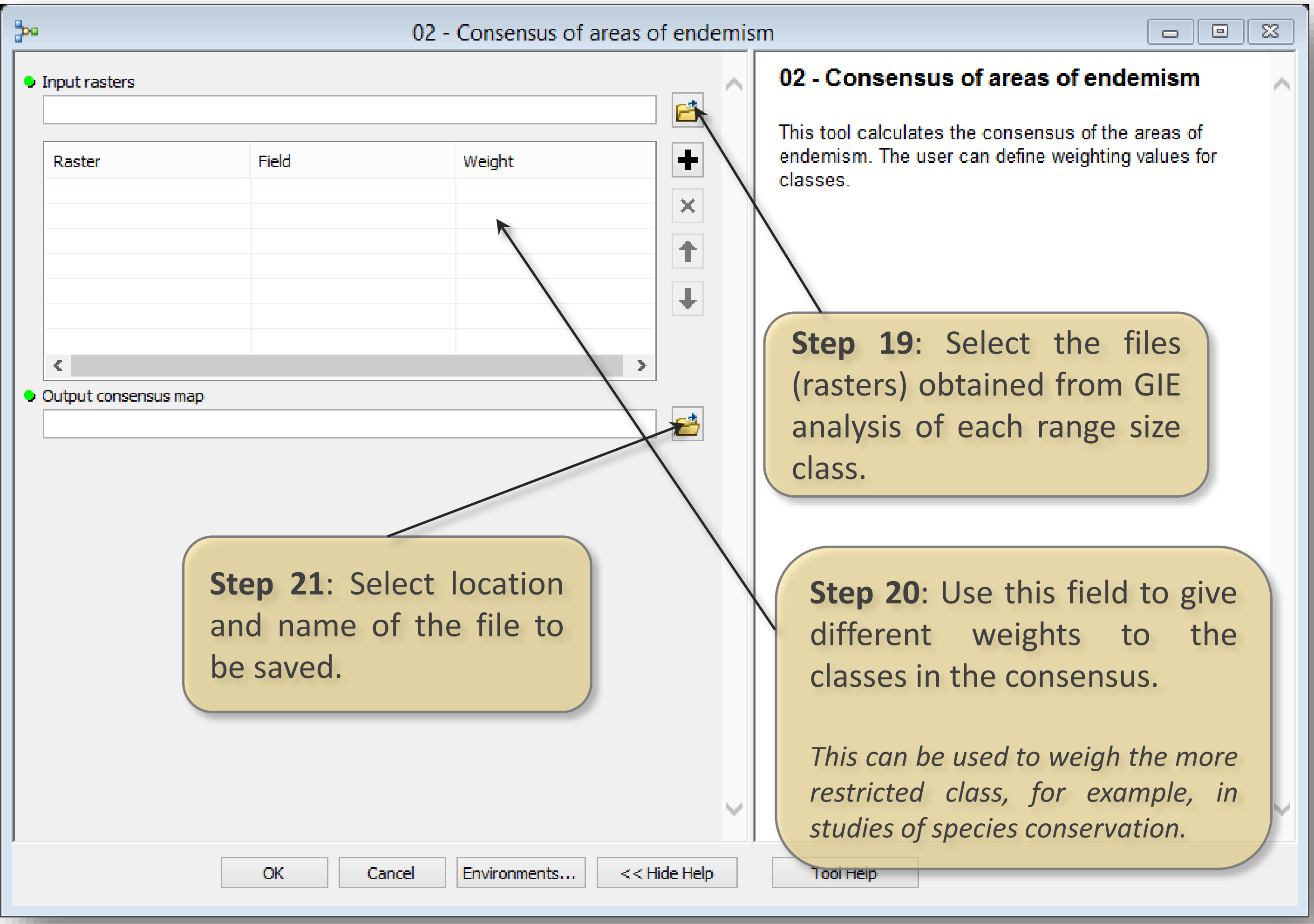

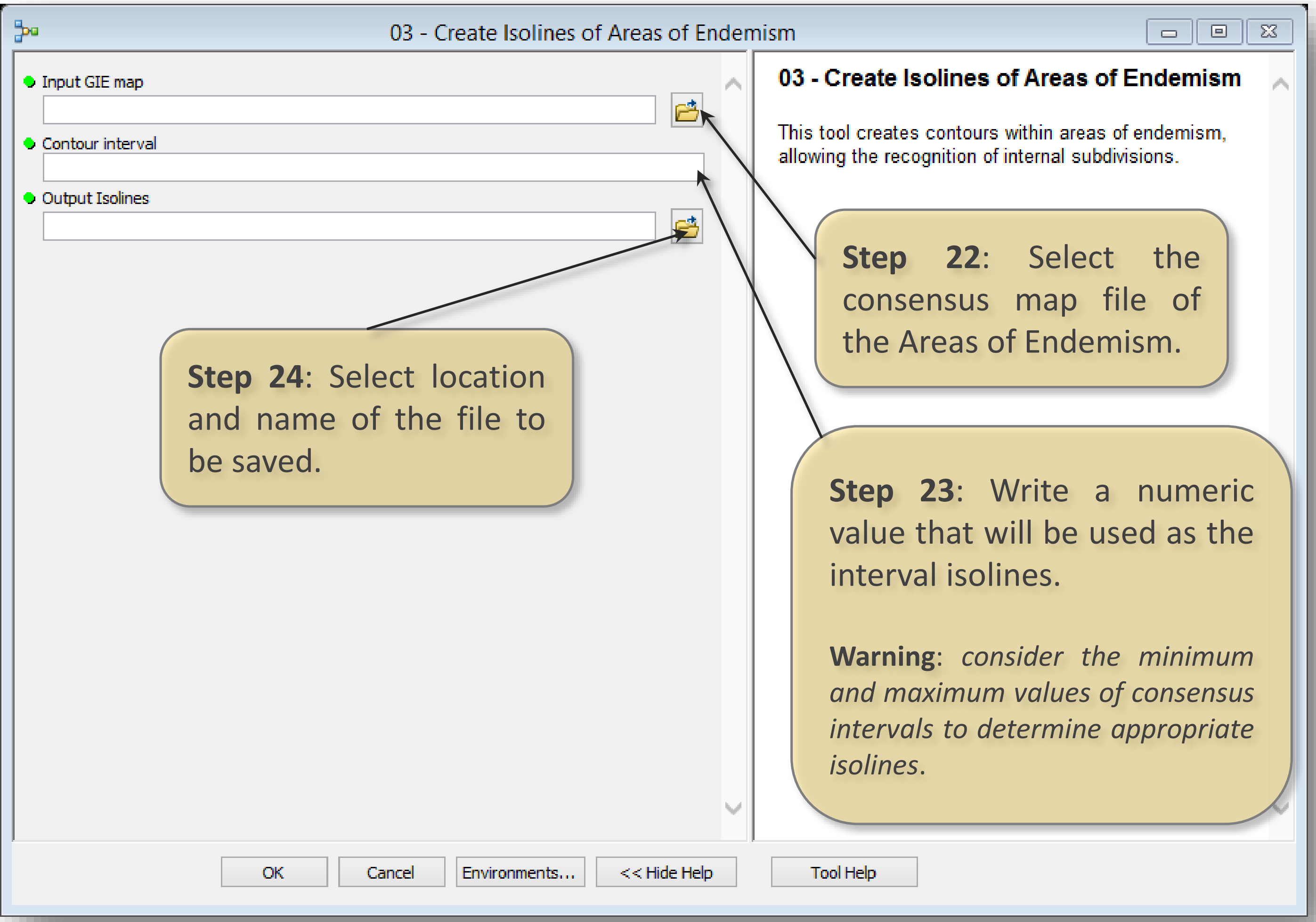

Results are displayed as distribution polygons on a map.

Identifying species with elongated distribution

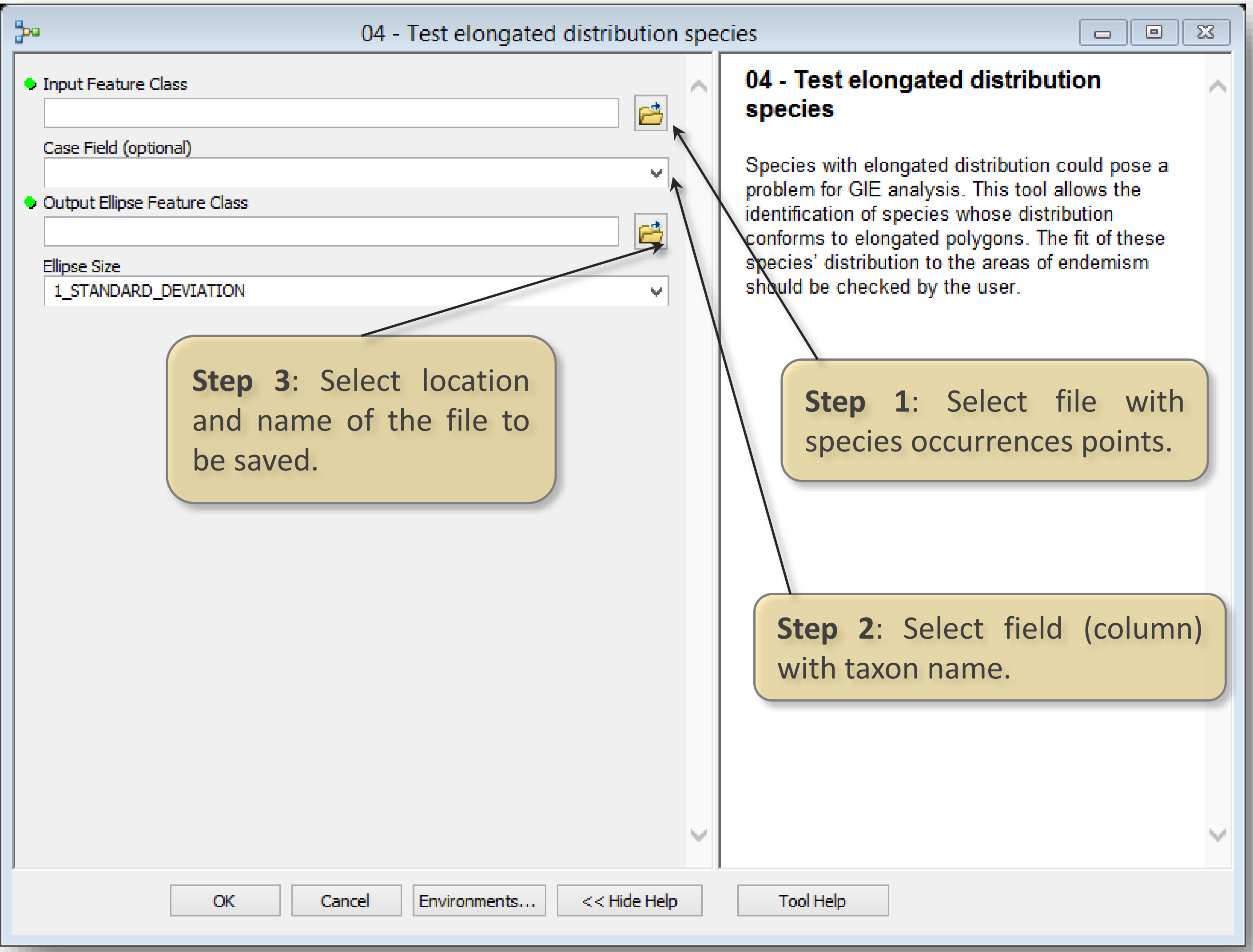

Supplement: S1 File — ArcGIS toolbox for Geographic interpolation of Endemism (GIE) and software “Geographic distance for GIE”, for calculation of distance between centroid and the farthest point of occurrence for each species. (ZIP) [file pone.0116673.s002.zip › 1/Tutorial.pdf]
