# Supplementary figures and images for: Delimiting Areas of Endemism through Kernel Interpolation
Source: PLoS One. 2015 Jan 22;10(1):e0116673. doi: 10.1371/journal.pone.0116673 (PMC4303434; doi:10.1371/journal.pone.0116673)

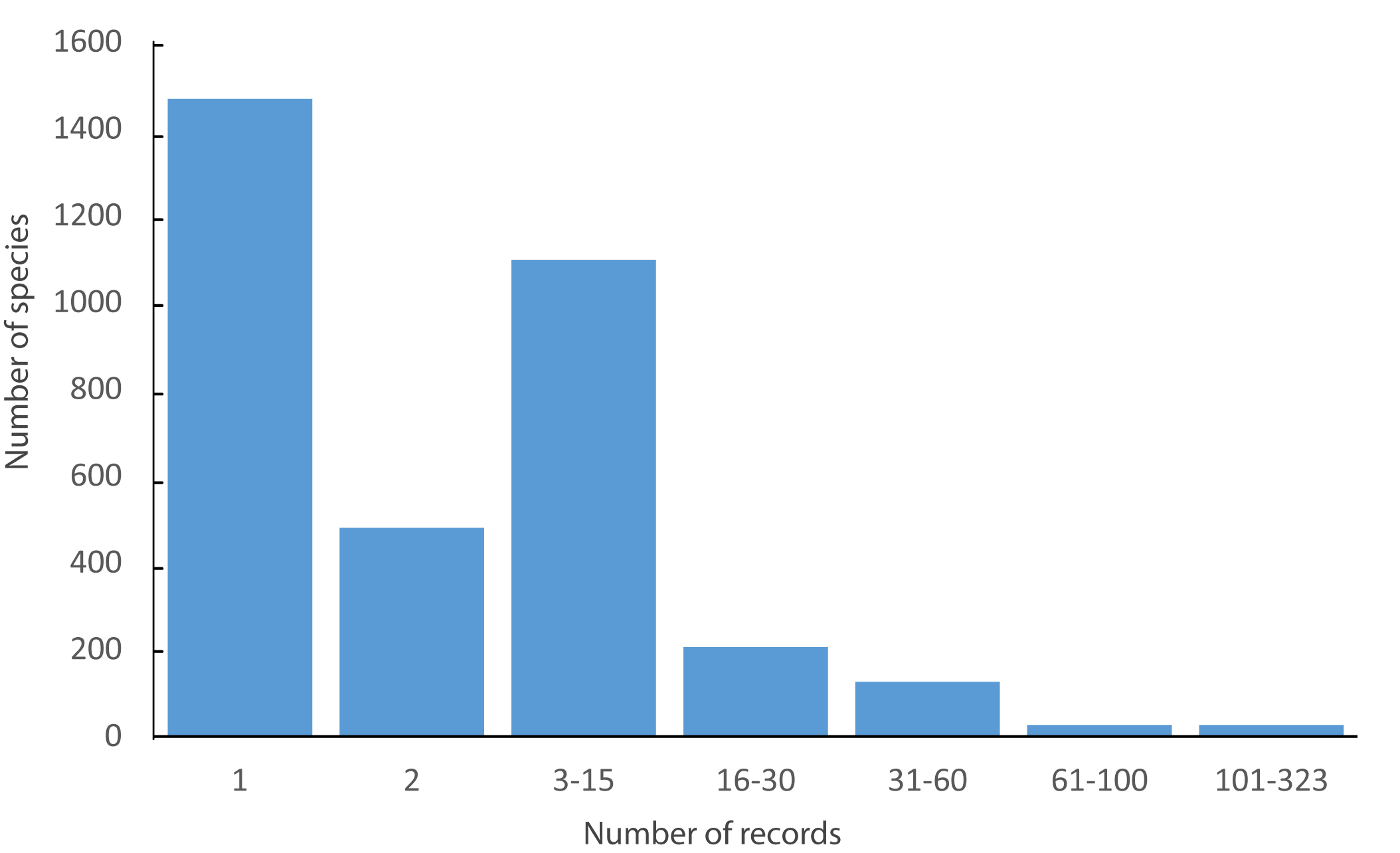

Supplement: S1 Fig — (TIF) [file pone.0116673.s005.tif]

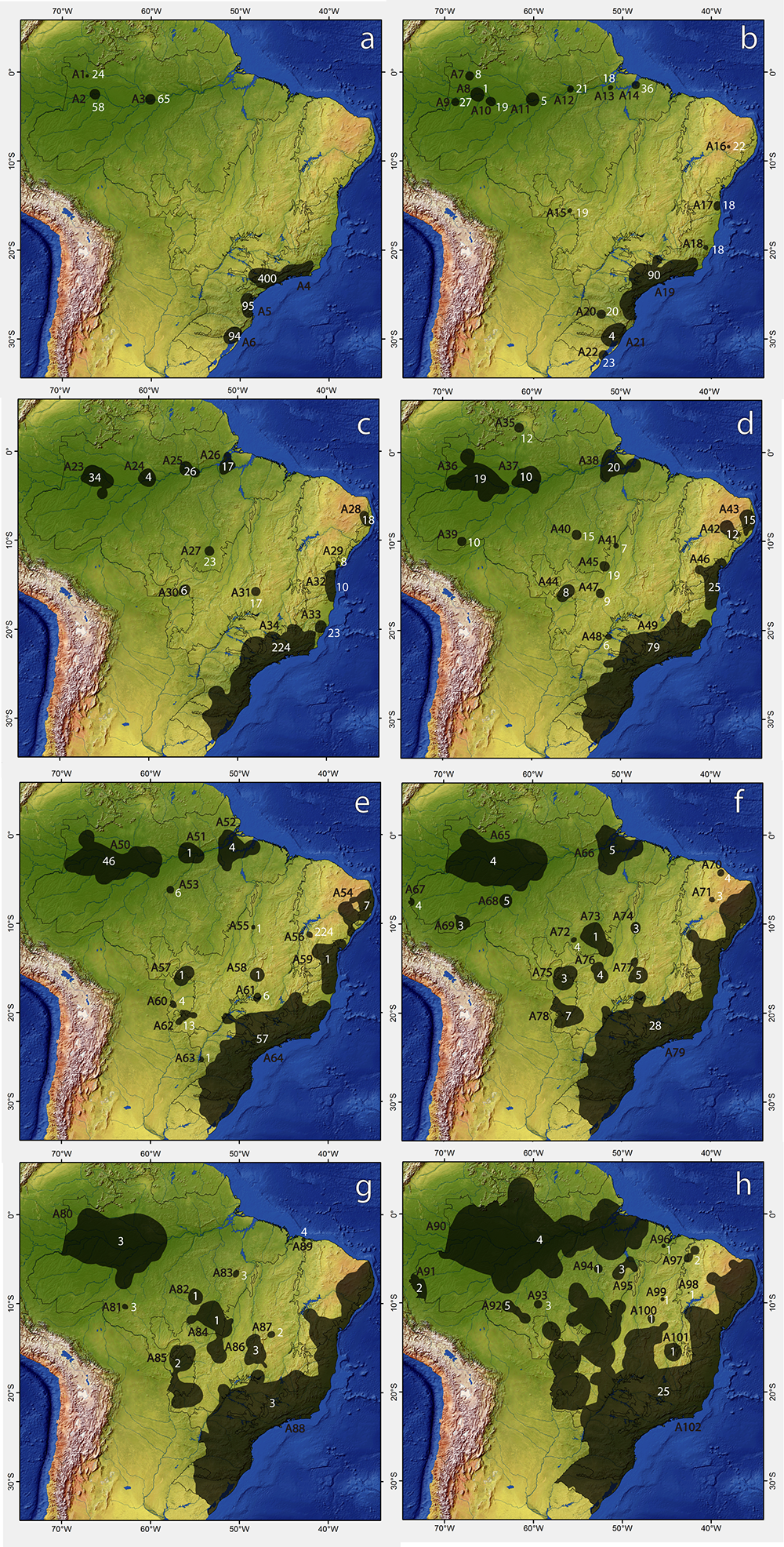

Supplement: S2 Fig — Text in black indicates the area code in S1 Table. White numbers indicate the number of endemic species in each area of endemism. (TIF) [file pone.0116673.s006.tif]

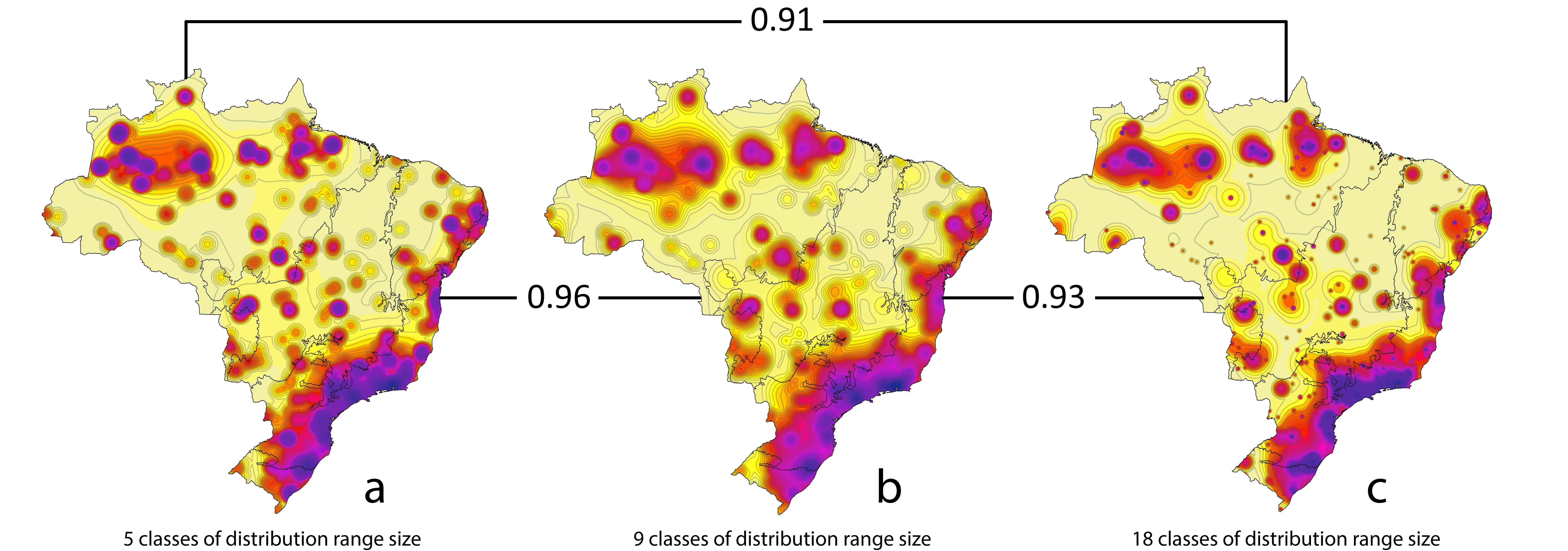

Supplement: S3 Fig — a: with five classes of range size; b: nine classes of range size and c: 18 classes of range size. Numbers indicates Pearson correlation (r) between maps. (TIF) [file pone.0116673.s007.tif]

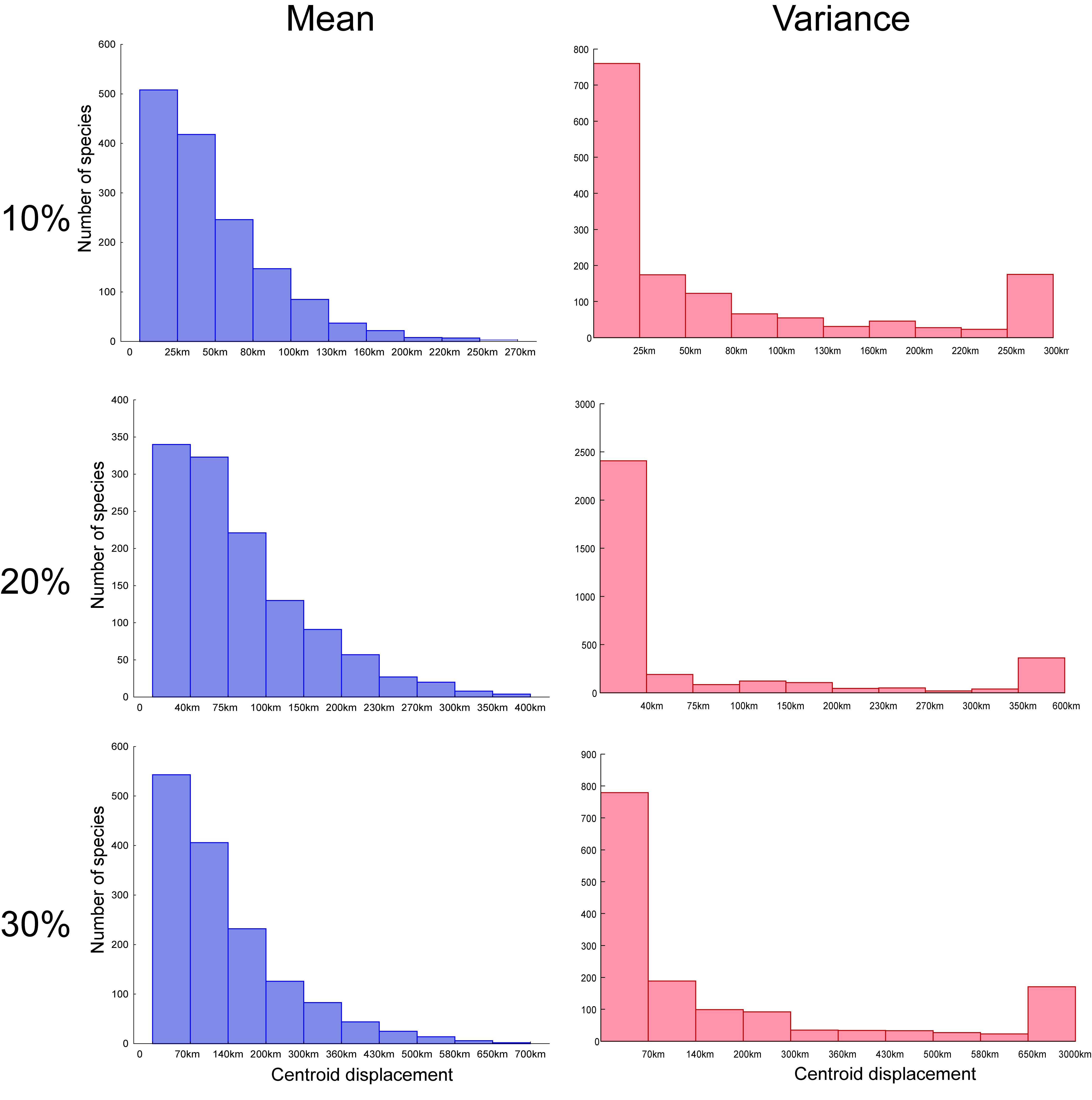

Supplement: S4 Fig — Graphs in the same line represent results of analyses removing 10, 20 and 30% of species records. Blue bars indicate frequency distribution of species in classes of average centroid displacement within 100 rarefaction replicates. Pink bars indicate frequency distribution of species in classes of variance of centroid displacement. (TIF) [file pone.0116673.s008.tif]

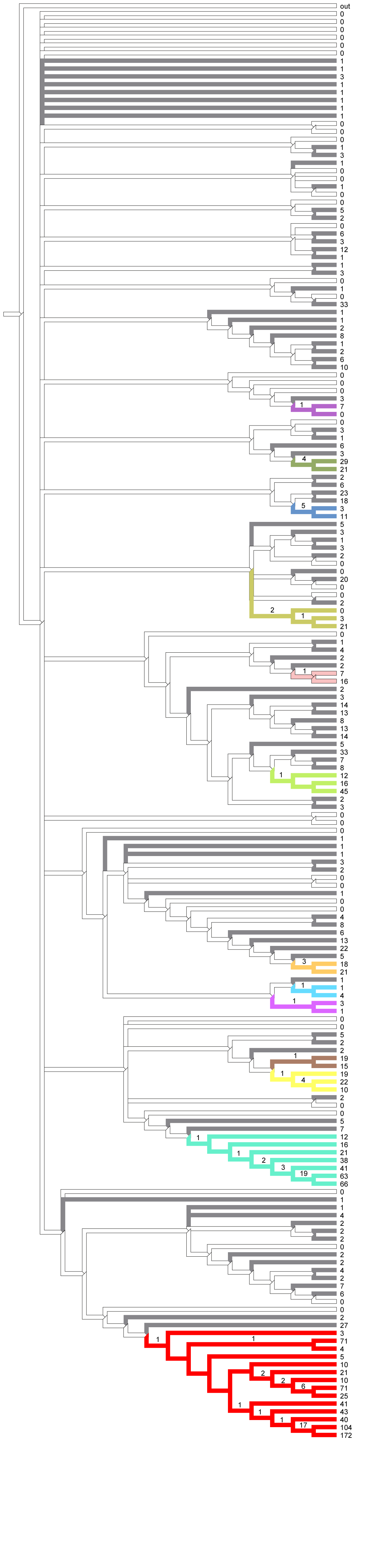

Supplement: S5 Fig — Numbers indicate endemic species within of grid cells (terminals) and groups of grid cells (branches). colours are the same as in Fig. 3. White branches correspond to terminals without exclusive species. (TIF) [file pone.0116673.s009.tif]

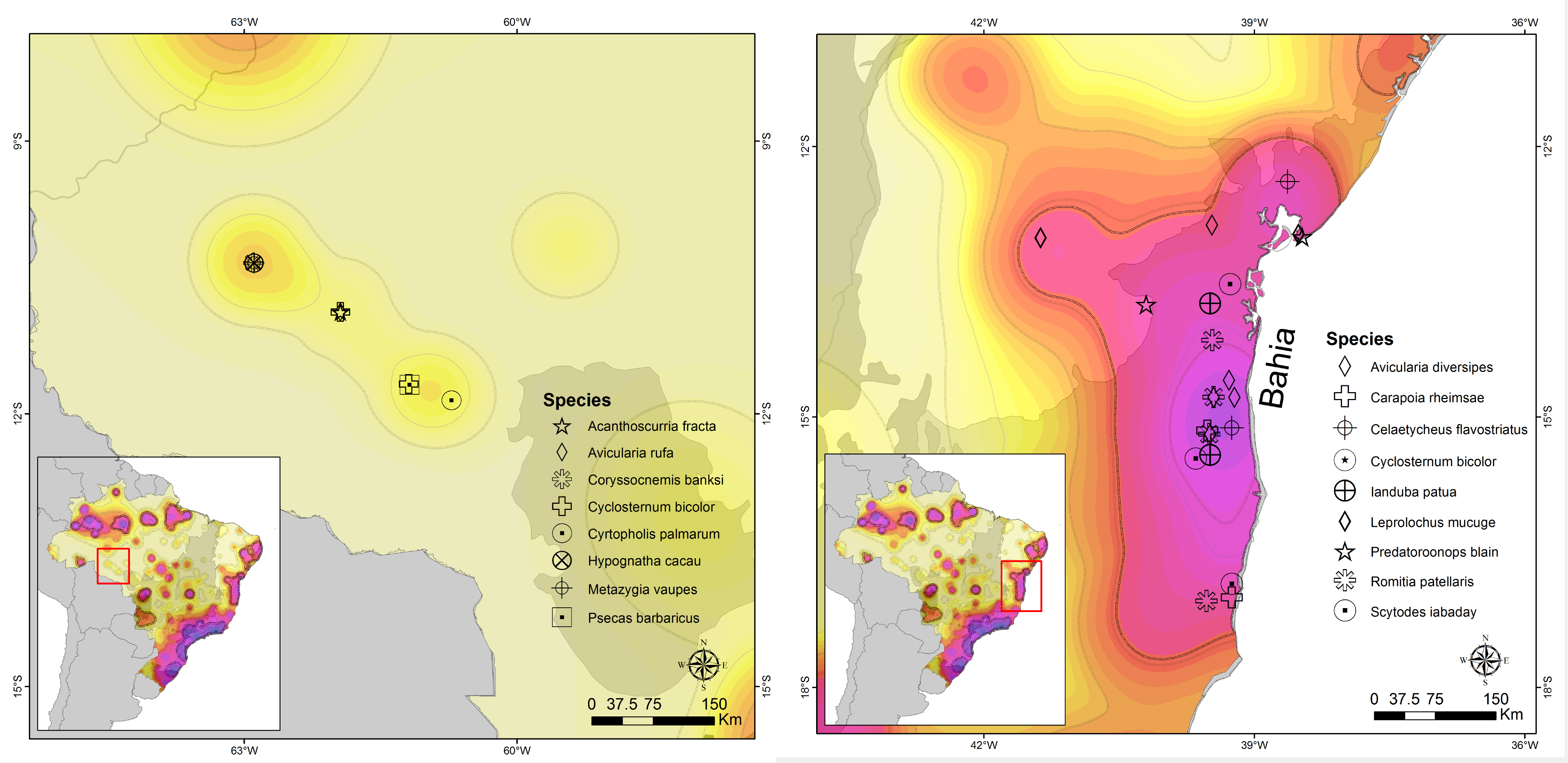

Supplement: S6 Fig — Elongated areas of endemism identified by GIE for spiders in Brazil. Species indicated by symbols present elongated distribution. (TIF) [file pone.0116673.s010.tif]
