# Supplementary material for: Delimiting Areas of Endemism through Kernel Interpolation
Source: PLoS One. 2015 Jan 22;10(1):e0116673. doi: 10.1371/journal.pone.0116673 (PMC4303434; doi:10.1371/journal.pone.0116673)
Supplement: S1 Table — Synendemic species of spiders supporting each area of endemism, as depicted in S2 Fig. (DOCX) [file pone.0116673.s011.docx]

| Area of endemism | Species |
| --- | --- |
| A1 | *Alpaida sandrei* |
| A1 | *Ceto setosa* |
| A1 | *Cetonana setosa* |
| A1 | *Chrysso gounellei* |
| A1 | *Gephyrina nigropunctata* |
| A1 | *Gnolus lemniscatus* |
| A1 | *Gnolus unipunctatus* |
| A1 | *Linothele bicolor* |
| A1 | *Lygarina nitida* |
| A1 | *Metadiaea fidelis* |
| A1 | *Nagaina diademata* |
| A1 | *Ocnotelus imberbis* |
| A1 | *Olbus gounellei* |
| A1 | *Oxysoma quinquenotatum* |
| A1 | *Sidymella obscura* |
| A1 | *Theridion aporum* |
| A1 | *Theridion umbilicus* |
| A1 | *Titidius dibius* |
| A1 | *Titidius marmoratus* |
| A1 | *Tmarus caretta* |
| A1 | *Tmarus lichenoides* |
| A1 | *Ulocymus sulcatus* |
| A1 | *Wulfilopsis martinsi* |
| A1 | *Zuniga laeta* |
| A2 | *Abapeba hoeferi* |
| A2 | *Acanthoctenus spiniger* |
| A2 | *Acragas castaneiceps* |
| A2 | *Alpaida acuta* |
| A2 | *Alpaida boa* |
| A2 | *Amazoromus becki* |
| A2 | *Amazoromus kedus* |
| A2 | *Amphidraus duckei* |
| A2 | *Anapis caluga* |
| A2 | *Anapisona schuhi* |
| A2 | *Anapistula secreta* |
| A2 | *Anyphaenoides coddingtoni* |
| A2 | *Chaetacis abrahami* |
| A2 | *Chaetacis cornuta* |
| A2 | *Chrosiothes venturosus* |
| A2 | *Chrysometa flavicans* |
| A2 | *Cryptachaea amazonas* |
| A2 | *Dipoena puertoricensis* |
| A2 | *Dolichognatha minuta* |
| A2 | *Echinotheridion lirum* |
| A2 | *Ecitocobius comissator* |
| A2 | *Eustiromastix falcatus* |
| A2 | *Faiditus analiae* |
| A2 | *Faiditus duckensis* |
| A2 | *Faiditus godmani* |
| A2 | *Hypaeus annulifer* |
| A2 | *Hypaeus luridomaculatus* |
| A2 | *Kupiuka extratheca* |
| A2 | *Mangora tarapuy* |
| A2 | *Metazygia ducke* |
| A2 | *Metazygia manu* |
| A2 | *Metazygia mariahelenae* |
| A2 | *Metazygia yucumo* |
| A2 | *Micrathena cucharas* |
| A2 | *Micrepeira tubulofaciens* |
| A2 | *Monoblemma becki* |
| A2 | *Myrmarachne sumana* |
| A2 | *Myrmecium bifasciatum* |
| A2 | *Nesticella ducke* |
| A2 | *Noegus coccineus* |
| A2 | *Ogulnius obtectus* |
| A2 | *Olios longipes* |
| A2 | *Onocolus granulosus* |
| A2 | *Scytodes paarmanni* |
| A2 | *Selenops ducke* |
| A2 | *Senoculus penicillatus* |
| A2 | *Simprulla nigricolor* |
| A2 | *Soesiladeepakius lyra* |
| A2 | *Speocera amazonica* |
| A2 | *Speocera molesta* |
| A2 | *Syntrechalea reimoseri* |
| A2 | *Tekellina crica* |
| A2 | *Testudinaria geometrica* |
| A2 | *Tobias pustulosus* |
| A2 | *Wagneriana bamba* |
| A2 | *Xyccarph myops* |
| A2 | *Xyccarph wellingtoni* |
| A2 | *Xylethrus scrupeus* |
| A3 | *Amazoromus cristus* |
| A3 | *Amazoromus janauari* |
| A3 | *Ancylometes riparius* |
| A3 | *Anelosimus domingo* |
| A3 | *Anyphaenoides xiboreninho* |
| A3 | *Avicularia bicegoi* |
| A3 | *Brasilionata arborense* |
| A3 | *Camillina taruma* |
| A3 | *Coxapopha bare* |
| A3 | *Coxapopha carinata* |
| A3 | *Dolichognatha erwini* |
| A3 | *Eilica amambay* |
| A3 | *Eilica marchantaria* |
| A3 | *Enna bartica* |
| A3 | *Envia garciai* |
| A3 | *Envia moleque* |
| A3 | *Ephebopus rufescens* |
| A3 | *Exechopsis eberhardi* |
| A3 | *Fufius albovittatus* |
| A3 | *Fufius striatipes* |
| A3 | *Gephyroctenus esteio* |
| A3 | *Gravipalpus crassus* |
| A3 | *Heidrunea arijana* |
| A3 | *Heidrunea irmleri* |
| A3 | *Heidrunea lobrita* |
| A3 | *Hypognatha janauari* |
| A3 | *Jollas amazonicus* |
| A3 | *Kaira sexta* |
| A3 | *Kalcerrytus salsicha* |
| A3 | *Kupiuka adisi* |
| A3 | *Kupiuka taruman* |
| A3 | *Lygromma taruma* |
| A3 | *Lyssomanes adisi* |
| A3 | *Lyssomanes janauari* |
| A3 | *Lyssomanes paravelox* |
| A3 | *Mangora morona* |
| A3 | *Masteria manaura* |
| A3 | *Meioneta adami* |
| A3 | *Metazygia adisi* |
| A3 | *Metazygia benella* |
| A3 | *Metazygia curari* |
| A3 | *Metazygia lopez* |
| A3 | *Ministigmata minuta* |
| A3 | *Nops nitidus* |
| A3 | *Oxyopes argyrotrichus* |
| A3 | *Oxyopes sexmaculatus* |
| A3 | *Plesiopiuka simplex* |
| A3 | *Rhoicinus gaujoni* |
| A3 | *Rhoicinus lugato* |
| A3 | *Scaphiella manaus* |
| A3 | *Schismatothele benedettiiß* |
| A3 | *Scytodes janauari* |
| A3 | *Senoculus cambridgei* |
| A3 | *Siloca campestrata* |
| A3 | *Soesilarishius ruizi* |
| A3 | *Stegodyphus manaus* |
| A3 | *Strophaeus austeni* |
| A3 | *Tekellina pretiosa* |
| A3 | *Thaumasia lisei* |
| A3 | *Tmarus digitatus* |
| A3 | *Trechalea lomalinda* |
| A3 | *Tricongius amazonicus* |
| A3 | *Xyccarph migrans* |
| A3 | *Zimiromus atrifus* |
| A3 | *Zimiromus boistus* |
| A4 | *Abapeba abalosi* |
| A4 | *Acanthoscurria aurita* |
| A4 | *Acanthoscurria pheopygus* |
| A4 | *Acentroscelus secundus* |
| A4 | *Achaearanea pallipes* |
| A4 | *Acracanthostoma bicornutum* |
| A4 | *Acragas nigromaculatus* |
| A4 | *Acragas trimaculatus* |
| A4 | *Alpaida alto* |
| A4 | *Alpaida orgaos* |
| A4 | *Alpaida sumare* |
| A4 | *Anelosimus baeza* |
| A4 | *Anelosimus dubiosus* |
| A4 | *Anelosimus jabaquara* |
| A4 | *Anelosimus tosum* |
| A4 | *Anodoration tantillum* |
| A4 | *Aphirape boliviensis* |
| A4 | *Apochinomma armatum* |
| A4 | *Apochinomma myrmecioides* |
| A4 | *Apochinomma pyriforme* |
| A4 | *Apopyllus isabelae* |
| A4 | *Arachnomura hieroglyphica* |
| A4 | *Arachnomura hyeroglyphica* |
| A4 | *Arachosia albiventris* |
| A4 | *Arachosia arachosia* |
| A4 | *Arachosia bergi* |
| A4 | *Arachosia freiburgensis* |
| A4 | *Arachosia mezenioides* |
| A4 | *Arachosia sulfurea* |
| A4 | *Arctosa humicola* |
| A4 | *Argyrodes longicaudatus* |
| A4 | *Argyrodes rigidus* |
| A4 | *Ariadna bicolor* |
| A4 | *Arochoides integrans* |
| A4 | *Atelurius segmentatus* |
| A4 | *Auximella spinosa* |
| A4 | *Avicularia gracilis* |
| A4 | *Aysha brasilisca* |
| A4 | *Aysha fortis* |
| A4 | *Aysha striolata* |
| A4 | *Balmaceda vera* |
| A4 | *Beata cinereonitida* |
| A4 | *Berlandiella insignis* |
| A4 | *Bertrana striolata* |
| A4 | *Bianor fasciatus* |
| A4 | *Caayguara poi* |
| A4 | *Camillina claro* |
| A4 | *Camillina minuta* |
| A4 | *Camillina pilar* |
| A4 | *Caponina tijuca* |
| A4 | *Castianeira albivulvae* |
| A4 | *Castianeira dubia* |
| A4 | *Castianeira luteipes* |
| A4 | *Castianeira maculata* |
| A4 | *Castianeira pyriformis* |
| A4 | *Castianeira sexmaculata* |
| A4 | *Castianeira virgulifera* |
| A4 | *Catumiri petrolium* |
| A4 | *Centroctenus sai* |
| A4 | *Chaetopelma gracile* |
| A4 | *Cheiracanthium montanum* |
| A4 | *Chirothecia botucatuensis* |
| A4 | *Chirothecia soaresi* |
| A4 | *Chirothecia uncata* |
| A4 | *Chrysometa jordao* |
| A4 | *Chrysometa raripila* |
| A4 | *Chrysometa sicki* |
| A4 | *Chrysometa sumare* |
| A4 | *Chrysso caraca* |
| A4 | *Chrysso pulchra* |
| A4 | *Ciniflella lutea* |
| A4 | *Cleocnemis serrana* |
| A4 | *Cleocnemis xenotypa* |
| A4 | *Coleosoma normale* |
| A4 | *Coreidon tropicum* |
| A4 | *Corinna alticeps* |
| A4 | *Corinna benefaciens* |
| A4 | *Corinna botucatensis* |
| A4 | *Corinna haemorrhoa* |
| A4 | *Corinna parva* |
| A4 | *Coryphasia monteverde* |
| A4 | *Coryphasia nuptialis* |
| A4 | *Coryssocnemis altiventer* |
| A4 | *Coryssocnemis discolor* |
| A4 | *Coryssocnemis lepidoptera* |
| A4 | *Coryssocnemis occulta* |
| A4 | *Coryssocnemis togata* |
| A4 | *Corythalia spiralis* |
| A4 | *Cotinusa albescens* |
| A4 | *Cotinusa magna* |
| A4 | *Cotinusa mathematica* |
| A4 | *Cotinusa pulchra* |
| A4 | *Cotinusa septempunctata* |
| A4 | *Cotinusa trimaculata* |
| A4 | *Cryptachaea eramus* |
| A4 | *Cryptachaea jequirituba* |
| A4 | *Cryptachaea pallipera* |
| A4 | *Cryptachaea sicki* |
| A4 | *Ctenus anahitiformis* |
| A4 | *Ctenus bulimus* |
| A4 | *Ctenus coxalis* |
| A4 | *Ctenus fernandae* |
| A4 | *Ctenus semiornatus* |
| A4 | *Ctenus serrichelis* |
| A4 | *Cyclosa oseret* |
| A4 | *Cyclosa punctata* |
| A4 | *Cylistella coccinelloides* |
| A4 | *Cylistella sanctipauli* |
| A4 | *Deinopis pallida* |
| A4 | *Deltoclita bioculata* |
| A4 | *Dendryphantes sexguttatus* |
| A4 | *Dictyna fluminensis* |
| A4 | *Diphya bicolor* |
| A4 | *Diplura annectens* |
| A4 | *Diplura borgmeyeri* |
| A4 | *Diplura taunayi* |
| A4 | *Dipoena cornuta* |
| A4 | *Dipoena flavomaculata* |
| A4 | *Dipoena niteroi* |
| A4 | *Dipoena obscura* |
| A4 | *Dossenus fluminensis* |
| A4 | *Dubiaranea argenteovittata* |
| A4 | *Dubiaranea decurtata* |
| A4 | *Echinotheridion andresito* |
| A4 | *Eilica maculipes* |
| A4 | *Enna trivittata* |
| A4 | *Epicadinus gavensis* |
| A4 | *Epicadinus polyophthalmus* |
| A4 | *Epicadus rubripes* |
| A4 | *Epicratinus petropolitanus* |
| A4 | *Episinus rio* |
| A4 | *Erissus bateae* |
| A4 | *Ero goeldii* |
| A4 | *Ero gracilis* |
| A4 | *Ero spinifrons* |
| A4 | *Ero valida* |
| A4 | *Eupalaestrus dubium* |
| A4 | *Euryopis notabilis* |
| A4 | *Eustala emertoni* |
| A4 | *Eustala vellardi* |
| A4 | *Eutichurus montanus* |
| A4 | *Exocora nogueirai* |
| A4 | *Faiditus solidao* |
| A4 | *Fufius lucasae* |
| A4 | *Gasteracantha versicolor* |
| A4 | *Gayenna alticola* |
| A4 | *Gayenna brasiliensis* |
| A4 | *Gayenna moreirae* |
| A4 | *Gayenna striata* |
| A4 | *Gayenna trivittata* |
| A4 | *Gelanor lanei* |
| A4 | *Geolycosa rufibarbis* |
| A4 | *Gephyrellula paulistana* |
| A4 | *Gephyrellula violacea* |
| A4 | *Goeldia nigra* |
| A4 | *Goeldia patellaris* |
| A4 | *Hahnia simoni* |
| A4 | *Hapalotremus cyclothorax* |
| A4 | *Heliconius antiochus* |
| A4 | *Helvibis longistyla* |
| A4 | *Helvibis monticola* |
| A4 | *Hentzia palmarum* |
| A4 | *Hermacha anomala* |
| A4 | *Hermacha fossor* |
| A4 | *Hermacha itatiaya* |
| A4 | *Hermachura leuderwaldti* |
| A4 | *Homoeomma hirsutum* |
| A4 | *Homoeomma montanum* |
| A4 | *Homoeomma strabo* |
| A4 | *Homoeomma stradlingi* |
| A4 | *Idiops germaini* |
| A4 | *Idiops montealegrensis* |
| A4 | *Ischnocolus rubropilosus* |
| A4 | *Isoctenus griseolus* |
| A4 | *Itatiaya apidema* |
| A4 | *Itatiaya iuba* |
| A4 | *Itatiaya ywyty* |
| A4 | *Jessica itatiaia* |
| A4 | *Lasiodora curtior* |
| A4 | *Lasiodora isabellina* |
| A4 | *Lasiodora sternalis* |
| A4 | *Leucauge argyrobapta* |
| A4 | *Leucauge roseosignata* |
| A4 | *Leucauge severa* |
| A4 | *Leucauge turbida* |
| A4 | *Leucauge uberta* |
| A4 | *Linothele gymnognatha* |
| A4 | *Linyphia armata* |
| A4 | *Linyphia clara* |
| A4 | *Linyphia cylindrata* |
| A4 | *Linyphia decorata* |
| A4 | *Linyphia leucosternum* |
| A4 | *Linyphia longiceps* |
| A4 | *Lizarba separata* |
| A4 | *Lycosa sericovittata* |
| A4 | *Lycosa tarantuloides* |
| A4 | *Lyssomanes devotoi* |
| A4 | *Lyssomanes leucomelas* |
| A4 | *Maeota dorsalis* |
| A4 | *Maeota flava* |
| A4 | *Magulla janeirus* |
| A4 | *Mastophora corumbatai* |
| A4 | *Mastophora felis* |
| A4 | *Mecynometa globosa* |
| A4 | *Mesabolivar azureus* |
| A4 | *Mesabolivar camussi* |
| A4 | *Mesabolivar cantharus* |
| A4 | *Mesabolivar cavicelatus* |
| A4 | *Mesabolivar fluminensis* |
| A4 | *Mesabolivar forceps* |
| A4 | *Mesabolivar levii* |
| A4 | *Mesabolivar mairyara* |
| A4 | *Metacleocnemis borgmeyeri* |
| A4 | *Metagonia bicornis* |
| A4 | *Metagonia heraldica* |
| A4 | *Metagonia paranapiacaba* |
| A4 | *Metagonia petropolis* |
| A4 | *Metagonia quadrifasciata* |
| A4 | *Metagonia unicolor* |
| A4 | *Metazygia barueri* |
| A4 | *Metazygia ipanga* |
| A4 | *Miagrammopes correai* |
| A4 | *Micrathena reimoseri* |
| A4 | *Micrathena teresopolis* |
| A4 | *Mimetus penicillaus* |
| A4 | *Misumenoides illotus* |
| A4 | *Misumenoides paucispinosus* |
| A4 | *Misumenops fluminensis* |
| A4 | *Misumenops lenis* |
| A4 | *Misumenops spinifer* |
| A4 | *Misumenops temibilis* |
| A4 | *Moyosi rugosa* |
| A4 | *Myrmarachne brasiliensis* |
| A4 | *Myrmecium itatiaiae* |
| A4 | *Myrmecium rufum* |
| A4 | *Mysmenopsis archeri* |
| A4 | *Naubolus sawayai* |
| A4 | *Naubolus tristis* |
| A4 | *Neodiplothele picta* |
| A4 | *Neohahnia ernsti* |
| A4 | *Neohahnia palmicola* |
| A4 | *Neohahnia sylviae* |
| A4 | *Neomaso arundicola* |
| A4 | *Neopisinus cognatus* |
| A4 | *Nesticus ramirezi* |
| A4 | *Notiohyphantes laudatus* |
| A4 | *Ochyrocera ibitipoca* |
| A4 | *Oligoctenus ornatus* |
| A4 | *Olios hieroglyphicus* |
| A4 | *Olios macroepigynum* |
| A4 | *Otiothops fulvus* |
| A4 | *Oxyopes fluminensis* |
| A4 | *Oxyopes m-fasciatus* |
| A4 | *Oxysoma bifasciatum* |
| A4 | *Oxysoma novum* |
| A4 | *Oxysoma polytrichium* |
| A4 | *Paracenobiopelma gerecormophila* |
| A4 | *Paradiestus aurantiacus* |
| A4 | *Paradiestus penicillatus* |
| A4 | *Parastrophius blanci* |
| A4 | *Paravulsor impudicus* |
| A4 | *Pensacolops rubrovittata* |
| A4 | *Phantyna mandibularis* |
| A4 | *Poecilochroa trifasciata* |
| A4 | *Predatoroonops peterhalli* |
| A4 | *Predatoroonops poncho* |
| A4 | *Predatoroonops vallarta* |
| A4 | *Procleocnemis concolor* |
| A4 | *Proctonemesia multicaudata* |
| A4 | *Pseudofluda pulcherrima* |
| A4 | *Psilochorus itaguyrussu* |
| A4 | *Psilochorus ybytyriguara* |
| A4 | *Pycnothele perdita* |
| A4 | *Pycnothele piracicabensis* |
| A4 | *Rachias aureus* |
| A4 | *Rachias brachytelus* |
| A4 | *Rachias caudatus* |
| A4 | *Rachias dispar* |
| A4 | *Rachias piracicabensis* |
| A4 | *Radulphius barueri* |
| A4 | *Radulphius bicolor* |
| A4 | *Radulphius lane* |
| A4 | *Radulphius latus* |
| A4 | *Radulphius monticola* |
| A4 | *Radulphius petropolis* |
| A4 | *Retiro lanceolatus* |
| A4 | *Retiro maculatus* |
| A4 | *Rudra polita* |
| A4 | *Salticus minax* |
| A4 | *Sanogasta puma* |
| A4 | *Sanogasta tenuis* |
| A4 | *Sarinoides violaceus* |
| A4 | *Sassacus aurantiacus* |
| A4 | *Sassacus glyphochela* |
| A4 | *Scopocira atypica* |
| A4 | *Scytodes bocaina* |
| A4 | *Scytodes genebra* |
| A4 | *Scytodes itapecerica* |
| A4 | *Scytodes maromba* |
| A4 | *Segestrioides badia* |
| A4 | *Selenops manzanoae* |
| A4 | *Semiopyla cataphracta* |
| A4 | *Senoculus fimbriatus* |
| A4 | *Senoculus incertus* |
| A4 | *Sidymella jordanensis* |
| A4 | *Sillus delicatus* |
| A4 | *Sillus pellucidus* |
| A4 | *Sphecozone melanocephala* |
| A4 | *Sphecozone tumidosa* |
| A4 | *Stenodeza fallax* |
| A4 | *Stenoteromata melloleitaoi* |
| A4 | *Stenoterommata leporina* |
| A4 | *Stephanopis borgmeyeri* |
| A4 | *Stephanopis macrostyla* |
| A4 | *Stephanopoides mirabilis* |
| A4 | *Strophius didacticus* |
| A4 | *Strophius melloleitaoi* |
| A4 | *Synema glaucothorax* |
| A4 | *Synemosyna scutata* |
| A4 | *Tariona mutica* |
| A4 | *Tasata fuscotaeniata* |
| A4 | *Tenedos garoa* |
| A4 | *Teudis concolor* |
| A4 | *Teudis griseus* |
| A4 | *Teudis itatiayae* |
| A4 | *Teudis lenis* |
| A4 | *Teudis opertaneus* |
| A4 | *Teudis parvulus* |
| A4 | *Teudis suspiciosus* |
| A4 | *Teudis tensus* |
| A4 | *Thaumasia marginella* |
| A4 | *Theridion apostoli* |
| A4 | *Theridion aulos* |
| A4 | *Theridion bellatulum* |
| A4 | *Theridion cochrum* |
| A4 | *Theridion evexum* |
| A4 | *Theridion nesticum* |
| A4 | *Theridion olaup* |
| A4 | *Theridion oswaldocruzi* |
| A4 | *Theridion pires* |
| A4 | *Theridion subrotundum* |
| A4 | *Theridion tungurahua* |
| A4 | *Theridion volubile* |
| A4 | *Thiodina candida* |
| A4 | *Thiodina punctulata* |
| A4 | *Thymoites ipiranga* |
| A4 | *Thymoites mirus* |
| A4 | *Thymoites palo* |
| A4 | *Tidarren sisyphoides* |
| A4 | *Titanattus pallidus* |
| A4 | *Titidiops melanosternus* |
| A4 | *Titidius albifrons* |
| A4 | *Titidius brasiliensis* |
| A4 | *Titidius difficilis* |
| A4 | *Titidius uncatus* |
| A4 | *Titiotus brasiliensis* |
| A4 | *Tmarus albifrons* |
| A4 | *Tmarus alticola* |
| A4 | *Tmarus atypicus* |
| A4 | *Tmarus bisectus* |
| A4 | *Tmarus borgmeyeri* |
| A4 | *Tmarus clavimanus* |
| A4 | *Tmarus formosus* |
| A4 | *Tmarus incognitus* |
| A4 | *Tmarus minensis* |
| A4 | *Tmarus mutabilis* |
| A4 | *Tmarus nigroviridis* |
| A4 | *Tmarus parki* |
| A4 | *Tmarus paulensis* |
| A4 | *Tmarus perditus* |
| A4 | *Tmarus pizai* |
| A4 | *Tmarus primitivus* |
| A4 | *Tmarus striolatus* |
| A4 | *Tobias gradiens* |
| A4 | *Toca bossanova* |
| A4 | *Tomopisthes horrendus* |
| A4 | *Trachelas vitiosus* |
| A4 | *Trechona adspersa* |
| A4 | *Trechona uniformis* |
| A4 | *Triaeris stenaspis* |
| A4 | *Tupigea altiventer* |
| A4 | *Tupigea cantareira* |
| A4 | *Tupigea guapia* |
| A4 | *Tupigea maza* |
| A4 | *Tupigea penedo* |
| A4 | *Tupigea sicki* |
| A4 | *Tupigea teresopolis* |
| A4 | *Uloborus ater* |
| A4 | *Uloborus niger* |
| A4 | *Umuara adfabilis* |
| A4 | *Valonia rubriventris* |
| A4 | *Vinnius calcarifer* |
| A4 | *Viracucha andicola* |
| A4 | *Vitalius cesteri* |
| A4 | *Vitalius tetracanthus* |
| A4 | *Vulsor occidentalis* |
| A4 | *Wedoquella punctata* |
| A4 | *Wirada tijuca* |
| A4 | *Xyccarph tenuis* |
| A4 | *Xylethrus ameda* |
| A5 | *Alpaida roemeri* |
| A5 | *Anapis hetschki* |
| A5 | *Araneus beebei* |
| A5 | *Argyrodes nephilae* |
| A5 | *Arocha rochai* |
| A5 | *Aysha brevimana* |
| A5 | *Aysha yacupoi* |
| A5 | *Baalzebub baubo* |
| A5 | *Cardimia eximia* |
| A5 | *Castianeira setosa* |
| A5 | *Castianeira vittata* |
| A5 | *Castianeira vittatula* |
| A5 | *Ceto lineolata* |
| A5 | *Cetonana lineolata* |
| A5 | *Chira penicillatus* |
| A5 | *Chthonos tuberosa* |
| A5 | *Coryphasia nigriventris* |
| A5 | *Cotinusa leucoprocta* |
| A5 | *Ctenus blumenauensis* |
| A5 | *Ctenus brevipes* |
| A5 | *Ctenus herteli* |
| A5 | *Ctenus mentor* |
| A5 | *Ctenus pauper* |
| A5 | *Ctenus sanctacatharinae* |
| A5 | *Epicadinus helenae* |
| A5 | *Epicadinus marmoratus* |
| A5 | *Escaphiella blumenau* |
| A5 | *Eustala mourei* |
| A5 | *Faiditus caronae* |
| A5 | *Faiditus morretensis* |
| A5 | *Gayenna tripunctata* |
| A5 | *Grammostola pulchripes* |
| A5 | *Hermacha iricolor* |
| A5 | *Hogna ornata* |
| A5 | *Isoctenus herteli* |
| A5 | *Labicymbium curitiba* |
| A5 | *Latonigena colombo* |
| A5 | *Leucauge polita* |
| A5 | *Mecicobothrium baccai* |
| A5 | *Mesabolivar embapua* |
| A5 | *Mesabolivar huberi* |
| A5 | *Miagrammopes luederwaldti* |
| A5 | *Microneta semiatra* |
| A5 | *Misumena terrosa* |
| A5 | *Misumenoides nigromaculatus* |
| A5 | *Misumenops paranensis* |
| A5 | *Mopiopia labyrinthea* |
| A5 | *Mopiopia tristis* |
| A5 | *Myrmecium camponotoides* |
| A5 | *Naubolus trifasciatus* |
| A5 | *Nesticus calilegua* |
| A5 | *Ochyrocera coerulea* |
| A5 | *Odo blumenauensis* |
| A5 | *Oecobius maculatus* |
| A5 | *Ogulnius clarus* |
| A5 | *Ogulnius obscurus* |
| A5 | *Opas paranensis* |
| A5 | *Opopaea concolor* |
| A5 | *Orfeo desolatus* |
| A5 | *Otiothops luteus* |
| A5 | *Oxyopes altifrons* |
| A5 | *Pardosa leprevosti* |
| A5 | *Pavocosa herteli* |
| A5 | *Pavocosa langei* |
| A5 | *Rhomphaea altissima* |
| A5 | *Schaenioscelis viridis* |
| A5 | *Scopocira fuscimana* |
| A5 | *Scytodes ilhota* |
| A5 | *Segestria ruficeps* |
| A5 | *Semora langei* |
| A5 | *Sidymella nigripes* |
| A5 | *Sphecozone modesta* |
| A5 | *Steatoda bipunctata* |
| A5 | *Synema bariguiensis* |
| A5 | *Synemosyna americana* |
| A5 | *Tasata tigris* |
| A5 | *Tenedos brescoviti* |
| A5 | *Tenedos hirsutus* |
| A5 | *Tenedos major* |
| A5 | *Tenedos minor* |
| A5 | *Teudis recentissimus* |
| A5 | *Theridion impegrum* |
| A5 | *Theridion pigrum* |
| A5 | *Theridion rubra* |
| A5 | *Theridula sexpupillata* |
| A5 | *Thymoites rarus* |
| A5 | *Titidius albiscriptus* |
| A5 | *Titidius curvilineatus* |
| A5 | *Tmarus candidissimmus* |
| A5 | *Tmarus misumenoides* |
| A5 | *Tmarus mourei* |
| A5 | *Tomopisthes frenatus* |
| A5 | *Trochosula luctuosa* |
| A5 | *Wendilgarda nigra* |
| A5 | *Zelotes paranaensis* |
| A6 | *Acanthogonatus ericae* |
| A6 | *Acanthogonatus tacuariensis* |
| A6 | *Achaearanea taim* |
| A6 | *Alpaida canela* |
| A6 | *Alpaida hartliebi* |
| A6 | *Anapisona bordeaux* |
| A6 | *Anokopsis avitoides* |
| A6 | *Arauchemus graudo* |
| A6 | *Arauchemus miudo* |
| A6 | *Arctosa sapiranga* |
| A6 | *Aysha chicama* |
| A6 | *Aysha guaiba* |
| A6 | *Catumiri argentinense* |
| A6 | *Ceratinopsis diversicolor* |
| A6 | *Chrysometa aramba* |
| A6 | *Cleocnemis taquarae* |
| A6 | *Cotinusa septenpunctata* |
| A6 | *Cryptachaea diamantina* |
| A6 | *Ctenus griseus* |
| A6 | *Cyrtopholis meridionalis* |
| A6 | *Deinopis rodophthalmus* |
| A6 | *Eilica pomposa* |
| A6 | *Erissus truncatifrons* |
| A6 | *Eupalaestrus weijenberghi* |
| A6 | *Eurymorion mourai* |
| A6 | *Euryopis spinifera* |
| A6 | *Fageia meridionalis* |
| A6 | *Gamasomorpha vianai* |
| A6 | *Gelanor mixtus* |
| A6 | *Hamataliwa constricta* |
| A6 | *Hasarius lisei* |
| A6 | *Hypognatha cambara* |
| A6 | *Jessica puava* |
| A6 | *Kaira erwini* |
| A6 | *Labicymbium otti* |
| A6 | *Lasiodora pantherina* |
| A6 | *Latonigena santana* |
| A6 | *Latonigena sapiranga* |
| A6 | *Losdolobus opytapora* |
| A6 | *Losdolobus ybypora* |
| A6 | *Lupettiana manauara* |
| A6 | *Magulla brescoviti* |
| A6 | *Mangora maximiano* |
| A6 | *Mangora paula* |
| A6 | *Mastophora extraordinaria* |
| A6 | *Mastophora pesqueiro* |
| A6 | *Metaltella simoni* |
| A6 | *Metepeira gressa* |
| A6 | *Mimetus hieroglyphicus* |
| A6 | *Misumena vatia* |
| A6 | *Misumenops maculisparsus* |
| A6 | *Myrmarachne parallela* |
| A6 | *Naatlo serrana* |
| A6 | *Odo serrimanus* |
| A6 | *Olios fasciatus* |
| A6 | *Opopaea viamao* |
| A6 | *Oxysoma itambezinho* |
| A6 | *Pescennina otti* |
| A6 | *Pikelinia arenicola* |
| A6 | *Platyarachne histrix* |
| A6 | *Polybetes pallidus* |
| A6 | *Pseudotyphistes biriva* |
| A6 | *Pseudotyphistes cambara* |
| A6 | *Pseudotyphistes vulpiscaudatus* |
| A6 | *Pycnothele auronitens* |
| A6 | *Radulphius cambara* |
| A6 | *Scolecura propinqua* |
| A6 | *Sitticus leucoproctus* |
| A6 | *Sphecozone nitens* |
| A6 | *Stenoteromata arnolisei* |
| A6 | *Stenoteromata curiy* |
| A6 | *Stenoteromata grimpa* |
| A6 | *Stenoterommata arnolisei* |
| A6 | *Stenoterommata curiy* |
| A6 | *Testudinaria elegans* |
| A6 | *Tetragnatha jaculator* |
| A6 | *Teuia beckeri* |
| A6 | *Theridion adjacens* |
| A6 | *Theridiosoma caaguara* |
| A6 | *Theriella bertoncello* |
| A6 | *Theriella bertoncelloi* |
| A6 | *Theriella galianoae* |
| A6 | *Thymoites melloleitaoi* |
| A6 | *Titanattus notabilis* |
| A6 | *Trachelopachys singularis* |
| A6 | *Tulpius gauchus* |
| A6 | *Tupigea paula* |
| A6 | *Uraarachne longa* |
| A6 | *Vesicapalpus serranus* |
| A6 | *Wediquela denticulata* |
| A6 | *Wirada araucaria* |
| A6 | *Wirada punctata* |
| A6 | *Wirada sigillata* |
| A6 | *Xenonemesia otti* |
| A7 | *Chrysometa candianii* |
| A7 | *Chrysometa lomanhungae* |
| A7 | *Chrysometa nubigena* |
| A7 | *Chrysometa petrasierwaldae* |
| A7 | *Chrysometa santosi* |
| A7 | *Chrysometa waikoxi* |
| A7 | *Chrysometa yanomami* |
| A7 | *Stethorrhagus archangelus* |
| A8 | *Ancylometes japura* |
| A9 | *Acentroscelus nigrianus* |
| A9 | *Aebutina binotata* |
| A9 | *Architis amazonica* |
| A9 | *Corinna aenea* |
| A9 | *Corinna annulipes* |
| A9 | *Corinna buccosa* |
| A9 | *Corinna eresiformis* |
| A9 | *Corinna galeata* |
| A9 | *Cyclosa olivenca* |
| A9 | *Cyrtognatha orphana* |
| A9 | *Hamataliwa bicolor* |
| A9 | *Hamataliwa biturberculata* |
| A9 | *Hamataliwa perdita* |
| A9 | *Hypaeus ignicomis* |
| A9 | *Kalcerrytus excultus* |
| A9 | *Nagaina tricincta* |
| A9 | *Noegus uncatus* |
| A9 | *Oxyopes pugilator* |
| A9 | *Pseudosparianthis picta* |
| A9 | *Strophaeus pentodon* |
| A9 | *Thammaca coriacea* |
| A9 | *Thiratoscirtus niveimanus* |
| A9 | *Titanattus pegaseus* |
| A9 | *Tmarus bifasciatus* |
| A9 | *Tmarus nigrofasciatus* |
| A9 | *Tmarus prognathus* |
| A9 | *Tobias pulcher* |
| A10 | *Acanthoscurria tarda* |
| A10 | *Amycus rufifrons* |
| A10 | *Audifia semigranosa* |
| A10 | *Chloridusa viridiaurea* |
| A10 | *Erissus spinosissimus* |
| A10 | *Hypaeus concinnus* |
| A10 | *Hypaeus nigrocomosus* |
| A10 | *Idiophthalma amazonica* |
| A10 | *Mangora argenteostriata* |
| A10 | *Misumenoides tibialis* |
| A10 | *Rhetenor diversipes* |
| A10 | *Sassacus arcuatus* |
| A10 | *Synema quadratum* |
| A10 | *Tmarus amazonicus* |
| A10 | *Tmarus caeruleus* |
| A10 | *Tmarus pallidus* |
| A10 | *Tylogonus prasinus* |
| A10 | *Tylogonus vachoni* |
| A10 | *Vindullus gracilipes* |
| A11 | *Amazonepeira manaus* |
| A11 | *Ariston mazolus* |
| A11 | *Mecoloesthus yawaperi* |
| A11 | *Xylethrus anomid* |
| A11 | *Zosis geniculata* |
| A12 | *Actinopus rufibarbis* |
| A12 | *Avicularia cuminami* |
| A12 | *Avicularia violacea* |
| A12 | *Castianeira isophthalma* |
| A12 | *Chira typica* |
| A12 | *Cyclosternum fasciatum* |
| A12 | *Elaver grandivulva* |
| A12 | *Enna paraensis* |
| A12 | *Hibana talmina* |
| A12 | *Hytanis oblonga* |
| A12 | *Itata partita* |
| A12 | *Leucauge funebris* |
| A12 | *Lurio conspicuus* |
| A12 | *Lycosa minima* |
| A12 | *Olios subadultus* |
| A12 | *Philoponella republicana* |
| A12 | *Rudra oriximina* |
| A12 | *Sampaiosia crulsi* |
| A12 | *Schaenioscelis exilis* |
| A12 | *Tapinillus roseisterni* |
| A12 | *Zelotes trimaculatus* |
| A13 | *Alpaida cuyabeno* |
| A13 | *Ami caxiuana* |
| A13 | *Attacobius blakei* |
| A13 | *Drymusa philomatica* |
| A13 | *Dubiepeira neptunina* |
| A13 | *Episinus vaticus* |
| A13 | *Eriophora ravilla* |
| A13 | *Helvibis rossi* |
| A13 | *Mangora ayo* |
| A13 | *Metazygia paquisha* |
| A13 | *Micrathena woytkowskii* |
| A13 | *Noegus franganilloi* |
| A13 | *Onofre necator* |
| A13 | *Pisaboa silvae* |
| A13 | *Scytodes cotopitoka* |
| A13 | *Scytodes piyampisi* |
| A13 | *Thymoites crassipes* |
| A13 | *Ummidia glabra* |
| A14 | *Abapeba echinus* |
| A14 | *Acanthogonatus fuegianus* |
| A14 | *Acentroscelus granulosus* |
| A14 | *Ameridion signaculum* |
| A14 | *Ameridion tempum* |
| A14 | *Ashtabula bicristata* |
| A14 | *Audifia laevithorax* |
| A14 | *Avicularia avicularia* |
| A14 | *Bellota peckhami* |
| A14 | *Bellota yacui* |
| A14 | *Brignolia parumpunctata* |
| A14 | *Chirothecia euchira* |
| A14 | *Chrysometa aureola* |
| A14 | *Episinus immundus* |
| A14 | *Euophrys ambigua* |
| A14 | *Fluda goianiae* |
| A14 | *Fluda inpae* |
| A14 | *Freya rubiginosa* |
| A14 | *Gravipalpus callosus* |
| A14 | *Idiops siolii* |
| A14 | *Lurio solennis* |
| A14 | *Lyssomanes sylvicola* |
| A14 | *Mago opiparis* |
| A14 | *Martella utingae* |
| A14 | *Metazygia goeldii* |
| A14 | *Noonops floridanus* |
| A14 | *Paraletes timidus* |
| A14 | *Rhomphaea velhaensis* |
| A14 | *Senoculus plumosus* |
| A14 | *Synemosyna paraensis* |
| A14 | *Taczanowskia trilobata* |
| A14 | *Testudinaria debsmithae* |
| A14 | *Theridion agrarium* |
| A14 | *Theridion paraense* |
| A14 | *Thymoites piarco* |
| A14 | *Tobias epicadoides* |
| A15 | *Acanthoscurria acuminata* |
| A15 | *Acanthoscurria rondoniae* |
| A15 | *Alpaida cuiaba* |
| A15 | *Aysha janaita* |
| A15 | *Beata pernix* |
| A15 | *Chapoda festiva* |
| A15 | *Corythalia fimbriata* |
| A15 | *Fluda narcissa* |
| A15 | *Longistylus ygapema* |
| A15 | *Martella maria* |
| A15 | *Metaphidippus albopilosus* |
| A15 | *Metaphidippus smithii* |
| A15 | *Otiothops platnicki* |
| A15 | *Otiothops typicus* |
| A15 | *Psecas chapoda* |
| A15 | *Sarinda armata* |
| A15 | *Scytodes akytaba* |
| A15 | *Synema spinosum* |
| A15 | *Synemosyna hentzi* |
| A16 | *Agalenoscosa pickeli* |
| A16 | *Aysha heraldica* |
| A16 | *Castianeira atypica* |
| A16 | *Castianeira leptopoda* |
| A16 | *Castianeira polyacantha* |
| A16 | *Ctenus taperae* |
| A16 | *Cyclosternum multicuspidatum* |
| A16 | *Gayenna taperae* |
| A16 | *Marpissa aureocomosus* |
| A16 | *Mastophora cranion* |
| A16 | *Mastophora gasteracanthoides* |
| A16 | *Mastophora pickeli* |
| A16 | *Misumenoides vulneratus* |
| A16 | *Misumenops biannulipes* |
| A16 | *Misumenops zeugma* |
| A16 | *Nothroctenus sericeus* |
| A16 | *Nothroctenus spinulosus* |
| A16 | *Phiale longibarba* |
| A16 | *Pseudoceto pickeli* |
| A16 | *Psilochorus taperae* |
| A16 | *Teudis dichotomus* |
| A16 | *Umuara lecochlorus* |
| A17 | *Acragas mendax* |
| A17 | *Alpaida almada* |
| A17 | *Alpaida santosi* |
| A17 | *Carapoia brescoviti* |
| A17 | *Carapoia una* |
| A17 | *Catumiri chicaoi* |
| A17 | *Celaetycheus mungunza* |
| A17 | *Ianduba caxixe* |
| A17 | *Ianduba vatapa* |
| A17 | *Lasiodora itabunae* |
| A17 | *Lyssomanes jucari* |
| A17 | *Metazygia atama* |
| A17 | *Metazygia patiama* |
| A17 | *Predatoroonops phillips* |
| A17 | *Selenops levii* |
| A17 | *Tmesiphantes amadoi* |
| A17 | *Tmesiphantes bethaniae* |
| A17 | *Wagneriana alma* |
| A18 | *Capidava saxatilis* |
| A18 | *Corythalia vervloeti* |
| A18 | *Dermochrosia maculatissima* |
| A18 | *Fissiscapus pusillus* |
| A18 | *Mesabolivar ceruleiventris* |
| A18 | *Metagonia furcata* |
| A18 | *Metagonia nadleri* |
| A18 | *Myrmecium aurantiacum* |
| A18 | *Noegus difficilis* |
| A18 | *Stephanopis ahenea* |
| A18 | *Stephanopis bella* |
| A18 | *Stephanopis colatinae* |
| A18 | *Tapinillus purpuratus* |
| A18 | *Thymoites iritus* |
| A18 | *Titidius dubitatus* |
| A18 | *Tmarus espiritosantense* |
| A18 | *Tmarus rarus* |
| A18 | *Viracucha silvicola* |
| A19 | *Acentroscelus muricatus* |
| A19 | *Alpaida rioja* |
| A19 | *Araneus lenkoi* |
| A19 | *Aysha garruchos* |
| A19 | *Beata sexmaculata* |
| A19 | *Camillina caldas* |
| A19 | *Castianeira carvalhoi* |
| A19 | *Chrysso intervales* |
| A19 | *Corinna selysi* |
| A19 | *Cotinusa gertschi* |
| A19 | *Cryptachaea canionis* |
| A19 | *Erica scutata* |
| A19 | *Fageia concolor* |
| A19 | *Gayenna bonneti* |
| A19 | *Hamataliwa albibarbis* |
| A19 | *Labicymbium rancho* |
| A19 | *Macutula santana* |
| A19 | *Mangora botelho* |
| A19 | *Mangora melloleitaoi* |
| A19 | *Mesabolivar guapiara* |
| A19 | *Mesabolivar samatiaguassu* |
| A19 | *Micrepeira albomaculata* |
| A19 | *Misumenoides rubroniger* |
| A19 | *Ocnotelus lunatus* |
| A19 | *Paradossenus isthmus* |
| A19 | *Parafluda albiana* |
| A19 | *Phiale rubroseriata* |
| A19 | *Phidippus tessellatus* |
| A19 | *Proctonemesia secunda* |
| A19 | *Psalistops nigrifemuratus* |
| A19 | *Radulphius boraceia* |
| A19 | *Radulphius caldas* |
| A19 | *Ramboia helenica* |
| A19 | *Romitia juqiuaensis* |
| A19 | *Rudra minensis* |
| A19 | *Soesilarishius albipes* |
| A19 | *Soesilarishius crispiventer* |
| A19 | *Synema haenschi* |
| A19 | *Synema interjectivum* |
| A19 | *Syntrechalea caballero* |
| A19 | *Tapinillus caldensis* |
| A19 | *Tariona albibarbis* |
| A19 | *Tetragnatha lactescensis* |
| A19 | *Thiodina setosa* |
| A19 | *Titidius pauper* |
| A19 | *Tmarus baptistai* |
| A19 | *Tmarus infrasigillatus* |
| A19 | *Trachelas tridentatus* |
| A19 | *Uloborus plumipes* |
| A19 | *Wendilgarda clara* |
| A19 | *Xiruana tetraseta* |
| A20 | *Anapisona platnicki* |
| A20 | *Anelosimus rabus* |
| A20 | *Argyrodes modestus* |
| A20 | *Dipoena opana* |
| A20 | *Guaraniella brancata* |
| A20 | *Harmiella schiapelliae* |
| A20 | *Holothele recta* |
| A20 | *Metazygia uratron* |
| A20 | *Metazygia valentim* |
| A20 | *Ochyrocera viridissima* |
| A20 | *Opopaea ita* |
| A20 | *Simprulla argentina* |
| A20 | *Speocera jucunda* |
| A20 | *Sphecozone novaeteutoniae* |
| A20 | *Teutoniella plaumanni* |
| A20 | *Theridion eremum* |
| A20 | *Thwaitesia splendida* |
| A20 | *Thymoites aloitus* |
| A20 | *Thymoites ebus* |
| A20 | *Trechalea langei* |
| A21 | *Dolichognatha proserpina* |
| A22 | *Anelosimus elegans* |
| A22 | *Ariadna boesenbergi* |
| A22 | *Catumiri parvum* |
| A22 | *Catumiri uruguayense* |
| A22 | *Ctenus minusculus* |
| A22 | *Diapontia uruguayensis* |
| A22 | *Dingosa venefica* |
| A22 | *Eilica obscura* |
| A22 | *Euophrys flordellago* |
| A22 | *Gayenna praesignis* |
| A22 | *Gayenna x-signata* |
| A22 | *Hogna nervosa* |
| A22 | *Hogna volxemi* |
| A22 | *Laminacauda ignobilis* |
| A22 | *Lycosa nervosa* |
| A22 | *Meriola foraminosa* |
| A22 | *Metaltella iheringi* |
| A22 | *Paradiestus vitiosus* |
| A22 | *Pardosa rara* |
| A22 | *Steatoda iheringii* |
| A22 | *Synema luteovittatum* |
| A22 | *Trachelas flavipes* |
| A22 | *Trechalea longitarsis* |
| A23 | *Anapis aragua* |
| A23 | *Cryptachaea maldonado* |
| A23 | *Cryptachaea uviana* |
| A23 | *Dubiepeira lamolina* |
| A23 | *Dysderina urucu* |
| A23 | *Episinus juarezi* |
| A23 | *Faiditus convolutus* |
| A23 | *Gelanor mabelae* |
| A23 | *Goeldia arnozoi* |
| A23 | *Ischnothyreus velox* |
| A23 | *Linothele fallax* |
| A23 | *Mangora barba* |
| A23 | *Mangora moyobamba* |
| A23 | *Metagonia ocaina* |
| A23 | *Metazygia sendero* |
| A23 | *Metazygia tapa* |
| A23 | *Neohahnia ernesti* |
| A23 | *Olios keyserlingi* |
| A23 | *Paratropis sanguinea* |
| A23 | *Scytodes iara* |
| A23 | *Sericopelma fallax* |
| A23 | *Soesiladeepakius arthrostylus* |
| A23 | *Soesiladeepakius retroversus* |
| A23 | *Soesiladeepakius uncinatus* |
| A23 | *Wagneriana silvae* |
| A23 | *Xeropigo perene* |
| A24 | *Ancylometes jau* |
| A24 | *Cyriocosmus butantan* |
| A25 | *Actinopus wallacei* |
| A25 | *Avicularia braunshauseni* |
| A25 | *Caponina papamanga* |
| A25 | *Ceriomura cruenta* |
| A25 | *Ctenus albofasciatus* |
| A25 | *Ctenus serratipes* |
| A25 | *Descanso vagus* |
| A25 | *Drymusa canhemabae* |
| A25 | *Drymusa colligata* |
| A25 | *Gephyroctenus juruti* |
| A25 | *Harmonicon rufescens* |
| A25 | *Idiops santaremius* |
| A25 | *Marpissa agricola* |
| A25 | *Paratropis papilligera* |
| A25 | *Platycryptus magnus* |
| A25 | *Pseudicius oblongus* |
| A25 | *Rudra tenera* |
| A25 | *Scaphiella irmaos* |
| A25 | *Selenops para* |
| A25 | *Soesiladeepakius biarmatus* |
| A25 | *Strophius signatus* |
| A25 | *Tacuna vaga* |
| A25 | *Teudis peragrans* |
| A25 | *Tmarus trifidus* |
| A25 | *Trochosa sericea* |
| A26 | *Alpaida gurupi* |
| A26 | *Anisaspoides gigantea* |
| A26 | *Chaetacis woytkowskii* |
| A26 | *Fluda usta* |
| A26 | *Litoporus uncatus* |
| A26 | *Mesabolivar paraensis* |
| A26 | *Metazygia aldela* |
| A26 | *Micrathena gurupi* |
| A26 | *Otiothops baculus* |
| A26 | *Otiothops curua* |
| A26 | *Otiothops facis* |
| A26 | *Otiothops puraquequara* |
| A26 | *Symphytognatha tacaca* |
| A26 | *Titidius gurupi* |
| A27 | *Acacesia foliata* |
| A27 | *Actinopus echinus* |
| A27 | *Cleocnemis punctulata* |
| A27 | *Erissoides vitttatus* |
| A27 | *Eustala anastera* |
| A27 | *Helvetia santarema* |
| A27 | *Miagrammopes rubripes* |
| A27 | *Misumenoides roseiceps* |
| A27 | *Misumenops haemorrhous* |
| A27 | *Misumenops longispinosus* |
| A27 | *Noegus trilineatus* |
| A27 | *Oxyopes inversus* |
| A27 | *Oxyopes taeniatulus* |
| A27 | *Pardosa albopilosa* |
| A27 | *Polybetes obnuptus* |
| A27 | *Simonurius campestratus* |
| A27 | *Stasina koluene* |
| A27 | *Tetragnatha elongata* |
| A27 | *Thiodina pallida* |
| A27 | *Thiodina vellardi* |
| A27 | *Titidius rubrosignatus* |
| A27 | *Tmarus villasboasi* |
| A27 | *Wagneriana vermiculata* |
| A28 | *Actinopus princeps* |
| A28 | *Avicularia hirsuta* |
| A28 | *Cleocnemis nigra* |
| A28 | *Cleocnemis rudolphi* |
| A28 | *Gephyrina albimarginata* |
| A28 | *Kambiwa anomala* |
| A28 | *Leprolochus parahybae* |
| A28 | *Litoporus agricola* |
| A28 | *Macutula aracoiaba* |
| A28 | *Neodiplothele irregularis* |
| A28 | *Plesiopelma physopus* |
| A28 | *Polybetes proximus* |
| A28 | *Psilochorus sectus* |
| A28 | *Scytodes maresi* |
| A28 | *Scytodes tapacura* |
| A28 | *Trachelopachys sericeus* |
| A28 | *Typhochlaena curumim* |
| A28 | *Viracucha exilis* |
| A29 | *Apochinomma constrictum* |
| A29 | *Cosmopelma decoratum* |
| A29 | *Lyssomanes parallelus* |
| A29 | *Misumena luteovariata* |
| A29 | *Sidymella parallela* |
| A29 | *Stephanopis salobrensis* |
| A29 | *Synema setiferum* |
| A29 | *Ulocymus intermedius* |
| A30 | *Chira guianensis* |
| A30 | *Leprolochus levergere* |
| A30 | *Lyssomanes hieroglyphicus* |
| A30 | *Mangora antonio* |
| A30 | *Mangora leverger* |
| A30 | *Theridion fungosum* |
| A31 | *Castianeira cyclindracea* |
| A31 | *Castianeira rutilans* |
| A31 | *Chaco melloleitaoi* |
| A31 | *Corinna inermis* |
| A31 | *Homoeomma familiare* |
| A31 | *Lasiodora fallax* |
| A31 | *Leptopholcus brazlandia* |
| A31 | *Noegus niveogularis* |
| A31 | *Olios attractus* |
| A31 | *Olios mutabilis* |
| A31 | *Otiothops recurvus* |
| A31 | *Schaenioscelis elegans* |
| A31 | *Selenops ximenae* |
| A31 | *Stasina americana* |
| A31 | *Tenedos eduardoi* |
| A31 | *Tetragnatha boydi* |
| A31 | *Tmarus nigriscens* |
| A32 | *Celaetycheus caruru* |
| A32 | *Celaetycheus vatapa* |
| A32 | *Ianduba abara* |
| A32 | *Ianduba mugunza* |
| A32 | *Lycinus portoseguro* |
| A32 | *Melychiopharis bibendum* |
| A32 | *Predatoroonops dutch* |
| A33 | *Predatoroonops chicano* |
| A33 | *Stephanopoides sanctaeleopoldinae* |
| A33 | *Trogloneta cariacica* |
| A33 | *Typhochlaena amma* |
| A34 | *Acanthoscurria cunhae* |
| A34 | *Acanthoscurria pugnax* |
| A34 | *Alcimosphenus rubripleurus* |
| A34 | *Alpaida itacolomi* |
| A34 | *Alpaida tonze* |
| A34 | *Alpaida yotoco* |
| A34 | *Anelosimus inhandava* |
| A34 | *Anelosimus sumisolena* |
| A34 | *Arachnothermes termitophilus* |
| A34 | *Arachosia minensis* |
| A34 | *Argyrodes weyrauchi* |
| A34 | *Auximella minensis* |
| A34 | *Castianeira minensis* |
| A34 | *Coryphasia castaneipeidis* |
| A34 | *Cryptachaea vivida* |
| A34 | *Ctenus forcipatus* |
| A34 | *Ctenus griseolus* |
| A34 | *Ctenus lagesicola* |
| A34 | *Cyrtognatha eberhardi* |
| A34 | *Deinopis armaticeps* |
| A34 | *Dubiaranea levii* |
| A34 | *Eilica trilineata* |
| A34 | *Epicadus pallidus* |
| A34 | *Episinus pyrus* |
| A34 | *Fernandezina divisa* |
| A34 | *Fernandezina pelta* |
| A34 | *Grammostola mollicoma* |
| A34 | *Helvetia labiata* |
| A34 | *Itatiaya pucupucu* |
| A34 | *Itatiaya pykyyra* |
| A34 | *Laminacauda montevidensis* |
| A34 | *Lasiodora differens* |
| A34 | *Lasiodora dulcicola* |
| A34 | *Lasiodora mariannae* |
| A34 | *Lasiodora subcanens* |
| A34 | *Leucauge venusta* |
| A34 | *Loxosceles immodesta* |
| A34 | *Lyssomanes fossor* |
| A34 | *Mago fonsecai* |
| A34 | *Mangora ouropreto* |
| A34 | *Mastophora brescoviti* |
| A34 | *Matta hambletoni* |
| A34 | *Mesabolivar uruguayensis* |
| A34 | *Metazygia amalla* |
| A34 | *Metazygia cunha* |
| A34 | *Misumenops variegatus* |
| A34 | *Nesticus taim* |
| A34 | *Olios minensis* |
| A34 | *Orfeo jobim* |
| A34 | *Philoponella ramirezi* |
| A34 | *Plesiopelma minensis* |
| A34 | *Predatoroonops rickhawkins* |
| A34 | *Predatoroonops yautja* |
| A34 | *Prusias brasiliensis* |
| A34 | *Radulphius caparao* |
| A34 | *Steatoda iheringi* |
| A34 | *Tenedos reygeli* |
| A34 | *Teudis bicornutus* |
| A34 | *Titidius haemorrhous* |
| A34 | *Titidius longicaudatus* |
| A34 | *Tmarus ampullatus* |
| A34 | *Tmarus nigridorsi* |
| A34 | *Trochanteria ranuncula* |
| A34 | *Uloborus minutus* |
| A34 | *Xenonemesia platensis* |
| A35 | *Achaearanea dalana* |
| A35 | *Cryptachaea maraca* |
| A35 | *Echinotheridion urarum* |
| A35 | *Episinus bruneoviridis* |
| A35 | *Episinus crysus* |
| A35 | *Episinus garisus* |
| A35 | *Hingstepeira arnolisei* |
| A35 | *Kaira shinguito* |
| A35 | *Metazygia souza* |
| A35 | *Stemmops orsus* |
| A35 | *Thymoites cravilus* |
| A35 | *Zimiromus racamus* |
| A36 | *Enna bonaldoi* |
| A36 | *Titidius urucu* |
| A36 | *Tobias inermis* |
| A37 | *Cryptachaea inops* |
| A37 | *Dolichognatha mapia* |
| A37 | *Gephyroctenus mapia* |
| A37 | *Ischnothele caudata* |
| A37 | *Mangora mapia* |
| A37 | *Maraca cabocla* |
| A37 | *Metagonia mariguitarensis* |
| A37 | *Misumenoides magnus* |
| A37 | *Syntrechalea neblina* |
| A38 | *Amphidraus santanae* |
| A38 | *Avicularia geroldi* |
| A38 | *Avitus anumbi* |
| A38 | *Cerionesta aurantia* |
| A38 | *Eustala pallida* |
| A38 | *Kalcerrytus nauticus* |
| A38 | *Leucauge henryi* |
| A38 | *Martella amapa* |
| A38 | *Noegus niveomarginatus* |
| A38 | *Onofre carnifex* |
| A38 | *Opas trilineata* |
| A38 | *Physocyclus viridis* |
| A38 | *Pseudosparianthis fusca* |
| A38 | *Tmarus hirsutus* |
| A38 | *Uloborus tetramaculatus* |
| A38 | *Vindullus concavus* |
| A39 | *Architis catuaba* |
| A39 | *Architis gracilis* |
| A39 | *Cyriocosmus bertae* |
| A39 | *Cyriocosmus nogueira-netoi* |
| A39 | *Cyriocosmus ritae* |
| A39 | *Ericaella kaxinawa* |
| A39 | *Jessica rafaeli* |
| A39 | *Metepeira roraima* |
| A39 | *Misionella jaminawa* |
| A39 | *Syntrechalea napoensis* |
| A40 | *Alijassa venezuelica* |
| A40 | *Amatorculus cristinae* |
| A40 | *Breda paraensis* |
| A40 | *Capeta cachimbo* |
| A40 | *Dipoena esra* |
| A40 | *Dipoena trinidensis* |
| A40 | *Escaphiella cachimbo* |
| A40 | *Freya rufohirta* |
| A40 | *Metazygia loque* |
| A40 | *Neopisinus bigibbosus* |
| A40 | *Neoxyphinus boibumba* |
| A40 | *Patrera armata* |
| A40 | *Spilasma utaca* |
| A40 | *Stemmops subtilis* |
| A40 | *Theridion metabolum* |
| A41 | *Alpaida morro* |
| A41 | *Lyssomanes tapirapensis* |
| A41 | *Ochyrocera cornuta* |
| A41 | *Opas caudata* |
| A41 | *Opas melanoleuca* |
| A41 | *Plesiopelma semiaurantiacum* |
| A41 | *Tmarus aberrans* |
| A42 | *Deinopis fastigata* |
| A42 | *Deinopis seriata* |
| A42 | *Gavarilla ianuzziae* |
| A42 | *Mecynogea buique* |
| A42 | *Misumenoides fusciventris* |
| A42 | *Neonella salafraria* |
| A42 | *Nosferattus palmatus* |
| A42 | *Senoculus proximus* |
| A42 | *Stephanopis renipalpis* |
| A42 | *Tmarus obesus* |
| A42 | *Tobias albicans* |
| A42 | *Zozis costalimae* |
| A43 | *Erissus roseus* |
| A43 | *Eurymorion murici* |
| A43 | *Hapalotremus scintillans* |
| A43 | *Kupiuka murici* |
| A43 | *Macutula caruaru* |
| A43 | *Mesabolivar spinulosus* |
| A43 | *Metazygia ipago* |
| A43 | *Predatoroonops dillon* |
| A43 | *Scaphiella murici* |
| A43 | *Scytodes saaristoi* |
| A43 | *Soesilarishius dromedarius* |
| A43 | *Spilasma baptistai* |
| A44 | *Chirothecia daguerrei* |
| A44 | *Freya chapare* |
| A44 | *Kupiuka vochysiae* |
| A44 | *Latonigena beni* |
| A44 | *Lycosa carbonelli* |
| A44 | *Mangora cercado* |
| A44 | *Tullgrenella guayapae* |
| A45 | *Alpaida cachimbo* |
| A45 | *Alpaida mato* |
| A45 | *Araneus matogrosso* |
| A45 | *Araneus xavantina* |
| A45 | *Berlandiella querencia* |
| A45 | *Chrysometa xavantina* |
| A45 | *Ilargus nitidisquamulatus* |
| A45 | *Leucauge argyroaffinis* |
| A45 | *Maenola braziliana* |
| A45 | *Mecynogea sucre* |
| A45 | *Menemerus delus* |
| A45 | *Mesabolivar xingu* |
| A45 | *Metazygia lagiana* |
| A45 | *Metazygia voxanta* |
| A45 | *Miagrammopes bambusicola* |
| A45 | *Saitis nanus* |
| A45 | *Steatoda bertkaui* |
| A45 | *Tmarus aporus* |
| A45 | *Tmarus littoralis* |
| A46 | *Alpaida jacaranda* |
| A46 | *Antillorena patapata* |
| A46 | *Avicularia gamba* |
| A46 | *Capeta tridens* |
| A46 | *Hapalotremus muticus* |
| A46 | *Homalometa nossa* |
| A46 | *Ianduba paubrasil* |
| A46 | *Lasiodora striatipes* |
| A46 | *Leprolochus mucuge* |
| A46 | *Metagonia diamantina* |
| A46 | *Neodiplothele leonardosi* |
| A46 | *Otiothops dubius* |
| A46 | *Predatoroonops anna* |
| A46 | *Predatoroonops blain* |
| A46 | *Pselligmus infaustus* |
| A46 | *Romitia bahiensis* |
| A46 | *Sumampattus quinqueradiatus* |
| A46 | *Thiodina pseustes* |
| A46 | *Tmesiphantes hypogeus* |
| A47 | *Alpaida oliverioi* |
| A47 | *Chiracanthops mandibularis* |
| A47 | *Corinna urbanae* |
| A47 | *Hamataliwa dimidiata* |
| A47 | *Nagaina berlandi* |
| A47 | *Paralophostica centralis* |
| A47 | *Pardosa v-signita* |
| A47 | *Teudis fatua* |
| A47 | *Thallumetus latifemur* |
| A48 | *Araneus castilho* |
| A48 | *Dossenus marginellus* |
| A48 | *Echemus inermis* |
| A48 | *Leucauge atrostricta* |
| A48 | *Predatoroonops schwarzeneggeri* |
| A48 | *Synemosyna magniscuti* |
| A49 | *Actinopus dubiomaculatus* |
| A49 | *Alpaida yucuma* |
| A49 | *Aphirape ancilla* |
| A49 | *Arachosia proseni* |
| A49 | *Araneus cohnae* |
| A49 | *Ariadna boliviana* |
| A49 | *Chira spinipes* |
| A49 | *Cleocnemis spinosa* |
| A49 | *Cotinusa rubriceps* |
| A49 | *Cyclosternum garbei* |
| A49 | *Enna segredo* |
| A49 | *Gayenna chrysophila* |
| A49 | *Isoctenus segredo* |
| A49 | *Losdolobus parana* |
| A49 | *Lygromma ybyguara* |
| A49 | *Mangora castelo* |
| A49 | *Mangora piratini* |
| A49 | *Mesabolivar botocudo* |
| A49 | *Mesabolivar maxacali* |
| A49 | *Metaphidippus coccinelloides* |
| A49 | *Micrathena soaresi* |
| A49 | *Nesticus ivone* |
| A49 | *Oligoxystre diamantinensis* |
| A49 | *Parabonna goffergei* |
| A49 | *Pholcomma mantinum* |
| A49 | *Phoroncidia tina* |
| A49 | *Psalistopoides emanueli* |
| A49 | *Scytodes marlieria* |
| A49 | *Thaumastobella mourei* |
| A49 | *Theridion bolum* |
| A49 | *Theridion cohni* |
| A49 | *Tmesiphantes perp* |
| A49 | *Tmesiphantes riopretano* |
| A49 | *Trochosa paranaensis* |
| A49 | *Vinnius paranaensis* |
| A50 | *Pamphobeteus nigricolor* |
| A51 | *Mesabolivar aurantiacus* |
| A51 | *Metagonia beni* |
| A51 | *Metagonia taruma* |
| A51 | *Scytodes lineatipes* |
| A52 | *Alpaida marmorata* |
| A52 | *Cryptachaea pussilana* |
| A52 | *Faiditus ecaudatus* |
| A52 | *Witica cayanus* |
| A53 | *Castianeira brevis* |
| A53 | *Hypognatha jacaza* |
| A53 | *Metazygia cazeaca* |
| A53 | *Odo similis* |
| A53 | *Sillus imbecillus* |
| A53 | *Teudis formosus* |
| A54 | *Goeldia zyngierae* |
| A54 | *Isoctenus areia* |
| A54 | *Lasiodora klugi* |
| A54 | *Onocolus echinurus* |
| A54 | *Scytodes itabaiana* |
| A54 | *Thaumasia diasi* |
| A55 | *Edilemma foraminifera* |
| A56 | *Anyphaenoides locksae* |
| A56 | *Iridopelma oliveirai* |
| A57 | *Loxosceles variegata* |
| A59 | *Scytodes sincora* |
| A60 | *Aillutticus raizeri* |
| A60 | *Dipoenata morosa* |
| A60 | *Metazygia corumba* |
| A60 | *Theridion urucum* |
| A61 | *Fufius funebris* |
| A61 | *Homoeomma humile* |
| A61 | *Mesabolivar delclaroi* |
| A61 | *Oligoxystre auratum* |
| A61 | *Olios lepidus* |
| A61 | *Olios vitiosus* |
| A62 | *Acanthoscurria sternalis* |
| A62 | *Corinna travassosi* |
| A62 | *Dyrines striatipes* |
| A62 | *Enoploctenus morbidus* |
| A62 | *Hogna travassosi* |
| A62 | *Isoctenus foliifer* |
| A62 | *Mirandia australis* |
| A62 | *Odo pulcher* |
| A62 | *Predatoroonops olddemon* |
| A62 | *Scytodes jyapara* |
| A62 | *Speocera eleonorae* |
| A62 | *Trachelopachys bidentatus* |
| A62 | *Zelotes zonatus* |
| A63 | *Stemmops belavista* |
| A64 | *Cyclosa turvo* |
| A64 | *Enna meridionalis* |
| A64 | *Losdolobus xaruanus* |
| A64 | *Lycosa leucotaeniata* |
| A64 | *Lycosa pintoi* |
| A64 | *Melocosa gertschi* |
| A64 | *Metazygia limonal* |
| A64 | *Paradossenus pozo* |
| A65 | *Architis neblina* |
| A65 | *Gephyroctenus atininga* |
| A65 | *Guadana neblina* |
| A65 | *Mangora manicore* |
| A66 | *Anisaspis tuberculata* |
| A66 | *Drymusa tobyi* |
| A66 | *Enna xingu* |
| A66 | *Sarinda capibarae* |
| A66 | *Scytodes championi* |
| A67 | *Dolichognatha kampa* |
| A67 | *Gephyroctenus acre* |
| A67 | *Gephyroctenus divisor* |
| A67 | *Pamphobeteus grandis* |
| A68 | *Acragas humaitae* |
| A68 | *Alpaida madeira* |
| A68 | *Eustiromastix efferatus* |
| A68 | *Helvibis germaini* |
| A68 | *Noegus mantovani* |
| A69 | *Hesydrus palustris* |
| A69 | *Lyssomanes taczanowskii* |
| A69 | *Micrathena mitrata* |
| A70 | *Eustiromastix macropalpus* |
| A70 | *Gelanor proximus* |
| A70 | *Hamataliwa argyrescens* |
| A70 | *Osericta dives* |
| A71 | *Attacobius verhaaghi* |
| A71 | *Soesilarishius cearensis* |
| A71 | *Soesilarishius macrochelis* |
| A72 | *Epicratinus pugionifer* |
| A72 | *Exechopsis conspicua* |
| A72 | *Meioneta propinqua* |
| A72 | *Zimiromus sinop* |
| A73 | *Scytodes ybyrapesse* |
| A74 | *Attacobius kitae* |
| A74 | *Hypognatha tocantins* |
| A74 | *Loxosceles puortoi* |
| A75 | *Scytodes xai* |
| A75 | *Temnida rosario* |
| A75 | *Tenedos carlosprestesi* |
| A76 | *Epeiroides lamprus* |
| A76 | *Hypognatha mirandariberoi* |
| A76 | *Saitis marcusi* |
| A76 | *Verrucosa lampra* |
| A77 | *Catanduba canabrava* |
| A77 | *Cetonana petrunkevitchi* |
| A77 | *Misumenops curadoi* |
| A77 | *Scytodes apuecatu* |
| A77 | *Scytodes becki* |
| A78 | *Actinopus nattereri* |
| A78 | *Ancylometes pantanal* |
| A78 | *Avicularia ochracea* |
| A78 | *Latrodectus mactans* |
| A78 | *Metepeira galatheae* |
| A78 | *Tetragnatha parva* |
| A78 | *Vitalius rondoniensis* |
| A79 | *Allocosa paraguayensis* |
| A79 | *Alpaida natal* |
| A79 | *Apodrassodes chula* |
| A79 | *Apodrassodes taim* |
| A79 | *Aysha taim* |
| A79 | *Carapoia crasto* |
| A79 | *Escaphiella bahia* |
| A79 | *Escaphiella maculosa* |
| A79 | *Eustiromastix nativo* |
| A79 | *Hamataliwa marmorata* |
| A79 | *Ibotyporanga diroa* |
| A79 | *Kupiuka paulista* |
| A79 | *Lapsamita maddisoni* |
| A79 | *Latonigena taim* |
| A79 | *Leptopholcus pataxo* |
| A79 | *Lyssomanes tapuiramae* |
| A79 | *Mastophora obtusa* |
| A79 | *Metazygia levii* |
| A79 | *Neocteniza australis* |
| A79 | *Ocrepeira macaiba* |
| A79 | *Oltacloea ribaslangei* |
| A79 | *Otiothops gounellei* |
| A79 | *Otiothops helena* |
| A79 | *Oxyopes macroscelides* |
| A79 | *Psilocymbium tuberosum* |
| A79 | *Soesilarishius tabernarius* |
| A79 | *Stemmops salenas* |
| A79 | *Yacolla pikelinae* |
| A80 | *Alpaida constant* |
| A80 | *Helvibis chilensis* |
| A80 | *Hypognatha solimoes* |
| A81 | *Coryssocnemis banksi* |
| A81 | *Hypognatha cacau* |
| A81 | *Metazygia vaupes* |
| A82 | *Architis altamira* |
| A83 | *Anapis discoidalis* |
| A83 | *Globignatha rohri* |
| A83 | *Symphytognatha brasiliana* |
| A84 | *Neocteniza myriamae* |
| A85 | *Gladicosa gulosa* |
| A85 | *Ibotyporanga naidae* |
| A86 | *Mangora bonaldoi* |
| A86 | *Mangora minacu* |
| A86 | *Martella goianensis* |
| A87 | *Isoctenus corymbus* |
| A87 | *Scytodes itacuruassu* |
| A88 | *Dipoena polita* |
| A88 | *Dubiaranea saucia* |
| A88 | *Mangora paranaiba* |
| A89 | *Cybaeodamus brescoviti* |
| A89 | *Gavarilla arretada* |
| A89 | *Nosferattus ciliatus* |
| A89 | *Nosferattus discus* |
| A90 | *Attacobius tucurui* |
| A90 | *Hesydrus yacuiba* |
| A90 | *Oligoxystre tucuruiense* |
| A90 | *Scaphiella capim* |
| A91 | *Exocora medonho* |
| A91 | *Lyssomanes romani* |
| A92 | *Acanthoscurria fracta* |
| A92 | *Avicularia rufa* |
| A92 | *Cyclosternum bicolor* |
| A92 | *Cyrtopholis palmarum* |
| A92 | *Psecas barbaricus* |
| A93 | *Mangora aripuana* |
| A93 | *Mesabolivar eberhardi* |
| A93 | *Scytodes skuki* |
| A94 | *Dossenus paraensis* |
| A95 | *Acanthoscurria transamazonica* |
| A95 | *Attacobius uiriri* |
| A95 | *Drymusa spelunca* |
| A96 | *Mesabolivar nigridentis* |
| A97 | *Catanduba piauiensis* |
| A97 | *Guaraguaoonops humbom* |
| A98 | *Loxosceles niedeguidonae* |
| A99 | *Soesilarishius bicrescens* |
| A100 | *Scytodes tapuia* |
| A101 | *Catanduba peruacu* |
| A102 | *Alpaida utiariti* |
| A102 | *Arocha erythrophthalma* |
| A102 | *Asaracus megacephalus* |
| A102 | *Catanduba araguaia* |
| A102 | *Chrysso antonio* |
| A102 | *Coleosoma floridana* |
| A102 | *Cryptachaea barra* |
| A102 | *Eupalaestrus weijenberghii* |
| A102 | *Freya champare* |
| A102 | *Grammostola pulchra* |
| A102 | *Guaranita munda* |
| A102 | *Ibotyporanga ramosae* |
| A102 | *Iridopelma marcoi* |
| A102 | *Longistylus ygopema* |
| A102 | *Meriola fasciata* |
| A102 | *Metagonia potiguar* |
| A102 | *Metazygia ituari* |
| A102 | *Metazygia redfordi* |
| A102 | *Neodrassex Iguatemi* |
| A102 | *Oarces ornatus* |
| A102 | *Onocolus perditus* |
| A102 | *Otiothops lajeado* |
| A102 | *Plesiopelma rectimanus* |
| A102 | *Runcinioides souzai* |
| A102 | *Scytodes adisi* |
